# Supplementary material for: Social support and very young adolescent girl’s knowledge on sexual relationships: A comparative qualitative study of Girl Only Clubs’ participants and non-participants in rural Malawi
Source: PLOS Glob Public Health. 2023 Jan 12;3(1):e0001339. doi: 10.1371/journal.pgph.0001339 (PMC10022037; doi:10.1371/journal.pgph.0001339)
Supplement: S1 Data — (ZIP) [file pgph.0001339.s003.zip › Anonymized/IDI_ND_XX_1.docx]

**Location**: YY_MI Village

**Date**: DD/MM/2020

**Time**: 9am

I: As I said earlier, I said my name is WWW, we are from COM, and we are conducting this study to find out how adolescent girls live, girls from ages of 10 to 15. Their ages can be 12, 13 but should not exceed 15. How old are you?

R: I am 14 years old.

I: So, first thing I would like to know about your school? Where do you learn?

R: I go to MA LEA school

I: In which class are you?

R: 6

I: Okay, what do you like at your school?

R: Subjects?

I: Yes, subjects that you like?

R: Mathematics

I: Why do you like mathematics?

R: I would like to work in the bank?

I: alright, what else do you like at school?

R: I also like life skills.

I: Why do you like life skills subject?

R: I like life skills because it teaches some other things.

I: What other things, I don’t know what topics are taught in Life skills. What do they teach in life skills?

R: They teach how to prevent and protect yourself from diseases.

I: How do you perform at school? Last term how did you preform?

R: I was on position 20

I: How many teachers do you have who teaches you?

R: 3

I: So, from the 3 who is your favourite?

R: Madam MP

I: Why is it that you like Madam MP?

R: She teaches very well

I: Other than the rest?

R: Mmm

I: Is there anything that is hard for you in class? Whether a subject that is hard for you or anything that is hard for you in class at school?

R: Social studies subject is the one that is very hard for me

I: Why is social studies hard?

R: [pause]

I: Is it just hard? Why is it difficult?

R: Because I don’t understand it properly.

I: What else is hard or difficult for you at school?

R: Concerning education?

I: Anything that is difficult, any problem you might have at school, what you think it you have concerns with it?

R: [long pause]

I: There are no problems? Everything is okay? Concerning education, where do you experience problems?

R: [pause]

I: Everything is okay, concerning school?

R: No

I: Mmmh

R: My friends sometimes steal my notebooks and agriculture subject is also a problem to me.

I: Okay, is there anyone who gives you trouble at school?

R: Maybe my friends

I: How do they trouble you?

R: Stealing my pens

I: that’s all?

R: Mmm

I: There is no one who troubles you at school?

R: Yes

I: Boys don’t give you troubles at school?

R: No

I: Okay. When you are at home, you stay with your grandparents, right?

R: Yes

I: When you wake up in the morning, what do you do?

R: I wash my face and wash plates, sweep the grounds and give my grandparents water to bath.

I: So, when you are not working here at home and you are not doing any school work, what do you do?

R: Sometimes I just sit and read, sometimes I play with ‘fulaye’ (game popular to girls).

I: How many friends do you have?

R: 10

I: Tell me their names?

R: UX, RX, Leah, GX AX, UX, SX, MX, HX, LX, DX

I: they are ten?

R: Yes

I: Alright, who are your loved friends amongssst them?

R: UX, LX and SX

I: These are your closest friends?

R: Yes

I: How are they different from the rest whom you didn’t mention?

R: The 3 , when I have problems, they do help me.

I: When you are saying that when you have problems, they do help you, what do you mean by that? In what aspect do they help you?

R: When I do not have soap, they share me 100 to buy soap.

I: From your friends?

R: Yes

I: Another problem, on which they help?

R: When I tell them that I am hungry they tell me to go home with them and cook.

I: What else?

R: When am sick, they are able to buy medication for me.

I: Are these your friends whom you share secrets with?

R: Yes

I: Which ones?

R: GX

I: And for girls here, which ones are secret stories that you share?

R: [pause]

I: Are you shy to mention the secret stories?

R: No

I: Examples of secret stories?

R: When we are talking about someone, and when we are talking issues of menstruation

I: Have you started menstruating yourself?

R: Yes

I: So, can you tell me what happened when you started menstruating, what did you experience, what happened as ritual and whom did you tell? Can you remember what happened that day?

R: I had stomach pains

I: Mmmh, what happened thereafter?

R: I told my mother that I had stomach aches.

I: Mmmh, what was next, I just want you to narrate it as a story that this what happened.

R: I had stomach aches and when I went to the bathroom to pee, I saw a drop of blood and I told my mother.

I: Why did you choose to tell your mother not any other person?

R: Because where I was, I was staying with her (my aunt)

I: You were staying with your Aunt?

R: Yes

I: And there was her, you were staying with?

R: I was staying with her and father

I: When did it happen?

R: Iiii

I: Long time?

R: Mmm

I: So, I would like to ask, before you experienced this, did you have prior knowledge about it? Before you went to the bathroom and saw that, did you have knowledge that this happens?

R: Yes, my friend told me

I: I would like you to remember and tell me, what were you discussing for your friend to reach to the point where you were discussing about this?

R: The discussion was that she said I have a book that talks about girls; and she said in this book they draw a girl and is written ‘a girl coming to age’ and I said yes I also have. And then she started explain all.

I: Where did you get the book, or where did your friend get the book from?

R: My relative gave me

I: The book called ‘a girl coming of age’ ‘*kukula kwa mtsikana’*?

R: Mmm

I: Can you explain to me; what is in the book?

R: Concerning period, concerning a cloth used as menstrual pad.

I: So, your friend was the first person to tell you that there is that book?

R: Yes

I: And you also told her that you also have it?

I: Now, tell me what you discussed later after that? I want to know that, if you were asking each other questions?

R: Yes, I asked her what it really was and she told me that it was ‘kugwa msinkhu’ ‘coming of age’. And I asked what she does, and she told me that they take a piece of cloth and we fold it and we wear it.

I: She didn’t say what happens? She just said that you take a piece of cloth and wear it?

R: She said that sometimes one has stomach ache on lower abdomen.

I: When you heard about this, how did you feel? Were you afraid or shy, how did you feel inside you?

R: I just leave it and wait to see what will happen

I: And that’s when you saw it happening.

R: Yes

I: And now, it has happened, and you’ve told your mother (aunt), what happened?

R: My mother explained to me what I will be doing things.

I: What did she tell you?

R: She told me that when I see this again, I should take the piece of cloth and wear it and I should also dress respectfully.

I: Apart from what your mother told you, what happened after that, was there anyone who came to advise you, it was just like that?

R: Yes

I: No one came to advise you after that?

R: No, they haven’t done that

I: Or your grandmother has never told you something?

R: No, they haven’t yet done that

I: It was from your mother, that is all?

R: Mmm

I: When your mother explained it to you, that was all?

R: Yes, I have just come here. I stay here, and I went to holiday there.

I: where does your mother stay?

R: My Aunt stays in LE.

I: Okay, after this did you tell your friend, or who else did you tell?

R: I told that same friend.

I: Did she experience this already or not?

R: She already experienced it.

I: So, when you told her, what did she say?

R: She just said okay

I: when you were at your mothers place; for the girls who has gone through this, they don’t have any gathering where girls come together and talk about it or they call someone to come and advise you?

R: I don’t go out I just stay inside the fence.

I: And since that time there is nothing that you have learnt or been told concerning period?

R: I was just told that I shouldn’t be busy with boys.

I: Who told you that?

R: My sister

I: What else did she tell you?

R: [pause]

I: why was the reason given as to not be busy with boys?

R: She told me that other boys are weed smokers and they can rape me and impregnate me.

I: Okay. You told me that you were staying with your aunt in Liwonde, do you have your real mother?

R: Yes

I: Where does she stay?

R: She has gone to Turn-off

I: Whom do you stay with?

R: My grandmother

I: Where does your mother stay?

R: That’s her house

I: Okay, is your father still alive?

R: Yes

I: Does your mother still live with your father?

R: No, they divorced

I: How many are you in your mothers house?

R: 12

I: In order of seniority, which number are you?

R: 8th, the first born is the one who passed here

I: The one who greeted us?

R: Yes

I: Where are the others? Tell me where each one of them is?

R: The first one is the one who has passed us here, the 2^nd^ is in CE married

I: A lady?

R: No, a boy, the 3^rd^ one is a boy who stays in LL with my uncle.

I: Mmmh

R: The other one is a girl married and stays in ME, and the other one was married in …. And the other one is that girl who has a child and is sitting there on whatsapp. And there another one who stays with uncle in BT and there is myself, and the other 3 stays with mother (aunt) in LE.

I: On the group of 12 children, did any one reached Form 4?

R: Yes

I: Who?

R: Iman

I: That’s your brother?

R: Yes, and the one who passed here

I: What does she do?

R: She just stays home

I: But she reached Form 4?

R: Yes. And also the other one who stays in ME

I: She also reached Form 4?

R: yes

I: What does she do?

R: At some point she was doing something, but now she just stays.

I: She just stays?

R: Mmm

I: Is there anyone of your siblings who is working somewhere?

R: The one in LL sells clothes

I: Alright, you were telling me that your parents divorced, but all of you have one father?

R: Yes

I: when did they divorce?

R: Long time

I: Where does your father stay?

R: At LK

I: what does your mother do?

R: Nothing

I: Oho, we were talking about issues of menstruation, what did your real mother tell you about it?

R: She told me to dress appropriately

I: You talked about your friends, did you meet them here in the village or at school?

R: Some I met them here

I: Okay, so when you are with your friends, when you are playing; what are the problems you meet as girls in this village?

R: Some insults their friends

I: Some girls insults their friends?

R: Yes

I: Some problems? What happens, things that happens?

R: there are some girls whom you would play with and chat with and you will talk of certain issues and they will go away and tell others about the issue. We would chat of non serious issues and one would take it to her parents who will now take the story to the other girls parents.

I: Alright. What else do you meet when you go to play?

R: There are some other boys who would just come where others are playing and just beat you up without issues.

I: Is there something that happened in your life that made you unhappy?

R: [pause]

I: You can tell me a few issues that happened and made you sad?

R: For example, I should lend clothes to a friend and because we no longer in terms, and then find that friend wearing the clothes and strip her of the clothes on the road.

I: Did it happen to you?

R: No, my friend

I: What happened to you and made you sad, whether at home or from friends, or at school?

R: In class, a friend just came and started insulting me when I didn’t know how I wronged her, and I was sad with that, and when I asked her she didn’t say the reason. That made me sad

I: Alright. And when you think o f you as a girl, can you tell me what you like as a girl, a good thing about you that you can speak of?

R: About myself?

I: Mmm

R: whether my body?

I: Mmm, you as a person.

R: I am proud because I speak English

I: You know how to speak English?

R: I do try

I: what else are you proud of apart from knowing how to speak English?

R: [pause]

I: I asked you that if you are not working at home what do you do and you said you go and play, what else do you do?

R: I come back home and cook and if there is nothing to do I just sit

I: Apart from going to play where else do girls go?

R: Us?

I: Yes, you said it is either home or where you go to play, or do you go to church?

R: We go Friday at Mosque

I: And when you are back to Mosque what do you do?

R: If it a market day we go to the market or go and play netball at the Dambo.

I: What do you do at the market?

R: When we have money, we go to buy clothes, and sometimes we just go and have walk there

I: Where do you get the money?

R: We do some piece works

I: So, the other things that you do is doing piece works?

R: Yes

I: Where do you go to do piece works?

R: If someone want you to sweep the house and give you K100, you will do that, and sometimes we work on the fields.

I: so they give you money?

R: Mmm and sometimes washing too.

I: And when you are walking, going for the piece works or to play, is there any bad that one can meet?

R: Maybe creatures like snakes.

I: Or being threatened by people like men? Not you, but other girls here what bad incidents do they meet?

R: there are other men who invite girls to go to their homes, and when girls ask why they just say that they will find out when they go. And girls usually say no, if you were staying with your wife I would have come.

I: Did it ever happen to you, that a man invited you to go to his house?

R: No

I: But you just hear that others are told?

R: Yes

I: Have you ever gone somewhere, walking and something bad happened to you?

R: No

I: Have you ever had anything in your mind that you wanted to share because it was troubling you?

R: [pause]

I: Maybe you have a problem and you would like to tell someone?

R: When I want to buy pads, I normally go and tell my granny that I don’t have thing like soap, and when she say she doesn’t have, then I just stay like that.

I: You said that with your friend you chat about menstruation, is there anything that you chat with her that you cannot talk with any other but you only talk just the two of you?

R: No

I: You said that you stay with your granny, why do you stay with her and not your mother’s home?

R: Granny took me when I was young.

I: Why did she take you?

R: She has legs problems and she took me so that I can help with work.

I: Alright, what is it that you like staying with your granny.

R: When I ask her something, when she has money she gives me and when she doesn’t have it she doesn’t give me.

I: What about those that you don’t like? I am not going to tell her?

R: when I boil water for someone and she tells me to go and put it in the bathroom, and I tell her that the water is not yet hot, I just get out of the place to wait for the water to boil.

I: Now, I would like to ask about HIV. Have you ever heard about HIV?

R: Mmm

I: Can you remember the day you first heard about HIV, the place, what was happening?

R: I heard about it at school

I: What was happening, were people just talking about it or it was in class?

R: Madam was teaching about it in class

I: What did she say HIV was?

R: She said that it was a virus that causes diseases.

I: What else did she taught?

R: She said that HIV is a virus that causes a lot of ailments

I: What else?

R: [pause]

I: Where else did you hear about HIV?

R: When we were playing with friends, they said that HIV is dangerous

I: You were discussing about it?

R: Yes. We were saying that other people contract HIV through sexual intercourse and sharing tooth brushes.

I: So, at first when you didn’t know much about HIV, what kind of questions did you have? What you wanted to know about HIV, or right now what are the questions you have about HIV?

R: At that time, I was asking myself what HIV was, but now, people said that HIV is virus that people contract.

I: Alright. Do you think girls of your age are at risk of contracting HIV?

R: Yes

I: Why are you thinking in that way?

R: When having sex and also sharing toothbrush

I: And when we are thinking of issues concerning HIV, they are secret issues, whom would you love to discuss such issues freely?

R: Upile

I: Why Upile?

R: I do chat with her very well

I: Okay. Have you ever heard about getting pregnant?

R: Yes

I: Where have you heard that before?

R: When we were playing.

I: Tell me what happened on that day?

R: There is a certain woman who was asking someone that a certain girl is pregnant, and the other girl said, ‘I also just heard that’, and they started talking.

I: What were they talking?

R: They said that she is very loose she is always with boys mostly and now she has been given a stopper.

I: When you heard about that what were you thinking?

R: [pause]

I: That was the first time to hear that people get pregnant, right?

R: Yes

I: Were you not surprised that what is pregnancy?

R: I was surprised

I: So, what did you do?

R: I came home and asked granny that what is it, and my granny told me

I: What else did you hear about pregnancy and where, at first you said where you were playing, where else?

R: Behind people were talking

I: What were they talking about?

R: They were saying, we should not get busy with boys they can impregnate you, and that you should see your friends who are giving birth while young.

I: Have you ever discussed with anyone how one can get pregnant?

R: Yes

I: With whom?

R: GX

I: From where?

R: A friend from there.

I: What happened for you to start discuss that?

R: She was saying that these other girls looks as if they get pregnant deliberately from what they do. Sometimes they don’t sleep in their homes, they sleep at boys places. And we started laughing and she also said instead of protecting themselves.

I: They should be protecting themselves?

R: Yes

I: So, how would one protect herself?

R: By using a condom

I: Another way of protecting yourself that you discussed?

R: [pause]

I: Okay, right now do you have questions that you would like to know about issues of getting pregnant and how one would protect themselves from getting pregnant, are there issues like that?

R: Yes, like how to protect yourself

I: So, whom do you like to discuss such kind of issues with? In your life whom you see can discuss with you?

R: My sister

I: Why didn’t you choose your granny, or mother or your friends?

R: My sister is the one who tells me a lot of things

I: I would like to ask; when a girl is growing she sees some changes in other parts of her body for example, breast grow and hairs and start menstruating. When you saw that your breast started growing, what did you think?

R: That I was growing

I: You didn’t ask that it was strange?

R: No, I didn’t ask

I: You didn’t ask because you thought that you were growing?

R: Mmm

I: Alright. We are about to finish. Have you ever heard that a girl has a child, or a girl is pregnant? Have you ever discussed with any one what happens for a girl to go and give birth? Concerning marriage?

R: No

I: You’ve never discussed with anyone concerning antenatal clinic?

R: No

I: Alright, can you tell me, have you ever heard about girl and boy relationship?

R: Yes

I: Where did you hear it from?

R: My friend discussing it

I: What were they saying?

R: they were saying that such such a girl goes out with such such a boy, and such a girl are fond of each other with such such a boy.

I: So, have you ever sat down and discussed such kind of relationship with someone about such relationships? Whether is your friend Upile to discuss what happens?

R: Yes

I: Whom did you discuss with?

R: GX sometimes ….

I: What did you discuss with GX?

R: She was saying that there is a relationship that is like a real relationship and there is another that they help each other and teach each other good things.

I: In this community, how common is it to find relationship between a girl and boy?

R: Not very common

I: They are a few who has relationships in this community?

R: Yes

I: Alright, do you have any question that you would like to ask on the issue of relationship between a girl and a boy?

R: Yes

I: Like what?

R: There is a type of relationship where they would wash clothes for each other, giving each other money and doing a lot of things and I get to wonder about such relationships.

I: Does it happen frequently? You just hear about it, right? That they give each other money?

R: Yes, and some fetch water for each other.

I: And when that is happening, what do parents say?

R: Other girls are from afar

I: So, they come to fetch water for them?

R: Yes

I: So, what is it that you wonder?

R: That I have never seen them talking but I see the girl coming to fetch water for the boy.

I: What else?

R: [pause]

I: Has a boy ever proposed to you?

R: Some boys just say that they want to be playing with me.

I: But not proposing to you?

R: No

I: No one ever proposed?

R: yes?

I: Alright, do you have anything that you would like to tell me, concerning you, that you would want me to know?

R: [long pause] There are other boys who threatens you when you tell them that you don’t want to play with them, and they can tell you that you shouldn’t pass on such such a place.

I: They say that they want to play with you, so why do you say no?

R: Some smoke weed and some drink beer.

I: What else?

R: [long pause]

I: When they threaten you that they will beat you, where do you go and report?

R: Would go to granny and tell her or go to his mothers place and tell her that your son told me that he will beat me.

I: And what happens?

R: Granny tell me that if he beats you come and tell me.

I: Is there anything that you would like to tell me?

R: [long pause]

I: Do you go to clubs?

R: No, I want to join a youth club.

I: But right now, you haven’t joined yet?

R: Yes

I: Thank you

R: Welcome.

END
