# Supplementary material for: Social support and very young adolescent girl’s knowledge on sexual relationships: A comparative qualitative study of Girl Only Clubs’ participants and non-participants in rural Malawi
Source: PLOS Glob Public Health. 2023 Jan 12;3(1):e0001339. doi: 10.1371/journal.pgph.0001339 (PMC10022037; doi:10.1371/journal.pgph.0001339)
Supplement: S1 Data — (ZIP) [file pgph.0001339.s003.zip › Anonymized/IDI_ND_XX_10.docx]

**Location**: XX-MI Village

**Date**: DD/MM/2020

**Time**: 11:15 pm

I: What’s your name?

R: HF

I: How old are you HF?

R: 12

I: Alright, do you go to school?

R: Yes

I: In which class are you?

R: 5

I: Where do you go to school?

R: XX LEA school

I: What makes you happy at your school?

R: What makes me happy when am going to school?

I: Yes, what encourages you to go to school and makes you happy?

R: I would love to finish school

I: When you’ve finished it, whom do you want to become?

R: I would like to be a doctor.

I: Good news. Which subjects do you put an extra effort on, for you to become a doctor?

R: Science

I: And what subjects do you do better in class?

R: Science

I: What about mathematics?

R: Mmmm, mathematics tend to be tough a little.

I: English?

R: I do pass

I: What position did you get last term?

R: 5

I: Why didn’t you scoop position 1?

R: I stayed for some days without going to school?

I: Why?

R: I didn’t have soap

I: If you were to change one thing at your school, something that you would want it to be different, what can it be?

I:

R: About education?

I: Mmm

R: Nothing

I: What are the problems that you meet when you are going to school?

R: Lack of school fees, and lack of money, uniform, shoes.

I: Whom do you stay with?

R: I stay with my aunt. (mom’s sister)

I: Where is your mother?

R: At LW

I: Is your dad alive?

R: Yes

I: Why don’t you stay with your parents?

R: They took me in because at LW we were staying in harsh condition.

I: How many children are you in your family?

R: 6

I: Who is the eldest?

R: CA

I: And yourself?

R: Am the 2nd born

I: Where are the other children?

R: Still there.

I: And what about CA, is she staying with your parents?

R: Yes

I: Alright, and here you stay with your aunt, and who else?

R: CWW

I: Who is CWW to you?

R: My relative

I: Is there anything that makes you unhappy while you are staying here?

R: No

I: Alright, what is it that makes you happy staying here with your aunt and your relatives?

R: They encourage me about school

I: Alright. Do you have friends?

R: A lot

I: Who is your best friend?

R: AX

I: Why do like AX?

R: Because we encourage each other about school?

I: She also goes to school?

R: Yes

I: In which class is AX?

R: 5

I: So, when you’ve finished your chores at home, where do you like to go?

R: To Madrassa

I: What do you learn at Madrassa?

R: Quran, Serra, Tawhid and Dower

I: Dower is a prayer, right?

R: Mmm

I: Alright, what else do you learn at Madrassa?

R: Quran

I: You learn word of God, right?

R: Mmm

I: What is the importance of going to Madrassa?

R: You learn a lot of things

I: Alright. Apart from Madrassa, where else do you go to play?

R: Here at home

I: Where else?

R: That’s all

I: You don’t go to play with friends?
R: I do go

I: Okay

R: I don’t go frequently.

I: Why do you mostly stay at home you don’t go out very often?

R: I don’t like roaming around.

I: Why? And what are your fears?

R: I feel like if one roams around might meet a problem

I: Have you heard that a girl was roaming around and has met a problem, or girls here when they go out roaming around they meet problems?

R: I have never heard of something like that.

I: Alright. Where was the first place where you heard about HIV?

R: At school

I: Explain to me, was it in class or you were with your friends?

R: In classroom

I: You were being taught?

R: Mmm

I: What did the teacher said?

R: HIV is AIDS

I: Mmmh

R: It is a dangerous disease

I: That’s all?

R: I have forgotten the other part

I: Where else did you learn about HIV?

R: Only at school

I: Do you learn about such things daily or there was a time when you learnt about it, or which subject has HIV/AIDS topics?

R: [long pause]

I: Did the teacher just started talking about it or it was a subject?

R: It was a subject

I: Which subject?

R: I think Social

I: I wanted to know, you as a person whom have heard things about HIV, do you have questions, or do you have a desire to know more about HIV?

R: Yes

I: Where do you want to hear it from or from who?

R: [long pause]

I: You want to hear it from school?

R: Mmm

I: From your teacher?

R: Mmm

I: With your from Liwonde or here, have you ever talked about HIV or discuss with you about HIV?

R: Mmm

I: How did it start, just share with me as a story where it started from, for them to start discussing this with you?

R: [long pause]

I: What happened?

R: They called us all together and told us that we should not be walking recklessly, because in doing so we can contract HIV through sex, and illness is dangerous.

I: That was your mother, here?

R: Mmm

I: What happened, did she just call you and start giving you advice or there was something that happened?

R: [long pause]

I: What happened?

R: There was something who was reckless and people started talking about her, and that what made her advise us.

I: Your relative?

R: Yes

I: Have you already started period or not?

R: Yes

I: I want to know, before starting your period, did you know about menstruation or you just found it out the day it started?

R: We were told at school?

I: Before you started it?

R: Mmm

I: Did they tell you in class?

R: No, they called all the girls from Standard 5 upwards, to go in class and they started telling us that.

I: Who called you?

R: Madam

I: Madam from your school?

R: Yes, with other people.

I: Where were the other people from?

R: I think from CM

I: There were the ones who told you what girls go through?

R: Mmm

I: So, can you tell me what happened on the day you had your first period?

R: I was sleeping and I just wake up with it.

I: What really happened, whom did you tell, as your story?

R: [pause]

I: What happened when you woke up during that night for other people to know that, this has happened to you?

R: [long pause]

I: What happened?

R: [long pause]

I: What happened?

R: When I woke up in the morning, I told my mother

I: So, what did your mother say?

R: [long pause]

I: Was it in Liwonde?

R: Here

I: When did it happen?

R: Saturday

I: This same year?

R: Yes

I: So, what did she say?

R: All the girls do that and for people not to know you need to wear pads.

I: And she gave pads?

R: Mmm

I: How did you feel when you woke in the midst of the night, were you afraid or had feeling of shame when you saw that?

R: I wasn’t afraid since we were told at school.

I: Okay, have you ever heard about pregnancy, how people get pregnant, or how you can avoid it, and if you heard about this, where and who was talking about it?

R: At school

I: Was it a lesson or what was happening?

R: It was a lesson

I: So, what was it all about?

R: When you want to avoid pregnancy, you need to use condoms.

I: Is there any other place where you learnt about this?

R: No

I: It was only at school?

R: Mmm

I: Is there any other place where you heard such issues?

R: No

I: It is only at school?

R: Yes

I: As a girl when you are growing up, you find out that some of things are changing on your body, such as breast growing, hair growing in some parts; have you ever discussed such thing with friends?

R: No

I: Whom would you want to discuss this with and find answers blissfully?

R: [pause]

I: You don’t want to know more?

R: I do

I: Whom would you want to discuss this with?

R: Parents

I: What is it that makes it difficult to discuss it with parents?

R: [long paus]

I: Alright. Have you ever heard about girl/boy relationships in this community?

R: yes

I: Why do you think most girls do that?

R: [long pause]

I: Why?

R: Some would want to drop out of school.

I: Why would they want to drop out of school?

R: [long pause] some lack assistance

I: Have you ever discussed issues of girl/boy relationships?

R: No

I: Do you have friends?

R: [pause]

I: It doesn’t happen to chat with friends?

R: My friends are young.

I: They are young?

R: Mmm

I: So, they have not yet started discussing such issues?

R: Mmm

I: Are there things that you would like to know about such issues?

R: No

I: Thank you very much for answering such questions, are there things that you would like to tell me, things that maybe I didn’t ask, or something that you have though of telling me about you?

R: Telling you or asking you?

I: You telling me, don’t go back with words.

R: I would like you to encourage me about education

END
