# Supplementary material for: Social support and very young adolescent girl’s knowledge on sexual relationships: A comparative qualitative study of Girl Only Clubs’ participants and non-participants in rural Malawi
Source: PLOS Glob Public Health. 2023 Jan 12;3(1):e0001339. doi: 10.1371/journal.pgph.0001339 (PMC10022037; doi:10.1371/journal.pgph.0001339)
Supplement: S1 Data — (ZIP) [file pgph.0001339.s003.zip › Anonymized/IDI_ND_XX_12.docx]

**Location**: XX-MI Village

**Date**: 8/10/2020

**Time**: 12:15 pm

I: What is your name?

R: SI.

I: SI?

R: Mmm.

I: Alright. How old are you?

R: I am 13 years old.

I: You are 13 years old?

R: Mmm.

I: Alright. Do you go to school?

R: Yes, I go to school.

I: What class are you?

R: 5

I: You are in standard 5?

R: Mmm.

I: Alright. So tell me, which subjects do you like/love at school?

R: English, Mathematics, Religious Studies, Social studies and Chichewa.

I: Alright. Are there some subjects which you struggle with?

R: Mmm, so much.

I: What are they?

R: I struggle with English, mathematics.

I: So, what do you do to pass?

R: I stay close to the person that is good at them in class and ask them to help me.

I: Mmm, okay. So you ask them to help you and it works?

I: Mmm, it works.

I: Are going in standard 6 now?

R: No

I: You are going in standard 6 or you are repeating standard 5?

R: I am repeating.

I: You are repeating standard 5, right?

R: Yes.

I: Mmm. Alright. So how many teachers do you have?

R: Mmm?

I: Teachers

R: 3

I: 3?

R: Mmm.

R: Mmm. Which teacher among the 3 do you like?

R: Mr X…….

I: Why do you like Mr X…

R: He/She teaches well and I listen very well when he/she is teaching

I: You understand what he/she teaches?

R: Mmm.

I: Alright, is there anything that disappoints you at school?

R: Mmm.

I: What is it?

R: Sometimes in class I am asked to answer a question and when I fail my friends laugh at me and it disappoints me.

I: Mmm. That’s the teacher?

R: Mmm.

*I: Mmm, so what does the teacher say when they are laughing?*

R: He/she may stop them and when they stop and on break time they laugh at me and it disappoints me.

I: Mmm, what else disappoints you?

R: Sometimes when I go to the market and make a mistake they shout at me when I come back and it disappoints me.

I: Alright. Who do you stay with?

R: I stay with my aunt.

I: Your aunt, the one that we found is your aunt?

R: Yes

I: Where is your biological mother?

R: She is behind that other house.

I: She stays in the other house?

R: Yes

I: Why don’t you stay with your mother?

R: It’s just like that.

I: Is your father around?

R: No.

I: Your father is dead?

R: Mmm.

I: So your mother stays alone?

R: She stays with my step father

I: So when you are staying at home you said sometimes they shout at you, what else disappoints you? The thing that you are not happy with, that you see that when it can change your life will be better?

R: Sometimes I may do everything but when I just do one mistake, she shouts at me and I want if it can change, that if she can just tell me nicely without shouting.

I: Alright. What do you do when you came back from school and finish doing everything?

R: I may read books.

I: Mmm. Do you have close friends that you chat with?

R: Yes I have.

I: How many?

R: 3

I: Mmm. Who and who?

R: AX, VX and CX.

I: Those are your friends that you rely on?

R: Mmm.

I: Why do you trust them?

R: They encourage me in many things.

I: They encourage you in many things like what?

R: When I fail, they encourage me to study and not to get discouraged.

I: Why do you fail? Is it that you don’t understand when you study or what happens?

R: I have a hearing disorder/problem and I also have a sleeping problem, I sleep frequently in class.

I: Mmm. Have you ever discussed this with your aunt?

R: Yes, she knows.

I: She knows?

R: Mmm.

I: Mmm. How about when you are with your friends, is there anything that disappoints you when you are with your friends?

R: No

I: There is nothing that ever disappointed you?

R: Mmm.

I: Alright. Do you face any challenges? Which places do you visit when you are playing?

R: Sometimes here in the village, when I have gone for chatting/playing

I don’t know that it’s I will start period/menstruation and I may stain my clothes and my friends just laugh at me. I lack materials like pads.

I: How about other methods since there are pads and things like pieces of clothes? How about that?

R: I may have but sometimes the period is heavy and the piece of clothe can’t stand it/come out.

I: It comes out?

R: Mmm.

I: And then you friends laugh at you?

R: Yes.

I: When did you start your menstruation?

R: On 1^st^ January

I: This year?

R: 2 years ago.

I: Mmm. Before starting menstruation, did you know anything about menstruation?

R: I didn’t know.

I: Alright, so tell me what happened the first day your menstruation started?

R: My friends went to chat at night and I entered in the house to sleep, there was no one at our home so I went inside and slept and when I was sleeping I wondered why I had a stomachache and headache, my underwear was wet but me beddings were dry so I woke up and went to the bathroom and saw blood so I told my parents and they said that was maturity so I didn’t know anything so I wanted to through away the underwear and they told me not to throw it away but rather wash and use it

I: Was that your mother? The parent that you told?

R: My mother.

I: Your biological mother or aunt?

R: Aunt.

I: Mmm. So what else did she counsel you?

R: That I shouldn’t chat with boys, I should chat with my friends whom also know about this that I should I should be telling them so that they may help me in some things.

I: Why did she said you should chat with boys?

R: Because when I am with a group of boy it the period can come/flood out and the boys would laugh at me.

I: Mmm, anything else? That was all?

R: Mmm.

I: I also wanted to know, when this had happened to you did you only get information from your aunt or did she invite over other people to counsel you?

R: Yes she invited them.

I: She invited other people?

R: Mmm.

I: Other people from where?

R: The grandmother in this village invited me and explained to me.

I: Things to do?

R: Mmm.

I: When you saw that it had happened like that what did you feel in your heart? Did you feel shy or fear? What was happening?

R: Fear.

I: You were afraid?

R: Mmm.

I: What did you perceive?

R: Sometimes I had bad thoughts about pregnancy and I was wondering.

I: That you are pregnant?

R: Mmm. So I was wondering, it made me afraid.

I: So how did you come to think about pregnancy? Had you already started sex?

R: No. I was just having bad thoughts.

I: That maybe that’s how a pregnancy comes?

R: Mmm.

I: Mmm. So now that it happened, do you know everything as to how it happens?

R: Yes I know.

I: Or is there anything that you still wonder and want to know things?

R: No, I know.

I: Okay. I wanted to know what happens if you have heard or ever discussed with someone about relationships between boys and girls?

R: Relationship between boys and girls?

I: That people are in a relationship and maybe you have ever discussed with your friends about what is involved in the relationships or if you have ever discussed with an old person or at school or somewhere else?

R: At school before I had reliable friends and when I was befriending everyone so some of my friends had boyfriends and they said I shouldn’t befriend them until I find myself I boyfriend then I can be their friend.

I: Mmm.

R: I was worried that i sometimes went go to school without eating, I could just take a bath without and end up not applying lotion and went to school so my friends were laughing at me that I should find a boyfriend that will be giving me money to but every material needed in my life so they didn’t allow me to be their friend.

I: So how did you deal with that?

R: I was telling my parents.

I: So what did your parents answer?

R: They said I shouldn’t be carried away with that, that they would try their best.

I: Mmm.

R: My parents do business in CE and sometimes they buy soap.

I: Mmm. Alright. Apart from your friends telling you to do this and find soap, is there anywhere you heard stories about that? Maybe the advantages and disadvantages of having these relationships? The advantages and disadvantages maybe if you discussed with someone either parents or your aunt, mother or your other friends in this village? Have you ever discussed if there are advantages and disadvantages?

R: Yes.

I: Mmm. Tell me, how how did it go?

R: The disadvantage of having a relationship is that one maybe forced by her partner to have sex and maybe she also wants, one ends up destroying her future by getting pregnant. In other relationships other partners don’t think about that, they help each other with school only.

I: Have you ever had a relationship?

R: No.

I: Alright, how about being approached by a man or your fellow youth?

R: Mmm.

I: They approach you?

R: Yes.

I: Explain to me, how did they approach you? What happened?

R: I help my grandmother sell tomato at CE so someone may come at my selling place wanting to buy tomato and I would serve him but before he gets going he proposes me and when I refuse he starts scaring me that they would do something bad when I will be going home so sometimes I get scared and I sometimes sleep over at my grandmother’s friend.

I: You fear that they would do something bad to you?

R: Mmm.

I: Are these people from there that you know them or they just come?

R: I may know some and not others.

I: How many times has this happened to you?

R: Twice.

I: What happened the second time?

R: The second time I also slept over.

I: At the same selling place?

R: I was sleeping at someone’s house.

I: No, I meant where they approached/propose you the second time?

R: At the bus stop.

I: It was at the bus stop.

R: Mmm. When I was getting off to do business boy that sell chips called me and I refused and he run after me and told me that he loves me and that he wants to be in relationship with me so I refused and he told me the same.

I: You said you do tomato business?

R: Yes

I: Where your mother sells tomato at CE?

R: Yes

I: And sometimes you go?

R: Yes.

I: Mmm. Is it during school time or after you knock off from school?

R: During holiday.

I: This Covid-19 holiday it’s when it happened?

R: Mmm.

I: How about boy like that in this village?

R: Boys in this village just approach me and I refuse, that’s all.

I: You just refuse?

R: Yes, it just ends there.

I: It just ends there?

R: Mmm.

I: Do you have friends who have boyfriends?

R: Yes.

I: Mmm. So don’t you admire them that they have and you don’t?

R: No, It doesn’t bother me.

I: Alright. I wanted to ask about HIV/AIDS. Where have you heard about HIV/AIDS?

R: I hear when I am learning at school.

I: Cn you remember the first time to hear stories about HIV/AIDS?

R: No

I: You don’t remember?

R: Mmm.

I: But where did you hear?

R: At school?

I: Can you explain to me how you heard at school? What happened for you to hear?

R: We were learning and they said if one touches a boy, one may get AIDS and when someone reuses a lazer blade which was initially used by an HIV patient one also gets it.

I: Apart from school, where else did you hear about HIV?

R: I have forgotten.

I: You have forgotten?

R: Mmm.

I: Don’t you talk about HIV/AIDS when you are with your friends?

R: No, am not much envolved with my friends, sometimes am at madrasah.

I: Oooh, you are mostly at Madrasah?

R: Mmm.

I: Don’t they talk about HIV/AIDS at the Madrasah?

R: They talk. Also about boys

I: About boys also?

R: Mmm.

I: What do they say? Can you explain to me?

R: We learn that when one is in contact with a boy, one may get AIDS.

I: Contacting like this?

R: No, sex. One may get AIDS.

I: Mmm. What else did they teach at the Madrasah about this issue of relationship and AIDS?

R: That when people are in a relationship and they don’t know each other’s status, they end up having unplanned and unprotected sex and also get AIDS.

I: Alright. Have you ever heard how one can prevent or get pregnancy? Have you ever learnt about how one can get or prevent pregnancy either at school or discussing with your friends?

R: Mmm.

I: Where? At school?

R: With my friends and at school as well.

I: So what do you discuss about issues of getting pregnancy?

R: If someone wants to prevent getting pregnancy there is need to use necessary method when having sex with a boy.

I: Necessary method?

R: Mmm.

I: What do you mean by necessary method?

R: Using condoms

I: Then one can’t get pregnant?

R: Mmm, people say like that.

I: What else do people say about getting and preventing pregnancy?

R: Restraining from boys, abstinence. …………With boys

I: Alright. Have you ever heard about child-bearing/motherhood, going for prenatal visits, delivering a baby and other things? Have you ever discussed with anyone? Or friends?

R: One delivers painfully at the hospital when she gives birth while still young because it happens that maybe the womb is not yet mature so when one starts giving birth early, she delivers painfully.

I: Whom did you discuss this with? Or where did you learn this?

R: With my friend…….

I: What prompted/made you to start talking about this?

R: My friend has a baby so she was explaining to me not to rush into boys since am still young and giving birth is painful and that my womb is not yet mature and that she struggled during giving birth.

I: Alright. Do you have many friends who have babies?

R: No, there are not many.

I: Mmm. Alright. Thank you so much, there are not many questions or maybe do you have anything that you can tell me, anything that I didn’t ask you but you would love to tell me?

R: Mmm.

I: Tell me.

R: Sometimes they send me back home from school because of school uniform and I sometimes stay for one week without going to school because of uniform and sometimes fees.

I: Mmm.

R: And I sometimes do go to school when I start menstruation because sometimes, I bleed heavily.

I: Mmm. About fees, can’t your aunt source fees? I mean is it difficult for her to find fees?

R: She struggles to find fees.

I: And also the same with uniform?

R: Mmm. Sometimes I fail to write exams because of fees.

I: Mmm. What class are you again? Standard5?

R: 5.

I: Do you pay for exams here?

R: Mmm.

I: Mmm. Did CAMFED organization not come here? The one that pays fees for learners and also distribute pads?

R: It came but they choose some people.

I: Okay. Thank so mu for your participation in answering these questions. I think I have asked every question that I was supposed to ask.

R: Alright.

I: Sure, thanks.

END
