# Supplementary material for: Social support and very young adolescent girl’s knowledge on sexual relationships: A comparative qualitative study of Girl Only Clubs’ participants and non-participants in rural Malawi
Source: PLOS Glob Public Health. 2023 Jan 12;3(1):e0001339. doi: 10.1371/journal.pgph.0001339 (PMC10022037; doi:10.1371/journal.pgph.0001339)
Supplement: S1 Data — (ZIP) [file pgph.0001339.s003.zip › Anonymized/IDI_ND_XX_2.docx]

**Location**: XX-MI Village

**Date**: DD/MM/2020

**Time**: 3pm

I: Now, tell me your name.

R: MC

I: Alright. Do you go to school?

R: Mmm

I: What is the name of your school?

R: XX

I: XX secondary school?

R: Yes.

I: XX secondary school?

R: No, LEA school.

I: Alright.

I: How old are you?

R: 12

I: Mmm. So tell me more about school. What do you like/love at your school?

R: I like/love mathematics, English and Chichewa subjects.

I: Why do you like these subjects?

R: It depends on intelligence.

I: Mmm?

R: It depends on intelligence.

I: When you say intelligence, do you mean you have more skills on these?

R: Yes, especially mathematics.

I: You do well in mathematics? You don’t do well in others?

R: Yes.

I: Mmm. How about another thing that you like, that which encourages you go to school, what is it?

R: Exams and learning.

I: Mmm. You also like exams?

R: Mmm.

I: Do you go to school so that you write exams?

R: No, and also to learn.

I: You want to learn?

R: Mmm.

I: How many teachers do you have?

R: Mmm?

I: How many teachers do you have?

R: There is one teacher and one male teacher

I: You have one teacher?

R: Mmm.

I: Mmm. You only have one teacher?

R: Mmm.

I: What did you say is your grade?/class?

R: 2

I: You are in standard 2?

I: You are in standard 2. What did you say is your age?

R: 12

I: Alright. So when you are at home, what do you to do?

R: I wash dishes, draw water and sweep the house.

I: Mmm

R: And cook.

I: Mmm. Okay. What do you do when you have finished cooking?

R: I bath the children and go to play.

I: You bath the children and go to play?

R: Mmm.

I: How many children are you in your family?

R: 3

I: Who is the firstborn?

R: I am the firstborn, the second born is XX who is 9 years old, the third born is 6 years old.

I: The one that was here?

R: Mmm

I: Mmm. So you bath HX and the one that was here?

R: I bath this one, the other started to bath herself.

I: The other started to bath herself, the 9 year old?

R: Mmm.

I: Mmm. Is HX also a girl?

R: XX not HF

I: XX?

R: Mmm.

I: Is a boy?

R: Mmm

I: Do you stay with your mother and father?

R: Yes.

I: Both of them are around?

R: Yes.

I: Alright. What does your father do to earn money?

R: Let me just say my other father in MJ died

I: Mmm

R: He used to do tobacco farming?

I: Mmm

R: So he was making a lot of money so he died in an accident.

I: He died, he had an accident in MJ?

R: Mmm.

I: Ooh sorry. Long time ago?

R: Yes, 2017.

I: 2017. So this is your step father?

R: Mmm

I: He is your step father?

R: Mmm.

I: So I want to know, is XX and you from the same father or you are the one whom your father died?

R: All of us, XX, XH

I: Also this one?

R: Mmm

I: Okay. So there is no child from this father?

R: Yes

I: There is no child with your mother?

R: Yes.

I: When did he get married to your mother?

R: Around 2018.

I: 2018?

R: Mmm

I: So I wanted to know how you live with your mother and step father.

R: Mmm

I: Whom do you open up to/feel comfortable to very much?

R: My mother.

I: You feel comfortable with your mother?

R: Mmm

I: Mmm. How is the relationship between you and your step father?

R: It is okay.

I: It’s not that I will tell your mother, she won’t listen to these. So don’t be shy to respond. I want you to tell me how you stay because others experience other things when they stay with their step father. How do you stay with him?

R: We stay well with him.

I: Mmm. What does this father do?

R: He does piece work.

I: He does piece work?

R: Mmm

I: Alright. Now, what does your mother do?

R: She just stays.

R: Okay. Do you have friends whom you chat/play with?

R: Mmm.

I: How many friends do you have?

R: Around 4

I: Mmm. Tell me their names.

R: The first is PX, the second is SX, the third CX, the fourth is NX, the one who is behind. The one that came here. The firth is JX and the sixth VX from AX.

I: Mmm. Among your friends, who is your best friend?

R: VX.

I: VX. Why are you friends with VX?

R: Because she doesn’t gossip, she does fight and is not two timing.

I: What else?

R: That’s all.

I: That’s all. Alright. What do you like to do when you are with your friends, the likes of VX, PX, JX?

R: We like to do masanje.

I: Masanje?

R: Mmm

I: What else?

R: We do ……. Shoe and play ball (fulaye).

I: You play Fulaye?

R: Mmm

I: Where else do you play?

R: That’s only where we play, here.

I: Do you only play around here, you don’t go elsewhere far?

I: What are the challenges that you face as girls when you are playing?

R: Nothing

I: There is nothing disappointing? Have you ever been disappointed in your life?

R: Mmm.

I: You have been disappointed before?

R: Mmm

I: What disappointed you?

R: People like to talk and they were gossiping and when they were gossiping, they were also name calling me on something that did not concern me.

I: Mmm.

R: They were name calling me saying that they will beat me up yet it doesn’t concern me so I told them to come and beat me but they did not beat me and I got disappoint and ask myself why would they want to beat me while I was not involved and I got disappointed for their name calling.

I: Mmm. How about when you are home, what disappoints you? Everyone gets disappointed at home, what disappointed you at home?

R: When I do a mistake, they shout at me.

I: Your mother?

R: Mmm.

I: Mmm. What else?

R: Nothing.

I: How about when you are in class at your school, what disappoints you?

R: There is nothing that disappoints me.

I: There is nothing that disappoints you at school?

R: Mmm.

I: Everything is smooth?

R: Mmm.

I: Alright. I wanted to know, among your friends that you have mentioned, you said your best friend is VX?

R: Mmm.

I: What kind of stories you talk with VX? When someone has a best friend they sometimes talk about their secret stories, what are the stories that you talk about?

R: We were discussing to have a party.

R: Mmm?

R: We were discussing to have a party and we didn’t do.

I: Party?

R: Mmm.

I: Mmm. What did you want to cerebrate at the party?

R: Just to cerebrate.

I: So what things that you planned to do on this party?

R: We wanted to have a party and buy different things, gifts.

I: Mmm. The gifts to give who?

R: People, those that are written. It depends on which gift they mention. Some mention four plates, some mention jags

I: Mmm.

R: Some mention small basins used to wash hands others basin for babies. There are many, others mention shoes.

I: Mmm. So what happens when you are having the party? All those money for party where do they come from? Do people contribute or what happens?

R: Parents contribute.

I: Parents contribute?

R: Mmm

I: Mmm. So someone receives the money and goes to buy the things?

R: Mmm.

I: The one who planned the party? For example you and VX would have received the money?

R: Mmm.

I: So where would you buy the gifts?

R: We didn’t do.

I: Had it been it happened, where would you buy the gifts?

R: At the turn-off.

I: At the turn-off, alright. Which turn-off, LE?

R: Mmm. Few meters from here.

I: Alright. What are other secretes that you discuss?

R: Nothing.

I: Alright. I wanted to know, did you start menstruating/period?

R: No, I haven’t starting menstruating.

I: Alright, but have you ever heard about these stories, that people menstruate or do initiation, haven’t you ever heard about it?

R: No.

I: Mmm. You haven’t discussed even with your friends?

R: Yes.

I: Alright. How about stories concerning HIV? Have you ever heard about HIV?

R: I haven’t.

I: You haven’t? At school…

R: I hear from the radio but I haven’t heard from school.

I: You hear from the radio but you haven’t heard from school. What do they say about HIV from the radio?

R: That someone has a virus and maybe about diabetes/sugar and fats. I don’t know about the others.

I: Alright. Perhaps, do you go to any club?

R: Club?

I: Mmm.

R: ……….

I: You haven’t gone to a club.

R: Mmm.

I: Alright. Didn’t you have questions about some things in your heard when you heard from the radio about HIV that you wondered if you could hear more?

R: I asked Patricia what the virus is and the said its HIV. She didn’t tell me about sugar and fats.

I: Mmm. What other things about HIV did Patricia say?

R: She said someone gets sick from HIV and dies when stops taking medication.

I: That one gets sick from HIV and dies when stops taking medication?

R: Mmm.

I: What else did Patricia tell you about HIV?

R: Nothing.

I: Mmm. You said when you heard from the radio it’s when you asked Patricia?, How about your parent, haven’t you ever asked your mother?

R: Mmm, I haven’t.

I: How about anyone else whom you ever discussed about HIV apart from Patricia?

R: I haven’t discussed it with anyone else.

I: Mmm. But don’t you want to know more about HIV?

R: I want but I have never asked anyone. I only asked one.

I: Mmm. What do you specifically want to hear?

R: Maybe how to help HIV patients.

I: Mmm.

R: I want to ask

I: Ask about what? What specifically? As we said that what we want to know what is lacking in girls from what we are discussing, right?

R: Mmm.

I: So what do you want to know?

R: What HIV helps when someone gets sick from it.

I: Mmm.

R: So that’s what I wanted to ask.

I: Okay. Another thing, I wanted to know, have you ever heard about getting pregnant? That a girl is pregnant and expecting? And also how people prevent it?

R: I only heard about pregnancy.

I: Mmm. Where did you hear it from?

R: I heard people talking that a certain 12 year girl got pregnant and bore a child. That’s all what I heard.

I: Mmm. But you don’t discuss this with your friends when you are playing?

R: Other people talk only that i don’t hear because I am far from those people.

I: Mmm. But your fellow girls discuss. How about at school, don’t you learn about these? About HIV and getting pregnant.

R: People learn.

I: You didn’t learn in your class?

R: I haven’t learn but maybe others learn.

I: But you haven’t learn or heard?

R: Yes.

I: When a girl is growing things change on the body,

R: Mmm.

I: For example you didn’t maybe have breast but now you are developing breasts, some start to develop hair in the armpits, it happens right?

R: Mmm.

I: And others start menstruating and you have said you haven’t heard about it, right? That a girl has grown up and started menstruating, right?

R: Mmm.

I: So I wanted to ask about other thing, that when you noticed that you were developing breasts, what did you do?

R: They clean

I: Mmm?

R: They clean

I: I mean you yourself, what did you do when you noticed that it started?

R: Nothing.

I: Didn’t you ask anyone? Or talk to anyone to see what was happening or your friends?

R: Yes.

I: Mmm?

R: Yes, I didn’t ask.

I: You haven’t asked, discussed with your friends?

R: Yes

I: Mmm. Alright. But do you have questions that you want to ask why these are happening? Do you have interest to know more about these? That why you are developing breasts and hair somewhere, don’t you have questions so that you know what is happening?

R: No, I don’t have. I just stay.

I: You just see that they are happening?

R: Mmm.

I: Mmm. You don’t even discuss with your friends?

R: Yes. Some of my friends are gossipers.

I: Mmm. So you fear to share with/talk to them?

R: Mmm.

I: Because you fear that others will gossip you?

R: Mmm.

I: Mmm. But don’t want to talk with an old person, to discuss with them?

R: No, I don’t want.

I: Mmm. Alright. Have you ever heard some people talking about child bearing or someone getting pregnant? That they go for postnatal visits and go to deliver at the hospital?

R: Mmm.

I: Have you ever heard people discussing?

R: Mmm.

I: What did they say?

R: They said someone gets pregnant, go for prenatal visits and deliver.

I: Mmm.

R: That they deliver a baby girl or boy.

I: Mmm. So about this issue, do you have interest to know more about it? Like how it happens or have you ever learnt somewhere how this issue happens?

R: No, I haven’t heard.

I: Alright. Have you ever heard about people being in a relationship, a boy and a girl?

R: Mmm.

I: Have you ever heard?

R: Mmm.

I: Does this happen in this village?

R: A boy and a girl?

I: Mmm.

R: It seems so.

I: Mmm?

R: It seems it happens here.

I: Does it happen?

R: Mmm.

I: Mmm. What makes boy and girls to be in such kind of relationships?

R: Some say one gets impregnated and deliver a baby. Some admire a baby girl some a boy.

I: Mmm.

R: Some want to get married.

I: Some want to get married?

R: Mmm.

I: Did parents or anyone ever counselled you about relationships between girls and boys?

R: As for me, they told me not to go somewhere at night and I accepted it.

I: Who told you?

R: My mother and father two.

I: What made them tell you that? What happened for them to tell you that?

R: Nothing.

I: Mmm.

R: Nothing.

I: Nothing. They just started telling you one day?

R: Mmm.

I: Where did they tell you that?

R: It was here, it was my Uncle from MA.

I: Mmm.

R: That time I came around 6 or 7pm, at night so they asked me where I was and I told them that I was at VX’s so they told me not to come at night so I accepted.

I: So did they mention about boys, that there are relationships between boys and girls?

R: No.

I: Alright. Would you have interest to know some of the things that happens in a relationship between girls and boys? Some things that you want to know, maybe questions or anything that gives you interest to know it?

R: I want to know.

I: What do you want to know?

R: I want to know how to be in a relationship and I also getting sick from HIV.

I: Mmm. What do you want to know about the relationship?

R: Some people want to know how to be in a relationship. Some people want to know.

I: Mmm. To know how to be in a relationship and maybe what is supposed to happen in the relationship?

R: Some impregnates each other

I: Mmm.

R: And give each other babies

I: Mmm. Do you think these are good or not?

R: No

I: About impregnating each other?

R: No.

I: It’s not good? Why is it not good?

R: Because some get pregnant while they are 9 some 10 years old.

I: How about if someone who is 12 years old like you can get pregnant?

R: It is not good to young people but old people.

I: When we say old people, what age are we starting from?

R: 14

I: 14? Is it okay for someone to get pregnant from 14 years?

R: Mmm. Some say that when we want to deliver a baby, we have to start menstruating/period.

I: To start menstruating?

R: Mmm.

I: And you said you haven’t started menstruating?

R: Mmm.

I: Mmm. So someone has to start menstruation if they want to deliver a baby? So to you, is it okay for everyone who has started menstruation to deliver a baby?

R: They say that parents are happy.

I: Parents are happy when a girl starts delivering…….?

R: when she starts menstruating.

I: When she starts menstruating parents are happy that she will bear a child?

R: Mmm.

I: Alright. Is there anything that you would want to tell me again?

R: No.

I: Anything about your life that you would want to tell me? Are there challenges that you face that you would want assistance, what are the challenges you face?

R: [silent]

I: when you are here at the village as a girl, what troubles you in your heart that if perhaps things would happen in a certain way or change for you to be happy?

R: Sometimes I go to school on an empty stomach because there is no food, sometimes I don’t have notebooks and pen no shoes and I sometimes go with bare foot.

I: Mmm. So you want things like these to change in your life?

R: Yes. I don’t have bags.

I: You also don’t have school bags to carry notebooks for school?

R: Mmm.

I: Mmm. Alright.

R: I don’t have uniform and I have never worn it ever since I started.

I: You have never worn uniform?

R: Mmm.

I: Mmm. Alright. So what is your vision for school/education? Where do you think you will reach with your education and what do you want to become in future? Why do you go to school?

R: Because I want to be a nurse.

I: You want to be nurse?

R: Mmm.

I: Why do you want to be a nurse?

R: To help the sick.

I: To help the sick?

R: Mmm.

I: So what do you think you should do to achieve this vision of you becoming a nurse?

R: I should start school.

I: To start school, don’t you go to school?

R: I go.

I: You do?

R: Mmm.

I: Mmm. Alright. Thank you so much. What did you say your name was, MX?

R: Mmm.

I: Thank you so much for your participation in answering these questions.

END
