# Supplementary material for: Social support and very young adolescent girl’s knowledge on sexual relationships: A comparative qualitative study of Girl Only Clubs’ participants and non-participants in rural Malawi
Source: PLOS Glob Public Health. 2023 Jan 12;3(1):e0001339. doi: 10.1371/journal.pgph.0001339 (PMC10022037; doi:10.1371/journal.pgph.0001339)
Supplement: S1 Data — (ZIP) [file pgph.0001339.s003.zip › Anonymized/IDI_ND_XX_3.docx]

**Location**: XX-MI Village

**Date**: DD/MM/2020

**Time**: 2:20 pm

I: Could you tell me what your name was again?

R: JJ

I: JJ. How old are you?

R: 13

I: You are thirteen years old right? Okay. Do you go to school?

R: I stopped going to school

I: You stopped going to school, you used to go but now you no longer do?

R: Yes

I: When did you stop going to school?

R: Before the Covid pandemic

I: Why did you stop going to school?

R: Lack of household help

I: Like what?

R: Books, school bags and we also didn’t find food at home when we come back from school. I would find my mom sad. My younger sibling and I would not find food at home when we come back from school in the afternoon and we would only eat at night. We also did not eat in the morning when going to school. The kids at school also used to tease me and then I stopped going to school.

I: The kids at school used to tease you?

R: Yes

I: What did they say

R: They said I am too old

I: In what class are you?

R: Standard 3

I: So the kids at school said you were too old for standard 3?

R: Yes

I: So you stopped going to school?

R: Yes

I: You stopped going to school because of shortage of food, so when you stopped going to school was there more food at home?

R: No

I: So how is it different from going to school?

R: I decided to go back

I: You decided to go back to school?

R: Yes. I admired my friends who still went to school

I: Why is it that you are behind in studies and are learning with younger children.

R: I was not smart enough and couldn’t understand what was being taught.

I: You did not understand what was being taught?

R: Yes

I: Were you repeating the classes?

R: Yes, I was repeating the classes

I: You were repeating the classes?

R: Yes

I: When you go back to school, will you continue in standard 3 or standard 4?

R: 4

I: You are going in standard four?

R: Mmm

I: Okay. Who do you live with at home?

R: My mom

I: Who else

R: My sister

I: Your sister?

R: Yes

I: Including you, how many children do you live with at home?

R: 3

I: The first is your elder sister?

R: Yes

I: Then you and then who?

R: My other sister

I: You are the last born? What about that one

R: That is the last born

I: The one I was speaking with?

R: Yes, SW

I: So how many children are in your household?

R: 4

I: All girls?

R: Yes

I: Where is your father?

R: He lives at FK

I: Why does he not live with your mother?

R: I don’t know

I: Did they separate?

R: It did. They don’t talk to each other

I: A long time ago?

R: Yes

I: Did he remarry?

R: No

I: Does he come to visit you

R: Mmm

I: What does your mom do to provide for the family?

R: She does nothing. Sometimes her friends share us flour and buy relish and then we eat. We eat in the evening and don’t eat in the afternoon.

I: Does she farm?

R: Yes, she does but the food which we get from the farm is too little.

I: Okay.

R: I used to go to go to school because the Youth organization forced me. (Not clear)

I: To go to school?

R: Yes, then I started to go to school.

I: Now you started to go to school?

R: Yes. (cannot hear)

I: What happens at home which you don’t like?

R: Which I don’t like?

I: Yes. Which things don’t you like or make you worried at home?

R: Not finding food at home

I: Could you tell me another?

R: Not having soap at home and wearing dirty clothes and also sometimes having flour with no relish.

I: Do you have any friends which you chat with?

R: Yes

I: Who do you chat with?

R: Sf, V, Sp, Rh, Ts, Mh, Sh and Ns

I: Of all your friends, who do you love more?

R: Sf and Ts

I: Why do you love them more?

R: When we don’t have food at home, I go to eat at their houses.

I: What about the rest?

R: Sometimes I don’t get the chance to and we also fight sometimes and I then go to Sf’s house to eat nsima. I also chat with Sf at school.

I: Who do you talk to when you are sad?

R: When one of my friends have made me sad?

I: When anything sad happens, who do you go to talk?

R: My parents

I: Has there been anything that irked you which you talked to your parents about?

R: I always used to fail at school which used to make sad and my mom encouraged me to continue going to school. I also didn’t have a uniform.

I: That is what irked you at school?

R: Yes, and carrying my things in a plastic bag because I didn’t have a school bag.

I: You told your mom about this

R: Yes

I: Okay. What did she say about this?

R: She told me that when she finds the money she would buy it for me and to thank God.

I: I wanted to know if have started having your period?

R: No

I: You haven’t started having periods?

R: No

I: Have you ever talked with anyone else about this topic?

R: No

I: Or any of your friends telling you that when girls get older they start having periods?

R: No they haven’t

I: Nobody has, including your friends?

R: No

I: Have you ever heard about HIV?

R: Yes

I: Where did you hear about HIV?

R: People talk about it

I: What?

R: People talk about preventing the spread of HIV

I: Could you tell me about the first time you heard about HIV?

R: On the ground at school. Our madam also told us that unprotected sex is bad because you can contract HIV

I: Where else?

R: There was a play on the ground where a child was sleeping with another person who was older contracted HIV.

I: Where else have you heard of HIV?

R: On the radio

I: What about with your friends?

R: When I bring it up they avoid the topic. For example, when I tell them that sex is bad they change the subject.

I: Why do you think they avoid the topic?

R: They support having unprotected sex and contracting HIV

I: Would you say that your friends have sexual relationships?

R: Maybe they do

I: Have you ever heard or discussed with anyone on how to get pregnant or avoid getting pregnant?

R: About avoiding pregnancy

I: Who did you discuss with?

R: My friend. She told me that `` Jq, don’t have unprotected sex with other people because you could get pregnant and your mother doesn’t have the resources to take care you if you do get pregnant. `` Someone else also told me not to have unprotected sex with people older than me. I then realized that If I get pregnant my mother wouldn’t have the resources to take care of me.( Can’t hear the last part)

I: Where were you talking about this?

R: School

I: In your class?

R: Yes

I: Okay. Have you ever discussed with anyone including your friends or parents about relationships between boys and girls for example it’s advantages and disadvantages.

R: Yes

I: You have?

R: Yes

I: With who?

R: My friend

I: How did it all happen?

R: She told me`` Jq, I have gotten a boyfriend,`` and I asked her who it was. She told me it was Yk and then told her that he was too old for her and that he could get her pregnant. I also told her not to sleep with him again and to end the relationship. We stopped being friends since then because I advised her and I know chat with the others.

I: Is there anything else you would want to tell me which I haven’t asked about?

R: (Long pause)

I: Okay. I would now like you to tell me your daily routine at home.

R: Daily?

I: When you wake up, what do you do up until evening.

R: When I wake up I take water to the bathroom and clean myself up. ( I cant hear what she is saying for two more lines)

I: When you want to go for a walk, where do you go?

R: That house over there

I: Where else?

R: Mn

I: Where is that?

R: At chief Mn’s house. Where you were. And also the houses near the graveyard.

I: What troubles do young girls face when they are going to play or chat with others?

R: There are a lot of people who like fighting and arguing with others. Most of us don’t want to do that though and when someone is causing trouble we just leave them alone and find another person to chat with. For example, when you tell them a story, they may tell another to start an argument which could turn into a fight and get us into jail. We just avoid them.

I: What other problems do girls face?

R: Problems?

I: Yes, when they go to play.

R: (Long pause) I don’t know

I: Don’t boys provoke you?

R: They do. They stop on the road and ask us to be in relationships with them. Even if you say no, they continue asking but they are some girls who say yes or want to say yes. Sometimes even if they don’t want to.

I: Has something like this ever happened to you?

R: Yes

I: Could you tell me what happened?

R: I was walking on the road and I met some boys coming from playing football. They called `` Hey you , stop!`` But I did not stop and one pursued me so I ran and continued on my way. I also met the same boy on another day and asked me to be in a relationship with him but I said no. He then told his family about it and later a girl started cursing me in public.

I: Why did she curse you?

R: She said I turned down her brother.

I: Has anything else similar to that happened to you?

R: With that?

I: Someone else asking you to be in a relationship with them

R: No. I just chat with the other boys but it was only him who asked me to be in a relationship with me. Sometimes ` the akabanza boys ` may ask me to stop but I heard it from school that unprotected sex is bad.

I: These where the questions I had for you today. I just remembered one question, you said you stopped school but you may go back.

R: I used to go then I stopped

I: When you stopped school how did you think your future would turn out.

R: Finishing school or stopping going to school?

I: People grow old right?

R: Yes

I: What do you see yourself doing when you are twenty or twenty-five years old?

R: 25?

I: What do you think you will be doing?

R: Encouraging girls to go to school

I: Who do you think you will be or what do you think you will be doing?

R: (Long pause)

I: Everyone gets old right? You want forever be thirteen and what do you see yourself doing when you are my age or your mothers age?

R: Married

I: How old do you think you will be when you get married?

R: Twenty-five

I: With children?

R: No

I: When you get married what will you be doing or will you just stay at home?

R: I will be a nurse

I: You will be a nurse?

R: Helping sick people

I: Okay. Thank you I just wanted to add these questions.

END
