# Supplementary material for: Social support and very young adolescent girl’s knowledge on sexual relationships: A comparative qualitative study of Girl Only Clubs’ participants and non-participants in rural Malawi
Source: PLOS Glob Public Health. 2023 Jan 12;3(1):e0001339. doi: 10.1371/journal.pgph.0001339 (PMC10022037; doi:10.1371/journal.pgph.0001339)
Supplement: S1 Data — (ZIP) [file pgph.0001339.s003.zip › Anonymized/IDI_ND_XX_4.docx]

**Location**: XX-MI Village

**Date**: DD/MM/2020

**Time**: 10:15 am

I: First of all tell me your name?

R: Mmm

I: What is your name?

R: RI.

I: RI?

R: Yes.

I: Alright, in which class are you?

R: 6

I: where do you go to school?

R: XX L.E.A

I: You learn at XX L.E.A?

R: Yes

I: Alright, what is it that you like at your school when you are learning?

R: English,

I: Mmmh.

R: It is because I would want to know how to read English very much, I like it when others are able to and it is embarrassing for you to fail, when you are failing and your friends should be able to.

I: Mmm

R: You just look at your friend being able to speak and you dislike it that your friend is able and you cannot.

I: Mmmm

R: So, I would like to know English well.

I: What else? Why do you go to school?

R: Why I go to school?

I: Mmm

R: I would like to have good life.

I: Mmm

R: I would like to help my parents.

I: Mmm

R: helping this household and be independent.

I: Mmm

R: Yes

I: How is your education going on? Do you pass in class very well or how do you do?

R: I pass very well.

I: Mmmm

R: But other things are still tough

I: Which subject do you like?

R: the subject that I like?

I: Yes

R: Social.

I: Last term, which position did you get?

R: 21.

I: 21?

R: Yes

I: You don’t admire to be number 1?

R: I admire very much

I: Mmmm, so what do you do to be number 1?

R: I try my best to study in order not to forget and also to be on position 1 where my friends are.

I: Mmm

R: Yes

I: What do you want to be when you finish school?

R: I want to be a nurse

I: Why do you want to be a nurse?

R: I want to help sick people including my family when it gets sick at home.

I: mmm

R: Yes

I: Do you have a role model or someone you aspire to be like. The person could either be from this village or not.

R: Yes, there is.

I: Who?

R: Miracle’s mother.

I: Does miracle’s mother live in this village?

R: Yes, she lives in this village.

I: What does she do and why do you admire her?

R: She is a nurse. I like the way she works and her uniform also looks good on her.

I: Mmm

R: Yes

I: She is a nurse who stays in this village?

R: Mmm

I: Where does she work?

R: At LE

I: At LE?

R: Mmm

I: Does she commute to and from?

R: Mmm

I: Could you tell me who you live with at home?

R: My mom

I: You stay with your mom?

R: Yes, my father lives in MC.

I: Mmm, what does your father do in MC?

R: He used to be a minibus driver, but right now he just stays there.

I: Mmm, does he ever come here?

R: Yes, he does.

I: How often does he come?

R: Not very often.

I: Mmm, why didn’t you want to go to live with him in MC?

R: We wanted to but he said the rent for the houses were expensive.

I: Mmm, is this your house?

R: Yes

I: How many kids are there?

R: 6

I: Who is the first born

R: The first born is a boy

I: What about you

R: Am the second born

I: Does the boy go to school?

R: Yes

I: Where is he learning?

R: He learns right here in XX

I: In which class?

R: 8

I: Who are you closest to at home?

R: My mom

I: You are very close to your mother?

R: Yes

I: Alright, to what extent do you feel comfortable with your mother? Do you share stories, is she a person that you can tell her anything, what is your relationship with her?

R: I can ask her anything, and why?

I: Mmm

R: The time I started my period, right?

I: Mmm

R: I told her that I have started this, and she told me what to do.

I: It was your mother who told you?

R: Yes

I: Alright, before you started your period, were you already aware of it, or heard about it from somewhere, or it just happened?

R: used to hear the name from friends

I: Mmm

R: But by that time, I didn’t know how one would take care of herself or what she can do.

I: where did you hear it from?

R: I heard when I was staying in NU.

I: Mmm

R: I have an older relative and was in a higher class than me and I could just see her reading I was just looking at the pictures, so when it happened, people told me its name ‘period’ and that when I realized.

I: Alright, I would like you tell me what happened the day, when you told your mother, what happened? And when this happened how did you feel inside you, were you afraid, worried or what happened?

R: On that day I was very worried because I didn’t know it.

I: What happened?

R: It never happened all my life.

I: Mmm

R: and I was afraid that maybe when am amongst my friends I can mess up myself.

I: Mmm

R: I worried how I would stay on the group with boys, and I was worried how I will be sitting down.

I: On the day it happened, what happened for you to gather courage and tell your mother? Tell me what happened?

R: She noticed and asked me if I have started period, and I told her that I have noticed red things of which I don’t know and she told me that it is period and she sat me down and started telling me how to take care of myself.

I: Mm

R: Yes

I: Did she call other people or elders to tell you as per tradition, did that happen?

R: Yes

I: What other things happened?

R: They just told me how to put on a pad.

I: Mmm

R: Yes, bathing and taking care of myself so that I don’t produce smell.

I: What about girl/boy relationships, what did they tell you?

R: They told me that when am doing my period, I should not stay much with boys since boys can embarrass me.

I: Alright, who are your friends you chat with?

R: I should mention them?

I: Yes, your best friend, how many best friends do you have?

R: Only 3

I: What are their names?

R: NX, UX and LX.

I: NX the one from the village chief?

R: Yes

I: Alright, why do you get along with them?

R: We share a lot of things.

I: What are the things you discuss?

R: We talk about period, we also talk about proceeding with our education and not mind about boys, listening to parents.

I: Mmm.

R: Yes

I: Alright, so, where do you go, as friends where do you go to chat?

R: We just go ro0und the village and after that we go and sit at one house and chat and when it’s dark, we go to our homes.

I: Apart from school where else do you go and chat and also go?

R: To watch balls somewhere, weddings, birthday parties, we also go there.

I: As you said, when you are going out like friends houses, football/netball, market, what are the problems that you meet or you may might, things that disappointed you?

R: Some say lies, some do things to make you feel bad about yourself because you don’t dress to impress.

I: Alright, in other areas, girls meet men who touches their breasts, proposing to them, here, does this happen?

R: Yes

I: Or being raped, or a boy touching your breast, does this happen?

R: Yes, it does

I: Have you ever had a similar situation?

R: It has never happened to me but my friends, I have heard them saying someone touched my bum or breast.

I: If such things happen, where do girls go to report, in this community?

R: To his mother, parents or village head.

I: I wanted to ask about HIV/AIDS, where have heard such issues?

R: Just heard from school when learning

I: they teach you?

R: Yes

I: In which subject did you learn this?

R: English and Science

I: What do they say, what do they say that HIV and AIDS is?

R: HIV is a virus that causes AIDS

I: What else did you learn about AIDS?

R: Protect yourself when having sex to avoid AIDS.

I: Apart from school where did you get such information?

R: it is only at school

I: They do talk, right?

R: Yes

I: Have you ever heard of stories about girls getting pregnant, or avoiding pregnancy, or anything about pregnancy?

R: At school there was a certain organization that was teaching us about that.

I: they taught you?

R: Yes

I: What were they saying?

R: How a girl or any person can protect themselves when one is about to ambush them, and how one can avoid pregnancy on her own.

I: Alright, sometimes in the community, girls can have a relationship with a boy/

R: Yes

I: Does it happen here, especially to girls of your age?

R: What happens

I: Why do girls do that?

R: Some do that since their parents assistance/help is not enough that is why they do that.

I: So you have a boyfriend?

R: No

I: Have you ever had a boyfriend?

R: No

I: you’ve never had one?

R: Yes

I: Alright, what are your views on girl/boy relationship, when you hear about them?

R: I don’t admire it at all even if I don’t have support of soap at home, I don’t admire.

I: why?

R: Because I have never had idea of doing that now.

I: Alright, you told me that you are staying with your mother and siblings?

R: Yes

I: What are the things that makes you feel unhappy with your parents or at home?

R: Parents sometimes shout at me with no reason, things that you didn’t do in a wrong, they just shout.

I: Alright, what else?

R: on the issue of doing house chores, you do them according to your age, right?

I: Yes

R: Sometimes, there are more work as compared to what you can accomplish at your age.

I: What kind of work?

R: Farming

I: we are at the end of our questions. Is there anything that I didn’t ask and you would love to tell me?

R: No

I: No?

R: Yes

I: Alright, so thank you very much for answering the question.

end
