# Supplementary material for: Social support and very young adolescent girl’s knowledge on sexual relationships: A comparative qualitative study of Girl Only Clubs’ participants and non-participants in rural Malawi
Source: PLOS Glob Public Health. 2023 Jan 12;3(1):e0001339. doi: 10.1371/journal.pgph.0001339 (PMC10022037; doi:10.1371/journal.pgph.0001339)
Supplement: S1 Data — (ZIP) [file pgph.0001339.s003.zip › Anonymized/IDI_ND_XX_5.docx]

**Location**: XX-MI Village

**Date**: DD/MM/2020

**Time**: 11:30 am

I: We are starting, what’s your name?

R: UA.

I: UA, how old are you?

R: 14.

I: Alright, which school do you go?

R: XX L.E.A school.

I: In which class are you?

R: 6.

I: I wanted to know more about your school, XX L.E.A, what you like about it and what motivates you to go to school.

R: on education?

I: Yes

R: I like Maths, Chichewa, English and other subject

I: You like all of them?

R: I don’t like all of them

I: What are those that you don’t like?

R: Social.

I: Why don’t you like it?

R: Because I don’t understand it properly

I: Ooh, you did not understand it? But you understand Math properly?

R: Yes

I: What about your grades, do you do well or poorly in class?

R: I often get ``ok`` scores

I: Is it a little hard?

R: Yes

I: What was your position in class last term?

R: I didn’t pass, I failed

I: You failed

R: Yes

I: What class did you say you were in?

R: 6.

I: Standard 6, so what are you doing to pull your grades up?

R: Mmm?

I: What measures are you taking to start passing and be on position 1?

R: Listening

I: Yes

R: Listening to what the teacher is teaching.

I: What do you do when you are home?

R: I read

I: You read?

R: Yes

I: When you think of your future, who do you want to be?

R: I want to be a bank teller

I: Mmm bank teller, why do you want to be a bank teller, the money you count won’t be yours but the company’s money

R: Yes I know that, but it is still a job

I: It’s the job you aspire to have?

R: Yes

I: For you to be a bank teller?

R: Eya. Yes

I: Is there anyone who inspired you to want to become a bank teller?

R: No

I: You just thought of it?

R: Yes, I just thought that when I finish my school, I want to be a bank teller

I: A bank teller?

R: Yes

I: Alright, is there anyone in this village either educated or not whose lifestyle you admire and which you want to have in the future?

R: ZS.

I: Yes

R: She is a nurse

I: Where does she work?

R: She doesn’t stay here but right now works at XX.

I: That’s the one you admire?

R: Yes

I: You admire to be like her?

R: Yes

I: Alright, whom do you stay with?

R: My grandmother

I: Just the two of you with your grandmother?

R: With the other girl who was here

I: Oky?

R: with another one who went to South Africa, she is my aunt

I: The girl?

R: She is also my aunt

I: She is also your aunt, so you are 3?

R: 4, with a baby

I: Just 4 of you?

R: Yes

I: Where are your parents?

R: My mother stays at KX and my father died.

I: Your father died, how many are you in your family?

R: 6.

I: Who is the first?

R: KXX, is in SXX.

I: The others?

R: The 2^nd^ one are twins, the other twin is here and the other one is in SXX, the 4rd is in SXX and am the 4^th^ one.

I: You are the last born?

R: No, the last born is in Grade 3.

I: Stays with your mother?

R: Yes

I: Why did you chose to come and stay with your granny and you don’t stay with your mother?

R: work

I: Work? What do you mean?

R: I want to help with household work

I: What about your granny? Is she a mother to your mommy?

R: My father

I: Who mothered your father, alright, why do you like staying with your granny?

R: Because she provides everything for my school.

I: She bought notebooks and what?

R: Uniform.

I: Your granny, she is loving granny?

R: Yes

I: What is it that you can say this should change, I don’t like it?

R: Nothing

I: Nothing?

R: Yes

I: Everything is okay?

R: Yes

I: Alright, I want to know if you have friends?

R: Yes, many.

I: You have more friends.

R: Yes

I: Where do the stay, who is your best friend?

R: Leah.

I: Why is Lea your best friend?

R: Because when I tell her my problems she tells me what is bad and good

I: Can you tell me one problem that you told Leah and she told you this is bad or good?

R: We were dancing and she told me that we should stop it because it makes people start promiscuous behavior.

I: What kind of dance?

R: You learn how to dance and they pick you for recordings and you may go to Lilongwe?

I: like practicing?

R: Mmm, and in Lilongwe they will call up that they want youth from XX, and will send a car to pick them up and go and record the songs, and after dancing they are paid money.

I: Okay?

R: I joined that group.

I: Mmm.

R: And she came and told me to stop because it makes people start promiscuous behavior, and I stopped.

I: Which singer were you dancing for?

R: Mmm, it was my first time, I have never done it before.

I: You were just dancing?

R: We were just training.

I: Training?

R: Yes

I: If someone does better?

R: on the 15^th^ October people will go to Mandevu to dance.

I: They already did training?

R: Yes

I: So, people come here and train them and thereafter they should go and dance?

R: Yes

I: And they should get paid?

R: And they are paid.

I: So, people here like that?

R: Yes, they do like it, but not everyone.

I: Mmm maybe because of money?

R: Yes

I: Alright, what else did she advise you?

R: Another one?

I: Yes

R: Some days I was not going to school, and she used to ask why, and I told her that I don’t have note books because my grandmother doesn’t have money, she gave me money and I went to buy.

I: Alright

R: She also bought me pads

I: Pads?

R: Yes

I: Oky alright, Leah goes to school?

R: Yes

I: In which class is she?

R: form 2.

I: She is in form 2?

R: Mmm

I: Alright, I would like to know where do you go to chat when you finish working at home?

R: At LXX place

I: Where else?

R: LXX place

I: Where else?

R: NXX’s

I: Oky at NXX’s, oky oky.

R: Sometimes from there we would go to watch ball

I: Alright, what are the problems that you meet every on your way to your friends place or when you are chatting?

R: No

I: There are no problems that you meet?

R: No

I: In other places girls are raped or boys touch their breast?

R: Such thing mostly happens at school

I: That happens at school?

R: Yes

I: Alright, but not here?

R: Aaaa, I have never seen that in the village.

I: Has it ever happened to you?

R: Me?

I: yes

R: No

I: Boys frightening you and proposing to you?

R: Yes, and many

I: proposing to you?

R: (laughing)

I: What do they say, they want you to be their boyfriend?

R: They just stop you on the way or send someone to tell you, to tell you that he loves you and you choose to say yes or no.

I: Mmh.

R: If you say yes then you tell the same person who brought the message to tell him.

I: What happens with girl/boy relationships here? What is it that makes girls have such relationships?

R: Some give each other money, some buys clothes for each other and others tell each other about marriage.

I: Girls of your age?

R: Yes, we have them in this community.

I: When you are with your friends, when you are chatting, do you discuss about relationships? Its advantages and disadvantages like girls?

R: For us we discuss about school and about relationships we say they are bad things because of pregnancies.

I: Did your granny, aunties or mother, did they ever advise you9r about girl/boy relationships?

R: My aunt, granny and mother all once advised me that relationships are not good.

I: Alright, I would like to know about HIVAIDS or sexual transmitted infections, do you discuss such issues with your friends when you are chatting?

R: When chatting?

I: Yes

R: No, we don’t.

I: What about school?

R: At school, in class we learn about HIV/AIDS.

I: Alright, when you have question or desire to know more about it, whether HIV or STIs, like an adolescent girl, do you have desire to know more about these? And how do you want to discuss it with, at the village?

R: In this village they are scarce.

I: No one for you to ask them questions?

R: Yes

I: Is there any place in this community where girls go to learn such information?

R: No

I: There’snt?

R: Yes

I: Do you need such things that you have a club and someone should come and you should ask questions?

R: Yes and also CAMFED

I: Do you have CAMFED?

R: Yes

I: But you don’t have in the community?

R: Yes

I: Okay, so here in the community you lack this too?

R: Yes

I: So, you said it is only at school where you learn that?

R: Yes

I: What about issues about pregnancy, how to avoid it, where do you get such advice?

R: From parents and school too.

I: At school you are taught?

R: Yes

I: Alright, do you discuss such issues in groups where you girls are chatting?

R: We discuss in groups

I: Do you discuss it?

R: Yes

I: Things like how you can avoid pregnancy?

R: That pregnancy is not good.

I: Let us go back, when you had your first period, before it happened, at that time, did you ever heard about it?

R: About having sex?

I: Period or coming of age?

R: Before I started?

I: Yes?

R: Yes

I: Where did you hear it from?

R: At school, when we are learning, they talk, some girls were saying that they are going to get pad, at that time we were not having it. Someone came who were distributing pads and note books for those who started having period.

I: Did you admire it?

R: I admired it

I: So, what happened on the first day when you started your period?

R: What happened?

I: Yes?

R: I just saw it in the morning and I told my grandmother.

I: you were staying with your granny at this time?

R: Iiii I started staying with her long time, she was the one who weaned me from being breatfed.

I: So, you told her?

R: Yes, and she told me how to do it.

I: Apart from your granny, was there anyone who advised you?

R: Yes, my friends, I told them.

I: Your friends? Other older people?

R: My mother

I: She also told you?

R: Yes

I: In your community, does girls go to initiation when they start having period?

R: When you start that?

I: Yes

R: Yes

I: You go to initiation?

R: Yes, people go but I didn’t go

I: Why didn’t you go?

R: Mmm I don’t know what my mother thought of.

I: They didn’t send you?

R: Yes

I: We have asked a lot of questions, do you have a boyfriend?

R: No

I: Do you have or you don’t or you are lying to me?

R: I don’t have

I: Okay, why don’t you have?

R: I feel like such relationships are not good.

I: , now we are at the end of the interview, maybe you have something you would like to tell me?

R: Me, on my life needs?

I: Mmmh, or things that you don’t like, things that worries you as a girl growing up here in MI?

R: When I fight with my friends I do become sad and if someone steals my notebooks.

I: That’s all that makes you sad?

R: Yes

I: If there maybe a new programme for girls, what kind of program would you like?

R: Any other program we can like it.

I: Ooh.

R: If they tell us to go somewhere we can go, like this study, they just told us that they have listed you but we don’t know what they are bringing, and we’ve come.

I: thank you Upile because of answering the questions.

R: thank you.

END
