# Supplementary material for: Social support and very young adolescent girl’s knowledge on sexual relationships: A comparative qualitative study of Girl Only Clubs’ participants and non-participants in rural Malawi
Source: PLOS Glob Public Health. 2023 Jan 12;3(1):e0001339. doi: 10.1371/journal.pgph.0001339 (PMC10022037; doi:10.1371/journal.pgph.0001339)
Supplement: S1 Data — (ZIP) [file pgph.0001339.s003.zip › Anonymized/IDI_ND_XX_6.docx]

**Location**: XX-MI Village

**Date**: DD/MM/2020

**Time**: 2:00 pm

I: What’s your name?

R: SJ

I: How old are you?

R: 14

I: Do you go to school?

R: No, I don’t go

I: When did you left school?

R: I left last year I have a child

I: Okay. So you gave birth to that child when you were 13, right?

R: No 12

I: so, how old are you now?

R: 14

I: So, you give birth in 20XX?

R: Mmm

I: I would like to know about your household. With whom do you stay with?

R: My grandmother

I: That’s your grandmother’s place?

R: Yes

I: Okay, do you have your mother and father?

R: Yes

I: Where do they stay?

R: In LXL. I wasn’t staying here, I was staying in LXL, I have just come here soon.

I: The time you were giving birth you were in LXL?

R: Yes, I was in LXL.

I: Aaaa okay. So you were staying with your mother and father?

R: Yes

I: How many children are you in your family?

R: 3

I: Who is the first born?

R: The one who is there, wearing a green clothe.

I: Second born?

R: Myself

I: The 3^rd^?

R: There is a girl

I: Where is that one?

R: She is in ZXA at my Aunt’s place (amai ang’ono)

I: I would like to know, why are you not staying with your parents? The 2 of you are here with your grandmother and the other one is in ZXA with your aunt? That means your mother doesn’t have any child, right?

R: Yes

I: Why?

R: My mother would like to move out, she had acquired passport and she is working in LXL.

I: Where does she want to go?

R: To Johannesburg

I: What does your father do?

R: My mother and father divorced when I was young.

I: And they don’t stay together? They are in LXL both of them but they don’t stay together?

R: My father is in Johannesburg, he married another wife.

I: Alright. Do you have friends here?

R: Yes

I: How many friends do you have?

R: the one we agree on things is one.

I: What is her name?

R: CX

I: Why do you click with her?

R: You know girl issues, if you talk of something the next thing you hear a different story and because of that I decided to have one friend.

I: Where does CX stay?

R: At LI

I: Does she go to school?

R: No, she is pregnant.

I: Is she 14 also?

R: No, she is older than me, she was born in 2001.

I: Alright. So, what kind of issues do you discus with her?

R: When we are together we do not talk about other people’s issues, we just talk about our issues, like how things are going.

I: You’ve said, you don’t go to school and she also doesn’t go to school, what are your plans?

R: In my plans, I would like to go back to school.

I: So, years has passed, you said you had your baby in 2018?

R: I was still going to school, the story was like this; I was raped, I was staying with my mother in Area 10. And my mother working at the same place, and there was a driver in the same compound. And this was a Sunday and my mother used to buy all groceries and relish every month end. So, my mother and his wife both went to the market, and at that time I never knew a man. It was Sunday, I washed my uniforms preparing for Monday to go to school. The man forced me in his house and covered my mouth and raped me. He looked as if he was the boss of the compound, he used to lash words on the guards but he wasn’t a boss, and he told me that if I ever mention it to anyone I will see what will happen. And then I stayed for some months, I didn’t know that I was pregnant, and I was still going to school.

I: In which class were you at that moment?

R: I was in Standard 5 that time. My friend were telling me that I was pregnant I used to refuse because I didn’t know. And at the time when we were paying examination fees expecting to start exams the other week, my moms friend called me and told me to close the door. And told me to remove the clothes and of which I did, and she showed me the line on my tummy and told me I was pregnant but I refused. She asked me whose pregnancy it was, I didn’t tell her. She told my mother that she need to get out maybe I can tell my mother. Now my mother asked me if I came pregnant from home and I told her that it was the neighbor. The man was having off duty on Sunday. The women agreed that we go there Sunday, and this time I wasn’t going to school, now my mothers friend said we should go to police. And I put on my uniform and went to police and explained everything and signed. They told us to go to Central hospital in the morning, and at the hospital they told me that I will be doing antenatal clinics until I give birth. We were going between hospital and court, the man was refusing saying why did I not cry for help if he raped me, and I answered that it was because he covered my mouth, he kept on saying that it wasn’t his pregnancy but I kept on saying it was his pregnancy. The man came to a point of selling his possession and got a lawyer and when the lawyer saw me, my age he said the man should serve his sentence. Until I came to give birth, we were still going to court and now he was given 10 sentence and he is serving his sentence.

I: When you they told you that you were pregnant, how were you feeling inside you?

R: I was also surprised because I wasn’t having my period and I was having backaches, and I was just ignoring them because I was young.

I: Were you not afraid?

R: I was becoming afraid as to why I was not having my period. And when my friend told me, I just accepted it that I was pregnant.

I: How was your mother treating you when you were pregnant?

R: I wasn’t working, my mother was the one doing household chores.

I: Where is your child?

R: Is at my grandmother’s house, is playing with friends.

I: What happened when the baby was born?

R: When the baby was born, I had a friend. In LXL, house workers stay at quarters and even when I was pregnant my friend used to visit me. And her mother said you should not lack soap for the child and she told me to be selling mandazi for her, she told me that she will be giving me 10,000 and I did that for 3 weeks. The boss who was staying there was working at Reserve bank and she saw going to sell mandazi. She told me to put down the bucket, and I did that, she asked me to show her the baby of which I did. She asked me if I can go back to school? I didn’t respond, in my heart I was thinking of my friends at school that they will be laughing at me. She said you are young, I want to talk to your mother. And I went to tell my mother, and my friend told me that I need to say yes, she is a good woman, she will support me. My mother went there with her friend. And my mother said yes, she bought everything for the baby as if I was pregnant. She also told me to look for a nanny for the baby so that I can go back to school. She provided milk for the baby and I started school. And it came to a point where my mother received her pension, I was still going to school. At this time, the company where my mother’s boss was working closed and we had to move from Area 10 to MCS. While staying there she told me to find a nanny for the baby who was being paid 25,000 but I wasn’t happy with the nanny I could differentiate with the one who was taking care of the baby at Area 10. Every month she would call me to go and get baby’s support. Every month she was asking me if the nanny was taking care of the baby very well, I used to answer yes, but I wasn’t happy, but these days she comes late to pick up the child, and was always meeting her on the way. And we stay far from each other, she was staying in KE and I was staying in MCS and I felt it was far, to knock off at 2 and go pick up the child and go home to cook and eat. So, most of the times I wasn’t eating, so, I explained to her what she used to do while taking care of the baby. And I suggested that we find a school for the baby and use the nanny pay to cover school fees for the baby. She agreed and told me that go ahead look for school and everything so that come January I should tell her. I think the woman went to ask the nanny about the whole thing, and she stopped supporting me. I feel the nanny said something bad about us, because when I met her she asked me if the lady was still supporting me, and I said no, and she said ‘what did you think’ as you thought we will still get support, and that’s when I knew that the lady was told a lie by the nanny to stop supporting me, and that is when I stopped going to school, from 2017 until now. But I still want to go to school.

I: Alright. And now you started taking care of the baby yourself?

R: Yes, with my mother, when she gets paid she send money for support of us and my sibling.

I: The child is a boy?

R: Yes, it’s a boy

I: Has started going to school?

R: No

I: Alright, so when did you come here?

R: last year, I came here during harvesting time.

I: I would like to know, right now you are not going to school, what do you do, when you wake up in the morning, what is your day like?

R: After waking up, I go to work in the fields, and thereafter I come and wash plates and if there is food, then grandmother would give me money to go and buy food if there is money, that’s all.

I: You said you only have one friend whom you play with, right?

R: Yes

I: Okay, have you ever heard about HIV/AIDS?

R: Yes, I have heard about it, at school at AIDS club, it was concerning girls.

I: At the time you were learning?

R: Yes

I: What did you learn?

R: They were asking us question on how we can protect ourselves from contracting HIV? They were asking us what one would do if wants to have sex with a man, and we were answering that one should use chishango to protect themselves from it.

I: All this you learnt from Edge?

R: Yes

I: Apart from Edge, where else have you heard about HIV?

R: No where

I: With your mother, have you ever discussed about HIV?

R: No

I: What about your friends?

R: With my friend we were telling each other to be careful

I: At the time this man raped you, did you know how one gets pregnant or how one can protect themselves?

R: I didn’t know anything about a man.

I: So, 2017 is long time ago, and the baby now is toddler, and the lady stopped supporting you, so, now have you found a boyfriend since you are just staying home?

R: No

I: It has only been this man only, you’ve never had another relationship?

R: In LXL?

I: Mmm

R: No, in LXL I was just staying

I: Why have you not had any relationship? I just want to know why, is it because of what happened to you?

R: Because of what happened to me and I decided just to stay.

I: Can you explain better, what is it that you think?

R: Am afraid that it can happen to me again

I: That the boyfriend would do what this man did to you?

R: yes

I: And what are your plans now?

R: To go back to school

I: Alright, you gave birth to a child, how did the hospital support you as young girl, when you were going for antenatal, how were they treating you?

R: The antenatal clinic I was attending, I wasn’t going there in the morning, they told me to go there in the afternoon, and were treating me well.

I: They knew what happened to you?

R: Yes, we explained

I: Were you going there alone?

R: I was going with my mother, she was escorting me.

I: And at the time you were giving birth, how did they treat you?

R: At the time of giving birth, mmm, problems were there.

I: Can you explain, I want to hear what happened?

R: The labour started during the night, I couldn’t catch sleep, I was failing to sleep and I told my mother, and when I told her my mother, at this moment she didn’t buy the necessities like paper and basin, she bought nothing, she bought all things when the baby was already born. The baby was born around 5pm.

I: You went there around 4 and 5 the baby was born?

R: No, we went there 4 am and the baby was born 5 pm.

I: So, how did they the nurses welcome you at the hospital?

R: They welcomed me.

I: You have gone through a pregnancy now. Have you ever talked to someone about pregnancy or there are other things that you would like to know?

R: Right now I know

I: Alright. The last question are about boy and girl relationships. Have you ever discussed with CX your friend about boy/girl relationships?

R: She is in a relationship, the boy who impregnated her, he accepted the responsibility but the boy is in form 2 and the girl is in Std 6.

I: And what are their plans?

R: She says they will marry each other.

I: Was she telling you how her relationship was going? And were you not ….to have yours too?

R: No

I: they say birds of the same feathers go together?

R: [laughs] no

I: Or you are just hiding it from me?

R: To say the truth, I had a boyfriend, but I ended the relationship.

I: Why?

R: I loved the guy but he didn’t love me the same, and I wrote a letter to end the relationship and he also had another woman and I thought that everyone should might own business. And now am alone.

I: Was from the same village?

R: No, form KL, from same village with my friend.

I: Was he married?

R: No, he has another girlfriend who stays in DA.

I: So you ended the relationship because of that girl?

R: Yes, I told myself not to stay in that state and I ended it.

I: How long did you stay together?

R: 5 months

I: Were you having sex in the relationship?

R: Yes, I had sex with him once

I: Mmm. Alright, what were you gaining from the relationship?

R: I should say that I wasn’t lacking money to buy soap. When ask him for money, he was giving me, but I just decided to end it.

I: Okay, within this relationship, were you able to discuss about HIV/AIDS?

R: Yes, we were discussing that

I: What else were you discussing in this relationship? Because I wanted to understand why people have relationships, what is the benefit?

R: There are advantages and disadvantages of the relationship. But as for me I was loving him and he wasn’t loving me.

I: What is it that made you think that he didn’t love you?

R: When I visit him at his place he wasn’t giving me attention, when I asked for money, he wasn’t giving me. So I thought staying alone as I was doing before.

I: But at first he was giving you attention?

R: Yes, he was giving me money and when I go to his place, but not now. And I just ended it.

I: Alright, I think those were the question I had, I am happy you are not shy. Is there anything that you would want to tell me that I didn’t ask? Or maybe I should this question; as you are staying here, what is it that makes you feel sad in your life?

R: I have a grandfather who doesn’t speak well to me, when were coming from LXL I found him sick, his hands does not work and he doesn’t walk very well. We are four girls staying with my grandmother, my sister and myself, and the other girls you saw going that way and her elder sister. When he wants to talk on issues of food, he doesn’t talk good things, although he didn’t help farming last season. There is an uncle whom was working but now he is not, he was working in Liwonde. He has his son, and he always says that if we eat here, we eat because of that boy, or otherwise. But my mother every month sends money for relish and 1000 for me to buy soap. And those are the problems we meet here, and another problem is food, it is very scarce here.

I: You only rely on harvest only?

R: Yes, and now the food is finish, we are just buying.

I: And with scarcity of money now.

R: Mmm

I: Okay, is there anything that you would like to tell me?

R: No

I: thank you very much for answering these questions.

END
