# Supplementary material for: Social support and very young adolescent girl’s knowledge on sexual relationships: A comparative qualitative study of Girl Only Clubs’ participants and non-participants in rural Malawi
Source: PLOS Glob Public Health. 2023 Jan 12;3(1):e0001339. doi: 10.1371/journal.pgph.0001339 (PMC10022037; doi:10.1371/journal.pgph.0001339)
Supplement: S1 Data — (ZIP) [file pgph.0001339.s003.zip › Anonymized/IDI_ND_XX_7.docx]

**Location**: XX-MI Village

**Date**: DD/MM/2020

**Time**: 3; 35 pm

I: So, what is your name?

R: HK

I: How old are you?

R: 14

I: Do you go to school?

R: Yes

I: In which class?

R: Standard 5

I: Alright.

I: Which school do you attend?

R: XX

I: XX what?

R: LEA school

I: What do you like at school?

R: I like learning, we go early to school to sweep the class and I go and welcome the teacher.

I: What is it that you like you like, what gives you energy to go to school everyday, what is it?

R: [pause]

I: What makes you say the school is good?

R: education

I: Which subject do you love?

R: Chichewa, English, Mathematics

I: that’s what you love?

R: Mmm

I: How many teachers do you have, those that teach you?

R: 3

I: Mostly at school they give us work to do like homework, assignments, how easy are these for you to finish them?

R: [pause]

I: Is it easy for you to finish or it is difficult?

R: I am able to finish all

I: Are there problems that you meet when you are going to school?

R: Yes

I: What kind of problems?

R: Parents showing abuses, they tell us not to eat meals, and when we go to the shop and tell them to buy us notebook, they don’t give us.

I: Are you staying with your parents now?

R: Mmm

I: Is she your biological mother?

R: No, my biological mother died

I: What about your father?

R: He is alive, he is stay at KC

I: What does he do at KC?

R: He stays with his relatives and working at a water company

I: So, you are just staying with your step mother?

R: Mmm

I: Does your father come to visit?

R: Yes, he comes.

I: Does he commutes everyday or he stays there?

R: He commutes daily

I: So, the way you have explained it shows as if you don’t relate well with your mother, right?

R: Mmm

I: You said that she can leave you without eating food, and go to school without food?

R: Mmm

I: How many are you in your family?

R: We are 5

I: All from same father?

R: Yes

I: So, how many are you from your father?

R: 5 of us

I: All of you?

R: Yes

I: This lady does not have children with your father?

R: Yes

I: I thought I saw her carrying a child, did she come with the child?

R: Yes, she came with the child.

I: When did you start staying with her?

R: Long time

I: Can you tell me, what do you do at home?

R: I wash plates, sweep the surrounding, fetch water and cook.

I: When you finish cooking?

R: I take a bath and then go to shop

I: Do you have a shop or hawker?

R: Yes

I: What do you sell?

R: Bread, fliters, Kamba, drinks, soap

I: Where is the shop?

R: Along the road

I: So, when you are here, who is in the shop?

R: My mother

I: Who was the woman I found here?

R: That one is my aunt. The other one is my granny and the other one is also my aunt

I: And when you finish doing that, what else do you do?

R: I come here to cook and also cook for the children and drop food for her since they sleep there.

I: In the shop?

R: Yes

I: So, as you’ve narrated, do you get a chance to play?

R: Yes

I: What time do you go to play?

R: I play in the morning, afternoon and in the evening, and when am tired we go and sleep.

I: You said you also go to the shop, so what time do you go to the shop?

R: We go there, like right now, we will eat and go to shop, and we will come in the evening and cook for the children and we should go and sleep.

I: So, as you are putting it up, where are you going to find time to play with your friends?

R: There no time

I: Do you have friends?

R: Mmm

I: How many friends do you have?

R: MX, AX, Gile, RX, another GX and SX.

I: Who is your best friend amongst them?

R: SX

I: Why is she your best friend?

R: I do chat with her.

I: But the others, don’t you chat with them?

R: I do chat with them

I: So, why is she your best friend?

R: Because when she tells me something, I can go and tell her mother and when I tell her something she also tells my mother.

I: So, is that good?

R: No.

I: Why is it not good?

R: Because it is those are lies

I: You said that she is your best friend but you go behind each others back and tell each others secrets to your mothers. What kind of things do you go and report for each other?

R: Quarrelling, insulting each other.

I: What kind of issues do you discuss with SX, because you’ve said she is your best friend, and that means you discuss some issues that you don’t discuss with any other friend?

R: I escort her to her granny’s place and we sleep there and when we are back they [ ….]

I: Why do you sleep there?

R: Her granny is the one who told us to do that.

I: Where does the granny stay?

R: CH

I: Why do you sleep there?

R: On that day she said that we shouldn’t go back home, and we said that no, we didn’t tell anyone and she said it was okay, our parents are not going to beat us, and we said it was okay and we slept there.

I: Alright. When you are at home, which things makes you unhappy?

R: Insulting me

I: Who insults you?

R: My relative Missy.

I: Mmm

R: Beating me, beating from my uncle

I: Why did your uncle beat you and why did Missy insult you?

R: He said I should not come home late but I go to the shop

I: Your uncle?

R: Yes

I: Was beating you because you came late?

R: Mmm

I: what else, what is it that you don’t like?

R: They abuse me, and when they chase me to go and play, I do go away and when they are talking about me I hear but I don’t react.

I: What kind of things do they talk?

R: Insults, telling me to go to graveyard and get my mother.

I: What is it that goes wrong for them to say that?

R: They say I don’t cook for the children but I do cook for them.

I: What else happens at home?

R: Poverty and shouting at me and my friends insulting me.

I: If you had a chance to change things at home, what would you change, what would you wish to change?

R: To listen to parents, stop being disrespectful, and coming home in good time.

I: can we say that you are disrespectful and you don’t listen to parents?

R: I do listen to them

I: Alright. What problems do you meet at school? You said lack of notebooks?

R: Mmm, when the teacher calls us we go there and when the headteacher calls us we do go, and when we are in the wrong side they tell us to go and call our parents.

I: What goes wrong at school?

R: Going to school late and beating up children

I: Do you fight with friends at school? Or someone threatening you? What happens at school? Does it happen to you?

R: Yes

I: Explain it to me

R: Beating up children or each other and later report to the teacher of the incident.

I: Has it ever happened that someone provoked you?

R: Mmm

I: Could you explain to me, what happened?

R: My younger sibling was sick and I went to the teacher to take permission to escort him home. My friend then said I was lying and that my younger sibling wasn’t sick and I only wanted to cut classes, that’s when we started to argue.

I: Okay. I wanted to ask where you and your friends chat?

R: We chat at his house, then his mother sends him for errands and I escort him. After that we go to the town and then we return and start plucking then eating mangoes.

I: As you have said that you go to your friend’s house then to the township and back. As young girls, what troubles do you face as you chat with your friends or your journey’s to your friend’s house, or in the way or when going to the townships?

R: We face people telling lies about us and when they do that, we tell their parents about it. The parents often tell us to hit them that we tell them that we don’t hit other peoples children.

I: Have you faced any other problems? For example, have you ever faced boys harassing girls, have you ever faced that?

R: I have

I: Could you tell me when you faced them?

R: Yesterday, I met some boys from some boys from (….), and they started threatening me. I told my uncle and he told their mothers that the boys were threatening me.

I: I wanted to talk to you about menstruation. Have you started menstruating?

R: Yes

I: If you can remember, could you tell me what happened on the first day. I also want ask if you had heard of menstruation before you started?

R: No

I: Now could you tell me what happened on the day it first started?

R: I sat on the veranda. As I sat there my stomach started to ache and I told my grandmother that my stomach was aching. She told me that you will start your period soon.

I: What happened after that?

R: Then it started to come out and drew water and bathed.

I: Okay, there after your grandmother told you that that it will soon start, and it started, right?

R: Mmm

I: So, after it started, whom did you report to?

R: My grandmother

I: And what did grandmother said?

R: She said, tear a cloth and put it on the panties and the blood will be soaking there.

I: Alright. Apart from being told by your grandmother, is there anything that you were told by other people about this issue?

R: Yes

I: Who was that?

R: My sister advised me

I: Who else advised you?

R: My aunt, and my other mother.

I: What happened, did they call all these people to come together and advise you or what really happened?

R: They first called me, and told me to be clean in what I do

I: That was your sister?

R: Mmm

I: What about the others?

R: They went to Boma and called me in the evening.

I: What about your friends, have you discussed this issue or you telling your friends about it?

R: Yes, I told her that I have started period today and she said okay, and she told me that she also started the same day.

I: Alright, issues of HIV, where have you heard about it?

R: At school

I: What do they say about HIV?

R: HIV and AIDS, we should not have sex with boys we might get pregnant, and we can contract the virus.

I: Apart from school, where else have you heard about HIV?

R: ?

I: Did you go alone at the hospital

R: No

I: How did you come to learn about HIV at the hospital?

R: They told me that I would have gotten very sick and died soon, you have a virus and I went home and told my uncle who went to the hospital.

I: Okay, where else do you learn about HIV?

R: No where

I: Do you have questions about HIV that you would like to know about HIV?

R: No

I: Have you ever heard about getting pregnant and avoid getting pregnant?

R: Yes

I: Where did you learn it from?

R: From my relatives, they told me not to indulge in sexual relationship, I might get pregnant before old enough to.

I: Alright. What else did they say?

R: That’s all

I: Do you have any other question that you would like to learn from them about how one can get pregnant and how to avoid them?

R: No

I: You don’t want someone to tell you that?

R: It is possible

I: Who would you love to chat with on such kind of issues?

R: My sister, my grandmother and my aunt

I: Why would you like to discuss with those people, not your friend or others?

R: They should tell me and I should listen to them

I: So, you cannot listen to your friends?

R: I can

I: Alright, when one is growing up, they see some signs like breast growing and other things; when you saw this, whom did you discuss with?

R: I discussed it with my friend.

I: What were you discussing?

R: I told her that I have started growing hair and breast, and she told me that I will get pregnant soon and I said no, it will not happen.

I: Your friend told you that?

R: Mmm

I: What about the issues of girl/boy relationships, are they common here?

R: Very common

I: Why do you think girls have such relationships?

R: When their parents tell them not to have such relationships, they act rudely to the parents.

I: Alright, have you ever had a relationship?

R: No

I: Or has a boy ever proposed to you?

R: Yes, but I said no

I: Why did you say no?

R: I told him that I have not grown to the extent of having relationships.

I: Alright, is there anyone you like to talk to; explain to you issues of relationships between girls and boys?

R: No

I: Alright, we are coming to an end, do you have anything that you can tell me?

R: No

I: Alright

END
