# Supplementary material for: Social support and very young adolescent girl’s knowledge on sexual relationships: A comparative qualitative study of Girl Only Clubs’ participants and non-participants in rural Malawi
Source: PLOS Glob Public Health. 2023 Jan 12;3(1):e0001339. doi: 10.1371/journal.pgph.0001339 (PMC10022037; doi:10.1371/journal.pgph.0001339)
Supplement: S1 Data — (ZIP) [file pgph.0001339.s003.zip › Anonymized/IDI_ND_XX_8.docx]

**Location**: XX-MI Village

**Date**: DD/MM/2020

**Time**: 4:20 pm

I: First thing first, can you tell me what your name is?

R: SL

I: How old are you?

R: Twelve

I: Do you go to school?

R: Yes

I: What class are you in?

R: Standard 6

I: Why do you go to school?

R: I want to have a job in the future

I: You want to have a job?

R: Yes

I: Why do you want to become a nurse?

R: I want to help the sick

I: In your village, do you have anyone who you look up to who became a nurse?

R: No

I: There isn’t?

R: Yes

I: In your village, is there anyone you look up to?

R: Yes

I: Who

R: j

I: Where does he stay?

R: Ms

I: What does he do?

R: He works at education?

I: He works at education?

R: Yes

I: Why do you look up to him?

R: Because he has a job and earns money from it and uses on his household

I: What do you like at school?

R: Learning

I: Is there another?

R: Playing with my friends

I: What troubles do you face at school? What bothers you when you go to school?

R: I go to school without eating. I also sometimes go on barefoot, sometimes I have no body lotion and I also don’t have pens and books.

I: Is there anything else?

R: (Long pause)

I: What troubles do you face with your friends or the people you meet at school?

R: They refuse to lend their pens to me when I ask them to. Some also discriminate me because I walk barefooted.

I: Is there anything else?

R: (Long pause)

I: Who do you live with at home?

R: My mother

I: Anyone else

R: Dn

I: How many siblings do you have?

R: Two

I: Who is older between you and your siblings?

R: The oldest is Dn

I: And then?

R: En

I: And then?

R: Me

I: You’re the lastborn?

R: Yes

I: Is your mother the woman I found outside?

R: No

I: Where is your mother?

R: At home

I: So you have here just to visit?

R: Yes

I: What about your father, is he around?

I: He passed away

I: When did he pass away?

R: When I was a child

I: Your mother did not remarry?

R: Yes

I: What do you like about living with mother and sisters?

R: They help read

I: What else?

R: When we play

I: What happens when you play?

R: I like it

I: Do you have friends?

R: Yes

I: Who is your closest friend?

R: Ms

I: Why are you very close to Ms?

R: She advises me

I: She advises you?

R: Yes

I: What other secret things do you discuss with M?

R: We play ball and when I ask her anything she answers me.

I: What else?

R: We walk together and When either of us has a problem, we tell each other about it.

I: Could you give me an example of anytime when either one of you was dealing with a problem and told the other about it?

R: She told me that she was having periods. I told my mother and she told her that she should wear a rag.

I: Your mom called for M?

R: Yes

I: She advised her?

R: Yes

I: Have you started to have periods?

R: No

I: You haven? Apart from when your friend told you she was having periods, have you ever heard of this topic elsewhere?

R: Yes

I: Where?

R: At school

I: Who was talking about this at your school? Was it your friends or your teachers, who was talking about this?

R: It was a certain group of people who (Long pause)

I: Was it an organization?

R: Yes

I: What did they say?

R: They said when a person has a period they have to wear a pad so the blood should fall on the pad. After that we have to throw it away.

I: How do you feel about having periods taking into consideration that you have not started having them?

R: It frightens me

I: Why is that?

R: I fear I might die

I: That periods might kill a person?

R: Me dying

I: Because of periods?

R: Yes

I: Why do you think you can die from having periods?

R: I feel like blood might run out in the body

I: Have you ever talked about this with your mother, so she should encourage you?

R: No

I: Have you ever talked about it with your friend or asked about what is happening to her?

R: No, she never told me

I: You said that your closest friend is M. When you knock off from school and finish your chores where do you and your and friend go to play?

R: At the ground

I: Where else?

R: At her house

I: We wanted to know about the troubles you face when you go to play.

R: Some curse at us and some gossip on us.

I: Who are those? Your friends?

R: Yes

I: Anything else?

R: Some say we gossip because we always walk together.

I: Okay. In other areas young girls face problems like being proposed to or threatened by boys or some get raped or proposed to by older men or some have uncles or relatives who sleep with them. How often does it happen?

R: It doesn’t happen a lot.

I: But does it happen?

R: No

I: You’ve never heard of this happening?

R: Yes

I: Okay. What problems do you face at home?

R: We sometimes get hungry, sometimes we don’t have soap or lotion

I: What else?

R: Sometimes we walk barefooted

I: What work does your mom do to provide for your family?

R: She does small jobs

I: What jobs does she do?

R: She farms

I: Where?

R: People call her to farm on their lands and she uses the money she gains to buy us maize?

I: Maize which u eat?

R: Yes

I: I wanted to ask about HIV and AIDS. Have you ever heard of it or any other Sexually Transmitted Infections?

R: No, I haven’t heard of it

I: You haven’t heard of it?

R: Yes

I: At your school?

R: I heard about it at school?

I: You heard about it from school?

R: Yes

I: About AIDS?

R: Yes

I: Who did you hear it from, the teacher or your fellow students?

R: The teacher

I: What did she say?

R: AIDS is transmitted through unprotected sexual intercourse

I: What else?

R: Sharing razor blades, needles and toothbrushes

I: That was in class?

R: Yes

I: Where else have you heard about this disease?

R: Nowhere else

I: You don’t discuss it with your friends?

R: Yes

I: Who would you want to talk to about it as girl if you ever wanted to more know more about the topic?

R: My mom or my friend

I: Why would you choose to discuss it with your mom?

R: If it was with my mom, no one would spread word on what we talked of but if it was with a friend, they might tell someone else what we discuss.

I: So you would choose your mom only?

R: Yes

I: You said you stay with your mom and you chat with your friend M. Who else do you interact with?

R: My uncle and my sister

I: Who else?

R: My aunt

I: In this whole village, you only talk with M?

R: No, I have other friends

I: Where do you go to play with your friends?

R: We play ball, we go to the bank and we play pretend games?

I: You play `zawana`?

R: Yes

I: I wanted to know if you ever heard of the topic of pregnancy or avoiding pregnancy.

R: I have never heard of it

I: Your parents never talked to you about it?

R: Yes

I: What about your friend?

R: My friend told me that you can get pregnant from having sex

I: What happened for you to discuss that topic?

R: My friend told me someone had gotten pregnant and I asked her if she was there when she got pregnant. She said no and then I asked her what happens for a person to get pregnant and then she told me.

I: What else have you hear of this topic like prevention?

R: I haven’t?

I: Has your school ever talked about this topic?

R: Yes

I: What did they say at school?

R: To reject boys who ask for relationships and to report boys who pull us in the bushes and they would deal with them.

I: Has any boy ever asked you to be in a relationship with them?

R: No

I: No?

R: Yes

I: Nobody has ever asked you?

R: Yes

I: Do you know the people who are in relationships in this village?

R: No

I: It doesn’t happen that often here?

R: Yes

I: Is there anything you want to know of this topic of relationships between boys and girls which would help you stay away from trouble?

R: Yes

I: What would you want to know?

R: When boys ask for relationships I should turn them down

I: What else?

R: What to do to avoid getting pregnant

I: Is there anything we didn’t talk about today but you would want to let me know of?

R: I don’t know

I: There isn’t any?

R: My mom sometimes gets sick in the head and we loose the support of our mother.

I: Is it like madness?

R: Yes

I: Who takes care of you when that happens?

R: My older sibling

I: Is there anything else you want to tell me ?

R: No

I: Thank you and what we have discussed today will help us bring programs to help young girls like you.

END
