# Supplementary material for: Social support and very young adolescent girl’s knowledge on sexual relationships: A comparative qualitative study of Girl Only Clubs’ participants and non-participants in rural Malawi
Source: PLOS Glob Public Health. 2023 Jan 12;3(1):e0001339. doi: 10.1371/journal.pgph.0001339 (PMC10022037; doi:10.1371/journal.pgph.0001339)
Supplement: S1 Data — (ZIP) [file pgph.0001339.s003.zip › Anonymized/IDI_ND_XX_9.docx]

**Location**: XX-MI Village

**Date**: DD/MM/2020

**Time**: 9:15 am

I: First thing I want you to tell me your name?

R: SMX

I: How old are you?

R: 14

I: Do you go to school?

R: No

I: When did you stop going to school?

R: Last year

I: What made you to stop going to school?

R: We were not going in good terms with mother, she wasn’t giving me soap, pens, books and I thought on how I would survive at school, and how I would everything

I: Mmm

R: So, I just became confused and at that time I had a boyfriend, and this boyfriend who was giving me soap but not notebooks. And at the time when he asked to have sex with me, it was difficult for me to say no because he was the one supporting me. But now we break the relationship with each other but he left me with pregnancy.

I: You said that, your parents were not giving you support that you wanted and you met this boy, who was giving you soap and he asked to have sex with you and you failed to say no because he was giving you soap.

R: Mmm

I: At that time you were staying with your father and mother?

R: Yes, I was staying with both and I still live with both mother and father.

I: What do your mother do?

R: For my mother it depends on season, during avocado pear season she buys them in bulk and sells them, during banana and potato season she buys in bulk and sells them, that’s what she does.

I: Both of them?

R: No, my dad is a carpenter, he is called for piece works and even when he finds money he doesn’t take care of any one other than that sister of mine the one who has grown up with […]. And at that time only my mother was providing notebooks and clothes but he wasn’t doing anything and at that time I just became confused. Everything that we were being provided for was done by my mother and things were hard for her to find and provide to us.

I: And at the time you were dropping out, in which class were you?

R: Std 7

I: But you would want to continue with your education?

R: Yes

I: Okay. You said that you married the guys? Where did you meet this man?

R: Here, at our neighbor’s place there was a houseboy who had his relative who was coming with a friend to visit. And in the midst of that he became my boyfriend.

I: He was from your neighbor’s place, right?

R: Mmm

I: How old is he?

R: I don’t know

I: Does he go to school?

R: No

I: What does he do?

R: Nothing

I: So, how were you staying with him, how was he treating you and how long did you stay with him?

R: I didn’t stay with him for a long time. He has a bad heart, he used to beat me everyday and the time I was going there my mother chased me, and after some time as a mother she came to pick me up. And now that am back they give me soap but it is difficult to get and sometime I put on same dirty clothes, and when they found money she gives me and I buy soap and then I will watch my clothes.

I: I want to know what happened; when you were going to stay with your boyfriend did your parent agree that you go there or what happened?

R: My mother was furious and didn’t want me to go there, but because of how I was being treated here, most of the time things were not well between my mother and myself, most of the times she was not giving me food. And then I thought that if I will keep on staying here how will I survive since I wasn’t getting food and soap, so I decided go in marriage.

I: And you just went in marriage?

R: Mmm

I: So, as in marriage you became like the woman of the house, what was happening there, were you taking care of the man, cooking for him?

R: Yes, I was cooking for him, washing for him, and I was doing everything for him.

I: And you were saying that the man was doing nothing?

R: Mmm, he was just doing piece works in people’s gardens.

I: So, you were able to find food at that place?

R: It was available, and I was eating properly.

I: So, why was he beating you?

R: He had a lot of girlfriends and when I tried to talk about he was telling me who am I to tell him that and was just beating me every day, I was looking as if I was wrong, up until people knew about what was happening and even here, my mother leant about it and she just came to pick me up.

I: So, the issue; why you were being beating was because he had extra marital relationships?

R: Mmm, some of them were my friends and they would come to make me feel pain

I: They would do that to make you feel pain that your boyfriend is also their boyfriend?

R: Mmm

I: Alright, and you said you drop out in which grade?

R: 7

I: Okay. Can you tell me a little about your school life? What was exciting for you to be going to school?

R: I knew that if I stay here at home I wouldn’t know more things like reading and writing, I knew how to read and write things that I wouldn’t have known if I didn’t go to school. At school I liked reading and writing and I also wanted to go further with my education, but it has failed.

I: Whom would you want to be when you finish school?

R: I wanted to become a nurse.

I: Why did you wish to become a nurse?

R: I feel pity when other people are not being nursed properly, where one need help but the nurses are chatting without helping them, and I wanted to take care of such people.

I: Is there anyone in the community whom you look up to, whom you admire to be like her?

R: In this village, we only have teachers.

I: Whom went far with school?

R: Mmm

I: someone you admire to be like them?

R: There is someone who is doing well but is not educated.

I: What does she do?

R: I don’t know how they make their wealth.

I: Alright, do you have friends?

R: Mmm

I: How many?

R: More

I: Which ones do you get well with them very well? How many are they?

R: 5

I: Have you ever told your friends about the problems you met when at home that made you go in marriage? Or whom were you telling your problems?

R: There is one girl whom I was able to tell everything and she was able to give me food when her parents were there, she wasn’t able to give me food.

I: Alright. Was there anyone whom you were free to tell them your problems, that you’ve found a man and anything from your heart?

R: The same person

I: What did your friend advise you?

R: She said I go and see if I can tolerate the man or not, but she cannot afford to help me anymore and she told me to go, because there is nothing else I can do?

I: Is your friend continuing with her education?

R: She finished long time ago, maybe 5 years ago, but she cannot find a job.

I: How many children are you?

R: 4

I: Who is the first born?

R: Myself

I: Are your siblings still going to school?

R: Yes

I: The one who came after you, in which class is she?

R: 5

I: Alright. I wanted to know; when did you start menstruating?

R: 20XX

I: Before that, have you ever heard of stories of menstruation?

R: By that time, I never heard of anything, but after that people told me what I was supposed to do and what I wasn’t supposed to do. I knew about it after I already started having it.

I: Can you tell me how it happened on the first day?

R: I was sent to go and buy cabbage, and I went there and I wore a jean short and I felt friction between the legs and I thought the blood I saw came from that, and when we reached home I told the people I was staying with since that time I wasn’t staying with my parents.

I: Whom were you staying with?

R: I was staying with my aunt, my dad’s sister in Thyolo, and when I told her she told me to go and wash myself and she treated the wounds from friction but blood never stopped coming out. And because they were elderly they knew and they told me w2hwat ii was supposed to do and what I wasn’t supposed to do, and they told me that I have now come of age.

I: Alright, after that, what happened later? Did she call other people to come and talk to you?

R: No, she first told me and thereafter my grandmother, and other 3 women who were staying with us at the same place, they called me and explained to me. Some other parts were the same some where just giving me bits of the issue and some explained all.

I: At the time you were going to school, such things were they discussed at school or not?

R: In one term, in August, after Grade 4 going in Grade 5, during life skills subject I also heard about the same thing. But I started in August, holiday time but when I started school, I heard about it during life skills subject. And the one who was teaching us didn’t hide anything and I was able to relate what I heard from home and what I was taught in class, and the issues were not that different. And when I went home I told my Asisi that what you told me I have also learnt it at school and she agreed that it was the same issue. And some of the issues I knew them from life skills.

I: What about discussing it with your friends?

R: We do discuss

I: Before starting or not?

R: After starting it. Sometimes you would want to ask for pads from your friends when you don’t have and that’s when she will tell you that she already started having it too. And when you don’t the pads, you share with each other.

I: I wanted to about issues of getting pregnant or avoiding pregnancy, where have you heard about this the first time?

R: I heard about what happens when one gets pregnant but one cannot get pregnant when she has sex using a condom, if the sperms enters your body you might get pregnant but if they don’t enter in your body you cannot get pregnant. But about what happens when one gets pregnant I don’t know.

I: Even when you were married, you never discussed it with anyone?

R: They just told me that when one is pregnant, you don’t do your menstrual.

I: Where did you learn it from?

R: My friend when we were chatting. I asked my friends how one would be noticed if she is pregnant. That is when they told me that some are seen because of change in complexion, some you get fat, some elders would ask you to show them your palms, and sometimes you don’t do your period and that is how I knew.

I: Have you ever heard or taught about such issues at school?

R: Yes, one time female teachers called us all girls at school and they explained that.

I: What about during life skills subject?

R: They do explain but some teachers explain explicitly but other hide some facts.

I: Why do you think they hide some things?

R: I don’t know but some teachers explain things properly while other just cut them through and in that way you may not understand things, but others explain very well from the roots to the end while others do not.

I: Do they allow you to ask question when they finish teaching?

R: Yes, but others who don’t explain things when you try to ask them that you didn’t understand they tell you, that you will understand when you are in heaven.

I: I wanted to know; were you not afraid that you will get pregnant when you go in marriage or you’ve given birth before?

R: No, I have never given birth and I have never been pregnant before, but I just told myself that whatever happens, it is okay, what else can I do, how will I survive, my mother loved that, they can starve you not for a single day but they can do that for a week.

I: Was it that the food was not there or what?

R: Food being there, but maybe I have washed plates and mopped the house and maybe I didn’t sweep the surrounding then she will not give me food for that. And maybe if my younger sister is the one cooking she would give me while my mother is not home, but if my mother tells her not to give me, she will not give me.

I: Is that one your biological mother?

R: Mmm, and the father is also a biological father.

I: Let me take you back to the time you were married. Were you not thinking what will happen to you when you get pregnant?

R: I wasn’t thinking about that, I just thought that all was good as compared to how I was being treated at home.

I: I wanted to ask about issues of HIV/AIDS? Have you ever heard about it, where was the first time you heard it what happened?

R: Mmm, I heard about it at school. And when I reached home I told my friend, I started chatting with her when I was young and she was older, that time I was in Std 3. And I told her that we learnt about HIV at school. And I asked her what AIDS is, and she told me that it was an illness that one contract through sexual intercourse. But I knew a lot from Std 5 to Std 7, that when I knew properly about AIDS.

I: You knew about it through school?

R: Mmm

I: When they were teaching?

R: Mmm

I: In this community, do you have clubs where young people like you go to learn about pregnancy and HIV?

R: No, we don’t have

I: It’s only at school?

R: Yes. And when you have someone who likes within the community can explain to you but mostly we learn much from school.

I: Maybe discussing that with parents; like you father or mother about such issues?

R: Parents from this community, they cannot explain things that we learn at school, sometimes they can and sometimes they also cannot explain, and that makes you to opt not to ask again since you know they will not explain.

I: You said that there is no where you learn about this but only at school, right?

R: Yes

I: But you also discuss this with your friends? When you are chatting with friends, do you discuss HIV/AIDS issues?

R: No

I: You don’t?

R: Mmm, so girls says rudely that it is better to contract syphilis other than HIV, if one gets syphilis, eee things will be bad for her than HIV, but we don’t talk such issues very well.

I: Alright. When one is growing up the body changes and one may notice breast growing, pubic hair in the armpits, did you discuss about this with any one?

R: My mother, at one time I was sick, I had small sores on my body and was weak to walk, my mother used to bath me and saw that and she asked what was it and I told her that I don’t know and that when she explained to me that I should not get scared because that is what happens when one is growing.

I: So, when it happened you were not surprised?

R: Yes, because when it was happening, people already told me about that.

I: Did you discuss with your friend about this?

R: Most people hide such issues, to find someone to tell you or show you it’s here and there, they will tell you I don’t have when they do have it.

I: Alright, I wanted to know; when you were going for marriage there was no one who came to tell you anything?

R: Mmm

I: You just went there and start marriage?

R: Mmm,

I: There were none who came to tell you that it was not a good decision, you should go back?

R: No one came to tell me that

I: They just left you?

R: Mmm

I: your father didn’t say anything?

R: Mmm

I: Your mother didn’t say anything?

R: Mmm

I: And tell me what happened for you to come back home? Your father and mother didn’t talk to you when you came back?

R: She said that; ‘you did what you wanted, and how were you treated there, now you have remembered me’; whatever she was saying there was not good, and she was not advising me.

I: Alright, I would like to know about boy/girl relationships; I would like to know if the man you were married to was he your first boyfriend or was the second one?

R: It was the first

I: At the time you find this relationship, have you ever heard of boy/girl relationships at the time when you had this relationship? Maybe you were able to chat with friends, how they go about, the advantages and disadvantages?

R: I didn’t know about advantages and disadvantages but my friends told me that they also live like that, soap, and body lotion is provided by their boyfriends and notebooks, and the way I was being treated at home.

I: So, your friends were the ones who were telling you that?

R: Mmm

I: That they are being provided by their boyfriends?

R: Mmmm

I: They had relationships?

R: Mmm

I: So, they told you the boyfriends were buying them soap, oil?

R: Mmm, and even notebooks

I: And told me that you were chatting with a friend from that place, she also had a boyfriend?

R: I don’t know at that time [………………] she didn’t tell me that she had a boyfriend, she never told me.

I: Okay. Thank you. As a girl, with these issues of boy/girl relationships, issues of HIV/AIDS most of the times, girls do not know much and you may want to know more, in this community, whom would girls of your age would want to discuss such issues with?

R: To me, I think for me to have good manners, then it should be an elder person and not young because they will be telling me bad things as thy had been telling me.

I: It is better someone older to be telling you?

R: Mmm

I: Now, what are the issues that you would like to know properly and you want someone older to tell you?

R: Like HIV issues, I don’t know all and I would like to know the medication for it, and also to know how people know that they have HIV. Because I hear that peo9ple have straight unhealth hair and how can we know if someone has it or not.

I: What else?

R: Issues of getting pregnant; what happens when one gets pregnant.

I: you as a girl who got married and came back, what would you advise your friend on issues of relationships? Because you have been through it?

R: It is not good, and there is no good thing from it. One looks as if you are old while you are young, and you do work that is not meant for someone young, and I am just asking my fellow girls not to do that, there are problems there.

I: When you were married, you had your in laws, how were you staying with them?

R: We were in terms

I: Why?

R: I don’t know, sometimes she would respond sometimes not, but also came a time when she was responding to my greetings and we could stay together.

I: Were you staying in your mother’s in law house or your own?

R: Our own house

I: Mmm Do you think of going back?

R: No

I: Why?

R: I figured it out that I was doing childish things but now I know where to go when am hungry like at my ground mother’s place. At first I thought she can stop me but now it is okay.

I: Alright, thank you for answering the questions, is there anything that you would like to tell me about yourself before we close; maybe I didn’t ask but you would love to tell me?

R: No

I: thank you. I forgot one thing, you said you dropped out of school last year, right?

R: Yes, when corona was coming I was already out of school.

I: Do you have thoughts of going back to school?

R: yes, I would like to go back to school

I: Do you think you will start school when they open?

R: I thinking so, but am wondering if it will be possible

I: What do you mean?

R: Are people not going to notice that am pregnant?

I: Oho, I didn’t know, you are pregnant?

R: Mmm

I: Okay. How many months?

R: I think 3

I: What does your boyfriend say about it?

R: I didn’t tell him anything

I: He doesn’t know?

R: Yes, but I don’t want to go and stay with him

I: Right now you are pregnant, but you don’t want to stay with him?

R: Mmm, I don’t want marriage

I: You want to stay at your parents house with the baby?

R: Mmm

I: So what are you thinking, what are your concerns right now that you are pregnant?

R: Although it happened this way, I didn’t want to have this pregnancy, and because it happened, I accepted it.

I: Yes, you accepted it that you aare pregnant, and you will have a baby, now what are your concerns?

R: [pause]

I: Do your parents know?

R: I think they don’t

I: So, maybe what is frightening you or giving you worries?

R: That if my mother discovers it I don’t know what will happen.

I: What else?

R: I don’t know if I will manage to get things for the child to use at the hospital, if I will find them or not?

I: And when you think of the baby, that you have a child growing inside you, and what will happen when you are giving birth? How are you looking at it?

R: I don’t know will happen at the hospital since am still young

I: When are you starting antenatal clinic?

R: I don’t know

I: Alright, so, have you talked to anyone older about this pregnancy?

R: I just know it by my self

I: Alright.

END
