# Supplementary material for: Social support and very young adolescent girl’s knowledge on sexual relationships: A comparative qualitative study of Girl Only Clubs’ participants and non-participants in rural Malawi
Source: PLOS Glob Public Health. 2023 Jan 12;3(1):e0001339. doi: 10.1371/journal.pgph.0001339 (PMC10022037; doi:10.1371/journal.pgph.0001339)
Supplement: S1 Data — (ZIP) [file pgph.0001339.s003.zip › Anonymized/IDI_ND_YY_1.docx]

**Location**: YY-NO Village

**Date**: DD/MM/2020

**Time**: 10:15 am

I: As I said earlier, we have come here in YY- NO village, so that we can have a chat with girls from 10 years old to 14, on different issues that concern your lives. Can you remind me how old are you?

R: 14

I: Alright, do you go to school?

R: Yes

I: Where?

R: CU

I: In which class are you?

R: 7

I: You said you are in Standard 7, right?

R: Yes

I: What is it that you like at school? Which subject do you like?

R: English

I: Why do you like English?

R: I should know how to read and know learn things. I cannot choose Chichewa since I know it very well, but English.

I: And which subject do you struggle with?

R: Mathematics, Expressive Arts and Social.

I: Why do you struggle with these subjects?

R: When they are teaching them, am always alert to listen but there is nothing I get in the end. They can teach and tell us that they will come again to ask questions; after that I would concentrate so that when they are asking questions I should answer them; and who answers them should go home; mostly I do not pass, and it end up that they tell us that maybe one day we will pay if we work hard. I do work hard so that maybe one day I can pass but I still fail.

I: So, they sometimes tell you that those who will pass will go home?

R: Sometimes they do that so that for us who have failed should stay and be trying, but we still fail.

I: When you are home, are you given something to work on?

R: Yes, homework and we are given work to do with our older relatives who finished school. But it is not all the time, it also fails sometimes.

I: What about your friends? Solving Maths at home, do you do that at home?

R: The problem is that we are only 2 who comes from our village to that school, the other go to another school. Some go to N and I go to C school, and for me to tell those who go to N to study with me; they tell me that they don’t go to same school with me. It was only that friend of mine, but now she moved to B, on my own it doesn’t stick in my head when I solve the maths.

I: So, you don’t have other friends?

R: They don’t stay near.

I: So, whom do you chat with, when you want to chat?

R: My friend, but she dropped out of school in Form 3, that whom I chat with, but she also don’t know Maths very well.

I: So, that’s the one you chat with mostly?

R: Yes

I: Is she your agemate?

R: No

I: How old is she?

R: Maybe she is 18 years old

I: 18?

R: Yes, her mom died and there is no one who was paying for her fees and she just dropped out.

I: How far does she want to continue with her education?

R: She just dropped out because of money problem; she said she cannot continue without school fees.

I: What are her plans?

R: If there was someone to pay for her fees, she would have been going to school.

I: Maybe she is thinking of getting married?

R: No, she just stays.

I: So, apart from that one, who else do you chat with?

R: I do chat with other girls, but we don’t connect very well.

I: What do you mean?

R: Doing different things when we have agreed another.

I: What things; I want to hear that?

R: [laughs] oh, when you tell them ; let us study; they are going to say; we don’t go to the same school, you can go and read with your friends.

I: Alright, I wanted to know much about your family. Whom do you stay with?

R: My father and my mother.

I: How many are there in your family?

R: We are three

I: Who is the first one?

R: Myself

I: Who are the others?

R: A girl and a boy

I: How old are they?

R: The other one is 11 and the other is 8

I: Mmm, okay. Tell me, when you are home, what do you like to do?

R: I study

I: What else do you do? For example, today; like how does your day go by?

R: For example, here in the village, we go to the gardens, and after that if it is school day we come and sweep the compound and by that time water is boiling on the fire and we take a bath and go to school. After that we found food prepared and because the parents prepared it in the afternoon, we make sure we prepare the evening food.

I: You said you have a 11 year old sibling, right?

R: Mmm

I: A girl?

R: Mmm

I: All girls, 3?

R: No, 2 girls and a boy

I: The boy is the 8 year old?

R: Mmm

I: Aha, so when you are saying you share household chores, you mean the other girl?

R: Yes

I: How is your relationship with your mother and father? Between the two whom do you like most?

R: Whom I like most?

I: Mmm. Whom do you get well with very much between your mother and father?

R: Both of them, since I do not look down on any of them, so maybe if I was of that kind of looking down on people then I would have trouble with one of them.

I: Do you chat with your father?

R: Ooh yes

I: What kind issues do you talk about?

R: School issues, him telling us to continue with school and that he didn’t continue with education himself, and maybe if we continue with education, in future we can help him.

I: And your mother?

R: The same issues

I: Everyday, they say the same things?

R: Nooo, some of the issues not only school, sometimes my mother would want to see school work since she knows how to read, so I can give her, although she also has a problem with English.

I: Your mother knows how to read? How far did she go with her education?

R: Standard 8

I: Your father?

R: My father Standard 4

I: So, he doesn’t know how to read?

R: [laughing] he doesn’t know

I: So, you mom knows how to read and she helps you with some of the things?

R: Yes

I: So, you as a girl, when you are home with your siblings and parents; a person sometimes gets disappointments, what is that disappoints you, or things that makes you wish you were born in that family other than yours? What is it that disappoints you at home?

R: Nothing, I don’t even wish if I was born in another family, I should not lie.

I: You have never been disappointed?

R: I do, when someone has said bad things about me, I can be disappointed, I would start thinking why that person said those words and I sit down and ponder about it and finds no answer to it.

I: Can you tell me of one incident when something happens and you were disappointed?

R: One time, they told me to go the garden, and I told them that I don’t want to go that day, and they told me that I will not eat food that day and I didn’t eat and I ate in the evening.

I: Who said that?

R: My mother

I: So, she denied you food?

R: Yes, but my father was not around.

I: Has your father ever disappointed you?

R: I asked my father to go to Christmas gatherings and he said no, and I went there despite him saying no, and he came to pick me up from that gathering and I was resisting and he whipped me, and it was years back, 2017. Since that time I fear him.

I: Why did he refuse for you to go to Christmas party?

R: It was because my friends I had that time were of bad behaviours, some of the promiscuous, some right now are married though under age, and some has children, and he didn’t want me to be part of that group. And since after that he has never beaten me again.

I: Can you tell me what happens at Christmas party and where do you go?

R: At the center called Mthundu

I: What happens there?

R: There is music system and when you have money you will buy your food and chat with friends and when morning comes then you go home. Some people gets into dancing competition for those who know how to dance, as for me I don’t know how to dance.

I: What are good things that happens there that the youths love? Do youths love to go there?

R: Boys go there and girls too.

I: What happens there?

R: People dance, and for others who are in relationships they meet there and my father thinking of my future that is when he denied me going there for the fear that I might influenced by my friends.

I: Have you ever gone there before?

R: I went there.

I: At that time what happened for your parents to allow you to go?

R: Before he whipped me. At that time it was when I went there around 2 pm and around 4 pm I was going back home. But the time I wanted to go there with my friends, it was in the evening after I had supper, I thought that I will be coming together with my friends when they are coming back.

I: Which other place apart from Christmas where youth usually go to have fun, apart from Christmas time?

R: At Mkhalabwazi where we do youth activities, some youth chat there.

I: What are the things that are done there?

R: They teach boys and girls, not to engage in sexual activities and to avoid getting pregnant when we are still young, and other more things. But others don’t go there to learn but just chatting. They say we should just go to Save and kill time there.

I: It is run by Save?

R: Yes, it is known as Save.

I: What are bad things that happen there?

R: Some people engage in fights on petty issues, and sometimes they give us books to read when we are home, and people steal from each other and this can also be an issue for some to fight.

I: Alright. We were talking about what disappoints you when you at home, but now what excites you at home.

R: After school, I like it that I live well with my parents because there are other people who doesn’t stay well with their parents, but I don’t ask them and I have never found an answer why they doesn’t stay in harmony with their parents. Sometimes others would be quarrelling with their parents daily or sometime fighting with their own mothers, so I just ask myself why would someone fight her own mother when she was born from her and without the mother she wouldn’t have been born.

I: Alright, I wanted to know, what does your mother and father do for you to survive at home?

R: They do piece works, when my father finds a piece work maybe in the garden, they go there together so that it doesn’t take time to finish and my sister and I will be gardening here little by little, so that whatever they bring from there then we can find food to eat.

I: What are your responsibilities as the first born of the family, what do you do?

R: When I wake up?

I: Mmm

R: I do cook, fetch water so that I can relieve my mother of some of her duties.

I: Mmm

R: We are two girls, and we share the house chores and she doesn’t look down on me; for example, Saturdays, I can tell her if want to cook lunch or supper and if she chooses lunch the we will fetch water together, and other things together and she will cook lunch and in that way we have relieved mother of some of the duties. And she appreciates us that we have helped with cooking.

I: Alright, in the village like this; it is possible girls meets somethings; there are places which are not good and in other areas, it can be found that girls has been raped or sometimes girls troubling girls or men troubling girls. How common are such things?

R: It is common, I just hear that such such a girl has been raped by her father and I just of how such things can happen as to how would someone do that since I have heard twice this happening to a girl with her biological father, but the issue will just die like that, and do not go far.

I: Step father?

R: No, biological father

I: Raping them?

R: Yes

I: Where do girls go to play?

R: We go to Save and chat, that’s the only place we go and chat.

I: Is there any place where you are afraid to go there?

R: Yes

I: Where?

R: At IS, to go there at night, some men drunk and other not drunk would be saying insults such as I would want to deal with you one day, or I want you and you should be denying. And some men would tell you that we should not meet, if we meet I will do anything I want with you and because of this I tell myself just to hang around Save and chat there until the sun sets and then I go home to cook.

I: What about boys proposing to girls?

R: It is common

I: Had it ever happened to you?

R: Yes

I: Can you tell me where it happened, where was that place?

R: I was coming from school, Kasimu, a boy proposed to me form Issa where I said I am afraid going there. But this boy didn’t not threaten me that he will bad things if meet at night, no. And when he proposed and I said no, he beat me, and I am afraid of him since that time, I always thing what is going to do to me if I meet him again.

I: This boy from IS?

R: Yes

I: Was it the first time?

R: No, it was for a month and kept saying no.

I: How was he proposing to you?

R: He was writing a letter and gave it to a friend who was delivering it to me. At first, I read it, and later I was just tearing them, I wasn’t responding them.

I: So, what happened for him to beat you up?

R: He said that he was annoyed because of my denials.

I: So, he just found you?

R: I met him on the way.

I: Where were you going?

R: I was going to fetch water

I: Mmmh

R: He called me and told me to stop and I asked why, and he said I was stubborn, and he caught up with me and asked me why I don’t answer his letters and I told him I cannot respond to such foolish things. And when he says somethings I was stubbornly answering him and that’s when he slapped me twice and run away.

I: so, what happened after beating you?

R: I continued to fetch water at the borehole and went back home and I told my mother, and he refused that he slapped me.

I: Why did refuse his proposal?

R: If I said yes, boys nowadays doesn’t want to have a relationship and end it before they sleep with you and if you sleep with him will impregnate you and you will suffer with the child.

I: Who gave you such ideas?

R: My granny advised me when I first had my period, so she advised me.

I: What did she say?

R: she said that when ones starts having the menstruals, sometime you have desires to have sex and you can get pregnant, and even after the period if you sleep with a man you can get pregnant.

I: Alright, I wanted to understand this, I would like to know what happened when you started having them? Did you have knowledge prior to having them or you had the period first?

R: It was my first time, and I was at school.

I: You never heard about it?

R: Yes

I: So, tell me what happened, I want to hear your stories?

R: After taking a bath going to school. At school, while learning I was just surprised to feel wet, and thought I had wet my pants. And when I went to the toilet, I found my pants with blood but I didn’t have any wound and I wasn’t shy, I went and tell Madam, and she told me to go home. And when I went home I told my grandmother first.

I: Your grandmother stays with you?

R: Now, she moved out.

I: Mmm

R: I told her and she told my mother and she gave me the pads and when I wore them that’s when she advised me.

I: What did she tell you?

R: She asked me what happened at school. I told them what happened, and that’s when she told me that I am now grown up and when you are experiencing this, you start having sexual desires and you can get pregnant, and also after having period if you want to have sex you can also get pregnant.

I: I wanted to know how you felt when you felt when you saw that you soiled you pants at school? I want to know how you felt, were you afraid?

R: I was afraid since it was my first time, and thinking that I didn’t have any wound, I wasn’t feeling okay.

I: Why didn’t you tell your friends and you chose to tell Madam? Do you have a close friend?

R: Yes

I: Why didn’t you tell her? I just want to know?

R: I wanted to but I didn’t know if she would help me, I wanted to tell Madam first and later my friend. And after I told madam, she told me that I should go home, and I didn’t even went back in class. I left the books inside and my friend brought them after school, and that’s when I told her and she told me that she would have explained if I told her because she experiences that too.

I: And you told me that you told your grandmother than your mother?

R: I was staying with her

I: Not your parents?

R: Yes, I was cooking for her

I: Where was your mother this time?

R: She was also staying there but in a different house, and my grandmother was staying alone and I was cooking for her and when my grandmother moved out that’s when I went to stay with my mother.

I: They told you to go and stay with your granny so that you should take care of her?

R: Yes

I: How old were you?

R: 10

I: And you got the first period, you were also 10?

R: No, 11

I: But you started staying with your granny when you were 10?

R: Mmm

I: Okay, was there any advises that they gave you, or they organized another ceremony for you to be advised?

R: My grandmother advised me and mother too, and at school there are other Madams, where they call up all girls who have started having periods to give them same advice. For example, telling us that when we go to watch games we should not go home late to avoid us getting pregnant. They pick up older girls only; they do this as mother groups.

I: Alright, I wanted to know if in this village people go to initiations?

R: they do

I: Did you go or not?

R: I did

I: Before having the periods or not?

R: Before

I: How old were you when you went there?

R: 9

I: They don’t tell you that in future….

R: No, they didn’t but they just called those who were older; they said those who know are of age should come and my mother told me not to go and I didn’t go. I didn’t know what they were called for, they didn’t tell us what will happen in future.

I: You didn’t go to the initiation ceremony?

R: No, I went there, but the other group I didn’t go.

I: Oho, so you mean at the initiation ceremony there divide the group into 2?

R: We were together with the older ones, and they wanted the older ones to go and advise them separately, they chose the older ones to go somewhere and be advised.

I: So, for you who were younger, what did they advise you at the initiation ceremony?

R: They told us that when we go home we should not be rude, we should help with chores.

I: After you started having the period, you’ve never went back to get the other advice?

R: No, I haven’t gone yet, I forget what I was told there.

I: Alright, when you have a problem or you are worried whom do you feel free to tell or whom do you tell?

R: For example, if I have school needs I tell my mother, I cannot tell a friend that I lack such such things, because my friends they cannot buy those things but my mother can buy them for me.

I: So, what are the problems that you meet at a person?

R: Maybe quarrelling with friends, but not being ill treated by parents, no.

I: What are the problems that you meet?

R: Maybe at the borehole, quarrelling with friends to draw water and maybe someone wants to beat me and because I don’t know how to fight I just allow them to pass me and draw first. And also some men proposes to me and I say no, and they offer me money and I say no and would tell that to my mother.

I: When one is growing up, breast starts growing, and having hairs in some parts. Where did you hear such stories at first?

R: I have never heard of such.

I: That when girls are growing…

R: My mother told me

I: Where else?

R: Even my friends has never told me

I: Chatting with friends that this one has started growing breast?

R: Iiiii we do such, talking about girls like this one will not have breast if at this age doesn’t have, or when they noticed me having breast growing, they said my breast have started growing.

I: You are able to discuss that with your friends?

R: Yes

I: So, where do they say they come from?

R: We don’t ask such questions.

I: Or when your friend were saying you don’t have breast, and you see that your friend has, were you not worried that you will never have them?

R: I just thought that breast come from the chest, that’s all.

I: Were you not worried that yours were not coming out?

R: I was just watching that maybe because I am also a girl one day I will have them.

I: What about issues of HIV/AIDS, where did you heard about it first time?

R: At school

I: Was it in class or from friends?

R: My friends talks about that, in class too, even in Life skill they also teach that HIV is deadly and they also advise us that if you have sex you can contract AIDS, and sharing razors.

I: Can you remember the first day you heard about it what were you thinking?

R: I cannot remember

I: What is it that you would like to know about HIV/AIDS?

R: Yes

I: What is it that you would like to know?

R: I would like to know if one can contract it from sex and sharing of razors only?

I: That’s the question you always have?

R: And for me to ask at school, I don’t I feel like I will ask some day. I just feel like my question is stupid.

I: Alright, what about issues of pregnancy; I know you told me that when you had your first period you grandmother, and your mother told you about pregnancy. What are other things that you know about pregnancy and where did you get that information from?

R: At school, they said that if one has to avoid pregnancy, should use chishango when having sex.

I: Where else?

R: At youth

I: Alright, as I asked you about HIV, do you have things that you wish you know them about pregnancy?

R: About HIV and AIDS?

I: No, about pregnancy?

R: yes, I do

I: Yes, what is it?

R: If a person gets pregnant, what happens to her. People say if one gets pregnant, they become rude, I would like to know if the same issue of pregnancy makes her do that.

I: Alright, so issue about HIV or pregnancy, you would love to be discussing this with what person so that you can learn more?

R: The girls I chat with at home, the one who dropped out of school

I: You don’t discuss that?

R: We do, but I feel like she hides some information but maybe if I can push her hard maybe she can tell me. My mother told me that if I want to ask I should not fear her, I can ask her or other people, so I can ask my mother about other things.

I: Alright, in the community, there are sexual relationships between girls and boys, how common is this in your village?

R: Not very common because now there are few girls, all girls are finished.

I: What makes them to have such relationships?

R: Sometime because they lack money to body lotion, for example, if my mother is refusing to give me body lotion so where can I get lotion, that will mean I will have a relationship and it will end up having pregnancy or contracting diseases. But I will think that if I have the relationship then I will be able to buy body lotion.

I: Is there a place where you can discuss issues about relationships?

R: To me I don’t sit on a group where people are discussing about relationships, I don’t talk about that to show that I don’t like it, but I just get out of that place.

I: Why?

R: It is because I know they will talking benefits from such relationships like money and they will asking me if I take money when going to school and they will want me to do the same. So, I feel like I cannot be in such groups.

I: I have finished the question I had, do you have anything that you would like to tell me?

R: I would like to thank you for asking me such questions, come again and be able to ask a lot of girls not only me, in the youth groups there are other people, so that you can learn from them to.

I: Thank you for coming.

END
