# Supplementary material for: Social support and very young adolescent girl’s knowledge on sexual relationships: A comparative qualitative study of Girl Only Clubs’ participants and non-participants in rural Malawi
Source: PLOS Glob Public Health. 2023 Jan 12;3(1):e0001339. doi: 10.1371/journal.pgph.0001339 (PMC10022037; doi:10.1371/journal.pgph.0001339)
Supplement: S1 Data — (ZIP) [file pgph.0001339.s003.zip › Anonymized/IDI_ND_YY_2.docx]

**Location**: YY-NO Village

**Date**: DD/MM/2020

**Time**: 11:00 am

I: Tell me your age again. How old are you?

R: 13.

I: You are 13 years old?

R: Mmm.

I: Alright. Do you go to school?

R: No, I lack notebooks.

I: Come closer so that you can be heard. Did you say you go to school?

R: I lack notebooks.

I: You don’t go to school because you don’t have notebooks? Have you ever gone to school?

R: Mmm.

I: What class did you dropped out?

R: 3.

I: You dropped out in standard 3? Mmm. So did not go back because you lacked notebooks?

R: Mmm.

I: Alright. Tell me, who do you stay with?

R: I stay with my mother.

I: Your mother? How about your father?

R: He died.

I: When did he die?

R: Its long time ago, last month.

I: Your father died long time ago so you are staying with your mother?

R: Mmm.

I: Mmm. How many children you are in your family?

R: 4 children.

I: Who is the firstborn?

R: The first born is a boy.

I: The first born is a boy, then?

R: A girl.

I: Your elder sister?

R: Mmm.

I: And then?

R: A boy.

I: And then?

R: Me, a girl.

I: Mmm. So I want to know, do all your other siblings go to school?

R: No, one got married and also another one is married.

I: The two older girls?

R: The other is a boy. We are two girls and two boys.

I: Okay. So the first born son got married?

R: Mmm. The girl is also married.

I: She is also married? And then you?

R: Mmm, then the boy.

I: The boy is younger than you?

R: No, he is first I am the last.

I: You are the last born? How about the other boy?

R: He does not have uniform and also notebooks.

I: So he does not go to school?

R: Mmm.

I: How about the girl who got married, how far did he go with school?

R: Form 1

I: She dropped out in form 1?

R: Mmm

I: How about the older boy?

R: I don’t know.

I: You don’t know. That’s your brother?

R: Mmm.

I: So you call him elder brother?

R: Mmm.

I: Mmm. Does he have children?

R: Mmm.

I: How many?

R: 3 children.

I: Mmm. How about the other girl who is married?

R: She has 1 child

I: She has 1 child? Mmm. So when you are staying at home, how does your mother get food and other basics?

R: She does piece works.

I: She does piece works? Mmm. So from those piece works, can’t there come money to buy notebooks?

R: My mother is very difficult.

I: Your mother is very difficult? How does she respond when you tell her about notebooks?

R: She just yells at me or beats me.

I: When you tell her that you want to go to school? But you are willing to go to school?

R: Mmm.

I: But she is difficult and yells when you tell her about notebooks?

R: Yes.

I: Mmm, alright. So tell me what disappoints you when you are at home?

R: When I don’t go to farm, my mother doesn’t give me food.

I: Mmm.

R: And when I don’t do any chore she beats me or chases me away.

I: She chases you away?

R: [Silent]

I: Mmm. What else disappoints you?

R: My other sibling, the boy abuses/mistreats me.

I: Your sibling, the boy born before you?

R: Mmm.

I: What kind of abuses does he do to you?

R: When I don’t go to the garden to irrigate he tells me that I will not be given food.

I: What else does he abuses you with?

R: He tears my clothes?

I: He tears your clothes?

R: Mmm.

I: Why?

R: When I don’t go to the garden to irrigate.

I: Mmm, alright. How about what pleases you at your home?

R: What pleases me?

I: Mmm.

R: [silent]

I: When you are at your home or I should say, is there what makes you happy at your home?

R: Mmm.

I: What is it?

R: My mother sometimes loves sometimes she doesn’t love me.

R: Mmm.

I: Your mother sometimes loves you?

R: Mmm.

I: When she loves you, what does she do?

R: My heart is happy.

I: So can you give me examples that shows that she loves you at that time? What does she do when she loves you?

R: She gives me money to buy food for myself and sometimes she tells me not to do any work.

I: Mmm. Money to buy food, like where?

R: At the market.

I: Mmm. How much money does she give you for example?

R: 100 Kwacha.

I: Mmm, alright. So when you wake up to start your day, what do you do?

R: I draw water, clean dishes, i go to farm and then I cook.

I: Mmm, alright. How about where you like to chat when you finish doing house chores? Where do you chat? Do you have friends?

R: Yes.

I: Who is your best friend?

R: YYY.

I: YYY?

R: Mmm.

I: Mmm. Does she go to school?

R: Yes.

I: She still goes to school? What class is she?

R: She is in standard 4.

I: She is in standard 4? Mmm. So what you like to talk about with YYY?

R: We tell stories, we get along very well.

I: What stories do you talk about?

R: [Silent]

I: have you forgotten? Mmm then you don’t chat, you don’t have any story that you have talked about recently?

R: Now we quarreled.

I: Why did you quarrel?

R: Because she has a friend and people say that they wher friend went for an injection so she was saying ………that why get involved in the issue that does not concern us then her friend started name calling us.

I: Okay, you quarreled because she went for an injection?

R: Mmm.

I: So the one that went for an injection is your friend?

R: Mmm.

I: to get an injection for contraception?

R: Mmm.

I: So she was angry because…

R: She was saying that I betrayed her by telling my other friends.

I: Did she tell you that she is going for an injection? Does she have a boyfriend?

R: Yes.

I: Mmm, alright. How about you?

R: Me?

I: Mmm.

R: Injection?

I: Boyfriend.

R: I don’t have.

I: You are lying.

R: I am telling the truth.

I: You don’t have? How come your best friend has but you don’t have and to a point where she tells you about injection stories?

R: She took me to with her to an injection place and the doctor asked me if I get injections and I told him/her that I don’t but my friend.

I: How old is she?

R: She is 15 years old.

I: She is 15 years old? She still goes to school, what class is she?

R: She is in standard 5.

I: She is in standard 5? Mmm. So she heard that people know that she went for an injection?

R: Mmm.

I: So she said that you were the one telling them?

R: [silent].

I: Mmm, alright. Where do you like to go and chat?

R: At the video.

I: You like to go and watch video?

R: Mmm.

I: Really? Do you watch for free?

R: Yes, sometimes I for to sports.

I: Mmm. So places like these; at video and sports what challenges do girls face when the go to chat in such places?

R: For example at sports one sometimes gets hurt and sometimes quarrels with frienfds

I: Mmm.

R: Mmm.

I: Alright, have you ever faced a disappointment either at the sports or at the video?

R: Yes, I got hurt at the sports before.

I: Mmm, alright. How about at the video show?

R: I don’t face any difficulty.

I: Alright. So did I maybe asked you before about what disappoints you at home?

R: Yes.

I: I already asked you? How about what pleases you?

R: I want to stay with my elder sister but my mother doesn’t want me to.

I: Who doesn’t allow you to stay with your elder sister?

R: My mother.

I: Mmm. Why do you think she doesn’t allow you to?

R: I don’t know about that.

I: Tell me where you first heard about HID/AIDS? Where did you hear it first from?

R: Some people came at the market and were talking about it and I heard from there.

I: At the market?

R: Mmm.

I: Where else did you hear HIV stories from?

R: Nowhere.

I: Nowhere?

R: Mmm.

I: Alright, so when you heard for the first time what happened? What did they say?

R: They asked what happen for one not to contract AIDS. So a woman with 1 child went and explained that one goes to the hospital to get a blood test if one had AIDS or not.

I: Mmm.

R: Yes.

I: Alright. How about where you first heard about a person getting pregnant? Where did you learn or hear it from? And also who was the person that was speaking?

R: I have never heard about it.

I: You have never heard about it? Alright. How about relationships that exist between girls and boys like boy-girl relationships.

R: I hear they are in love and they talk.

I: Mmm. What else did you hear about boy-girl relationships?

R: [silent]

I: You have never heard?

R: Yes.

I: Don’t people, girls talk about relationships in this village?

R: Many girls don’t tell me.

I: Alright. I wanted to know if you have already started menstruation.

R: I have not started menstruation.

I: Alright. Is there someone who has told you in advance about what will happen?

R: Yes.

I: Who told you?

R: My elder sister told me.

I: Your elder sister?

R: Mmm.

I: What made her to tell you that?

R: I got sick that day and she told me that when I start to menstruate I will have stomachache and that I should tell her when I will start menstruating.

I: Mmm. How did you feel when she told you that? Did you feel shy, fear or hope?

R: I had fear.

I: Why did you have fear?

R: Because she said witches take one’s blood and takes it below oceans.

I: Mmm, alright. So when someone is growing, she develops breasts or hair in other places. Have you ever seen that?

R: No

I: Or maybe to talk about it with someone else that you will grow, breasts and hair will grow in the armpits?

R: Yes, people were talking.

I: Where?

R: At our place, that breasts starts slowly and when they grow they decrease and then increase and decrease, then the just grow.

I: Mmm. Okay. Tell me, the frequency of the relationships between girls and boys in your village?

R: Mmm maybe……..100.

I: And why to boys and girls have relationships?

R: I cannot know about that.

I: You cannot know why they do it?

R: Mmm.

I: Mmm, alright. Sometimes girls face different challenges when they are walking around the village maybe raping or beating them, how frequent do these happen in your village?

R: It is frequent…

I: Mmm?

R: It is frequent in ……….. 19.

I: Is there anyone you know that got raped?

R: Yes.

I: So what happened for her to get raped?

R: The man was escorting her and she went with him in the bush and he raped her and kids saw them and went and tell her mother.

I: So what happened? What happened to the man?

R: The man got arrested.

I: Mmm, alright. Has it ever happened to you?

R: No.

I: Mmm. How about boys proposing you?

R: They propose me but I refuse.

I: Mmm, alright. It seems you have finished all your questions that wanted to ask you.
