# Supplementary material for: Social support and very young adolescent girl’s knowledge on sexual relationships: A comparative qualitative study of Girl Only Clubs’ participants and non-participants in rural Malawi
Source: PLOS Glob Public Health. 2023 Jan 12;3(1):e0001339. doi: 10.1371/journal.pgph.0001339 (PMC10022037; doi:10.1371/journal.pgph.0001339)
Supplement: S1 Data — (ZIP) [file pgph.0001339.s003.zip › Anonymized/IDI_ND_YY_3.docx]

**Location**: YY-NO Village

**Date**: DD/MM/2020

**Time**: 2:15 pm

I: Can you remind me about your age, you said how old are you?

R: 11

I: Your 11 years old?

R: Yes.

I: Alright, in which class are you?

R: 3.

I: Where are you learning?

R: At CKE.

I: CKE?

R: CKE.

I: CKE?

R: Yes.

I: Alright, when you are in class and your learning, which subject do you like?

R: Mathematics.

I: You like mathematics? Why do you like mathematics?

R: Because there not difficult to study and mentioning of numbers.

I: You don’t find it difficulty?

R: Yes.

I: And it means you do well in class right?

R: Yes.

I: When you were going to standard 3, which number did you score?

R: I was number 11.

I: Wow, it means your intelligent right?

R: Yes.

I: This time which number are you going to get?

R: I want to be on position 1.

I: You want to be on position 1?

R: Yes.

I: Alright, what are the subject that you find them to be very difficult for you?

R: English and mathematics.

I: You find them difficult?

R: Yes.

I: Why do you find them to be difficulty?

R: Because I don’t know them very well and because am not very conversant with them.

I: Your not conversant with them?

R: Yes.

I: What makes you to be happy when your at school?

R: To be yourself and be studying.

I: What makes you happy is studying?

R: Yes.

I: Where do you stay?

R: I stay at T/As house

I: You stay at T/As house?

R: Yes.

I: In T/As house? Are you related with T/A?

R: Yes.

I: Who is he to you?

R: Our Granny.

I: Is your granny? Alright is your family, who do you stay with at your house?

R: My mother.

I: Who else?

R: Only my mother?

I: You only stay with your mother you don’t have your siblings?

R: I do have.

I: How many siblings do you have?

R: First one L, second LX, Third one ZX, fourth SP and CT.

I: SP?

R: Yes, and the last born is CT.

I: You said who is the first born?

R: No, the first one is L.

I: L, how old is she?

R: I don’t know her age.

I: Where is love? What does she do? Does she go to school?

R: Yes, she does.

I: In which class is love?

R: In 1 and she is repeating.

I: That’s the first born?

R: Yes.

I: So you’re a head of her your in standard 3?

R: Yes, because she was playing too much, while her friends are in class learning, she was busy outside playing.

I: Alright, who is second.

R: The second one is LX.

I: Yes.

R: The other one MU, CT and I am the last.

I: Where is L, does L goes to school?

R: Yes, at nursery school.

I: She is in nursery?

R: Yes.

I: Who else?

R: ZU and I already mentioned.

I: Nursery school?

R: Yes.

I: The other one ZX?

R: Yes, and I am the last born.

I: You’re the last born, so where is ZX?

R: She is a small baby.

I: So the other one is at nursery. So who is older between you and the one who is at nursery school?

R: The one who is at nursery is very young.

I: So in your house you’re the one who is in high class?

R: No, we have MX,

I: In which class is he?

R: 5.

I: You all girls?

R: Yes, with one man.

I: The young one?

R: Older.

I: LX?

R: No, he is a boy.

I: what’s is his name?

R: CT, the one I mentioned.

I: The one who is repeating standard 5?

R: Oooh the one who is repeating standard 5 is a girl.

I: Does CT go to school?

R: Yes he does.

I: In which class is he?

R: I don’t know which class because he stays far from here.

I: So you don’t stay with him?

R: Yes he stays where my father was staying.

I: Is your father alive?

R: No he is died.

I: He is dead?

R: Yes.

I: Ooho, so can you tell me about your day, how does it go when your home? What do you like to do when your home?

R: When I am happy I wash plates, swiping in the house, cleaning the surrounding area and bathing my young siblings.

I: After that what do you do?

R: I tell them lets go to play.

I: How about cooking? You don’t cook?

R: No, I don’t cook, I don’t know how to cook.

I: You don’t know how to cook? so where do you go to chat?

R: At SS.

I: At SS?

R: Yes.

I: Who do you chat with there?

R: There come visitors from children’s corner, we chat, we sing songs and dance with them and also we do storytelling and riddles.

I: When you are home, when you say you are happy, what makes you happy?

R: When am home, am happy because in my life I am happy and so other times am stressed.

I: What makes you happy in your life?

R: What makes me happy is that when I come from school and have washed plates, and after that I take a bath and go to sisi for chat.

I: Have you ever been happy at home?

R: Yes am happy.

I: You have been happy before?

R: Yes.

I: Can you tell me one thing you were happy with, tell me what happened for you to be happy?

R: It’s that am very happy in my life.

I: Yes, what happened?

R: What makes me happy is that I don’t insult my friends and chat with them properly without insulting them.

I: Yes?

R: I don’t insult my friends.

I: You don’t insult your friends?

R: Yes.

I: You don’t insult your friends that’s what makes you happy?

R: Yes.

I: Have you ever been depressed?

R: Yes.

I: Why were you depressed?

R: Because I think what my father was doing to me, right now I don’t see them and the money he was sending for me now I can’t see it.

I: What was your father doing for you?

R: He was sending money, when I don’t have shoes he was sending money so that I can buy shoes, when I say I don’t have clothes he was sending money so that I should by clothes.

I: When did he die?

R: He died not so long ago.

I: He died this year?

R: Yes this year.

I: What happened was he sick?

R: No, he took tameck.

I: He killed himself?

R: Yes.

I: Sorry, so do you go to church or mosque?

R: Mosque.

I: Do you have friends?

R: Yes I do.

I: How many friends do you have?

R: 5.

I: Among your friends do you have anyone who is your best friend?

R: Yes.

I: Who is she?

R: LY.

I: Why do you like LY?

R: Because I am happy with her.

I: Because your happy with her?

R: Yes.

I: Why are you happy with her?

R: Because she doesn’t insult me where ever she goes she takes me along.

I: There others who insult you?

R: Yes.

I: Friends who insult you?

R: Yes, when there going somewhere there send me back, you go back even if its short distance there send me back.

I: Send you back because of the way you are?

R: Yes, when its far there send me back, when its near there take me along.

I: When you have problems or you are stressed who do you tell?

R: I tell my mother.

I: Why do you tell your mother not telling your friends.

R: Because my friends cannot help me.

I: Who do you love most? Your mother or your friends?

R: My mother.

I: So here in your village, can you tell me where you like go?

R: Yes I have.

I: Where do you like to go?

R: At CA’s Place.

I: Why do you like to go at CA’s place?

R: Because I like the place.

I: You said you have 5 friends and you best friend is LY right?

R: Yes?

I: CA is one of the 5 of your friends?

R: Yes.

I: What makes you happy at CAs place?

R: because she doesn’t insult me we chat very well even today we were together at school, we also share food.

I: You are in the same class?

R: Yes, we are in the class.

I: With CA, How about LY?

R: We are also in the same class with LY, LY is repeating the class and I found her there.

I: Apart from CA place where else do you like to go?

R: At T/As house.

I: T/A?

R: Yes, because there we like doing some games like zawana, playing teaching each other things from school.

I: What other things do you do?

R: About the games?

I: Yes.

R: We call our friends saying that its children court, teaching each other there.

I: Alright, when you go where you have said you go like CAs place, at T/As house where you play. What kind of problems do you face when you go to play?

R: I meet problems like when we are doing zawana my friend discriminates me that this one should not be with me, others also says this one should not be with me and then I do stay by myself.

I: So when you meet such kind of problems whom do you tell?

R: I tell my mother?

I: What does she says?

R: She says just leave them, you should be chatting with your other friend you love.

I: What other problems do you meet?

R: At home?

I: No, where you go for playing?

R: My friends just beats me up and be stepping on me.

I: Alright, sometime the people In the village mostly girls of your age meets different kind of challenges like being raped, being proposed, does it happen here?

R: Yes, it happens.

I: Have you ever heard?

R: Yes. And it just happen not so long ago.

I: Where did it happen?

R: It happened there, it not that far.

I: A girl was raped?

R: Yes, a very young child that doesn’t even know to speak and of my age.

I: Yes.

R: She was asked” is there anyone here? With difficulties she answered, no there is no one. And then there went in a kitchen and start raping her.

I: There were no people who were passing by?

R: Yes, it happened on Sunday, she was told to go at her home and check it seems there were thieves. She didn’t know and starting calling people she was raped, and people went there for help.

I: Did they find the person?

R: Yes, was found him in the fence in kitchen.

I: And he was caught?

R: Yes, he was caught and taken to the chief, was arrested and was asked how much do you want to pay and he said two thousand he said when he will find the money he will pay.

I: Was the person who raped the girl from this village?

R: Yes.

I: They left him, he was just told to pay two thousand and left him?

R: Yes, he was just told to pay two thousand.

I: He was not arrested, he was not reported to the police?

R: Yes, was not reported to the police because they are related.

I: They are related?

R: Yes, that’s why there just thought he should pay.

I: Has it ever happened to you that someone wanted to rape you?

R: No, that has never happened since I was born.

I: How about men wanted to propose to you or doing anything to abuse you?

R: No it has never happened.

I: Have you ever heard about HIV?

R: Yes

I: Where did you hear it?

R: At the hospital when you go to get medicine, they do talk about HIV.

I: Yes.

R: There said if you are not taking your medication properly the virus increases in the body, and also if you take a year without taking any medication the virus will be increasing.

I: What do they say about HIV?

R: It’s a certain virus, when you don’t take medication in order and also you are not taking them on time that’s how that virus comes.

I: What else do you know? How does that virus come?

R: When you are not taking your medication that’s when the virus starts.

I: So that disease comes when you are not taking your medication properly?

R: Yes and when you are not taking the medication at right time, like you were told at the hospital and you are just taking them according to your head. Or either your taking them at 6 or 7 o’clock and you are not taking at required time then the virus increases.

I: That was said to the hospital?

R: Yes.

I: How about issues about reaching puberty? Have you ever held about it?

R: Yes.

I: Where did you held about it?

R: At the hospital and even here at home people do discuss.

I: What do they say at the hospital? When you go to take medication?

R: Yes.

I: Have you ever heard from the hospital?

R: Yes.

I: Can you tell me what happened?

R: They said a person should not get pregnant while young.

I: You said you heard from the hospital?

R: Yes.

I: So I want you to tell me what happened that day so that you should hear about it?

R: Because people were discussing it, and a nurse was explaining to the women.

I: What was she saying?

R: She was saying that people should not be getting married while there young and not before right age.

I: Did they said about starting menstruation?

R: Yes she did

I: What did she say?

R: They said a person should wait till you finish school.

I: But I am not hearing about attending menstruation, okay you said at home they do also discuss about it right?

R: Yes.

I: Who talks about it at home?

R: Women, or my mother telling her friends.

I: How about you being told, have you ever been told about it?

R: No.

I: Have you started having monthly periods?

R: No.

I: Do you have interest so that you know more about such issues?

R: Yes.

I: You want to know more?

R: Yes.

I: What do you want to know?

R: To me, I should work hard at school and I should not rush to get married.

I: Mmm.

R: And also when you get married fast you will fail to achieve what you wanted.

I: Do you know what period means?

R: No, I don’t know.

I: Have you ever heard about *kusamba*?

R: Mmm

I: What does it mean?

R: Bleeding.

I: That’s reaching puberty we are talking about.

R: Mmm

I: Have you ever held about it?

R: Yes.

I: Where?

R: Amongst my sisters there is one who is experiencing that.

I: Did she tell you?

R: No, she told her friends.

I: How did you hear about it?

R: When she is telling her friends, am always behind her. When she says lets go Linda for chat and the same time I listen.

I: But would you want to know what happens?

R: I would like to know.

I: Who do you want to hear from?

R: My young sister.

I: You’re younger sister to tell you?

R: Yes.

I: Do you think its important that younger girls you should be knowing before about issues like this?

R: Mmm

I: You think its good why?

R: Because a person cannot be surprised because you know it already.

I: Have you ever heard about it?

R: Yes.

I: Where did you hear about it?

R: During chief’s court and we go there and listen.

I: Alright, how about here in the village it happens sometimes that there issues about relationships, maybe a girl being in a relationship or a boy being in a relationship. Does it happen in this village?

R: Yes it happens.

I: How common is it?

R: If someone proposes to you and you accepts that and girls would want to get pregnant.

I: The girl?

R: Mmm

I: She wants pregnant, why would she want pregnancy?

R: Maybe because she is not happy with school or her parents are abusing her and then she just think of getting married.

I: Does it happen that parents abusing children?

R: Yes, it happen.

I: Maybe abusing them in what way?

R: As a child, you are told to go to the garden and work if they don’t they are told not to eat and one time I asked my mother that I went to the garden and she told me that after gardening did I sweep the compound, and I asked her if that was a reason enough to stop me from eating.

I: Yes.

R: Those are the problems there meet.

I: How about you, have you ever being proposed by a boy?

R: No.

I: Have you ever had a boyfriend?

R: No.

I: What are your opinions for a girl of a boy to be in a relationship as young as you are?

R: No it’s not good.

I: Why?

R: Because you don’t know so many things that’s why they say you should not be in a relationship.

I: Do you have anything that you want to say which I did not ask you?

R: Yes.

I: Yes, explain to me.

R: Why is it everywhere people make children in the family go into temptations?

I: Anything else you wanted to know?

R: Why are orphans abused?

I: When children are orphans there abused?

R: Yes.

I: So you want to know why orphans are abused?

R: Yes.

I: Anything that you would like to know?

R: That’s all.

I: I will answer you later, thanks for answering these questions.

R: Mm.

END
