# Supplementary material for: Social support and very young adolescent girl’s knowledge on sexual relationships: A comparative qualitative study of Girl Only Clubs’ participants and non-participants in rural Malawi
Source: PLOS Glob Public Health. 2023 Jan 12;3(1):e0001339. doi: 10.1371/journal.pgph.0001339 (PMC10022037; doi:10.1371/journal.pgph.0001339)
Supplement: S1 Data — (ZIP) [file pgph.0001339.s003.zip › Anonymized/IDI_ND_YY_4.docx]

**Location**: YY-NO Village

**Date**: DD/MM/2020

**Time**: 2:10 pm

I: How old are you?
R: 12

I: Do you go to school?

R: Yes

I: What class are you in?
R: Standard four

I: What are your favourite subjects?

R: Chichewa

I: Why is Chichewa your favourite subject?

R: I want to learn to read

I: What is your easiest subject?
R: Social

I: What about your hardest subject?

R: English and Maths

I: Why are they hard for you?

R: (Long pause)

I: How is it hard for you?

R: I don’t know how to read English and how to add numbers

I: What are you doing to get better grades in those subjects?
R: I ask my siblings to help me

I: And they help?

R: Yes

I: I want to know more about your home. Who do you live with?

R: My grandmother

I: What about your parents?

R: They passed away

I: Both of them?

R: Yes

I: When did you start living with your grandmother?

R: I used to live with my aunt but she was cruel to me so I went to live with my grandmother.

I: How old were you?

R: Six

I: What was your aunt doing to you?

R: I should never be late for any errands, if I make any small mistake she would hit me

I: Did you live with a lot of other children?

R: Yes, they had all boys and I was the only girl.

I: What jobs did they want you to do?

R: Cooking, looking for food which I did in the morning

I: Where were you getting the food?

R: In their garden

I: What type of food? Vegetables?
R: Yes

I: What was happening when you get back home?

R: They told me not to live the compound while their children roam free.

I: What about cooking?

R: They said I should be cooking

I: Nsima?
R: Yes

I: Relish?

R: Yes I was cooking the relish too

I: And their children?

R: Their children were just eating. I was told not to go out to play or even visit anyone including my grandmother.

I: Then your grandmother told you to live with her?

R: At the time my father hadn’t passed yet but my aunt refused me to go and visit him so I went to my grandmother’s house and my dad picked me up from there. I started living with him but he was sick and later on passed away.

I: When you were living with your dad?

R: Yes

I: How many children did your mother and father have?

R: I was an only child

I: It was only you?

R: Yes

I: Then your grandmother took you in?

R: Yes

I: This is your mother’s mother?

R: Yes

I: That is where you are living now?

R: Yes

I: Arlight

R: She also never share’s us flour unless we beg.

I: Who does that?

R: She has only bought me one shirt since I left her house.

I: Since you left her house and went to live with your grandmother?

R: Yes

I: That means your grandmother buys all your clothes?

R: Yes but she is also not well to do. I also receive clothes from the river banks?

I: Whose banks?

R: My father

I: Who lives there now?

R: My other grandparents and my dad’s brother?

I: So it’s your grandparents who buy you clothes?

R: Yes

I: With your dad’s brother?

R: Yes

I: You said that your grandmother who you live with is not well to do?

R: Yes, she is helped by her in-laws.

I: Who else do you live with apart from your grandmother?

R: A boy

I: You’re living with a boy?

R: Yes

I: Who is he?

R: CF

I: Who is he to you?

R: My brother

I: But you said you were an only child

R: His mother is mad and my mother took him in and breastfed him. His mother used to pinch him. She didn’t breastfeed him.

I: So she took him in when he was an infant?

R: Yes

I: Is he younger than you?

R: No, he is older?

I: Does he also go to school?

R: Yes

I: What class is he in?

R: Standard four

I: So you’re both in standard four?

R: Yes

I: Do you learn in the same classroom or separate classrooms?

R: Separate

I: You live with your grandmother and CF?

R: Yes

I: I just want to understand how you live. How do you share chores?

R: He doesn’t work and my grandmother can’t manage to fetch water.

I: What work do you do when your home?

R: I fetch water, I sweep, I wash the plates, I look for relish, I cook and then I go to school.

I: What do you do first when you wake up?

R: I fetch water

I: fetching water

R: And then I sweep

I: How much water do you fetch?

R: Two trips

I: That’s two buckets?

R: Yes

I: Then you take it home?

R: Yes

I: What will you use it for?

R: I use it to cook and wash plates

I: You use it to cook and wash plates, what about water for bathing before you go to school?

R: I go back to get more water

I: You go back to fetch water for bathing?

R: Yes

I: What about CF’s bath water?

R: He gets his own water

I: He gets his own water?

R: Yes

I: What about your grandmother’s bath water?

R: I fetch it when am back from school

I: That means your mother doesn’t bath until you’re back from school?

R: Yes

I: Your grandparent is a woman?

R: Yes

I: And she doesn’t work?

R: Yes

I: Why doesn’t she work, is she very old?

R: Yes and she also gets sick a lot

I: She gets sick a lot?

R: Yes

I: Who takes care of her when she is sick?

R: My uncle is there?

I: You live with him?

R: Yes

I: So you live with your grandmother, CF and your uncle?

R: Yes

I: So when you cook, you also cook for your uncle?

R: No, he has a wife

I: So you don’t live together just near each other?

R: Yes

I: You wake up in the morning, fetch water and you cook

R: Yes

I: What do you cook?

R: Relish

I: You cook it right then?

R: Yes

I: Then you bath and go to

R: School

I: What time do you start your classes?

R: We start late around eight or nine am.

I: When you knock off?

R: I go back home and cook

I: Now nsima?

R: Yes, we eat lunch and when I finish wash the plates. Then I go to the borehole to get water then prepare bath water for my grandmother.

I: Plates?

R: I wash them too

I: And when you’re done with that?

R: If there is no relish, I go and look for it.

I: And when your grandmother has bathed and relish is there, what do you do?

R: I go out to play but I don’t take long there?

I: Who do you play with?

R: MM

I: This friend of yours goes to school?

R: Yes

I: What class is she in?

R: Standard two

I: How old is she?

R: Ten years old

I: What do to talk about with MM?

R: We talk about working hard in school and what we want to be in the future

I: What type of games do you play?

R: We play ball

I: You play ball?

R: Yes

I: What other games do you play?

R: Jump rope and ‘ndado’

I: Do you only play with MM or you have another person you play with?

R: No, there is another person

I: Does she go to school?

R: Yes

I: Who is she?

R: Her name is EL

I: How old is she?

R: Twelve

I: What class is she in?

R: Standard three

I: Arlight. You said that you go to MM’s house and sometimes EL’s house. Apart from those places, where else do you play?

R: I don’t go out that much

I: These are the only places MM’s house and EL’s house?

R: Yes

I: Then you go back home in the evening?

R: Yes

I: Then you cook supper

R: Yes

I: Then you sleep

R: Yes

I: Do you go to Church or to the Mosque?

R: The Mosque

I: You go to the mosque?

R: Yes

I: On Friday

R: Yes

I: What worries you in the house of your grandmother?

R: What worries me?

I: Yes

R: Because of my Grandmother’s sickness she isn’t able to help me with my school.

I: What other problems do you face living with your grandmother?

R: Hunger

I: Could you talk more on that?

R: (Long pause)

I: Sometimes you don’t have food?

R: Yes

I: What other problems do you face?

R: Lack of clothes, school books, home appliances, plates and buckets

I: What are with living at your grandmother?

R: What am I happy with?

I: Yes

R: She tell me stories of the old times

I: Your grandmother?

R: Yes

I: Have you started having your period?
R: No

I: You haven’t started having your period

R: No

I: Have you ever heard about periods?

R: Yes

I: Who told you?

R: My grandmother

I: What happened for her to tell you?

R: I asked her?

I: You asked her?

R: Yes

I: What made you ask her?

R: What I heard about it, I thought it was a lie

I: I want you to explain to me what happened

R: I was with my friend while going home and she told me her stomach was hurting. When she got home and told her mother she just nodded and told her not to leave the house. Out of the blue, something started coming out. I asked my friend what it was and she didn’t answer. That is when her mother said she was having her period. That is when I asked my grandmother.

I: What did you ask your grandmother?

R: I asked her if people had periods. She told me “yes, people have periods.” Then I asked her how it happens and she told me I will know about it when I get older.

I: She didn’t explain what happens?

R: Yes

I: How did you feel after she gave you that answer?

R: I thought she was hiding something

I: You thought she was hiding something?

R: Yes

I: But you wanted to have known more on the subject?

R: Yes

I: They don’t talk about at school?

R: No, they don’t tell us about it

I: Have you ever been sent to an initiation ceremony?

R: No

I: You have never been sent to an initiation ceremony?

R: Yes

I: Where have you heard about HIV and AIDS?

R: Never heard about it

I: You have never heard about HIV and AIDS?

R: Yes

I: You don’t know what AIDS is?

R: Yes

I: Not even at school?

R: Yes

I: On the radio?

R: No

I: Even that people get tested for HIV at the hospital?

R: No

I: What about a person getting pregnant?

R: I have heard about it

I: Could you tell me what happened for you to hear about it?

R: A person stopped school and looking for a marriage. She had an affair with a man even though her mother told her not to. It became known that she was pregnant and some people told me about it.

I: Since it was the first time that you heard about people getting pregnant, weren’t you confuse with what it was.

R: I was and then I asked my aunt about it and she told me.

I: People were talking that she was pregnant?

R: Yes

I: And it was your first time to hear that?

R: Mmm

I: So, for you understand, what happened?

R: What?

I: Because it was your first time to hear that; when you heard that she is pregnant, were you not surprised?

R: I was and I asked my Aunt (younger sister to mother) what it means to be pregnant (*kuyembekezera ndikutani*). And she told me that one becomes pregnant and I asked that everyone has a stomach that God created, and she said no, this one is where the baby stays. And she asked me if I didn’t know about it, and I said yes, that’s when she said okay now I know.

I: So, where else did you hear that?

R: that was the only place.

I: So, do you have other questions to know more about this issue?

R: No, but one day she called me

I: Your Aunt?

R: Yes, she told me that I should work hard at school because if you rush to get married it will not help you , when a young girl have a baby while young they can die.

I: That’s what she told you?

R: Mmm

I: You didn’t ask why?

R: I did and she said because when you have a baby while young, your body is not mature yet.

I: Apart from you Aunt, is there any other place where you’ve heard of such issues?

R: No

I: If you were to choose whom you would want to discuss such issues with, whom would you chose?

R: My grandmother

I: Why your grandmother?

R: Because she knows more.

I: Alright. Most of the times girls encounter violence, sometimes being raped, sometimes boys proposing to them, how common is this in your village?

R: It is common

I: Can you tell me more?

R: Someone being raped while she doesn’t want, or being proposed to and impregnating her, destroying her life and the man run away, that is common.

I: It has happened to people you know?

R: Yes, a lot of people run to Mozambique.

I: Have you ever encountered any violence? Such as boys proposing to you?

R: No

I: Or threatening you?

R: No

I: When a girl is growing up, their breast starts growing and also growth of pubic hairs and starting period, have such things happen to you and you were also surprised how such things are happening?

R: Yes

I: So, did you ask someone about it?

R: No

I: Why were you not worried?

R: I just looked at it

I: There was no one who told you that it will happen that way? Would you have liked someone to tell you in advance that when you are growing up, things will be like this?

R: [pause]

I: Or you would want things to be like this and you should just see things when they come?

R: [laughs]

I: Alright, when you are worried, whom do you share them with?

R: There is no one I share with.

I: Why don’t you share?

R: I don’t want people to know

I: Why don’t you want people to know?

R: I just want to know them alone.

I: When you are alone?

R: Mmm

I: Okay, what is it that you like? Can you tell me when you were very happy. Or let us start with the time when you were sad, what happened?

R: When my father died.

I: What about the time you were happy?

R: When my mother bought me clothes.

I: Do you have anything to tell me?

R: [long pause]

I: Okay, am happy because you have answered the question, I do not have any more questions to ask you. Thank you.

END
