# Supplementary material for: Social support and very young adolescent girl’s knowledge on sexual relationships: A comparative qualitative study of Girl Only Clubs’ participants and non-participants in rural Malawi
Source: PLOS Glob Public Health. 2023 Jan 12;3(1):e0001339. doi: 10.1371/journal.pgph.0001339 (PMC10022037; doi:10.1371/journal.pgph.0001339)
Supplement: S1 Data — (ZIP) [file pgph.0001339.s003.zip › Anonymized/IDI_ND_YY_5.docx]

**Location**: YY-NO Village

**Date**: DD/MM/2020

**Time**: 11:20 am

I: What is your name again?

R: GM.

I: GM. In which class are you?

R: 8.

I: 8 have you just entered it recently or you have written?

R: I have written.

I: You have written 8 exams?

R: Yes.

I: Alright. Where do you learn?

R: NA school.

I: What?

R: NA

I: NA?

R: Mmm

I: School. Alright. Is the school nearby?

R: No it is very far.

I: It is far?

R: Yes.

I: From your house, how far it is?

R: Maybe 350 metres.

I: For you to leave here and get to home?

R: No, the home is a bit far.

I: From the school?

R: Mmm.

I: So every day you were walking. Were you travelling by foot?

R: Mmm.

I: Or you were taking a bicycle?

R: We were walking by foot.

I: Mmh.

How long have you learnt at that school?

R: For one year.

I: Ooh, the other time you were learning where?

R: At SA.

I: Where?

R: At SA school.

I: Ooh where is it?

R: It is down there close to [1:08]

I: Mmh.

R: Mmm.

I: That is near?

R: It is also far.

I: It is also far, so why did you stop there to go here?

R: There were some reasons right;

(interruption)

I: Like what?

R: Travelling was difficult.

I: Going there?

R: Yes, it was like very far while here was like a bit near so they told me that “its better you learn here.”

I: Mmh.

R: Mmm and also the issue of that we cross the river;

I: Mmh.

R: Yes, here also we cross the river but there it was like a bit difficult that I should be going to school.

I: Mmh, okay, alright.

Umm I wanted to know how the day is when it is morning, how you stay at home right?

R: Mmm.

I: Girls your age what do they like…….what do you do in a day when it is morning at home? To you for your day when you wake up, how is it?

R: I do household chores.

I: Mmh.

R: Mmm, then after that maybe we will go…. When we do the work and finish, we go and play.

I: Mmh.

R: Mmm.

I: I want you to explain to me that when I wake up, for example me like a woman, since I am a woman right?

R: Yes.

I: I have my own house, when I wake up my day starts with like when I wake I go and wash my face, brush my teeth after that I should go to the kitchen and see “what will we eat today?” I should organise, if there are potatoes I should wash them and put them on the fire then I should start thinking “what should I do? Ooh I should start mopping the house.” When I finish mopping the house, I happen that when I finish then I look around if there is laundry to wash, I wash, if there is not I now look for relish, meaning time is now getting close to 11/12 right?

R: Mmm.

I: After that I cook nsima we will eat, if there is no one to wash the plates for me then it means I will also wash the plates, dry them and pack them then it means the time is now close to 2/3, then I should chat with people then after that when we have finished chatting, maybe around 4/5 I should go and cook porridge for the children, then when I have fed the children, in the evening I cook nsima, then we sleep. So I want that…… that is my day right?

R: Yes.

I: So I want to hear how your day goes like.

R: Okay.

I: Most girls, when you explain to me about how your day is like, you should also tell me about most girls on their day what do they do?

R: Okay. For me in the morning when I wake up, I wash my face and brush my teeth then I go the farm and farm, when I come back from the farm I sweep around the household area. When I finish sweeping then I wash the plates, when I wash the plates I mop. When I mop I then go and draw water.

I: Mmh.

R: When I draw water then I wash the laundry. While washing like that it means the food is already on the fire for breakfast.

I: Who cooks?

R: It is parents who cook sometimes.

I: Mmh.

R: Yes, so when we do so, it means when we are done we go and bath, when we bath we eat breakfast. When we eat like that it means when it around past 11 then we organise cooking relish. When we cook relish we cook nsima. When we have cooked, we eat, after that we bath and go and play.

I: Mmh.

R: Mmm.

I: Okay, alright. Where do you go to play?

R: At the ball games, we like going there.

I: Okay, ball games for girls or boys?

R: For girls.

I: Okay. To watch or play?

R: To play

I: You also play ball?

R: Mmm.

I: Do you play for practice or it is like somewhere there is a competition to say this village and that village?

R: We do like practices, but when it is between one village and the other village we also play.

I: You also play?

R: Yes.

I: Mmh, alright, alright.

Most girls in this area,

R: Mmm

I: What do they do at the time that they are not doing household chores or they have not been asked to go on errands by parents, what do they do?

R: They go and study.

I: Mmh, most girls in your area they like to study, what else?

R: They like to go and play.

I: They go and play, play how?

R: Others doing like fish, fish (jump rope) yes.

I: Mmh, what else, other games? You are playing fish, fish, you are playing ball.

R: Yes, hopscotch.

I: Hop scotch.

R: Yes.

I: What other things do they do? Apart from playing?

R: Playing, others when they have finished work they just rest waiting “how will they day end?”

I: Mmh.

R: Mmm.

I: Okay, alright. Now at home, for girls like you at home what roles do they have? Roles?

R: That we should take care of our parents and also listen to our parents.

I: Mmh.

R: Yes, that is our role that we should listen to our parents when they are sending us on errands, we should be able to do the work and not refuse.

I: Mmh.

R: Yes.

I: Umm, the role of being submissive at home?

R: Mmm.

I: Mmh.

Which place in this area do girls like to go?

R: To play?

I: Mmh.

R: For example, like at school at CO.

I: There is place girls like to go there?

R: Yes, and play there.

I: Okay, where else? Another place?

R: Another place like at the hospital there.

I: At the hospital also?

R: Yes, others go.

I: What do they play there at the hospital?

R: Jumping maybe hopscotch, yeah.

I: But many girls also go there?

R: Yeah others also go there. Others also go in their schools like maybe in the trading.

I: In the trading like a market?

R: No, for example like a place where there is a good ground right?

I: Mmh.

R: A good place where people can play.

I: Ooh.

R: Mmm.

I: Alright. So these laces do you think they are safe places? Where the girls like to go and play.

R: Mmm.

I: They are safe places?

R: Yes, they are safe places?

I: Why do you think they are safe places?

R: Because there are also watchers to say if there happened an accident, then it means someone can what? Can carry the person or even us we can tell the watchers there.

I: There are people?

R: Yes, there are people.

I: Mmh.

R: Yes.

I: Can you tell me maybe another place where you’ve ever been where you went to play but when leaving there you left while feeling like “No I thought it was a safe place but it is not a safe place.” Is there a place where you’ve been and when leaving, feeling like “Iiih here I think it is not safe?”

R: No, at such a place I’ve never been there.

I: All the places where you go and play in you feel that they are safe?

R: Mmm.

I: Ooh. Do you have friends?

R: Yes.

I: How many friends do you have?

R: I have 4 friends.

I: Which one do you say “this is my best friend?”

R: Only one.

I: Who?

R: ZM.

I: ZM?

R: Yes.

I: Mmh, alright. What do you do when you are with ZM?

R: We take the books sometimes and be studying them, sometimes we…... when we’ve seen that we have studied, we go and play ZM.

I: Mmh.

R: Yeah.

I: You go together to play?

R: Mmm.

I: You have also said you study together?

R: Mmm.

I: What stories do you discuss with ZM?

R: We discuss that “my friend, when you finish school what do you want to be?” so she speaks and I also tell her. So we encourage each other to say “my friend we should work hard in school so that we should be like that woman we see our teacher.

I: Mmh.

R: Mmm, we tell each other that.

I: Yeah, any other stories which you discuss with ZM?

R: We discuss that for example I am at a puberty stage; this month was not good for me.

I: Mmh.

R: Yeah.

I: When you say the month was not good for you what do you mean?

R: On that it means that month was very difficult for me, maybe I was sick.

I: That you were on your period and you got sick because of it?

R: Yes.

I: Mmh. You are able to tell each other stories like those?

R: Yes.

Other stories that we are able to discuss are like maybe I am not seeing my body well.

I: Like you are sick?

R: Yes. Or like maybe I am scared that I am seeing my armpits differently, yeah, my body is changing.

I: Mmh.

R: Telling my friend.

I: Mmh. These stories you are only able to discuss them with ZM and not you other friends, No?

R: Yes.

I: Mmh, alright.

Are there any other stories that you discuss with ZM which you didn’t tell me?

R: No.

I: Mmh. Can you tell me of a time were you faced a certain problem and you wished to tell someone about the problem?

R: Mmm.

I: There is?

R: Yes, there is.

For example, like this other time I was asked being out on a date, so I wanted to tell my friend then I just said “Aahh No.” I did not tell her.

I: You also did not tell ZM?

R: Yes, I did not tell her.

I: Mmh. You also did not tell any other person?

R: On that, I told my father.

I: You told your father?

R: Mmm.

I: Mmh. Why did you tell your father? Why didn’t you tell ZM?

R: I told my father so that he should tell the person, yeah. And also that maybe ZM would fear to tell him, so I just went to tell him as my father.

I: Mmh.

R: Yeah.

I: Mmh, alright. You wanted your dad to go and talk to the person?

R: Mmm.

I: Did he tell him?

R: At that time, I was going away, I was going to like a holiday;

I: Mmh.

R: So I was going away.

I: So what did your father say to you?

R: He told me that “I will tell the person.”

I: Mmh.

R: Yes.

I: That’s all? You only told your father?

R: Yes, when I came back, at that time I had left ZM here right?

I: Mmh.

R: When I came back I explained and I explained it to ZM.

I: Mmh.

R: Yeah.

I: So what did she say?

R: She said “this is not good, its better you should tell your parents.” So I told her that I told them, then she said “you did good.”

I: Mmh.

R: Mmm.

I: Alright. You said you stay around where?

R: I stay around SE; there

I: Okay. You are staying with your parents?

R: Yes.

I: Biological parents?

R: Yes.

I: Both mother and father?

R: My mother stays on her own and my dad also stays on her own, they are divorced.

I: Mmh, so who do you stay with?

R: I stay with my mother.

I: You stay with your mother?

R: Mmm.

I: Okay, alright. I should go back a little, you said that story you told it to your father, why did you choose to tell it to your father?

R: I saw that he was the one who could help me because my mother doesn’t speak properly right?

I: Mmh.

R: Yeah.

I: When you say she doesn’t speak properly what do you mean?

R: That maybe that speaking is difficult for her, that to understand her speaking you wouldn’t understand each other clearly. But maybe if I was there I can be able to…

I: To explain properly?

R: Mmm, to explain properly but for someone else it cannot be possible.

I: Mmh. Okay, alright. How many children are in your fanily?

R: 1.

I: Only you?

R: Mmm.

I: Your mother got married again?

R: She did not get married again?

I: Your dad?

R: He got married.

I: Where he is there are no other children?

R: There are.

I: There are?

R: Mmm.

I: How many children are there?

R: 4.

I: Mmh, alright. But you are staying with your mother?

R: Yes.

I: Are you happy with staying where you are staying?

R: I am happy.

I: That you are staying there?

R: Mmm.

I: Mmh, alright. What excites you about staying there?

R: What excites me is that I am able to check on my mother;

I: Mmh.

R: Yeah, drawing water for her and also she struggles to go to the market;

I: Mmh.

R: Yeah, she doesn’t go to the market, I am able to go and buy relish.

I: Okay, alright. Staying with your mother, do you feel that you are safe?

R: Yes.

I: Properly safe?

R: Mmh.

I: Okay, alright. Can you tell me that maybe someday while staying there you thought that “iih my life was not safe?”

R: The time where my life was not safe;

I: Mmh.

R: There is none.

I: There is none?

R: Mmm.

I: All the time you see that you are safe?

R: Mmm.

I: Mmh. You have stayed with your mother all your life?

R: No.

I: Mmh.

R: At first I was staying with my same mother then my other mother took me;

I: Mmh.

R: Yes, and then….

I: A relative to your mum?

R: Yes, relative to my mum. Then I went to my father, there it was like I was just visiting right?

I: Ooh at your father’s you just go and visit?

R: Mmm.

I: Mmh, alright.

Do you feel that you are flexible to chat about your everyday life with people who you stay with?

R: Yes.

I: Like your mother.

R: I am able to.

I: You are flexible?

R: Yes.

I: Mmh, okay, alright. Can you tell me stories that you chat with your mother?

R: Yes.

I: What stories do you chat with her?

R: I chat with her stories…...

(Laughter)

I: What stories do you chat with her?

R: She is able to encourage me to say “my child what you are doing is not good, do this and this, it is what can help you. She is able to tell me that when I am chatting with her. Sometimes she tells me that our past was like this and this.”

I: Ooh she tells you about her past?

R: Yeah.

I: Mmh, what she explains to you saying “this what you are doing is not good,” what is it that you do?

R: It means I was… I was misbehaving (laughs)

I: You were misbehaving what? (laughs)

R: Or maybe I was not listening.

I: Mmh.

R: Yeah.

I: Okay, alright.

Have you ever heard of HIV and AIDs? Stories about HIV and AIDS?

R: Yes.

I: You have ever heard?

R: Yes.

I: At first, where did you hear it?

R: I heard it at first at school while we were learning right?

I: The first time ever when you heard about HIV where did you hear about it?

R: I heard it at the hospital.

I: At the hospital?

R: Mmm.

I: What did you go to do there?

R: To get tested.

I: Ooh! When you were leaving home you had already made up your mind that you are going for testing?

R: Yes. They came like at school right?

I: Ooh so you first heard it from school?

R: Yes.

I: The first time ever of hearing about HIV, you heard it from school?

R: Yes they were saying there is HIV and I said “okay.” So there came like health workers right?

I: Yeah.

R: On a certain day.

I: At school?

R: Yes, they came at school and started testing.

I: Mmh.

R: Mmm. Then I went and got tested.

I: They didn’t explain what HIV is?

R: The explained that HIV is a virus.

I: Mmh.

R: Yes.

I: What does it do?

R: HIV is a virus that is found in a person’s body.

I: Mmh.

R: Yes.

I: Ooh, alright. Okay.

So you’ve said you heard it from school right?

R: Mmm.

I: There came health workers?

R: Mmm.

I: And you’ve also said you went to the hospital for testing?

R: No, they came right there at school.

I: They were testing you at school?

R: Mmm.

I: They tested you?

R: Mmm.

I: Okay, alright. Did you ask them questions?

R: I was asking questions that “for a person, HIV how can you prevent it?”

I: Yeah;

R: So they were giving me ways.

I: What were they saying?

R: They were saying there are preventive ways; “if you want to have sexual intercourse, you should use a condom.”

I: Mmh.

R: Yeah.

I: Any other place where you heard apart from these health workers that came to school, where else did you hear about HIV and AIDS?

R: Like, right there at home right?

I: At home?

R: Yes.

I: Who?

R: My parents were talking saying that “outside here there is HIV my child, a disease.”

I: Who was telling you?

R: My mother she was like advising me right?

I: Ooh.

R: Saying “outside here there is HIV, it is a disease so you have to be careful.” So I said “alright.”

I: Mmh.

R: Mmm.

I: Do you think that girls your age are at a risk of contracting this disease of AIDS trough HIV?

R: Yes.

I: You think they are at a risk?

R: They are at a risk.

I: Why?

R: If they don’t protect themselves or abstain right, they can contract HIV.

I: Mmh.

R: Mmm.

I: Do you have questions in your heart about HIV/AIDS.

R: Mmm, I have them to say “this HIV if I happen to be found with it, what would I do?” I ask myself that “this HIV virus, how does it come?”

I: Mmh.

R: Yeah, I ask myself such questions.

I: Mmh, you think that whom would you like to explain to you more about HIV?

R: Health workers.

I: Health workers?

R: Yes

I: Why health workers to explain it to you?

R: They are the ones who knows more

I: they know more about what?

R: HIV

I: Have you ever heard about getting pregnant?

R: Getting pregnant?

I: Yes

R: I heard it before

I: For the first time, where did you hear it from?

R: My friend, who was telling me that so, so is pregnant

I: Did you have questions?

R: Yes

I: What were the questions?

R: How did she got pregnant and she told me that she doesn’t know.

I: Do you have question that you would like to know about pregnancy?

R: No

I: Where else did you hear about getting pregnant?

R: At school

I: What did the teacher say?

R: Said that one can get pregnant if she is troublesome and has a boyfriend, she can get unwanted pregnancy.

I: Which lesson was this?

R: Life skills

I: Do you wish to know more about getting pregnant?

R: Yes

I: Whom would you like to talk to about it and explain such things?

R: Or a teacher or anyone else

I: Name someone whom you would feel free to talk to?

R: A health worker

I: Why are you choosing a health worker?

R: That one can explain it very well, I cannot choose someone who doesn’t know the stuff.

I: Have you see some changes in your body, having hair growing on armpits?

R: Yes

I: or breast growing?

R: Yes

I: Have you started period?

R: Yes

I: When did you start?

R: 2019

I: Would you tell me what happened, I would like to hear the whole story what happened on that day you started?

R: On that day, I was just surprised that I had back pain and abdominal pains and I told my mother that am having those. And she just said okay, and I went to my mothers sister house where now the pain was more, and they asked me, and I also told them that I have had this pain for some time now. And I just saw the blood, and they told me that am now a grown up.

I: How did you see the blood?

R: I was getting up I saw my skirt stained with blood and knowing that I do not have a wound, I thought of telling her.

I: So, whom did you tell?

R: My mother’s sister because I was right at her house.

I: why did you choose to tell her?

R: Because it happened when I was at her place and I couldn’t go home because of distance.

I: Ohoo, so what did she tell you?

R: She told me that you are now grown up and that I need sanitary pads and that she should buy them for me, and she explained how to wear them.

I: What else did she explain?

R: She told me that I need to take care now, because if I start having boyfriends and having sex I can get pregnant.

I: Alright. Did you go anywhere to be given counselling or call some people to sit down with you?

R: Yes, they called my grandmother

I: What happened?

R: They told her that I have seen my first period and she told me that I should not indulge myself in sexual relationships with boys, I should stay as I used to stay, and I should abstain from sexual activities.

I: Apart from grandmother and aunt, ws there anyone who sat down with you to counsel you?

R: No

I: Do initiations happen here?

R: Yes, but I went there before I started my period.

I: Do they mention such things at the initiation ceremony?

R: Yes, they do

I: They explained to you?

R: Yes, but it was before ii started having period

I: Meaning that when you saw first period, you already had information about it, right?

R: Yes

I: So, what did they tell you about period at the initiation ceremony?

R: They said that ‘some of you girls when you start having period you start misbehaving, when you have sexual desires, when you meet a boy and he proposes to you, you get taken up, you need to take care, you are at risk.’

I: At what risk?

R: ‘that you can get pregnant if you are not careful, and if you are not abstaining.

I: When you saw that your body was changing, you are having breast and other things, did you have questions?

R: Yes, I asked my mother. I asked her that my breast are growing and she said ‘hey girl, you are growing you have started developing breast’.

I: right now, do you have question concerning your body or period?

R: I do have them. For example, I feel pain when I am having period and I want to ask why this is happening to me, if everyone feels that way.

I: Have you ever talked to someone?

R: Yes, I talked to my friend, and she told me that it happens.

I: Apart from her, have talked to anyone else?

R: I asked my mother’s elder sister. I told her that I feel abdominal pains and she told me that that’s how I am, it depends on how your body is.

I: Whom would you want to talk to about such issues?

R: Madam

I: You would want to talk to Madam to ask more?

R: Mmm

I: Right now, I would like us to discuss about boys and girls relationships.

R: [laughs]

I: Are you laughing. So, that mean it is not the first time to hear about those relationships?

R: Yes, I have heard about it from the teacher.

I: From the teacher?

R: Yes, was talking about boys and girls relationships.

I: Was it a lesson or was just counselling you?

R: Was a lesson.

I: Which lesson?

R: Lifeskills, and was saying ‘some of you rush into this, when you fee itching, that’s all, you are not there yet’ [laughing]

I: Was a female or male teacher?

R: Was a female

I: And that was the first time you heard someone talking about boys and girls relationships?

R: Yes

I: Okay. How common is it in this community to see girls and boys relationships?

R: It is not very common. Because most of the girls who usually do that are those are the ones who finished their school.

I: But for the girls of your age?

R: Not very common

I: Okay, so, for those who does that, when they are doing it, what are the reasons that they do it?

R: Some because of problems/poverty but some because of being troublesome or misbehaviours just wanting to be in a relationship.

I: Have you ever discussed issues of sexual relationships with your parents?

R: No

I: You’ve never discussed it before?

R: yes

I: Alright. But with your friends?

R: Yes, she told me that people are having relationships and we should not rush into it, and I said alright.

I: What else did you discuss?

R: We also discussed that one can get pregnant and encounter problems at the hospital.

I: Alright, what do you want to hear concerning boy and girl relationships?

R: Nothing

I: whom would you love to discuss with on issues of boys and girls relationships?

R: With a nurse

I: Why have chosen a nurse?

R: Because is the one we can talk to each other freely and answer each other questions freely, ‘I cannot speak to a man’

I: [laughs] okay, is there something that you would like to tell me?

R: No

I: Do you have any problem that you have encountered in your life that you would like to share?

R: The problem can be there

I: Mmmh, what kind of a problem?

R: Being proposed to

I: Okay, so what did you do when he proposed?

R: I was sad

I: What did he say?

R: I was in the farm, and he came and told me that he wants me to be his lover, and I said I don’t want I am still young.

I: How old was the boy?

R: Maybe a year older than me.

I: So, how did you handle it?

R: I told him that I don’t want, and I also told my mother who went to his home and told his mother that she doesn’t want that behaviour with me because I am young, and if the boy feels he is grown up he should go look somewhere.

I: So, your mother went to the boy’s house?

R: Yes, to tell them to look elsewhere because I am young.

I: Alright, thank you very much for your time.

END
