# Supplementary material for: Social support and very young adolescent girl’s knowledge on sexual relationships: A comparative qualitative study of Girl Only Clubs’ participants and non-participants in rural Malawi
Source: PLOS Glob Public Health. 2023 Jan 12;3(1):e0001339. doi: 10.1371/journal.pgph.0001339 (PMC10022037; doi:10.1371/journal.pgph.0001339)
Supplement: S1 Data — (ZIP) [file pgph.0001339.s003.zip › Anonymized/IDI_ND_YY_6.docx]

**Location**: YY-NO Village

**Date**: DD/MM/2020

**Time**: 3:00p m

I: What’s your name?

R: EM

I: How old are you?

R: 10

I: In which class are you?

R: 3

I: Where do you go to school?

R: At KU

I: Mmmh, how many children are you in your family?

R: 3

I: Three children?

R: Yes

I: Whom are you staying with?

R: My mother

I: Your father too?

R: Yes

I: Both biological parents?

R: Yes

I: Who is the first born?

R: PA

I: In which class is PA?

R: 5

I: The 2^nd^ one?

R: Myself

I: The 3^rd^ one?

R: VA

I: How old is VA?

R: 7

I: Does she go to school?

R: Yes

I: In which class is she?

R: 2

I: What do you do at home when you wake up in the morning?

R: I sweep the house and wash plates.

I: Oho, do you know how to farm?

R: yes

I: Mmm, okay. Do you go to school?

R: Yes

I: How many teachers to do you have in your class?

R: 4

I: Which teacher do you like most?

R: ST

I: Why do you like that teacher more?

R: She teaches well

I: What else?

R: Doesn’t like to beat up children in class.

I: Alright. What is it that you like at home?

R: [pause]

I: When you are home, what do you like most?

R: Sweeping

I: In your life what do you like at home?

R: My mother doesn’t get angry at me most of the times and doesn’t insult me most of the times.

I: Who insults you or gets angry at you at home?

R: No one

I: No one?

R: Yes

I: Mmm. At school, what it that you don’t like?

R: My friends insults me

I: Why?

R: Because I don’t share them my food

I: What else?

R: Nothing

I: What about home, what is it that you don’t like at home?

R: To be scolded

I: To be scolded by who?

R: My parents

I: Why do they scold you?

R: [pause]

I: Mostly, who scolds you?

R: My mother

I: Why does she scold you?

R: Because sometimes I don’t listen to her

I: Okay, so, whom do you talk to when you want to?

R: ED

I: Who is ED?

R: My friend

I: In which class is ED?

R: Standard 3

I: Are you provided with food at home, properly?

R: Mmm

I: When you are wrong in somethings, what does your parents do to you?

R: They just scold me

I: They don’t starve you?

R: No

I: Okay. Whom do you tell when they scold you?

R: My grandmother

I: What kind of issues do you tell grandmother?

R: She says I should not be disrespectful.

I: Have you gone to initiation ceremony?

R: No

I: What does your mother say about initiation?

R: At our place we don’t do initiations.

I: Okay. Do you go to church?

R: Yes

I: Where?

R: HT

I: You go together with your mother?

R: Yes

I: Have you ever heard about HIV/AIDS?

R: yes

I: Where?

R: At school

I: Were you learning?

R: Mmm

I: What lesson?

R: Luso

I: What did the teacher say about HIV?

R: That we should not indulge ourselves in sexual activities

I: Have your parents talked to you about HIV?

R: No

I: Mom has never said anything about it?

R: No

I: What about dad, and grandmother?

R: No

I: Okay, do you have questions about HIV/AIDS?

R: I would like to know, but my friends don’t tell me.

I: Have you ever asked your friends?

R: Mmm

I: What do they say?

R: They say that they don’t know.

I: Whom do you want to talk to on issues concerning HIV/AIDS?

R: My friends

I: Why your friends?

R: Because they keep my secrets

I: Does ED keep secrets for you?

R: Yes

I: What secret did you tell ED that she kept?

R: [long pause]

I: I am not going to reveal your secrets

R: [long pause]

I: Have you forgotten?

R: Mmm

I: Have you ever heard about relationships of boys and girls?

R: I have heard about it but I don’t do that

I: Where have you heard it from?

R: My friends

I: Oho, what were they saying about boys and girls relationships?

R: They told me but I forgot it

I: If you were to know more about girls and boys relationships, whom would you love to know it from?

R: My friends

I: Alright. Have you started having periods?

R: No

I: Has anyone tried to scare you?

R: No

I: Where do you go to play?

R: At home

I: Where else?

R: At the market

I: Are you not afraid that something can happen to you at the market?

R: I do

I: What do you fear?

R: People to harm me

I: Have you ever heard someone was hurt there?

R: Yes

I: What did you hear?

R: I heard that a certain girl was raped

I: A young girl

R: Yes

I: Where else do you go to play?

R: Home

I: Does your mother and father give you necessary things for school?

R: Yes

I: Like what?

R: Food

I: What else?

R: Money

I: What else?

R: Notebooks

I: Okay. What subject do you love?

R: Mathematics

I: Okay. When you grow up, who would you like to be?

R: I would like to be a nurse

I: Why a nurse? where you are staying, is there a nurse?

R: Yes

I: What’s her name?

R: MN

I: She is a nurse?

R: Mmm

I: So, you admire her?

R: yes

I: Apart from mathematics, what else do you like?

R: Chichewa

I: On what position were you last time?

R: 15

I: How many are you in your class?

R: More I cant count.

I: Oho. Thank you very much that you came, right?

R: Mmm

END
