# Supplementary material for: Social support and very young adolescent girl’s knowledge on sexual relationships: A comparative qualitative study of Girl Only Clubs’ participants and non-participants in rural Malawi
Source: PLOS Glob Public Health. 2023 Jan 12;3(1):e0001339. doi: 10.1371/journal.pgph.0001339 (PMC10022037; doi:10.1371/journal.pgph.0001339)
Supplement: S1 Data — (ZIP) [file pgph.0001339.s003.zip › Anonymized/IDI_ND_YY_7.docx]

**Location**: YY-NO Village

**Date**: DD/MM/2020

**Time**: 12:45pm

I: What is your name?

R: EP

I: Okay. Are you still in school?

R: Yes

I: Where?

R: B CDSS

I: In which class are you?

R: Form 1

I: Alright. Where are you staying?

R: NTK

I: How many are you in your family?

R: 5

I: Who is the first born?

R: Myself. There is CP, BP, CB

I: How many children?

R: three

I: In which class is the last born?

R: 3

I: Alright. How old are you?

R: 14

I: For a normal girl, how does her day goes by?

R: As a girl, our day goes by well

I: What do you do?

R: We work, we help our parents household chores and when we do that, then we have time to play.

I: Where do you go to play?

R: At the ground to play

I: What kind of games?

R: Netball

I: where else do you go?

R: At the market

I: Where else?

R: At the river

I: In all the places where girls go to place, do you think they are protected areas?

R: Looks to be protected

I: You feel like there is risk in all those places?

R: The river is not protected, they tell us to take a bath carefully that we should not be bitten by wild animals.

I: Apart from wild animals what else is dangerous there?

R: There is; there are other places where it is very deep, and they tell to be careful or we can die there.

I: What else

R: They tell us that we should not be fighting each other because we can harm each other.

I: Alright. What responsibility does girls have at home?

R: We are just told what to do by parents at home. To wash plates, sweep house surroundings.

I: Have you ever gone somewhere to play and felt that, you didn’t have protection?

R: Yes

I: Where?

R: At the market place

I: What happened?

R: One day I went to the market late afternoon and I met someone who was proposing to me and I said no.

I: What time was that?

R: It was late in the afternoon.

I: What really happened?

R: I just met him at the market and he made me to stop and started proposing to me, I said no, but he continued pestering me, of which I thought that there was no protection there.

I: Was he touching you?

R: No

I: Does it happen that girls are touched by boys in private parts?

R: Yes it happens

I: Usually where?

R: At school and also at home.

I: Where do boys from home come from?

R: Neighbors

I: At school which people do this?

R: Fellow students

I: Whom do you like chatting with?

R: My friends

I: Mmmh

R: Should I mentioned them?

I: Yes, who is your best friend?

R: ES

I: I would like to know what issues do you chat with ES?

R: We talk about girls life

I: Like what?

R: {laughs} Like issues of period, we advise each other on how to prepare for it so that it doesn’t catch unexpectedly.

I: What else?

R: School issues, how we can work hard

I: All the time do you discuss period and school issues? What else do you discuss?

R: [laughing] we also discuss issues of relationships with boys, like for her, she had had a boyfriend before, and we discuss such things.

I: What kind of issues of boy and girl relationships?

R: She once had a boyfriend, and she was telling me that there are problems in relationships. And I asked her what problems, she told me that relationship are troublesome I should not try it.

I: What kind of problems?

R: She said that people hurt each other

I: Like doing what?

R: Having sex

I: What kind of problems did ES meet?

R: She had sex with her boyfriend and also the boyfriend used to beat her whenever they agreed to meet and they don’t, next time they met he would beat her.

I: In which class is ES?

R: Form 1 also

I: How old is she?

R: I think 16

I: Alright. Was there any day that you encounter problems in your life and you wanted to tell someone about it?

R: No.

I: You said you are staying with both parents, right?

R: Mmm

I: Is there any problem that you meet at home?

R: No

I: Problems from your friends, siblings, father and mother?

R: No

I: You are laughing. What is it that you don’t like at home?

R: Nothing

I: Or what is it that you like at home?

R: They buy us clothes, give us enough food

I: Alright. Was there anytime that you felt unsafe at home?

R: It happens. Sometimes they can tell me to stay behind at home and do some work while the other children can go and play and I feel like they are favoring other

I: Have you stayed with your parents all your life?

R: Mmm

I: You’ve never stayed with someone else?

R: At some point I moved and I was staying with my uncle.

I: How were you staying there?

R: I was suffering

I: What was happening?

R: They were not giving me food, not buying clothes for me.

I: Where was that?

R: NGW

I: right here?

R: It was a bit far

I: What happened for you start staying with your uncle?

R: He just came to ask me to go and stay with him

I: Were you just staying with the uncle only?

R: At first he was not married and I was cooking for him and I started being mistreated when he got married.

I: The uncle was from which side?

R: My mothers’ brother.

I: Can you tell me what you know about HIV/AIDS? What do you know about HIV/AIDS?

R: I know that AIDS is dangerous

I: Where did you hear about HIV/AIDS first time in your life?

R: At school during Life Skills

I: So, what did the teacher say?

R: Said AIDS is dangerous and people contract it when they have unprotected sex.

I: Did you have questions at that time?

R: Yes

I: Did ask them?

R: Yes

I: What were the questions?

R: How does it look like? And the teacher said that it looks like a life cycle that circulates in the body and destroys a human body.

I: Where else have you heard about HIV/AIDS?

R: Home, from neighbors

I: What were they saying about it?

R: They were saying that AIDS is dangerous.

I: That’s all

R: Mmm

I: What transpired for them to start talking about HIV/AIDS?

R: They were chatting.

I: Alright. Do you think girls of your age are at risk of contracting HIV/AIDS?

R: Yes

I: Why?

R: Because some girls of my age are having unprotected sex and they can also share sharp objects like razors.

I: Do you have question that you would like to know more about HIV?

R: Yes

I: Whom would you love to talk to about them?

R: My friends

I: Alright. What have you heard about pregnancy? And the first time you heard about it, how did you feel?

R: I heard that pregnancy is dangerous.

I: Where did you hear it from?

R: Parents, they were discussing it with friends.

I: What were they saying?

R: They were saying; getting pregnant is dangerous.

I: What is it that they were discussing?

R: [long pause]

I: Am not going to tell them

R: They said that we need to protect ourselves when having sex

I: Okay. Did you have questions?

R: I just heard them discussing and I didn’t want to get involved

I: Where else did you hear about pregnancy?

R: At school

I: Was it a lesson?

R: No, they were just talking to us; counselling us

I: Did you have questions?

R: No.

I: With ES, have you ever discussed issues concerning pregnancy?

R: Yes, we discussed that we should not have boyfriends since if we do that, accidentally people have sex and get pregnant.

I: The questions that you have, whom would you like to discuss it with you and tell you all that you want to hear about pregnancy?

R: Parents

I: Mother or father?

R: Mother

I: Why?

R: Because she is the one who carries pregnancy

I: So, you think she might know more?

R: Yes, she might know more.

I: Have you started doing your periods?

R: Yes

I: When?

R: Last year

I: What happened?

R: In morning when I woke up I saw blood on my petticoat, and I remembered what we discussed with friends if I ever see such a thing. And I did as I was instructed.

I: By the time you came to see this, you already have heard about it, right?

R: Yes

I: Who told you?

R: My friend and also at school.

I: At school, were you learning?

R: No, mother group women came to talk to us.

I: And when you saw that, whom did you tell that?

R: My parents

I: Mother or father?

R: The mother

I: And what did she say?

R: She just said that ‘you are now an adult, and you should not have boyfriends, because if you do that can get unwanted pregnant.

I: Who else talked to you after this happened, was it just your mother?

R: No, my grandmother.

I: What did she say.

R: She called me and counselled me.

I: What did grandmother say?

R: She also talked about boyfriend issues, that I should not have boyfriends

I: Who else counselled you?

R: My friends

I: What did they say?

R: [laughed] They said that when you start having period and you are having boyfriends you can get pregnant.

I: Do you have initiation ceremonies here?

R: Yes

I: Do you go there?

R: Yes

I: So, when you start having period they call for initiators to come and talk to you?

R: No, the counselling is done right at the initiation ceremony.

I: So, did you go to initiation ceremony after starting period or before period?

R: Before period

I: Did they explain about period at that time?

R: Yes

I: So, how did you feel when you saw the blood?

R: [laughs]

I: Were you afraid?

R: No

I: How did you feel? Were you happy to see that?

R: No

I: Why?

R: [laughs] they came early, but I just had to accept it.

I: When you saw that were you afraid although you already heard about it?

R: I was afraid and I went to tell my friend and they told me that it was one part of becoming of age.

I: Do you have any question concerning starting period or your body changing?

R: Yes

I: Whom would you want to discuss such issues?

R: My friends

I: Why?

R: You cannot be telling parents everything [laughing]

I: Have you ever told them something and they told you that?

R: Yes, they said ‘that should not be discussed here’

I: Parents? What do they say?

R: They say that ‘ such things are not supposed to be discussed here, you should discuss them elsewhere’

I: Okay. Let us now discuss about issues of boy and girl relationships. Do you have a boyfriend?

R: [laughs] no, I don’t have

I: You have, right? Laughing like that?

R: [laughs] no

I: Alright. When was the first time you heard about boy and girl relationship?

R: At the initiation ceremony

I: Where else?

R: At school too

I: During classes?

R: Yes were learning life skills.

I: Alright. What was it that you were being told at the initiation ceremony concerning boy and girl relationships?

R: they said that when you go home; ‘we are just counselling you, it is not that you should indulge yourselves girls and boys relationships’

I: Alright. What did exactly did they tell you at the initiation ceremony?

R: They told us that when we go home we need to find someone ‘kusasa fumbi’ since we are now grown ups.

I: How old were you when you went to initiation?

R: I was 9 years old

I: In this community, how common are these boy and girl relationships?

R: Nowadays it is very common

I: Why do young people involve themselves in this?

R: They say it is because of poverty, and they get benefits.

I: What kind of benefits?

R: Money

I: Was there time when parents had to talk to you about boy and girls relationships?

R: Yes

I: What is it that they discussed with you?

R: They just said we should not have boyfriends, such issues are not allowed.

I: You said that you discussed this with your friends?

R: [laughs] yes

I: What were you discussing about boyfriends?

R: We were not discussing it but she was just telling me

I: What is it that girls discuss about relationships?

R: They say they are given money and enough love.

I: Oho, what else?

R: That’s all.

I: Alright. Are there things that you would like to know about relationships?

R: No

I: Is there anything that you would like to know about relationships, or anything that you feel we didn’t talk, or problems that you meet that you would want to share. Are there problems that you have met that you would like to share?

R: Yes

I: What kind of issues?

R: There are times you meet a boy and is proposing to you and you said no, and your friends might be saying why did you say no, and they will tell you that you didn’t think properly to say no.

I: At school, what kind of problems do you meet?

R: Teacher give us punishments and sometimes same teacher would also propose to you.

I: At home, what kind of problems do you meet? At home we live with a lot of people, right?

R: Mmm

I: What problems do you meet there?

R: Nothing.

I: Thank you very much for your time.

END
