# Supplementary material for: Social support and very young adolescent girl’s knowledge on sexual relationships: A comparative qualitative study of Girl Only Clubs’ participants and non-participants in rural Malawi
Source: PLOS Glob Public Health. 2023 Jan 12;3(1):e0001339. doi: 10.1371/journal.pgph.0001339 (PMC10022037; doi:10.1371/journal.pgph.0001339)
Supplement: S1 Data — (ZIP) [file pgph.0001339.s003.zip › Anonymized/IDI_ND_YY_8.docx]

**Location**: YY-NO Village

**Date**: DD/MM/2020

**Time**: 9:15 am

I: How old are you?

R: 11 years old

I: In what class are you?

R: Standard three

I: Including yourself, how many children there in your home?

R: Five

I: Who is the first born?

R: Me

I: You’re the first born?

R: Yes

I: Do all of you live with your mother?

R: We used to be only two children. Then our father broke up with our mother and married another woman. Then we stayed with our father.

I: So you live with your father now?

R: Yes, but the woman had her own two children and now another has been born to make it five children.

I: Where does your mother live?

R: Balaka

I: Did she get married again?

R: Yes

I: Does she have children?

R: Yes, she has one.

I: What is your daily routine from morning?

R: I wash the plates and then I go to draw water. I mop the house and prepare water to bath. We cook breakfast and then we go to the market and sell

I: What do you sell?

R: *Thobwa*

I: You sell *thobwa*?

R: Yes

I: How do you sell thobwa during school period?

R: I take turns with my younger sibling who is in standard two. If she goes to sell today, then I go to sell the next day.

I: How many of you go to sell thobwa?

R: My three siblings and I

I: Three?

R: Yes

I: So it is four people?

R: Yes

I: How many of you are sent to sell thobwa in a day?

R: One goes to school and another to the hospital

I: So everyone goes to sell alone?

R: Yes

I: Do you feel that you are well protected at the place you sell thobwa?

R: Yes

I: When you are selling do you feel safe or do you sometimes feel that someone could hurt you?

R: I’m not afraid

I: You’re not afraid?

R: Yes

I: Nothing concerning has ever happened at the places you sell thobwa?

R: Yes

I: It very safe?

R: Yes

I: Nobody has ever harassed you there?

R: Yes

I: Or touched you on a bad place?

R: Yes

I: Or talked to you in a bad way?

R: Yes

I: Nobody has ever done that?

R: Yes

I: When you aren’t selling thobwa, what do you do? When you aren’t selling thobwa or doing household chores.

R: They tell to stay home or go to play

I: Where do you go to play?

R: Either at my friends place or at the ground where we play ball

I: Is the ground safe and free of boys who might bully you?

R: Yes, the ground has girls only. The boys have their own ground where they play ball.

I: When you are at the ground, you don’t come across any bullying?

R: Yes, but some people swear at each other even though their friends advise them no to. For example; just yesterday, some girls were swearing at each other. Then another girl tried to end the argument but the girls who were arguing accused her of barging into other people’s business and threatened to beat her if she continued.

I: What did they get into an argument about?

R: They were arguing about who gets to draw water first because one found the other. Then they started throwing each other’s bucket in the forest.

I: What don’t you like and feel like it needs to be changed in your life? Is there anything?

R: Yes

I: Like what?

R: To be honest, I am a very peaceful but I do not understand why my siblings despise me. I haven’t wronged them in any way but I don’t know why they despise me. Sometimes I ask them why they do that because family loves one another and doesn’t despise each other but they say that it doesn’t matter.

I: Are these siblings the children of your biological mother or stepmother?

R: The children of my stepmother

I: Who is older? You or them?

R: I am, that is why I just leave them to do whatever they want. When they are done, they will stop

I: Is there anything else that disappoints you?

R: No

I: What disappoints you at school?

R: Some people make noise in class deliberately to annoy the teachers so that they dismiss the class. I wonder why they do this instead of just chasing out the noise makers so the others can continue to learn. When some don’t want to marked, they make noise on the class so the teacher gets angry and refuses to mark anyone’s book.

I: Could you now tell me the things you like at home?

R: They don’t stop me from playing. They relieve me of cooking after I play.

I: You’re not the only one who cooks?

R: We assign the work amongst ourselves. Some cook in the morning and the rest in the evening.

I: What are the things you like at school?

R: I am never short of friends to chat with. When one brings food, we all share and eat together. We also go home together and when we eat at home, we go to play.

I: When you are having a problem at home, who do you go to?

R: I go to my mother and tell her that I am sick and having pains either in the head or the stomach

I: Who do you go to when you have a problem at school?

R: The teacher

I: When you go to your mother for help, does she help you?

R: Yes, because she is a doctor

I: What other problems do you tell your mother about apart from sickness?

R: When someone has harassed me or hurt me, I tell my mother

I: You are not afraid of her?

R: Yes

I: Why don’t you tell the father?

R: He is at the school for most of the day and comes back home late. Therefore, my mother tells him about it when he comes back.

I: You aren’t afraid of your father?

R: No

I: You aren’t afraid?

R: I am

I: You are afraid of him?

R: Yes

I: Why is that?

R: He is a difficult man

I: You said that you play with your friends at the ground right?

R: Yes

I: Who is your best friend?

R: EL

I: What type of things do you talk about with EL?

R: We encourage each other to study and work hard so that we pass our examinations. Our mother also told us both to pass our exams. She said that it shouldn’t just be one of us but both. My friend didn’t use to know how to read and I had to teach her. The only thing that is hard for us to read now is English.

I: What were you doing to improve your English reading?

R: I practice with my siblings and when we mispronounce something, our mother corrects us. Or we go to part time classes.

I: Have you had any problems with the parents you are living with?

R: No

I: Have you ever felt unsafe or threatened while living in the house? Is there anyone who makes you feel unsafe or threatened?

R: No

I: Have you ever heard of HIV?

R: Yes

I: Where did you hear about it?

R: I heard about it at school, a teacher taught about it

I: What subject was that?

R: (Long pause)

I: What subject was it?

R: They said that… (Long pause)

I: They taught about HIV and AIDS?

R: Yes

I: Do you want to know more about HIV and AIDS?

R: Yes

I: Did you ask the teacher any questions about it?

R: No I didn’t

I: Who do you want to learn about it from?

R: The teacher or my mother

I: Has your mother ever taught you anything about HIV?

R: No she hasn’t

I: Who would you want to teach you more about it, your mother or your teacher?

R: My teacher

I: And why is that?

R: My teacher might be able to explain it better to me than my mother?

I: Have you ever heard of stories concerning pregnancy? Or someone getting pregnant?

R: I have

I: Who?

R: A relative of mine got pregnant?

I: Who said that?

R: My mother was telling my grandparent

I: Your biological mother?

R: Yes

I: Was telling your grandparent?

R: Yes

I: Where else have you heard about stories about pregnancy?

R: (Long pause)

I: Have you ever heard about it at school?

R: No I haven’t

I: Do you ask yourself any questions about pregnancy?

R: No

I: You also don’t know anything about pregnancy?

R: Yes

I: Do you want to know things like how people get pregnant?

R: I do

I: Who would you want to teach you about it?

R: My mother]

I: You open up to her?

R: Yes

I: You’re biological mother or your stepmother?

R: My stepmother

I: You open up to her?

R: Yes

I: Have you experienced any changes on your body?

R: Yes

I: You experienced changes?

R: Yes

I: What are these changes?

R: My father used to beat us up before but now it has changed

I: He has stopped beating you?

R: Yes

I: Why would he beat you up?

R: When one of us does something wrong, he would beat us

I: When one of you does something wrong =, he would beat all of you?

R: Yes

I: Have you started having armpit hair?

R: No I haven’t

I: You haven’t?

R: Yes

I: Have you heard about people starting to grow armpit hair?

R: No

I: Have you ever heard of people’s breasts getting bigger?

R: No

I: But you have seen people with breasts?

R: Yes

I: Have you ever heard of people going to initiation ceremonies?

R: No I do not know about initiation ceremonies

I: Have you heard of people starting periods?

R: No

I: You haven’t heard of it?

R: Yes

I: Have you ever had a period?

R: (Long pause)

I: You haven’t?

R: Yes

I: Have you ever gone to an initiation ceremony?

R: I haven’t

I: But people do go to initiation ceremonies?

R: Yes

I: Have you ever seen anyone going there?

R: No

I: You just heard about people going to initiation ceremonies?

R: Yes

I: Have you ever heard of boys and girls relationships?

R: No I haven’t

I: You haven’t heard that a certain girl is in a relationship with a boy?

R: I have

I: You have right?

R: Yes

I: If your body started to change, who would you want to talk about it with?

R: I would tell my mother

I: If you saw that your body was changing, who would you tell?

R: My mother or my father

I: You told me that you have heard of boys and girls having relationships right?

R: Yes

I: Who did you hear it from?

R: My friends were talking about it. They said that one of the girl’s relative was in a relationship

I: How often do you hear that adolescent girls get involved in relationships?

R: It is not often

I: It is not that often?

R: Yes

I: Why do you think they get into relationships?

R: I don’t know why they do it

I: You don’t know?

R: Yes

I: You don’t know what they do in relationships?

R: Yes

I: Have your parents ever told you anything about relationships?

R: They have never

I: Is there anything you want to know about boys and girls relationships?

R: Yes there is something I want to know

I: And what is it you want to know?

R: What do boys and girls do when there in a relationship?

I: Who would you want to talk about it with?

R: My mother

I: With your mother?

R: (Long pause)

I: Concerning your life, it could be a hardship you have faced in your life as a girl or hardships other girls face in this village

R: Yes

I: What is it?

R: A long time ago when I used to live in BK, I was sent on an errand with my sibling in the evening. There were a lot of bushes because it was the rainy season. Then a man in a jacket approached me and asked me to go into the bushes with him. I told him that I didn’t want to but he kept on asking and lying about things like how no one would ever know and how he wanted to give me 2 thousand kwacha in the bushes.

I: What happened next?

R: The man ran away after he was spotted by my mother

I: She saw him?

R: Yes

I: What year was that?

R: A long time ago

I: What did your mother say when this happened? Did she sit you down and talked to you?

R: Yes, she told me to always to refuse strangers from taking me anywhere. Even if they told me that my mother sent them, I should always say no.

I: Is that all you wanted to tell me?

R: Yes

I: Thank you very much for agreeing to sit down with me and chat.
