# Supplementary material for: Social support and very young adolescent girl’s knowledge on sexual relationships: A comparative qualitative study of Girl Only Clubs’ participants and non-participants in rural Malawi
Source: PLOS Glob Public Health. 2023 Jan 12;3(1):e0001339. doi: 10.1371/journal.pgph.0001339 (PMC10022037; doi:10.1371/journal.pgph.0001339)
Supplement: S1 Data — (ZIP) [file pgph.0001339.s003.zip › Anonymized/IDI_ND_YY_9.docx]

**Location**: YY-NO Village

**Date**: DD/MM/2020

**Time**: 10:15 am

I: I just want to know, what is your name?

R: LM

I: In which class are you?

R: 8

I: Are you new in grade 8 or you’ve just written the exams?

R: I have written the exams

I: How old are you?

R: 12

I: How many are you in your family?

R: 3

I: Who is the first born?

R: LN

I: How old?

R: 15

I: The second born?

R: Myself

I: The third one?

R: V

I: How old is V?

R: 8

I: In what class is V?

R: 3

I: Whom do you stay with?

R: My mother

I: Only your mother?

R: With my dad too

I: Biological mother and father?

R: Yes

I: Alright, at the time you are not working at home, what do you do?

R: When we are not working at home, we play and we also find time to ready when schools are open.

I: You read, and also play?

R: Mmm

I: Alright, what is your day like when you woke up in the morning?

R: My day?

I: Yes, isn’t it that you are not going to school?

R: Yes

I: Mmmh

R: I woke up, clean my face and start working, wash plates and there after I should go and fetch water and after fetching water, time will be up, and after that we should eat breakfast. And then they tell us what kind of relish to cook, and later she comes to cook nsima.

I: Your mother cooks?

R: Yes

I: Do you have time to play?

R: Yes

I: Where do you go to play?

R: At the ground

I: What kind of games do you do?

R: Netball, holleyball.

I: Apart from the ground where else do go to play?

R: At home

I: What kind of games do you play at home?

R: *Phada, chipako.*

I: Apart from home and at the ground, where else do you go to play?

R: At school, during break time.

I: In all the places where you go to play, do you think they are protected?

R: No, there are other places which are not protected, like on the ground.

I: What do you fear that it may happen at the ground?

R: We fear that other people may come to rape us.

I: Is it common here, girls of your age being raped?

R: Yes

I: Have you ever heard about a girl of your age being raped?

R: Yes

I: Where?

R: She was raped at the river.

I: What was she doing at the river?

R: She followed her parents who were farming at the river bank.

I: So, what happened?

R: The person who raped her, was never known.

I: Did they report at police?

R: No

I: Was the girl, a school going child?

R: Yes

I: What are other places where you feel you are not protected?

R: At school, sometimes, boys would touch you on private parts.

I: Alright. What do they usually do?

R: They would pretend to be playing with you while they want to touch your private parts.

I: Have you ever been to a place where you felt that you were not protected?

R: No, it never happened.

I: Most of the times, whom do you chat with?

R: Most of the times, I chat with my siblings.

I: Who is your best friend?

R: MY

I: Why is MY your best friend?

R: Because we help each other with school work.

I: What else?

R: We also help each other that we should be going to church

I: Alright, what kind of things do you discuss when you are with MY?

R: We tell each other that we should not be playing with boys since some boys are not serious.

I: Do you have girl chats?

R: Yes

I: What do you tell each other?

R: That when we finish school we should become a nurse, pilot or driver.

I: Have you ever met some problems in life and you wanted to share them with someone?

R: No

I: There are no problems that you’ve ever met?

R: Mmm

I: Or if you may encounter a problem, whom would you tell?

R: My mother

I: Why would you tell your mother?

R: Because I trust her

I: You said you are staying with your mother, right?

R: Mmm

I: Are you happy staying with her or there are problems that you encounter there?

R: I still love to stay with my mother

I: Why?

R: Because she loves me

I: How?

R: Because when I asked things she provides them.

I: Do you think at the place/house you are staying, you are protected?

R: Yes

I: Apart your household, do you also stay with other people surrounding you?

R: Yes

I: Have you ever felt that you have been exposed or you were protected?

R: No.

I: Okay. For how long have you stayed with your mother and father? Have you stayed with other people?

R: No, I have stayed with my parents ever.

I: Alright. How do you think you can feel free to discuss issues of life with?

R: My mother and my aunt

I: For your mother, you said it is because you trust her, what about your aunt?

R: I trust my aunt because she is the one who gave me my name and she cannot do useless things.

I: Alright, this aunt, your dad’s sister or your mom’s sister?

R: My mom’s sister.

I: Alright. How you ever heard about HIV?

R: Yes

I: From where?

R: School.

I: The very first time, where did you hear it from?

R: From my parents.

I: Particularly which parent?

R: My mother

I: What did she say about HIV?

R: She told us that HIV is dangerous we should not share sharp things.

I: How dangerous?

R: She said that it can kill

I: You mentioned that you’ve also heard this at school, what have you heard at school?

R: They told us that HIV is dangerous since when you contract it, you never get well all your life.

I: Were you being taught or you were being given counseling?

R: We were learning

I: Which subject was that?

R: Life skills

I: Alright. Do you have questions concerning about HIV which you would like someone to answer you?

R: Yes

I: Whom do you want to answer your questions about HIV?

R: A doctor

I: Do you think girls of your age are at risk of contracting HIV?

R: Yes

I: Why do you think so?

R: Since sometimes girls can be raped by someone who has the virus and the girl can contract it.

I: What kind of questions do you have, and would you like to know?

R: My question is; why is it that a person who contracts it never gets healed?

I: Have you ever heard about pregnancy?

R: Yes

I: Where did you first heard about it?

R: At school.

I: On what occasion?

R: From my teacher

I: Was it a lesson?

R: Yes

I: From which subject?

R: Life skills

I: What did the teacher say?

R: If a girl starts having menstrual period and she has sex can get pregnant.

I: Where else have you heard about pregnancy apart from school?

R: None

I: Alright. Did you ask questions on that day, about pregnancy?

R: No

I: Have you ever discussed about pregnancy with your best friend?

R: No

I: Do you have questions that you would like to ask about pregnancy?

R: No

I: You are smiling, anything else that you would like to know about pregnancy?

R: Nothing

I: Alright. Have you noticed growing puberty hairs under your arm pits or having breasts?
R: Yes

I: Have you ever told anyone about it when you saw it?

R: Yes

I: Who?

R: My mother

I: What did you tell her?

R: I told her that I surprised that I have started growing hair under my armpits and she told me that it was a sign of puberty age starting.

I: Have you ever heard someone talking about that before you saw them on your body?

R: Yes

I: Where did you hear it from?

R: From the teacher when she was teaching Life Skills at school.

I: Okay. Do you have any questions concerning growing up?

R: No, I don’t have.

I: Do you want to know more or not?

R: I would like to know more.

I: Whom would you love to talk to if you want to know more?

R: My grandmother

I: Why?

R: Because she is old enough

I: Alright. Do you have a boyfriend?

R: No

I: The first time you heard about boy and girl relationships, where did you hear it from?

R: From people

I: Like who, your friends, older people?

R: Older people.

I: When they were discussing?

R: Yes

I: What were they saying?

R: They were saying that if one does not have a boyfriend then you are not in fashion.

I: What else?

R: That’s all

I: In this community, how common are relationships of boys and girls of your age?

R: They are few

I: For those who have such relationships, why do they do that?

R: They say that they would like to find money.

I: What else?

R: Nothing

I: Was there any time when you discussed with your parents about boy and girl relationships?

R: No

I: Alright. Are there things that you would like to know about relationships of girls and boys?

R: Yes

I: Whom would you want to discuss that with?

R: I would like to ask those who have such relationships as why they do that and the benefits they found it, because I heard that it can destroy their lives.

I: I would like to know if there is anything that you would like to tell me, or any problem that you have met and you would like to share as a girl?

R: [long pause]

I: Have you ever met any problem that you would like to share with me?

R: No

I: Or anything we didn’t discuss concerning girls?

R: No

I: Alright. Thank you very much for your time.

I: I forgot to ask you, who do you want to be when you finish education?

R: A nurse

I: Why a nurse?

R: I admire my aunt who is also a nurse.

I: Oho, thank you. What position were you in last exams before writing PSLC?

R: I was on position 5

I: Alright thank you.

END
