# Supplementary material for: Social support and very young adolescent girl’s knowledge on sexual relationships: A comparative qualitative study of Girl Only Clubs’ participants and non-participants in rural Malawi
Source: PLOS Glob Public Health. 2023 Jan 12;3(1):e0001339. doi: 10.1371/journal.pgph.0001339 (PMC10022037; doi:10.1371/journal.pgph.0001339)
Supplement: S1 Data — (ZIP) [file pgph.0001339.s003.zip › Anonymized/IDI1_R1_XX1_May.docx]

District: XX

IDI ID: IDI5_R1_X1_May2019

Location: B

Interviewer: XXX

Data collection date: DD/MM/2019

I: So as I explained earlier right, I am XXX I have come from XYZ and we are working together with XXX Organization concerning the issue of clubs of girls of XXX Organization, or maybe here we call it XXX Club groups right? Where girls like you take part in these groups. So as I explained earlier, our mission is to just want to understand how your life is as a girl in this village and also how the clubs of girls of XXX Organization are beneficial for girls like you in this village. So as I explained, you should feel free, don’t feel uncomfortable. There is no question that will be; where we will say this you have answered well or you have not answered well. Any answer that you will give me will be important to me.

So first of all, I wanted to understand your life as a girl who is growing in this village. I wanted to know that as you live, maybe as a girl, how does your day go like? Like maybe when you wake up in the morning, what it is that you do at home, where you like to go, where you like to play, as a girl who is growing in this village. So you should explain to me that, You; what do you like to do, whether when you wake up in the morning, how dies your day go like?

P: Okay. when I wake in the morning, I first of all sweep the yard, when I finish I wash the dishes, scrub the pots, I should cook porridge, I should eat; I should then go and chat there, at my in-laws house there. After that I go and search for relish, when I search for relish, I cook Nsima and eat.

I: Mmh? I wanted to know that in this home whom do you live with?

P: My mother.

I: Who else?

P: My father.

I: Mmh? Do you have siblings?

P: Yes.

I: How many siblings?

P: 4

I: 4 siblings?

P: Yes.

I: Alright. Who is the first born in your family?

P: MMM.

I: A boy?

P: Yes.

I: Mmh? Who else?

P: XXXX. A boy also.

I: Mmmh, then?

P: Then us were born; twins, me and my brother.

I: Okay. Alright. What about your brother, what does he do when he wakes up in the morning?

I: He just; other days he sweeps, other days I am sweep; me.

I: Mmmh. What about cooking?

P: We take turns.

I: You take turns?

P: Yes. I cook in the afternoon; they cook in the evening.

I: The boys?

P: Yes.

I: Mmmh. everyone meaning works chores in this home?

P: Yes we take turns.

I: Okay. Now you told me that you go for a chat at your inlaws house there; there, whom do you chat with?

P: My in-law.

I: That your in-law?

P: Yes.

I: How old is she?

P: Umm, just like my age. The one who was passing here; the one who was going there.

I: Mmh?

P: Yes.

I: Is she married?

P: Yes she is married.

I: So what issues do you chat about?

P: We just talk about these girly things. She tells me about her background. That when she was a girl she liked being in sexual relationships but still the relationships were just for fun.

I: Mmh?

P: Yes. So she tells me. So I said that “You, how many sexual relationships did you engage in?” and her, saying “Aaah, Me I only engaged in two sexual relationships, then came your brother to marry me.”

I: Okay. Relationships just for fun, when you say relationships just for fun what do you mean?

P: She says it is just to tell us that somehow getting involved in so many sexual relationships; it is not good.

I: Okay. Her, she is engaging in many sexual relationships?

P: Me?

I: Her.

P: No. She says only two, which is not; it is not a lot.

I: Okay. But that is not a lot?

P: Yes.

I: Alright. Other issues that you feel comfortable with her, what are they concerning on what you tell each other in girly things?

P: I am able to tell her when I am maybe having my monthly period.

I: Mm

P: I tell her that “iii, me; this week’s monthly period, my stomach was just paining me. And her she says, “iii, me; too sometimes it happens to me.”

I: Okay. You, when did you start having your monthly periods?

P: Umm when; this month I haven’t had it yet. I have it on the 25^th^ .

I: Okay. But you started; when did it start? That you should start having monthly periods like a girl?

P: The month of May, that has just ended.

I: Last year?

P: Yes.

I: Okay. I just wanted to understand, now when it happened that you have started having monthly periods, whom did you tell?

P: I told my in-law.

I: You told your in-law?

P: Yes.

I: Okay.

P: My in-law is the one that told my mother.

I: Okay. So what kind of counseling did you receive?

P: They told me that when it is that I am having monthly periods, my things should be taken care of. In the morning when I bath, whether I am bathing at a bathroom that others use, when I wash my under wears, I should take care of them, not putting them at the bedroom or at your mother’s, or in a house where many people sleep, putting them anyhow, No. I should put them where only I know.

I: Okay, other counsels that you received at that time what were they?

P: They told me that; while I am having my monthly period and when I have stopped, right?

I: Yes.

P: If I just have sexual intercourse with boys, I can get pregnant.

I: Now that you should not get pregnant, what did they say you should do?

P: I should reduce that, having boyfriends, I should reduce; whether I am having or I am not having, I should just stay. And others also told me that “whether having boyfriends, but you should use a condom.”

I: Okay. Who else, the one who told you that; the others?

P: My in-law.

I: Your in-law?

P: Yes.

I: Umm alright. I wanted to know that on this issue of not getting pregnant and others, have you ever discussed issues like of HIV/AIDS?

P: Yes we discussed. I was just explaining to my in-law because this other time I fell sick and I went the hospital, right? And at the hospital they were saying “get tested, get tested.” So I did not go for testing, I just went where? I just went for consultation, received drugs and came here. The I asked my in-law that; I told her that, “I went to the hospital, and people were talking about getting tested for AIDS.” “Did you get tested?” and I said, “iii, me I did not get tested I was afraid.” She said “iii, You; just go and receive, and get tested, you will receive counseling there.” So I went for testing. Then they asked me that “you have come here so that you should get tested and hear; you should know your status?” and I agreed saying “yes.” if we find you with the HIV virus, you will start receiving drugs today, if we don’t find it, then your body is okay. So I got tested, came back, I reached; they tested me, they told me to go outside and wait for results. I waited, they called me that I should go, I went, and found that I did not have the disease.

I: Alright. But what kind of counseling did they give you at the hospital?

P: They told me that, now that we have found you negative, don’t regard yourself as “I am Me.” No. just continue taking care of yourself because maybe here we can test you and say you are negative but the next time you come again we may find you with the virus.

I: Umm alright. Where else do you hear messages like these of HIV in this village, where is it?

P: When we go at the XXX Organization.

I: Okay, when you go at the XXX Organization?

P: Yes

I: What do you discuss?

P: We discuss; they said that, they were asking, right?

I: Mm

P: What is HIV? So us we were responding that “HIV is a virus that infects AIDS.” “What about what is AIDS?” “Now that was when we were saying AIDS is a disorder of diseases. And then they were asking that, “disease like what and what?” and we say “as a person, you might feel maybe diarrhea, headache, feeling cold.” And then they said “Oh, others meaning you don’t know them.” and they said that “we will remind again next week.”

I: Umm. Okay. Alright. I wanted to know, you have said that most issues you discuss with your in-law, for example like these girly issues, about relationships, what others so you discuss with your in-law? Maybe as a person you might have questions especially on these issues of relationships, other questions which you have, which you are able to discuss with your in-law are like what? You as a person, maybe you have things that when you are alone you wonder saying “how do such things work, and who should I ask?” what kind of questions do you have? Those that maybe you are able to ask your in-law or maybe those that you have never asked your in-law; you have a wish of learning as a girl because maybe you are growing. Maybe as you are growing like that, you start developing breasts, whatsoever as you are growing, what thought do you have and whom would you like to discuss them with?

P: I am sometimes able to ask my in-law, because this other time I was in a relationship with a certain boy, and him he loved sexual intercourse, right?

I: Mm

P: So I said that, me; I said that “I don’t want,” and he said that “if you refuse, me and you, our relationship will end.” I agreed that even if it ends. I came here I asked; I asked my in-law. She said “Aah you; leave him, if he is talking about that, leave him. There is something that he knows, don’t waste time with him.” So I stopped it.

I: Okay. So I wanted to ask on that; when you have talked about a relationship with that boy. As you are having boyfriends, what is the reason for having boyfriends?

P: Nothing, you just do it that maybe my friends are doing it, right?

I: Mm

P: You are walking with your friends and all your friends have boyfriends, so you too you just do it, saying “Aah I should be left alone?”

I: Now in the relationship, what do you do?

P: Some have sexual intercourse.

I: But you, for example with this boy the one you had a relationship with, how was your relationship like?

P: Nothing, we were just chatting.

I: You were just chatting when you meet?

P: Yes.

I: While knowing that this is my boyfriend? How did you differentiate, since when he is your boyfriend, there are also other boys who you chat with, right?

P: Mm

I: So how is the difference between this boy you are just chatting with and this boy who is your boyfriend?

P: A person whom you are just chatting with you wouldn’t; you are chatting with him, right?

I: Mm

P: You wouldn’t say to him that “aa You, don’t shame me, one day I will help you.” So when it is that maybe the boys you are just chatting with them, you may maybe swear at each other, it is of no use.

I: But when it is a relationship? I wanted to see the difference between chatting with a boy whom you just chat with and this boy who is your boyfriend. Is it different? I thought you have said that you were not having sexual intercourse, right?

P: Yes.

I: These ones you don’t have sexual intercourse with, this one you don’t have sexual intercourse with. What is the difference?

P: The difference is that you are able to respect him. But the other one, when you maybe meet him somewhere with your mum you can talk to him. But your boyfriend; even when you are with your mum, you just pass him. And continue with your journey.

I: Without talking to him?

P: Without talking to him.

I: Umm. But then what is the good thing of it, there is a difference, but then what is the good thing of it? What do you benefit in a relationship?

P: They are able to give you money.

I: Money? Umm. What else that is he able to give you as your boyfriend?

P: He gives you money, and the money you use it to buy soap.

I: Umm. Alright. How many boyfriends have you had?

P: One. This one that I have ended it with.

I: Umm. It has ended because he wanted to have sexual intercourse with you?

P: Yes. because I asked my in-law. And my in-law said “you should end it with that boy.” So I ended it

I: Umm. Alright. So I also wanted to that apart from your in-law, which other person do you feel comfortable with?

P: This my other friend who stays this side.

I: Mmh?

P: This my other friend who stays this side.

I: Okay. With that one also you discuss your girly issues?

P: But it is not much.

I: Okay. I also wanted to know that in this village, where else are girls able to go, to talk about issue to deal with maybe HIV, issues of sexual intercourse, issues of maybe preventing pregnancy, prevention getting sexually transmitted diseases, where is it?

P: When they do social gatherings like, they may come maybe members of parliament, so they say, “there have come organizations.” They mention the organizations, right?

I: Mm.

P: Whether XXX Organization or Youth Compact. They mention them. And also many people are able to go to watch games. When there is a game, many girls are there and boys are also there. So they discuss there. And others say that, “I wouldn’t get pregnant because I use a condom.” And some say, “we don’t like it when using a condom because you don’t eat a sweet while it is in its paper.”

I: Umm. Those are boys?

P: Yes.

I: Umm. Alright. What about other organizations? In this village maybe are there other people who where girls like you maybe are able to go and seek counseling?

P: No. it is only at the XXX Organization, when it is Tuesday we go and meet, discuss and comeback.

I: You meet on Tuesday?

P: Yes.

I: Each and every week?

P: Yes.

I: I wanted to know that how you chat with your parents right?, for example your father. Have you ever discussed issues to deal with HIV, or about sexual intercourse, with your father?

P: About sexual intercourse we have never discussed before.

I: Mm.

P: But about HIV. He was asking us that, “have you ever gone for testing?” and I said that, “I have never gone for testing.” And he said that, you should go and get tested so that you should know how you are in your body.” Then when I went I explained to him that “I went,” and he said that, “so have they found you with the virus?” “No I don’t have.”

I: And then what other kind of counseling did he give?

P: He said that “you should work hard in abstaining. Because if you get excited saying “ii, since I don’t have the virus;” you should do things that you should not have it because these days people are in danger.”

I: Alright. What about your mother? Especially on these issues of sexual intercourse; whether on HIV? Does she sit down with you, other days? I wanted to know if maybe you have free time to chat issues like these with your mum.

P: We sometimes sit. We sit; me, my mother, my in-law. We sit, three of us. Talking issue like those saying, “Beware of your movements, because these days it is dangerous. Some people get you pregnant and run away or refuse saying “it is not me.” While deep down their hearts they know that it is them.” So when we talk of the issues, she just laughs. Sometimes I ask her that; my mother, asking here that “but did you go for testing?” “she says, yes I went, but they told me that I should go again sometime since they found me negative.”

I: Umm. Alright. I wanted to know that in this village, young girls; especially your age who are maybe not married, if it was that a girl has been found pregnant; a girl has been found pregnant or maybe she has been found with a disease, maybe sexually transmitted diseases, where is it that she can run to and report? Or to seek help? Or maybe if she had questions concerning issues like these, where is it that girls go? Or if it was You, for example it has happened to you, you have been found maybe with sexually transmitted diseases or maybe you are pregnant, where would you run to and report?

P: People go to the hospital for testing, and when they go to the hospital for testing, they are told at the hospital that “you have it or you don’t have it.” So when it is about pregnancy, many people are also able to go to the hospital and get tested for pregnancy. When they have found you pregnant, they tell you; if you dint have, they also tell you. So when you get here you are able to tell whether your mum, or your friends that “I went to the hospital, I have been found with the virus. Or I went to the hospital they did not find me with the virus. Or I went to the hospital they have found me pregnant.” You are able to tell your mother or your friends which you rely on. Or telling them that, “I went to the hospital, they have found me pregnant.” The they ask, “now when you sit down and think, this pregnancy, who is responsible for it?” so if you know the boy whom you were having the relationship with, right?

I: Mm.

P: You are able to mention his name that “I was having a sexual relationship with such such.” Now the parents tell your relatives, maybe your brothers and your brothers; who are older in your house are able to go at the boy’s house and ask the boy.

I: Umm. I wanted to know that when you go to the hospital; the youth maybe; especially the youth as young as you are,

P: Mm.

I: How do they welcome you? Even you, when you went for testing; you told me that you went for HIV testing. Considering that you are young, how did they welcome you at the hospital?

P: They welcomed me in that “welcome, feel free. Who has told you to come here? And get tested?” so we said “on our own we have just decided, so that we should know how my body is.” They said “okay. Now that you have come here, first of all, if we find you with the virus, what will you do?” so we responded that, “we will just accept it.”….

I …You went two people?

P: Yes.

I: With your friend?

P: Yes.

I: Mm.

P: “We will just accept it” then they said, “okay, we should start, have you ever heard what HIV is?” and we spoke. Then they said, “or what is AIDS? If we find you with the virus, would you say you will throw yourself in the river or what would you do?” and we said “Aah, we will just accept it, what else can we do? If we have it, that’s all; It is not possible that it can vanish just today. If we don’t have it, that’s all, then it means our bodies are okay.” And then they counseled us; we went for testing, then they called us to receive our results; we received and came back.

I: Umm. Alright, alright. Okay so I also wanted to know how it is in this village especially in this issue of relationships. Where boys should be involved in sexual relationships with girls, what are your views on that? why is it that girls are involved in sexual relationships; here?

P: People just do it because of their friends who they chat with, right?

I: Mm.

P: For example like me I am chatting with my friend but my friend has a boyfriend. So I don’t have a boyfriend, right?

I: Mm.

P: It may happen that; your friend maybe his boyfriend gives him money or what. So she would say “you should also do it; see we have money. Then you will not be spending our money, because we cannot be receiving money from boys and while you don’t. so you will not be spending our money. It will not be possible.”

I: Like you; for example you said that you had a boyfriend whom you ended it with because he was forcing you to have sexual intercourse. You; what made you to have a boyfriend?

P: My friend the one I was walking with had a boyfriend. So this my friend was like when we go maybe for a chat, she carried money. So I said “that money you should buy things we should share.” And she said “It will not be possible because Aaah, you should just be spending our money, cant you have your own boyfriend?” so I said “should I have a boyfriend at this age?” “Aaah, You; us we just do it not be having sexual intercourse with them, No. we just want to spend his money.” So she had a boy who was disturbing her saying “tell your friend that I want her to be my girlfriend.” So I said “I don’t want.” And she said, “Aaah, you; just say yes. if you don’t, you will not be chatting with us.” So I just agreed because when I say I have a friend I chat with, it is her; when we are going maybe to play netball.

I: Umm. Alright. So as you live, maybe with your friend, are you able to discuss how those sexual relationships are doing? With your friend?

P: Her, she…

I: …discussing that maybe how are your relationships doing, that maybe where did you meet or what. How does it go like?

P: Her, she tells me. When it is that she went away to meet with her boyfriend, she tells me.”I went. I found him or I did not found him.” So I ask that “when you found him, what did you do with him?” “Aaah we were just chatting. Don’t you know that he is my friend?”

I: Umm. Alright. I just wanted to know that these relationships, how is it? Do most girls have sexual intercourse with their boyfriends? How is it here in this village? These issues of sexual intercourse?

P: Some are able to have sexual intercourse with their boyfriends; others what? It does not happen.

I: Umm. You; do you think it is good or? What are you are views on this?

P: It is not good.

I: Why not?

P: Because you might be having a sexual relationship with a person, he will be forcing you and you will be refusing; to have sexual intercourse with him, right?

I: Mm.

P: He will be forcing you, you will be refusing. He will tell you that the relationship should end. It is up to you for the relationship to end; it is up to you to say “this relationship will not end.” As for me I agreed that even if it ended, It doesn’t concern me.

I: Mm.

P: So there are some of friends who do what? They don’t agreed. They say “iii, that’s all, what is to happen should happen but for me, this relationship will not end.”

I: Umm. Alright. But you a s girls, don’t you have feelings inside you that you should have sexual intercourse with this boy? Apart from the issue of money.

P: It happens, having the feelings; but on your own you say “Aaah, if I follow my heart, I will be the one in trouble.”

I: Alright. Now how common is it in this village that young girls like your age, being found that they are married?

P: Most people, in that area, right?

I: Mm.

P: Most people are married. They just say “Aaah if my friends are doing it, me too I need to be doing it. So when they are told to go and have sexual intercourse, they say “I will what? I will go.” In that way of having sexual intercourse they have gotten pregnant, they have been refused. The baby there has been left with who? Isn’t it the girl?

I: Mm. so they are found married?

P: They are not married. They just stay with their mothers.

I: They just have kids?

P: Yes.

I: What about those who are married? Are there here? Girls who are married?

P: Yes there are.

I: What kind of men do they get married to? What kind of men are they? Older men or those of their age?

P: Of their age just get them pregnant and refuse. Then there comes some older men who are not of their age, right?

I: Mm.

P: Just to report to their mothers saying “you see how your daughter is suffering, she has a child, she is not finding any help.” It is up the parents to agree that “Aaah he should marry my daughter. Because with how she is suffering, I too cannot manage to feed her and her daughter. It will not be possible.” So they get married with men who are not of their age.

I: Umm. What are your views, that is it good that girls should get married while they are young?

P: It is not good.

I: Why not?

P: Because that you get married while your body organs are not yet strong enough. So it is not good. Or maybe getting married because you are being forced. So in the end, the husband can be maybe abusing you.

I: Umm. Alright. Have you ever gone to a girl’s initiation ceremony?

P: Yes I have.

I: At the girl’s initiation ceremony, when did you go? When you had started having your monthly periods? Or sometime when you had not yet started?

P: Here they go; while we are kids. We are initiated while we are kids.

I: For example like at what age?

P: Eight years maybe nine years.

I: While you haven’t started having your monthly periods?

P: Yes.

I: So at the initiation ceremony there, what activities are there?

P: They just counsel you that “don’t regard yourself that now that you have come at this stage of being initiated, you are an elder. Saying “because I was initiated I can go and engage in sexual relationships because I was initiated.” It is not a good behavior. If you have come here, you have that you should learn good behaviors; your mother and father you should respect them. Not that because you were respecting them you should say “Aaah, now I can be rude since I have been initiated.” It is not a good behavior.”

I: There are no activities concerning sexual intercourse? At the initiation ceremony?

P: Some people tell you; but it is up to the willingness of the person. Some people tell you that “when you leave from here, you should go and have sexual intercourse with a boy.” But some say “it is not a good behavior.” Because as a person…..

I: But; me, I want to hear what you were faced with, when you were being counseled. So I want to hear. I want to learn; to hear that what was your counsel like? What you were taught.

P: They told me that, now that you have come and being initiated here, it should not be that you should be going out, having sexual intercourse with men or you should find a boyfriend; it is not good. Because if you have been initiated, it means that your parents have seen that you have reached the stage of initiation.

I: Mm.

P: So not that you should be rude to your parents, No.

I: Alright. Now there is something that you are not explaining. When you say initiated, what is it?

P: They just give you counseling.

I: okay. So I wanted to hear, what kind of counseling is it? They say you should not do what? Anything, whether not having sexual intercourse. But the counseling that you receive. For example you have said they say you should respect your parents, “don’t be rude.’ what else?

P: They said that at your mothers bedroom, you should not enter. Be it that your mother is bathing; if you want to bath, you should first of all knock if there is a person or there is no person. If there is a person, you should not enter. Or if in the house there is your mother and father, you should not enter.

I: Umm. Alright. Other counsels, whom do they say you should also give respect to?

P: To elders. “When you see elders carrying things, you should go and help them carry.”

I: Alright. On the part concerning getting pregnant or those we discussed about like AIDS, at the initiation ceremony do they give counseling concerning these?

P: No they don’t give because they see that there are kids.

I: Okay. They don’t talk about HIV/AIDS?

P: They don’t talk. Because a child like me; I was initiated when I was seven years old, right?

I: Mm.

P: I was still a kid. They wouldn’t say pregnancy you can get it or pregnancy you cannot get it. They know that there if you have been initiated while young, you have not yet started having monthly periods, so how can you get pregnant? When you are young?

I: Umm. Alright, they don’t say that you should go and have sexual intercourse?

P: They don’t say.

I: Or the disadvantage of doing that?

P: No.

I: Okay. Alright, we are concluding. So you have said that you don’t go to school, right?

P: Yes I don’t go.

I: Why did you stop going to school? Have you ever gone to school before?

P: Yes I have ever gone before.

I: In which class did you stop?

P: Eight.

I: Oh standard eight? Then it is not very long. When was it when you were in standard eight?

P: [Silence]

I: When did you enter in standard eight?

P: 2014.

I: 2014?

P: I think so, yeah.

I: And then you stopped. Why did you stop going to school?

P: I wrote exams for the first time, I failed; I got a pass, right?

I: Mm.

P: Then when I got a pass, my friends were laughing at me :Aaah, you; you should be going where;” because here those with a pass go in the afternoon. Some go in the morning others in the afternoon. “you should be going for afternoon schooling? Are you an owl or what?” so I repeated the class. So I went back to school that I should repeat. At the time of paying money that we should write exams, I told my mother “you should give me money to pay for exams.” She said “you should tell your uncle.” So when I told my uncle he said “I have already paid the money. You can go.” On the day of sitting plan when I got there, I found that my name was not there. They said that “you did not pay exam fees. On this paper there are names of people who paid for exams.” So I did not go there again because it hurt me. I had learnt all this time, and then him telling me that “I did not pay for you the money.” It disgusted me and I did not go there again.

I: Okay. So since 2014 up to today you have not gone back to school?

P: I did not go again.

I: So what do you do now? Aren’t your friends in school? In the afternoon what do you do?

P: Others don’t go. So I chat with those that don’t go.

I: You just chat?

P: Yes.

I: So your thoughts when you think of your future, what are your thoughts since you dropped out of school?

P: I think; I say even now that but if there was found someone to pay me school fees, I would have gone to school. Because the dropping out I dropped out in standard eight its not that I wouldn’t have passed. I would have passed. So people say that “when we find the money, not know but when we find the money we will pay for you. You will what? You will get back to school.”

I: So your thought are that you should get back to school?

P: Yes I will get back. As long the one to pay for my fees is found.

I: Okay. I forgot to as one question. In the places where you chat, whether for playing football where you go with your fellow girls, how is it secured for a girl. There are other places where girls are abused maybe by men or boys and other people. This behavior here, how common is it? For a girl being abused in any way, in the places where they go?

P: For example at netball grounds, right? There might be elder people so as a boy, you cannot rape a girl, at a place where there are elders. You feel embarrassed.

I: But at what places does it happen that they have been raped? Whether in this village?

P: When they are going like maybe at school, because grass has grown; right?

I: Mm.

P: So if you are going on your own to school, they can rape you. Because even if you scream, that it should reach here, it cannot be heard.

I: But does it happen here?

P: In this village?

I: Yes. that girls are being raped?

P: No. they have never been raped.

I: Alright. But there are some small abuses maybe being touched breasts, maybe not being raped but being touched breasts by teachers or what? Those things here, how often do they happen?

P: They don’t happen. Because like being touched breasts, it happened some other time. Some teacher touched some students’ breasts. She reported the issue to the head teacher was fired.

I: Okay. So you as a girl, do you; what do you see is missing for girls? What maybe you want should happen to girls like you. Especially help on the part of like maybe question which you may have, what you want to know on issues of HIV/AIDS, issues of sexual intercourse whether of prevention or getting sexually transmitted diseases. Help that you think should reach the girls, which is maybe missing, what kind of help?

P: People, right? Maybe to say that here they should be organizations where people can meet, when they meet, we are able to say that if a as a person you want to be buying sanitary pads or be buying what. You are able to say at those meetings with your fellow youth; you can tell them that. “Iiih we should do such things, we should find money.” So you maybe ask your mother for one hundred kwacha and contribute there. When they say “we should share the money we have been saving.” You share and buy what you want.

I: Umm. Alright. Now that you should be found in the XXX Organizationgroup, how were you found? How did you join the XXX Organizationgroup? And when did you join?

P: I started in September. I joined because my friends, right? A lot of them, right? Were saying “lets go and join, lets join.” So I said “I will not join, because I do not know what I will learn there. I stopped school a long time ago. So I should be going there, what will I learn? And they said, “go, go” so I went. And when I went, it was when we were earning about pregnancies; how can we avoid pregnancies, or HIV how can we avoid it. We learnt it there at XXX Organization.

I: Your friends are the ones who told you?

P: Yes.

I: Okay. Alright. So with how you have been at the club of XXX Organization, what have you benefited?

P: I have benefited, right? Because I did not know that us; pregnancy how can we avoid it? Or AIDS how can we avoid it? We did not know. And also that people do cleanliness when they are on their monthly periods, we did not know; most of us; right? Because we learnt while we were young. So we learnt it there at XXX Organization.

I: So I also heard that at XXX Organization there are different activities which girls do there. What do you do?

P: When it is about topics that people should organize comedies, right? We are able to do a comedy or a poem or if the topic is about playing netball, people play netball. We arrange plastic papers, make a ball and play.

I: Other things that happen there, what are they? I wanted to know what happens at the club of XXX Organization, apart from the comedies, you have talked of poems. What are the other things that happen there?

P: Like we are able to; maybe when we learn, right?

I: Mm.

P: About how to wear a condom; everyone when coming; maybe they choose people, right?

I: Mm.

P: You and you when coming, you should bring a penis-like object; maybe some bring a maize cob and others will bring a bottle. The they will dress those objects for demonstration.

I: Umm. Alright. Now have they ever at XXX Organization; your teacher, does it happen that they take maybe some girls who are in need of help; maybe they want contraceptives or to get tested. Does it happen that the teacher is able to send the girls to find help?

P: Yes.

I: Like where?

P: It is like when they want to get tested, right? Or contraceptives, right?

I: Mm.

P: They say “wait, I should call GGG.” Now when they call him, the medical people come right there. At the school. And the people go there.

I: Do you have thought that maybe in the future you will stop this program?

P: No. I wouldn’t stop since we did not know a lot of things; we are learning them from there.

I: Umm. Now would you tell other girls in this village who maybe did not join; that they should also join and benefit in this program?

P: Yes I would tell them that “let’s go and join the XXX Organization club because some of the things which they did not know or some which we have not yet learnt; we would what? Learn them there at XXX Organization.

I: Thank you very much my friend for answering these questions. I am happy for chatting with you. It was a good chat.

P: Mm.

I: Because of your feeling comfortable. So right now we should close. We should finish off on this. I don’t know if you have a question?

P: No I don’t.
