# Supplementary material for: Social support and very young adolescent girl’s knowledge on sexual relationships: A comparative qualitative study of Girl Only Clubs’ participants and non-participants in rural Malawi
Source: PLOS Glob Public Health. 2023 Jan 12;3(1):e0001339. doi: 10.1371/journal.pgph.0001339 (PMC10022037; doi:10.1371/journal.pgph.0001339)
Supplement: S1 Data — (ZIP) [file pgph.0001339.s003.zip › Anonymized/IDI10_R2_YY12_Apr.docx]

District: YY

Location: A

IDI ID: IDI1_R1_YY12_May2019

Interviewer: WWW

Data collection date: DD/MM/2019

**Interviewer**: As I have already said I work for COM and we are doing this study with PC of the USA. We are conducting interviews with various girls in the age ranges of 10 to 14 years who are enrolled in OC GG clubs. We are basically interested in learning your experiences with GG clubs and how you acquire some information on family planning, HIV and AIDS, and other sexual health issues. So, we are talking with girls like you here in Zomba but also in Machinga. So, to begin with whom do you stay with?

**Respondent**: I stay with my mother and father.

**Interviewer**: Okay, any other relatives you stay with? Do you have relatives?

**Respondent**: Yes, I do have.

**Interviewer**: Relatives like you brothers and sisters?

**Respondent**: Yes, we are seven children in our family.

**Interviewer**: Who is the first born?

**Respondent**: xxxxxx.

**Interviewer**: How many are girls?

**Respondent**: There are five girls.

**Interviewer:** how about boys? **Respondent:** One boy

**Interviewer**: Meaning in total you are six in your family?

**Respondent**: Yes.

**Interviewer**: Okay, do you go to school?

**Respondent**: Yes, I do.

**Interviewer**: Where do you learn?

**Respondent**: at NMS primary school.

**Interviewer**: What grade?

**Respondent**: Standard six.

**Interviewer**: Okay…, okay, have you ever dropped out of school due to other challenges?

**Respondent**: No, I haven’t.

**Interviewer**: Mmmh, okay, who do you want to be when you finish your studies?

**Respondent**: I want to be a nurse.

**Interviewer**: Why do you want to be a nurse?

**Respondent**: I am motivated by nurses at the C clinic.

**Interviewer**: What are you doing to achieve this dream?

**Respondent**: I have to abstain from sexual activities and working hard at school.

**Interviewer**: Okay, what do you like doing when you have finished your household chores like, cooking, cleaning the surrounding, and drawing water?

**Respondent**: I like reading.

**Interviewer**: Okay, you read school work?

**Respondent**: Yes.

**Interviewer**: What any other things do you like doing?

**Respondent**: I like playing.

**Interviewer**: Where do you play?

**Respondent**: At my friends’ house.

**Interviewer**: Okay, apart from going to your friends what other places do you like going?

**Respondent:** (silent)

**Interviewer**: Where else do you like going when it happens that you haven’t gone to play, that is during your free time?

**Respondent**: I do go at OC club sometimes I go to church.

**Interviewer**: mmmh, which denomination do you belong?

**Respondent**: COC.

**Interviewer**: Okay, in some places, girls do face some challenges when they go out to play or for a walk. What challenges do girls here face when they go for a walk?

**Respondent**: Girls face problems like being raped, men grabbing their breast and even they can be kidnapped.

**Interviewer**: Those things happen in this village?

**Respondent**: yes.

**Interviewer**: ooh, tell me the situation you have ever witnessed such cases?

**Respondent**: I have never seen such cases.

**Interviewer**: You just hear the news?

**Respondent**: Yes.

**Interviewer**: Has anything of that sort happened to you?

**Respondent**: No, it has never happened to me.

**Interviewer**: Okay, when such things happen where do people report?

**Respondent**: They report to mother groups, school teachers, traditional chiefs and OC club teachers depending on where it has happened.

**Interviewer**: Okay, if such incident happened to you, where are you going to report to?

**Respondent**: At mother group.

**Interviewer**: Why would you report there? And firstly, can you explain what a mother group is?

**Respondent:** You mean those from mother groups?

**Interviewer**: Yeah

**Respondent**: Mrs. xxxx.

**Interviewer**: Tell me what they do?

**Respondent**: They advise girls

**Interviewer**: mmmh

**Respondent:** To work hard at school.

**Interviewer**: Any other thing they do?

**Respondent**: They advise girls not to walk during late hours like at night

**Interviewer**: mmmh

**Respondent:** So, that girls should be doing household chores.

**Interviewer**: Do these women go house by house advising girls or they organize meetings?

**Respondent**: They do organize meetings.

**Interviewer**: They call you for a meeting?

**Respondent**: yes.

**Interviewer**: So, that’s where they do advice you on various topics.

**Respondent**: yes.

**Interviewer**: So, they tell you not to walk at night and that you should be working hard at school?

**Respondent**: Yes, and that we should not be going to video shows.

**Interviewer**: What do they say are the negative consequence of going there?

**Respondent**: We can fall pregnant.

**Interviewer**: mmmh

**Respondent:** And also, we cannot go far with our education.

**Interviewer**: Okay, what other advice do you get there?

**Respondent**: at OC club?

**Interviewer**: no at mother group.

**Respondent**: (silent)

**Interviewer**: How many times do you meet at the mother group?

**Respondent**: We only met once.

**Interviewer**: only once?

**Respondent**: yeah

**Interviewer**: mmmh, you said that you stay with your parents here?

**Respondent**: yeah

**Interviewer**: Have you been staying with them since you were born?

**Respondent**: yes.

**Interviewer**: So, I also wanted to know the roles your father play, does your father advise you about HIV & AIDS, relationships, family planning and about pregnancy?

**Respondent**: No.

**Interviewer**: What about your mother?

**Respondent**: yes.

**Interviewer**: Tell me what happened for her to advise you?

**Respondent**: (silent)

**Interviewer**: Tell me how it started, what happened for her to start advising you?

**Respondent**: She just told us that if we walk around anyhow, especially at night, we can get unexpected pregnancy.

**Interviewer**: She just started advising you or something happened?

**Respondent**: She just started advising me.

**Interviewer**: I know you hear about HIV at OC and mother group, apart from this place tell me the first place you ever heard about this?

**Respondent**: I heard it from my friends.

**Interviewer**: What were your friends saying?

**Respondent**: They were saying that HIV can be contracted through sexual intercourse, sharing of sharp objects like razor blades, needles and using same tooth brush with infected person.

**Interviewer**: mmmh

**Respondent:** yeah

**Interviewer**: You heard all of this from your friends?

**Respondent**: yes, and also at OC.

**Interviewer**: Do you see any difference on your knowledge about HIV before and after you started going to clubs like OC?

**Respondent**: Repeat the question?

**Interviewer**: At first you were not a member of OC, do you see the difference ever since you joined this club?

**Respondent**: At first before joining OC when we were back from school, we were not doing household chores but rather just playing but now we do household chores and thereafter do our studies.

**Interviewer**: Any other thing which has changed after joining these clubs?

**Respondent**: We were also being abused by parents but ever since we joined OC they stopped because we explained to them everything, we learned from OC.

**Interviewer**: At OC what do they teach you about child abuse?

**Respondent**: They teach us examples of child abuse like overworking a child, forcing girls into marriage, leaving children to take care of babies and absenting us from school.

**Interviewer**: What else?

**Respondent:** Like making a girl babysit her siblings without going to school….

**Interviewer:** mmmh

**Respondent:** …never force a girl into marriage.

**Interviewer**: So, all these you we taught at OC

**Respondent**: Yes.

**Interviewer**: Then you were telling your parents?

**Respondent:** yeah

**Interviewer**: mmmh mmmh, okay, what about issues of pregnancy...where did you first hear about these issues?

**Respondent**: (silent), from parents.

**Interviewer**: What do you mean by saying parents?

**Respondent**: I mean my sisters, and also my mother.

**Interviewer**: mmmh

**Respondent:** mmmh. My bothers

**Interviewer**: They ever told you?

**Respondent**: yes.

**Interviewer**: What happened for them to tell you about this?

**Respondent**: (silent)

**Interviewer**: Did they just start telling you…, tell me what happened?

**Respondent**: I have forgotten.

**Interviewer**: Have you experienced your first period?

**Respondent:** What?

**Interviewer**: Did you start experiencing menstruation?

**Respondent**: No.

**Interviewer**: Have you ever discussed this with anyone about menstruation?...

**Respondent**: No.

**Interviewer**: …or anyone else telling you about this issue that as a girl you reach a point when you start menstruating?

**Respondent:** No

**Interviewer:** You have never heard such kind of advice?

Respondent: Yeah

**Interviewer**: Have you ever participated in any initiation ceremony?

**Respondent:** Initiation?

**Respondent**: yes, but I was young.

**Interviewer**: so, they never talked about menstruations?

**Respondent**: I don’t know if they talked about it because I was only four years old by then.

**Interviewer**: mmmh, you were really young. Okay, so you have never discussed about menstruation with anyone?

**Respondent**: I just heard about it.

**Interviewer**: Where did you hear about it?

**Respondent**: I heard it from my sisters.

**Interviewer**: Were they telling you or someone else?

**Respondent**: They were discussing it, I just overheard it.

**Interviewer**: What questions do you have about menstruation?

**Respondent:** (silent)

**Interviewer**: Don’t you think about it or sometimes having a sense of fear as to what will happen if you will ever experience it?

**Respondent**: I do think about this.

**Interviewer**: Tell me what you think?

**Respondent:** I do have questions like what happens for menstruations to start

**Interviewer**: mmmh, okay, any other question?

**Respondent**: and also, how to stop this.

**Interviewer**: mmmh, You, mean how it starts and how you can stop it…

**Respondent**: yes.

**Interviewer**: …but there is no one to ask?

**Respondent:** yeah

**Interviewer**: Mmmh, okay fine, anything you want to know, and about HIV (coughs) what questions do you have concerning HIV which bother you a lot and you want to know more about6, or issues concerning pregnancy… what questions do you have?

**Respondent**: (Short pause), can you repeat the question please?

**Interviewer**: I was saying that what questions do you have on HIV and how one can get pregnant, things you just hear but don’t understand them..what questions do you have that you need to be answered?

**Respondent**: I want to know how one can become pregnant and contract HIV.

**Interviewer**: You haven’t understood this?

**Respondent**: Yes.

**Interviewer**: You would love to understand them?

**Respondent:** yeah

**Interviewer**: Who do you think can be the right person the one you can be so open to approach so that can they can explain this to you?

**Respondent**: My mother.

**Interviewer**: Have you ever asked her?

**Respondent**: No,

**Interviewer**: (laughs), why?

**Respondent**: I will start asking her.

**Interviewer**: Okay, I also want to know more on issues concerning relationships that exist between boys and girls of your age, how common are relationships in this community... do girls of your age have boyfriends?

**Respondent**: I have seen five cases.

**Interviewer**: Mmmh, okay, what causes girls of your age to end up in relationship?

**Respondent**: They do have the desire to have sex.

**Interviewer**: Okay, any other thing?

**Respondent**: (silent), I have forgotten.

**Interviewer**: Do you think it is good to be in such relationships?

**Respondent**: It is not good.

**Interviewer**: How can one end or stop the desires?

**Respondent:** (silent)

**Interviewer**: How can they help themselves?

**Respondent**: Do you discuss such issues at OC or at mother groups?

**Respondent**: yeah

**Interviewer**: What do they say about the desire for sex?

**Respondent**: (silent), mmmh I have forgotten.

**Interviewer**: Have you really forgotten, or they don’t tell you about this?

**Respondent**: At mother group they don’t discuss this.

**Interviewer**: They don’t talk about this?

**Respondent:** yeah, they don’t

**Interviewer**: what about at OC?

**Respondent**: they also don’t tell us.

**Interviewer**: they also don’t talk about it?

**Interviewer**: yeah

**Interviewer**: Ok let us now discuss about your OC club, what is the name of your club again?

**Respondent**: XX GGs club.

**Interviewer**: okay, (yawns), what motivated you to join this club?

**Respondent**: I wanted to find out where I can go to report if my parents abuse me. And I also wanted to know how I can prevent getting HIV/AIDs,

**Interviewer**: So how were you selected? How were you recruited into the club?

**Respondent**: Initially they called for a meeting where our parents were invited, and our parents told us and we started going to the club

**Interviewer**: Mhnnn...so how long have been in this club?

**Respondent**: I started in 2016 until now I am still attending the club

**Interviewer**: Ok...so we have heard that in these clubs there are several services that are offered such as HIV testing and the like, how have you benefited from any of these services?

**Respondent**: We received school bags, pencils, erasers, notebooks, uniforms and we also got tested for HIV

**Interviewer**: Ok, What else have you benefitted from the club?

**Respondent**: I have also benefited some lessons

**Interviewer**: What kind of lessons?

**Respondent**: Lessons about abuse, knowing each other, about boys and girls being equal in doing activities,

**Interviewer**: You have said lessons about abuse, and knowing each other? What do you mean when you say knowing each other?

**Respondent**: When we meet as a group, the first thing should be knowing each other’s’ names,

**Interviewer**: Oh ok...what did they say is the importance of doing that?

**Respondent**: If your friend fell on the road, if you knew her, you would pick them up and take them to their home

**Interviewer**: Oh ok...

**Respondent**: But also when they get sick at school, you would escort them home

**Interviewer**: Ok...so how has your life changed?

**Respondent**: As compared to my past?

**Interviewer**: Ever since you joined the GG clubs, how has your life changed?

**Respondent**: When my friend gets sick, I escort her

**Interviewer**: What else has changed?

**Respondent**: I am making progress in my studies

**Interviewer**: Ok....I also wanted to know in the GG club, you said you joined in 2016, how comfortable are you to participate in the activities? What challenges have you faced in participating in the club?

**Respondent**: Challenges?

**Interviewer**: Anything disappointing that you have met at the club?

**Respondent**: Sometimes when you respond to a questions, if your response is wrong so people laugh at you

**Interviewer**: Ok what else?

**Respondent**: Sometimes people in the club don’t speak nicely to each other

**Interviewer**: If you found out that there is another girl who wants to join the club, how will you encourage them?

**Respondent**: I would tell her that there are good lessons, and that she should tell her parents to let her join and also that it is going to help her with her studies as well.

**Interviewer**: Ok...thank you very much for responding brilliantly to these questions. Do you have any question?

**Respondent**: No, I don’t have any question.
