# Supplementary material for: Social support and very young adolescent girl’s knowledge on sexual relationships: A comparative qualitative study of Girl Only Clubs’ participants and non-participants in rural Malawi
Source: PLOS Glob Public Health. 2023 Jan 12;3(1):e0001339. doi: 10.1371/journal.pgph.0001339 (PMC10022037; doi:10.1371/journal.pgph.0001339)
Supplement: S1 Data — (ZIP) [file pgph.0001339.s003.zip › Anonymized/IDI12_R1_YY13_May.docx]

District: YY

IDI ID: IDI2_R1_YY13_May2019

Location: A

Interviewer: WWW

Data collection date: DD/MM/ 2019

I: Alright . As I explained earlier, right?

P: Yes.

I: I am from COM, I am chatting with different girls in this your area concerning what you learn at the GG club and also more about your life as a girl who has grown up in this village, right?

P: Yes.

I: First of all, I want you to explain to me how is your day like when you wake up in the morning? For you as ZZ? Just like today, you have woke up in the morning just like any day how it is, whether Monday, Tuesday or Sunday; when you wake in the morning as ZZ, what do you do?

P: I wash the dishes, draw water, cleaning the house, doing house hold chores, then play with my friends at the GGs club.

I: Alright. Now at the GG club do you go daily?

P: No. But I just pick my friend from there and teach each other on our own.

I: Your friend from where?

P: From there.

I: She is the one you teach each other with on your own?

P: Yes ,when it is not our meeting day. So that we should not forget.

I: Okay. So you teach each other things like what?

P: About my dreams, about visions, gender, HIV.

I: Okay. About my dreams, what do they say about my dreams?

P: My dreams, “it is up to me to choose the job I want when I finish school.

I: Okay. About HIV?

P: HIV is…(breaths) HIV, they told us that it is a virus that infects a disease……umm..[breaths]..HIV they told us that is a virus that infects when protection is low.

I: Alright. So you have said that when you are done with your chores, then you get together with your friend and talk about GG club.

P: Yes.

I: But are you telling me that each and every day you only talk about GG club?

P: No.

I: Yes?

P: We go to play netball

I: Playing netball?

P: Yes.

I: Alright. I just wanted to know the places that you like to be. You have said at the GG club on some days, other days you get together with your friend and talk about GG club, and also playing netball. But now on other days? Anywhere else that you like to be on other days, apart from going to play netball, where else do you go?

P: I go and chat at CA.

I: You go at CA?

P: Yes.

I: Alright. Where else?

P: At LA?

I: That’s also a chatting place?

P: Yes.

I: Alright. Now in this your village, for a girl like you when you are chatting, maybe you have gone for a chat, maybe to play, what are some of the problems you face?

P: Our friends laugh at us saying, “you are busy with the GGs Club, what will they do for you, they are satanic.” So we don’t pay attention to them. We say, “okay, they are satanic, but we know what we are doing because we are learning. We just want to learn.”

I: Alright. Other things that you face, difficult in your life, maybe abuses that you face when you go to play, playing netball or some other places, what are some that you face?

P: We meet with boys who ask us to be their girlfriends.

I: Mmh?

P: Yes.

I: So when you refuse them, what happens?

P: We respond and then swear at them.

I: They swear at you?

P: We are the ones who swear at them.

I: You swear at the boys?

P: Yes.

I: Alright. What else? Other abuses that happen to you girls when you are at different places where you are staying?

P: Fighting.

I: Mmm.

P: Your friends provoking you and then swearing at you. Yeah.

I: Okay. What else?

P: That is all.

I: That is all?

P: Yes.

I: Alright. When you look at the places that you go to, whether to play, or to the other places you mentioned, the security in these places how secure is it? For you as a girl, do you feel that you are secured? Like are you taken care of? You are not in any type of danger?

P: Yes.

I: It is secured?

P: Yes.

I: Why do you think it is secured?

P: Because when we go to these places, there are also some older people who say “don’t disturb the children, they are doing their studies.”

I: Mmm.

P: Yes. So they don’t provoke us.

I: Alright. Now you as; I wanted to ask you as a girl, right?

P: Yes.

I: Is there anyone whom you feel comfortable with to tell everything? There are some other stories that you cannot talk with everyone. But there is that one person, or people that you just feel comfortable with.

P: No.

I: You don’t have anyone that you feel comfortable with? Whom you chat with on confidential matters? You don’t have?

P: Yes.

I: Alright. So I just wanted to know. You said that at the GG club they teach you about HIV. What were the others?

P: About visions.

I: Visions. What else?

P: About my dreams, about gender.

I: Okay. About gender?

P: Yes.

I: On the gender, I wanted to understand. About gender, what do they say?

P: Gender they taught us that “a girl when she works, cooking, a boy also cooks. Or a girl washing dishes, a boy also washes dishes.”

I: Mmh?

P: Yes.

I: Now the other thing I wanted to understand is that at the GG Club have you ever learnt about issues of contraception?

P: About Cholera?

I: Contraception.

P: Contaception?

I: Yes.

P: Yes they’ve taught us before.

I: Mmh?

P: Yes.

I: HIV, you have already mentioned about it. Now what about sexual intercourse?

P: They taught us that we can use condoms.

I: Okay. What else? On issues concerning sexual intercourse?

P: Prevention of sexually transmitted diseases and prevention of pregnancy.

I: Prevention of pregnancy?

P: Yes.

I: So I wanted to know that apart from the GG club, these issues like sexual intercourse, issues of contraception, contraception so that you do not get pregnant. Issues about prevention of sexually transmitted diseases. Issues about relationships among boys and girls, maybe being asked to be a love relationship partner. Issues like these, right?

P: Yes.

I: These issues, I wanted to know that as for you apart from the GG club, issues like these, who else do you discuss them with? Where you also hear such issues about HIV, issues of contraception……

P: At school they tell us.

I: At school they tell you?

P: Yes. Not at MM, but where I used to learn before they used to tell us.

I: Okay. Where else? Where else do they tell you?

P: At the GGs there.

I: Right there at the GGs?

P: Yes. Sometimes when we go for a chat at our friends’ place they tell us about that.

I: Your friends?

P: Yes.

I: You are able to discuss with your friends?

P: Yes.

I: What about in this village, are there some people whom their job is to advice girls about these issues?

P: Yes.

I: There are?

P: Yes.

I: Who are they, who advice you on these issues?

P: Who advices us?

I: Yes.

P: Mrs Wisikisi.

I: Mrs Wisikisi?

P: Yes.

I: She advices you on these issues?

P: Okay.

I: What does she say?

P: She says we should not have sexual intercourse with boys. “Or else you will get pregnant or get sexually transmitted diseases.”

I: What time does she advice you? Does she go door to door visiting every girl or how does it happen?

P: She does not visit us at our homes, No. But the day we went there to learn, we also discussed about these issues. She talked about them to us.

I: How did you go there? Was there a gathering?

P: No. We just went to learn.

I: You went to learn?

P: Yes. About school stuff.

I: About school?

P: Yes. Afternoon schooling.

I: Oooh! Okay. So I just wanted you to explain to me, if it was to happen to you; just imagining, right; not that it has really happened to you.

P: Mmm.

I: Just imagining that you have a boyfriend, you are having sexual intercourse. And you have been found with sexually transmitted diseases. That matter, whom would you tell?

P: I would tell my mother; I can tell her. And also our teachers at GGs club I can tell them.

I: Why would you tell your mother? Why have you chosen to tell it to your mother or to tell your teacher at GGs club?

P: Or maybe….[breaths]….

I: Why would you tell your mother? Not that it is wrong to tell her but I just want to know why you have chosen to tell it to your mother? I just want to know.

P: So that maybe she should know that my child, this has happened to her.

I: What if you are found to be pregnant?

P: Telling, I would tell my mother.

I: You would also tell it to your mother?

P: Yes.

I: Alright. What about on issues of love relationships? Maybe a boy has asked you to be his girlfriend, whom do you discuss them with?

P: Me?

I: Yes. Has a boy ever asked you to be his girlfriend?

P: Yes. But at the places where we go for chats.

I: Mmh? They ask you to be their girlfriend?

P: Yes. So we swore at them, together with my friend from there.

I: Okay. Boys want you? What do they say?

P: They say “I want you to be my girlfriend.” So I say don’t kill our future because we are still studying.”

I: Alright. So these issues about love relationships, whom do you tell them to? When they ask you to be their girlfriend like that, who else do you tell it to?

P: My mother or that my friend. And she tells me “don’t agree to that, it is bad. Continue with your studies.”

I: Alright. Now in this village, girls; are there girls who are facing different problems on these issues maybe on issues of sexual intercourse?

P: Yes there are. Many of them.

I: So their problems, girls like these, where do they take them to? Where do they tell them to? When they are faced with such? Maybe they have been found pregnant?

P: On that I don’t really know.

I: You don’t really know?

P: Yes.

I: Alright. Do you have monthly periods?

P: Yes.

I: You have monthly periods?

P: Yes.

I: When you started having monthly periods, whom did you tell it to?

P: I told my mother.

I: Okay. So what did she say?

P: She taught me to wear sanitary pads during the periods.

I: Then, what else happened?

P: She told me that, don’t walk around unnecessarily, don’t have sexual intercourse with boys. If you have sexual intercourse with boys you will get pregnant.”

I: Your mother said that?

P: Yes.

I: What about other counseling’s that you received at that time, where did it come from?

P: The one who gave me my name also counseled me.

I: Who is that?

P: Someone from down the road there.

I: Who is this person to you?

P: My aunt; the one who named me.

I: You were taken to your aunt?

P: Yes. And I was advised there.

I: So at your aunt’s house did you talk about issues of HIV?

P: No.

I: You did not talk about HIV?

P: Yes.

I: She just talked to you about what? I want to know, explain to me what your aunt told you.

P: She said that,” now that you are having monthly periods, don’t just walk around unnecessarily, don’t have sexual intercourse with boys. If you have sexual intercourse with boys you will get pregnant. Your used sanitary pads and all your belongings you should take care of them. If you don’t, people will use them for rituals.”

I: Okay. What about at the girls initiation ceremony, did you go?

P: Girls initiation ceremony?

I: Okay.

P: Yes I went.

I: Okay. I just wanted to know that there at the girls initiation ceremony, issues of HIV were they discussed?

P: I went there when I was a child so I would not really know.

I: You went there when you when you were child?

P: Yes.

I: Alright. I thought you go there when you start having your monthly periods, isn’t it so?

P: That kind of initiation ceremony has never happened here.

I: Alright. Which religion do you belong to?

P: Religion?

I: Yes.

P: Islam.

I: Islam?

P: Okay.

I: What about at the mosque, do you talk about issues of HIV?

P: No.

I: They don’t talk about it?

P: Yes.

I: What about issues of contraception for girls?

P: They don’t talk about it.

I: About sexual intercourse between boys and girls?

P: They don’t talk about it.

I: They don’t talk about it?

P: Yes.

I: Alright. So I now want to ask, is your father alive?

P: Yes.

I: You father is alive?

P: Yes.

I: Your father, has he ever talked to you about issues of HIV?

P: Yes. He has ever talked about it.

I: He talked about it? What did he say, your father?

P: He told me that it is a disease that affects people. That when you are found with the AIDS disease, you are likely to die. Or if you do not take drugs you are likely to die.

I: Okay. Anything else that you learnt from your father?

P: He taught me that “don’t walk around unnecessarily, even if you are among your fellow GG club friends don’t walk around unnecessarily, just stay right there and chat with your friends.”

I: What about your mother?

P: She also taught me the same thing.

I: Alright. Now you as a girl who is growing in this village, what other problems do you face on issues like these? We have talked about sexual intercourse, relationship issues. What problems do you face?

P: Problems?

I: Yes.

P: I don’t face any kind of problems.

I: What about questions that you have concerning these issues, do you have any questions?

P: When ER comes, we ask her.

I: Whom do you ask?

P: ER. When she comes to our club.

I: Who is ER?

P: Someone. She visits us. She comes from Blantyre.

I: Mmh?

P: Yes. She tells us about HIV. So we ask “what is HIV?” and she tells us, “HIV is a disease that arouses when there is not enough protection in the body.”

I: Okay. So I also wanted to know that when did you join the GG Club?

P: I joined in 2016.

I: Okay. What is it that made you to join GG Club?

P: What made me join the Club is that, we went to NK, there was a gathering there that all children should go and attend. And they told us that, “choose a leader among yourselves.” We chose IN and EM to be the ones supervising us. It then happened that before a month, they were called for training, they learnt there and then came and taught us. Now us, many people were discouraging us saying “don’t go there, it is satanic.” But us, we did not care, we went there so that we should learn. So what made me to join the GG club is learning.

I: So what have you benefited from the GG Club?

P: Me, Through GG Club, village banks were introduced in our village, my mother managed to join the village bank. She borrowed money from there and do business. The profits from the business are used to get me necessities for my school.

I: Alright. What about you, what have you benefited?

P: Me?

I: Yes.

P: I have benefited from GG Club that I did not really know the reason for my going to school, but I have known it. Through GG Club I have learnt that it is up to you to choose which job you want after finishing school. So I was convinced, I went to continue with school.

I: Mmh?

P: I have known the main reason of going to school.

I: Alright. If your friend wanted to join the GG Club, would you tell her to join the GG Club?

P: Me?

I: Yes.

P: I would not be able to tell her because; maybe if it was that she was not shaming us, right?

I: Yes.

P: Or maybe tell her that if she likes. But again, they said that we should not exceed 25 members. No one else should come and join.

I: Alright. You, sometime back you were not going to school?

P: Yes.

I: Why were you not going to school?

P: Me?

I: Yes.

P: I was lacking necessities for my school.

I: Like what?

P: Notebooks, uniform. When I go to school, I was sent back home. Washing clothes was difficult, there was no soap, no body lotion. Yeah.

I: So what is it that made you to go back to school?

P: My mother joined the village bank she went and borrowed money and started doing business to help provide me with my school necessities.

I: Alright. Now I want to talk about the relationships that are there among boys and girls in this village. What are your views on this issue, that a boy should be involved in a love relationship with a girl?

P: Me, my views?

I: Yes.

P: My views are that it is not right that a girl should be involved in a love relationship with a boy because it is killing her future.

I: Who is killing the others future?

P: The boy is killing the future of the girl.

I: Why? How would he kill the future?

P: Because for example like me, the girl is schooling and the boy asks her to be his girlfriend, he will get her pregnant. She would not go to school while pregnant.

I: Alright. When you are with your friends, do you discuss about love relationships like these?

P: We don’t discuss them.

I: You don’t discuss them?

P: Yes.

I: Why don’t you discuss them?

P: [silence]

I: When you have been asked by a boy for a love relationship, don’t you discuss it?

P: We discuss it. Saying “my fried, such a boy has asked to be his girlfriend. What should we do to him?” so she says “let us just talk to him so that he should not do it again to us.”

I: Now issues like these, concerning love relationships among boys and girls, whom would you love to discuss them with?

P: Me?

I: Yes. Aren’t there times, for you as a girl who is growing you would have questions?

P: Yes.

I: What would you want to know about. For example, what would you like to know?

P: I would discuss with my friend not a boy. I would discuss with my fellow girl.

I: But what views do you have when you think about relationships like these? What goes on in your head? What do you think about? I know that there are views in this head concerning issues like these. Am I lying?

P: No.

I: Am I lying?

P: Yes. Because I would not think about boys because I am still studying.

I: You don’t think about it?

P: Yes.

I: Alright. Okay then, the questions that I had for you I think we have discussed them. They were the only ones. Thank you for your answers to these questions.

P: Mmm.

I: Maybe you have a question?

P: No. I don’t have a question.

I: Thank you very much because of your answering to these questions.
