# Supplementary material for: Social support and very young adolescent girl’s knowledge on sexual relationships: A comparative qualitative study of Girl Only Clubs’ participants and non-participants in rural Malawi
Source: PLOS Glob Public Health. 2023 Jan 12;3(1):e0001339. doi: 10.1371/journal.pgph.0001339 (PMC10022037; doi:10.1371/journal.pgph.0001339)
Supplement: S1 Data — (ZIP) [file pgph.0001339.s003.zip › Anonymized/IDI12_R2_YY14_Apr.docx]

District: YY

Location: A

IDI ID: IDI1_R2_YY14_May2019

Interviewer: WWW

Data collection date: DD/MM/2019

**Interviewer:** Like I already explained at the beginning that my name is WWW and I have come from the COM. We are conducting this research together with OC and PP of the USA. In this survey we are talking to different girls who joined the GGs Clubs, especially adolescent girls that are aged between 10 and 14 years old. We want to know what your experiences have been like during the time that you have been participating in the OC clubs and we are also interested in knowing how girls of your ages in this village and the surrounding villages learn about issues to do with HIV and how they can protect themselves from getting pregnant as well as other issues that affect their health. We just want to know how you know about such issues. Whether you know these issues from the clubs or maybe there are other ways you know about these issues, is it through the mother groups or through the radio? So I want you to tell me how you know about these issues or where you learn these issues from. So I want you to be relaxed, there is no response that you can tell me that is wrong. Any response you give me is an answer to me. The first thing that I want to ask you is that are you currently in school?

**Participant:** Yes.

**Interviewer:** Where do you learn?

**Participant:** At CA.

**Interviewer:** Which class are you in?

**Participant:** I am I standard 5.

**Interviewer:** Have you at one time ever dropped out of school?

**Participant:** Yes.

**Interviewer:** Why did you drop out of school?

**Participant:** I just dropped out of school because I wanted to and a certain friend of mine invited me to join the GGs Club so I joined…

**Interviewer:** You just dropped out because you wanted to drop out,

**Participant:** Yes.

**I:** what caused this to happen in order for you to drop out of school? What made you to no longer want to go to school?

**P:** There was no reason at all, I just wanted to!

**I:** Oh?

**P:** Yes.

**I:**  You just wanted to stay at home?

**P:** *(Chucking.)*

**I:** You just wanted to stay at home?

**P:** Yes.

**I:** What made you want to just stay at home?

**P:** There was nothing that caused me to just stay at home.

**I:** Uhmm, so long did you stay at home for? What class did you drop out from?

**P:** When I was in still in Standard 5.

**I:** Uhmm. So how long did you stay at home for?

**P:** I dropped out when people were about to receive the results of their exams, in the last term that is when I dropped out.

**I:** Okay, now you have returned back to school?

**P:** Yes.

**I:** Uhmm. So when you think of your future, what do you think about your future; what do you want your future to be like?

**P:** *(Silence).*

**I:** What is it you want to do in the future?

**P:** I want to be a nurse.

**I:** Uhmm…

**P:** I want to be injecting people.

**I:** Uhmm, why do you want to become a nurse?

**P:** The reason is because there is a nurse there(at some house in the village) who I find interesting.

**I:** Okay. Alright. When you are not some household chores, like mopping the house, sweeping, fetching water, cooking; the time that you have finished all your chores, what is it you like to do?

**P:** I like to read at home.

**I:** You like to read?

**P:** Yes.

**I:** Okay, what else do you like to do? Where is it you like to go when you are not reading, where do you like to go? Where do you go to chat?

**P:** I like going to visit my friends.

**I:** Uhmm, where are your friends from?

**P:** One lives over there, yes…

**I:** Where else do you like to go?

**P:** I only go there to chat…

**I:** You only visit your friends, there is nowhere else where girls go in this village?

**P:** *(Silence.)*

**I:** So you are either at home or school or only visiting your friends, is there nowhere else you go as girls?

**P:** In the evenings when we want to walk about when my friend has been sent that is when I escort her.

**I:** Okay, what are the challenges that the girls in this area face? Maybe when the girls have gone somewhere just to walk about? I know sometimes unfortunate things can happen to girls, does it happen here?

**P:** Some girls face violence such as having their breast touched.

**I:** Uhmm…

**P:** Yes.

**I:** How often do such things happen here?

**P:** It happens but it is not very often. Maybe you will be walking then a boy just runs at you and touches your breast.

**I:** Uhmm.

**P:** Yes.

**I:** Has something like this ever happened to you?

**P:** For me it has never happened to me before.

**I:** So when something like this happens to the girls where do the girls go to report such issues?

**P:** They report to the mother-groups or maybe to the teachers and also maybe to the police.

**I:** Okay, alright. So here at home who do you live with?

**P:** I live with my mother and my father.

**I:** Your mother and your father?

**P:** Yes.

**I:** Okay, in order to find food for the household, so that food should be available as well as soap and such other items, what does your father do in order to find these things?

**P:** My father does some piece works.

**I:** He does some piece works.

**P:** Yes.

**I:** What about your mother?

**P:** On such issues my mother just stays idle.

**I:** She just stays idle?

**P:** Yes.

**I:** Okay, when it comes to issues of HIV, where did you hear such issues from for the first time? For you to hear…like I explained that we want to know where the adolescent girls here hear issues about HIV. So we wanted to know where you head such issues from for the first time.

**P:** Sometimes we here from…sometimes we here from…

**I:** Where you heard such issues for the first time? The first time you heard about HIV issues, where did you hear these issue from?

**P:** The first time I heard it from school.

**I:** You heard it from school?

**P:** Yes.

**I:** Who was talking about these issues at school?

**P:** Our teacher…

**I:** In the classroom?

**P:** Yes.

**I:** What did they say HIV was?

**P:** They said that HIV is a dangerous virus…HIV…

**I:** Uhmm. What else did they say that you learnt?

**P:** They said that we should avoid AIDS…

**I:** How did they say you should avoid it?

**P:** We should not have sex with men…we should not engage in sexual relationships. Yes.

**I:** Uhmm…

**P:** Yes.

**I:** Alright, where else have you heard about HIV issues?

**P:** Another place where I have heard about these issues is at the GGs club; they teach us about preventing HIV. Yes.

**I:** Where else?

**P:** Those are the only places.

**I:** So you have told me that you first heard it from the school and then you also heard about it at the GGs club?

**P:** Yes.

**I:** So I want you to tell me how you differentiate the information that you heard from the school through your teacher and the information that you heard through the GGs club? How is it different?

**P:** *(Silence.)*

**I:** Or how did it change you?

**P:** It changed me because through the teachers they taught us how to avoid it…

**I:** I want you to tell me the difference; how does it differ from what you heard at the school and what you were taught through OC?

**P:** The difference is that at this other place they hide some of the issues from us while at the GGs club they tell us everything.

**I:** Uhmm. Like what is it that they hid from you at school?

**P:** About how to avoid engaging in sexual intercourse, some of the issues they hide from us…yes.

**I:** Why do you think they hide some of the issues from you?

**P:** Maybe the teachers hide these issues from us because we are still young that is why they chose to hide the issues from us…

**I:** *(Chuckling.)* Okay, what about issues to do with avoiding getting pregnant. Where did you hear these issues for the first time from?

**P:** On how to avoid getting pregnant, I heard it from the GGs club and I also heard about it from the school.

**I:** What did they say about how you can avoid getting pregnant?

**P:** They said that you can avoid getting pregnant by…after your periods if you…just a day after finishing your periods if you engage in sexual intercourse with a man then you might get pregnant. *(Atha kumakuphonya-meaning you can miss your period)*

**I:** Meaning that you might get pregnant as a result?

**P:** Yes.

**I:** Alright, I wanted to know what you experienced the time when you started your menstrual periods, before you started your menstrual periods did you talk to anyone about these issues. Or were you given any type of advice related to menstruation? Maybe from your parents…

**P:** I was told by my mother and my friend was also talking about this.

**I:** What did they say? that is before you started, we are talking about the time when you had not started…?

**P:** *(Speaking with a stammer so audio not audible for about 5 seconds)…*they were saying that if a person is menstruating blood comes out from them.

**I:** So they told you about this when you had not started your menstrual periods?

**P:** Yes.

**I:** So when it started, on the day that you started your menstrual periods who did you tell?

**P:** The day that it started I just told my friend.

**I:** You told your friend?

**P:** Yes.

**I:** Why did you chose to tell your friend?

**P:** *(While chuckling and speaking with a stammer)* it is because I could not tell anyone else because I felt embarrassed.

**I:** Okay, so who is it that told the adults?

**P:** Who told the adults…?

**I:** How did your parents find out? Or how did the adults find out that you have started to menstruate?

**P:** The friend that I told, when I told her she is the one who then told my mother?

**I:** Uhmm, so what did your mother say?

**P:** My mother just said that okay.

**I:** So who gave you advice? About what you should be doing; that you should be doing this and that…or this is what it means?

**P:** So my mother told my friend to explain it to me…

**I:** So it was your friend who advised you?

**P:** Yes.

**I:** Did your parents not find people to advise you? Or did they not advice you themselves?

**P:** Not other people *(Stammering)* but my mother she was also able to advise me.

**I:** Uhmm, okay. How often is it in this village for a girl and a girl to be in a relationship? Girls that are of your age…

**P:** In this village it…it is very common.

**I:** It is very common?

**P:** Yes.

**I:** What makes girls to engage in relationships with boys? What causes them to engage in these relationships…what is it that they want?

**P:** On that I do not know.

**I:** You do not know?

**P:** Yes.

**I:** Okay. Have you ever talked to anyone about wanting to know relationships that exist between boys and girls?

**P:** Yes.

**I:** Who did you talk to?

**P:** My friend?

**I:** What is it that you were talking about?

**P:** *(Stammering) (Inaudible speech for about 15 seconds)* she said that it is not good to engage in such relationships.

**I:** Uhmm.

**P:** Yes.

**I:** Okay, what was the reason that caused you to join the DREAMS GGs club?

**P:** (Speaking with a stammer) I wanted…I wanted to know about a lot of things…

**I:** Uhmm, okay, so how did you join? How did you know that there are groups like these? *(Clearing throat)* Who invited you to join this group? How were chosen?

**P:** The chief!

**I:** Uhmm. Is he the one who called for you girls?

**P:** Yes.

**I:** Okay. What if there is another girl that wants to join this group *(Yawning)* what advice can you give that girl? How can you encourage them?

**P:** *(Stammering)* I can encourage the girl that…I can encourage her that in the GGs club there are a lot of things…a lot of things that can…a lot of things that can encourage her…

**I:** Uhmm. Thank you very much for your responses to these questions. Do you have any questions?

**P:** No I do not have any question.

**I:** Thank you very Much.

***End of interview.***
