# Supplementary material for: Social support and very young adolescent girl’s knowledge on sexual relationships: A comparative qualitative study of Girl Only Clubs’ participants and non-participants in rural Malawi
Source: PLOS Glob Public Health. 2023 Jan 12;3(1):e0001339. doi: 10.1371/journal.pgph.0001339 (PMC10022037; doi:10.1371/journal.pgph.0001339)
Supplement: S1 Data — (ZIP) [file pgph.0001339.s003.zip › Anonymized/IDI13_R1_YY15_May.docx]

**INDEPTH INTERVIEWS WITH 10-14 YEAR OLD GIRLS**

District: YY

IDI ID: IDI3_R1_YY15_May2019

Location: A

Interviewer: WWW

Data collection date: DD/MM/2019

I: Now first of all, I wanted to know that; for you, in this village as you live on each and every day what do you like to do?

P: Me, as I live here each and every day…...

I: Yes

P: I like reading school stuff.

I: You like reading school stuff?

P: Yes. And also I like reading what our teacher teaches us at GGs.

I: Mmh?

P: Yes.

I: When you wake up in the morning, what do you do?

P: When I wake up in the morning, I first of all go and draw water, then wash the dishes and sweep the yard.

I: Alright. What else? When you are done, what do you do?

P: When I am done, I just stay.

I: What do you do?

P: I just stay.

I: You just stay?

P: Yes

I: Then?

P: Then when it is now 12 o’clock my mother cooks nsima and we eat.

I: Mmh?

P: Yes.

I: Alright. Now in the afternoon what do you do?

P: In the afternoon I I go to the GGs to learn.

I: Every day?

P: No. each and every Saturday

I: Alright. What about on the other days?

P: Nothing on the other days.

I: What do you in the afternoons?

P: In the afternoon I cook Nsima; we eat.

I: When you eat Nsima, you’ve washed the dishes, what do you do?

P: We either play netball.

I: Alright. What else?

P: We either play with dolls.

I: Oooh! You play with dolls; together with your friends?

P: Yes.

I: Alright. Where else do you like to go apart from where you play netball?

P: What I like, I go to chat at my friends’.

I: Alright. So in all these places where you like to go, when you think of your security as a girl, how secured do you see yourself there?

P: My security, I see myself as secured?

I: Why is that?

P: Because I do physical exercises.

I: Alright. Are there no any a dangerous things? Or maybe facing dangers or hindrances as girls?

P: No.

I: Alright. Now you, who do you like to chat with?

P: Me?

I: Yes.

P: I like to chat with..[breathes]…

I: The person whom you feel comfortable with

P: Oooh!

I: Whom do you feel comfortable with?

P: MM.

I: Mmh?

P: Yes.

I: That is your best friend?

P: Yes.

I: With MM, what stories do you like to chat about?

P: We just like to chat, just chatting.

I: Stories like what?

P: Stories like about….like about reading.

I: Mmh?

P: Yes.

I: Alright. Now I wanted to know, umm; on issues concerning HIV, what do you know?

P: HIV?

I: Yes.

P: Iiih! I don’t know anything.

I: You don’t know anything?

P: Yes.

I: At the GG club they’ve never told you about HIV?

P: They told us.

I: What did they say?

P: They said that HIV is a virus that infects in the body when protection is low.

I: Okay. What does it infect.

P: The virus.

I: Alright. What else?

P: They said that; they talked of AIDS. When they talked of AIDS, they said that AIDS is a disorder of a disease that starts when protection is low. And they told us about prevention of AIDS.

I: Mmm.

P: Abstinence

I: Mmm.

P: Using a condom.

I: Alright. These issues like abstinence, using a condom; have you ever discussed them with your parents?

P: Yes.

I: What do they say?

P: They said that “abstain. Don’t have sexual intercourse with boys. No.”

I: Is that your mother or your father?

P: My mother.

I: Okay. What about your father?

P: My father said “abstain,” just the same things.

I: Alright. Now do you have an Uncle?

P: Yes.

I: What about Aunties, do you have?

P: Who?

I: An aunt.

P: No.

I: You don’t have?

P: Yes.

I: These people, have you ever discussed with them before issues like these?

P: Yes.

I: Okay. What do they say?

P: They say the same things that “you should not have sexual intercourse with boys.”

I: Mmh?

P: Yes.

I: Alright. What about with your friends in this village?

P: My friends also tell me the same thing that I should continue with GGs they’ll teach me good things. “Don’t have sexual intercourse with boys.”

I: Mmh?

P: Yes.

I: Alright. What about in this village. Where young people like you; like young girls like you, were you can go to seek advice on issues of HIV or that you should not have sexual intercourse with boys or if maybe someone has been found with sexually transmitted diseases. Where young girls like you can go and seek counseling or help, where is it?

P: At MTA. At the doctors at MTA maybe.

I: Alright. Where else? What about in this village?

P: In this village?

I: Yes. Where they can seek counseling or advice on issues of HIV? Or about sexual intercourse?

P: At the Chiefs house.

I: At the Chiefs house?

P: Yes.

I: The Chief also gives advice?

P: No. but Non Governmental Organizations.

I: Non Governmental Organizations?

P: Yes.

I: In this village there are Non Governmental Organizations?

P: Yes.

I: Do you know where the Non Governmental Organizations are? Where people can go?

P: That house there.

I: Mmh?

P: Yes.

I: What about you? Have you ever gone there to seek counseling

P: No.

I: Why?

P: Nothing.

I: Okay. Now just imagining, if it was that you have been faced with a problem. For example maybe you have been found pregnant. Whom would you tell?

P: I would tell my teacher at OC.

I: Why?

P: What?

I: Why would you choose to tell your teacher?

P: There is no reason.

I: There is no reason? You would just tell it to your teacher at OC?

P: Yes. So that maybe they should help me.

I: Mmh?

P: Yes.

I: What should they help you with?

P: [Silence]

I: What if you were found with sexually transmitted diseases, where would you seek help?

P: At the hospital.

I: At the hospital?

P: Yes.

I: Which other person would you tell?

P: My mother.

I: Alright. What if you had different questions on things that you wanted to know about, maybe issues of HIV/AIDS or about sexually transmitted diseases. If you had questions and you wanted to find answers to them, whom would you ask?

P: I would ask my teacher at GGs.

I: Mmh?

P: Yes.

I: Alright. Apart from the GGs, where else would you ask questions? Or where else do girls go and ask questions?

P: [Silence]

I: In this village?

P: At Non Governmental Organizations.

I: What?

P: Non Governmental Organizations.

I: Non Governmental Organizations?

P: Yes.

I: In this village there are other Non Governmental Organizations?

P: Yes.

I: Where girls go?

P: There at the Non Governmental Organizations.

I: There are Non Governmental Organizations?

P: Yes.

I: Okay. So I’ve seen that you live here right?

P: Yes.

I: You like living here?

P: Yes.

I: Why do you like living here?

P: I wanted to be chatting with my mum.

I: Mmh?

P: Yes.

I: Alright. How secured in this place.

P: Security?

I: Yes. When you are here at your home do you feel secured?

P: Yes

I: Why?

P: Because….[silence]

I: Who else do you live with? Here in this house, who else lives here?

P: In this house?

I: Yes. Whom do you live with?

P: My mother and my father.

I: Who else?

P: GA.

I: What?

P: GA.

I: Your sister?

P: My younger sister?

I: You are the first born?

P: Yes.

I: Alright. Apart from the GG Club, these issues of HIV, have you ever discussed them with anyone else before?

P: No.

I: Alright. Now I want to ask you about relationships that takes place between girls and boys. Or love relationships, right?

P: Yes.

I: What are your views on that boys and girls should be in love relationships?

P: [Silence]

I: Is it good that Girls should be involved in love relationships?

P: No.

I: Why is that?

P: Because they can contract HIV.

I: Mmh?

P: Yes.

I: What else?

P: Maybe the girls they can get pregnant.

I: Mmh?

P: Yes.

I: So these issues do you discuss them with your friends whom you chat with?

P: No. I don’t discuss with them.

I: Why not?

P: Nothing.

I: So whom do you discuss them with? From where do know these things?

P: About HIV?

I: Yes.

P: I knew about it through Banja La Mtsogolo; the OC People who came to test us for HIV.

I: Mmh?

P: Yes.

I: Alright. Now we should talk about other girls, young girls like young girls like you in this village. Are there your fellow friends, young girls like you who are married?

P: Yes.

I: There are?

P: Yes.

I: Why is it that….. Why do you think they got married while they are still young? What is it that makes girls to get married while they are still young?

P: What makes them to get married while they are still young?

I: Yes.

P: Maybe their mother did not advice them well. So they got married while they are still young.

I: Mmh?

P: Yes. But they were also having sexual intercourse with boys. They did not listen to their

I: Mmh?

P: Yes.

I: Alright. What else?

P: Maybe they wanted money. That the boys should give them.

I: What kind of men do they get married to? Or what kind of men?

P: Whom do they get married to?

I: Yes. What kind of husbands do they have?

P: [Silence]

I: When I say what kind of men, I mean that are they business men, or teachers or what kind of men?

P: Ooh! They are business men.

I: Alright. Who else?

P: Some do business.

I: Mmh?

P: Yes.

I: What are your views on the issue that girls should be found that they have gotten married while they are still young? Is it good?

P: No it is not good.

I: Why is that?

P: Because they have killed their own future. Instead of going to school, they are busy with marriages

I: Alright. So I heard that at first you were not going to school?

P: Yes.

I: Why were you not going to school?

P: The reason why I was not going to school?

I: Yes.

P: I was lacking school uniform, school necessities; notebooks. I was lacking those.

I: Alright. So now you are back in school?

P: Yes. I am back in school.

I: Who is helping you in schooling?

P: Who is helping me?

I: Yes. I thought you said at first you were lacking notebooks and others?

P: Yes.

I: So right now, who is helping you?

P: Then my mother joined village bank there, and started borrowing money. So she bought me a school uniform. Then I started going to school.

I: Alright. Now I want to ask about the program of dreams; of the GG Club. How did you know about the GG Club? How were you found in the GG Club?

P: How I was found in the GG Club?

I: Yes.

P: The teacher said “come here and join the group of GGs.”

I: Who?

P: Our teacher who teaches us.

I: She came here?

P: No. she just said it at her home that “those who would like to join should come.”

I: Where did she say this?

P: At her home?

I: Where is here home?

P: There. While we were drawing water. So she said “those who want to join GGs.” Some people refused. And we said, “us, we would like to join.” So we joined.

I: Mmh?

P: Yes.

I: So what is its importance?

P: The importance of GGs?

I: Yes.

P: For example, I had stopped going to school. But when I started learning at GGs, I continued with school and they encouraged us about school. And also I learnt about my dreams that “it is up to you to decide which job to work in the future when you finish school.” So I would like that when do my school and finish, I should be a supervisor in the OC.

I: You want to be a supervisor in the OC?

P: Yes.

I: You should work right here?

P: Yes.

I: Alright. What about what you can tell other girls about GG Club; Those that have not yet joined GG Club; the other girls, what can you tell them?

P: Me?

I: Yes.

P: I cannot tell them anything because they were laughing at us saying “what are you going there to do?” so I would not tell them anything.

I: They laugh at you?

P: Yes.

I: What do they say?

P: That time we were going they used to say you are busy going to GG Club but you don’t receive anything there.” So we did not pay them attention. We just went.

I: They laugh at you?

P: Yes.

I: Saying you don’t receive anything?

P: Yes. But since last time when we received school bags they stopped laughing.

I: Mmh?

P: Yes.

I: Alright. Thank you very much for your answering to these questions. Maybe you have a question?

P: No I don’t have a question.

I: You don’t have a question?

P: Yes.

I: Alright. Thank you very much that you have answered these questions well, right?

P: Yes.

I: Thank you.
