# Supplementary material for: Social support and very young adolescent girl’s knowledge on sexual relationships: A comparative qualitative study of Girl Only Clubs’ participants and non-participants in rural Malawi
Source: PLOS Glob Public Health. 2023 Jan 12;3(1):e0001339. doi: 10.1371/journal.pgph.0001339 (PMC10022037; doi:10.1371/journal.pgph.0001339)
Supplement: S1 Data — (ZIP) [file pgph.0001339.s003.zip › Anonymized/IDI17_R1_XX8_May.docx]

**INDEPTH INTERVIEWS WITH 10-14 YEAR OLD GIRLS**

District: XX

IDI ID: IDI9_R1_XX8_May2019

Location: B

Interviewer: WWW

Data collection date: DD/MM/2019

I: Okay, so the first question I wanted to know that you as a girl in this village as you live, here at your home, how does your day go like? When you wake up in the morning? Or maybe up until the afternoon, what do you do? Whom do you chat with? And also what do you do? How the whole of your day goes like? As the first question right?

P: My name is XXX. I am fourteen years old.

I: Yes,

P: In my daily living, sometimes I face problems.

I: Yes,

P: Problems like maybe at my home they shouted at me. And also sometimes I am happy.

I: Yes,

P: My friend whom I chat with most of the times is CJ

I: Okay. How many children are you at your home?

P: My mother left behind three children.

I: Okay. Your mother passed away?

P: Yes.

I: But you live with your father?

P: My grandmother.

I: This is your grandmother’s house?

P: Yes.

I: Alright. So here at your grandmother’s house, you live with whom and whom? Just tell me how many you are in this home.

P: At my home there is SK, MX, ML, JX, WN, YS” who else?”and me; AX.

I: Okay. Now tell me in your day; when it is morning, what do you do?

P: When it is morning, I wake up; sweep the yard, after I sweep the yard, maybe I’ll sweep inside the house. I’ll then go draw water and wash the dishes.

I: Okay.

P: When it is that I am going to school, sometimes I go to school, then I’ll go to school.

I: Okay. So as you live; like how you live in this home. Whom do you feel comfortable with?

P: I feel comfortable with ML.

I: Okay. Who is ML to you?

P: My Aunt.

I: Your Aunt?

P: Yes.

I: Okay. So what stories do you talk about?

P: Whether just about encouraging me. And making me not to feel sad.

I: What do you feel sad about?

P: My grandmother sometimes talks. She reminds me of my mum, right?

I: Yes,

P: So most times I feel sad. Sometimes she refuses to buy me notebooks, and others. So all the times I am always very sad.

I: When did your mother pass away?

P: Iiih, while I was young.

I: Okay.

P: She passed while my younger sister was two months old.

I: Alright. Now I wanted us to talk about; for example issues like about HIV and AIDS. Have you ever heard about issues of HIV and AIDS?

P: Yes.

I: Where did you hear about them?

P: I heard about them at our group; the one we do.

I: Your group of OC?

P: Yes.

I: Okay. There, what do you say HIV and AIDS is?

P: HIV/AIDS is a disorder of many diseases.

I: Alright. Anything else that you learnt about HIV? You just learnt that HIV is a disorder of many diseases?

P: No.

I: Or what else did you learn concerning the disease of HIV?

P: Umm, on that I have forgotten.

I: On this you have forgotten?

P: Yes.

I: You just know that it is a disorder of diseases?

P: Yes.

I: Alright. Apart from OC, where else do you as a girl; able to hear stories of HIV? Where is it?

P: Maybe at hospitals.

I: Yes,

P: Sometimes when we go to the market we are able to find comedies, people talking about HIV and AIDS.

I: At the hospital you are able to go?

P: Yes.

I: To hear these kinds of messages?

P: Maybe when I go when I am sick, I am able to hear people talking. Sometimes we read in the posters.

I: Okay. Now in this your village, where girls can go and hear about the disease of HIV/AIDS where is it?

P: In this our village I don’t know.

I: Okay.

P: Maybe it is only this group that we have started.

I: Only this group you have started?

P: Yes.

I: Alright. What about with your Aunt, as you said. You said ML, right?

P: Yes.

I: Have you ever discussed issues concerning HIV and AIDS?

P: Yes.

I: You were discussing based on what? What were you discussing?

P: She asked me “do you have a boyfriend?”

I: Yes,

P: I answered her “yes.” she said “but be careful, there are sexually transmitted diseases like HIV and AIDS. So to prevent, you can use condoms.” So I responded, “Okay.” “And also you need to go and get tested, you should know your status.” So we got together and went with her.

I: You got together with your Aunt and went for HIV testing?

P: Yes.

I: Where did you go for testing?

P: At the hospital.

I: Okay. Now being that you are a young girl, how did they welcome you at the hospital?

P: At the hospital when we got there, we stood on a line, right?

I: Yes,

P: When we got there, then they welcomed us warmly that we should feel free. They sang different songs. And then they took our cards and started calling person by person. When they called me in there, I went, they injected me and then I came out, right?

I: Yes,

P: Now when the results were out, my aunt went. When my aunt went, it happened that they found me negative of HIV and they told her. Then they came to me and said, “are you ready to hear your results?” I responded, “yes I am ready.”

I: Yes,

P: And then they took a long time. When they took a long time they said “don’t be sad. We have found you negative of HIV.” Then right there they started encouraging me so that my life should continue to be protected. And we came back home

I: Okay. What about other messages, like on the part concerning protection from getting early pregnancies, or protecting yourself from sexually transmitted diseases. These kinds of issues, where do you hear them from? As a girl?

P: Me, most of these I am hearing them from this same group that I have joined.

I: Okay. What about in this village, isn’t there somewhere where you discuss? Or even with your friends? Discussing issues like these?

P: With my friend CJ, we are able to discuss.

I: What do you discuss about?

P: That prevention of pregnancy maybe using condoms. There are other girls who go and get contraceptives.

I: Mmh? Where do they go and get them?

P: At the hospital.

I: Okay. Where else? Maybe you discussed with your aunt? Issues like these, about prevention of pregnancy.

P: Umm, with her, we have never discussed this.

I: Okay. What about with your grandmother?

P: [Laughs]

I: With your grandmother, have you never discussed issues about HIV and AIDS? You live with your grandmother, right?

P: Yes. I have never discussed with her before.

I: What about these other issues? The one’s I have explained?

P: On that she just says “don’t do what? Don’t have sexual intercourse with boys. In this world there are sexually transmitted diseases. If you want to be doing that, maybe you should be using condoms.” And also her, she does not want this kind of behavior.

I: Okay. Now what about at the time that you started having monthly periods, whom did you tell?

P: I told my mother’s older sister?

I: You told your mothers older sister?

P: Yes.

I: Why did you choose to tell her?

P: She is the one I saw that she can help me.

I: Okay.So what kind of counseling did you receive?

P: She told me that I should avoid sexual intercourse.

I: Yes,

P: And also she taught me how to wear sanitary pads. And other things.

I: Okay. Now these issues I have talked about are interesting issues. Issues like those of HIV/AIDS, issues of preventing sexually transmitted diseases, prevention of early pregnancies. They are issues that are very important to girls like you. Youths like you. Now my question is that; for you, issues like these, whom would you love to discuss them with? Or who should talk about them to you? Or say you have questions concerning these issues, whether concerning sexual relationships how they are or how to protect yourself. You as a girl, whom would you love to discuss them with? Or who should talk about them to you? Or maybe if you have a question, you can go with your question freely. And also if you had a problem, you want help. Say for example you have been found pregnant or sexually transmitted diseases. Where you would love to go and seek help as a girl, where would it be?

P: Umm…

I ….If you were told to choose the person whom you would discuss with these issues. The one you would ask these kind of questions for them to tell you the correct counseling, and also where you can find appropriate help concerning these issues? Whom would you love to be telling you?

P: Should I mention my relative?

I: Anyone else that you are thinking about. Right now it is up to you. If they would talk with you, and you would feel comfortable to ask the questions you have. Or maybe you want help on these kinds of issues. For you, yourself; who would you love it to be?

P: I would love MKK.

I: Who?

P: MKK.

I: Who is MK?

P: The one who teaches us about OC.

I: Ooh! Your facilitator?

P: Yes.

I: The one who came here?

P: Yes.

I: Okay. Why?

P: Because those issues I knew them through her.

I: Through her?

P: Yes.

I: Alright. What about in this village, is there anywhere else where there is a Non Governmental Organization like that of OC where the young maybe; or some older person you are able to go and find messages like these?

P: Umm. There is none.

I: In this village there is none?

P: Yes.

I: When there was no OC, where did people go and learn these things? Where did they hear them from?

P: Maybe they were learning; but us we weren’t learning. We were just staying.

I: You were just staying? You were just staying, ignorantly?

P: Yes.

I: Okay. Now, in this your village, or here in your area, how common is it that girls are found in sexual relationships with boys?

P: It is very common.

I: Your views on this behavior, what are they?

P: This behavior is bad.

I: Why?

P: You can get early pregnancies.

I: Yes,

P: Sexually transmitted diseases, and also HIV and AIDS.

I: Okay. Now what is it that makes girls to be involved in this kind of behavior?

P: What causes?

I: Yes.

P: Maybe there is lack of social help in their homes.

I: Yes,

P: Others do not listen when their parents advice them. They just say “it’s better we should be doing this we should find money daily.”

I: Yes,

P: Others maybe peer pressure, if you chat with friends who do the same things. You also copy their behavior that you should also be doing the same things.

I: What about when you are with your friends whom you chat with, is there a time when you discuss about relationships as these?

P: [Silence]

I: With your best friends whom you chat with?

P: But it is not often. I discuss with my friend, the one I chat with.

I: Do you have a boyfriend?

P: Yes.

I: Why did you decide to have a boyfriend? Not that I’m saying that it is wrong. But I just want to know.

P: Me too I was peer pressured, right?

I: Yes,

P: All my friends I used to chat with happened to have boyfriends. So I used to walk with them, right?

I: Yes,

P: I walking with them, walking with them daily and asking them “where are you getting money from?” they started telling me, “you should have boyfriends.” And then a guy asked me to be his girlfriend and I accepted. But it doesn’t please me to be doing this in my life.

I: So he gives you money? Sometimes?

P: Yes.

I: Money like how much?

P: He gives me two thousand. And sometimes maybe five thousand.

I: So what do you do with it?

P: I hide it. It doesn’t reach my home.

I: [Laughs]. But what do you do with it?

P: I just spend it.

I: Okay. Apart from giving you money, what other thing do you find in a relationship? That pleases you in the relationship, what is it?

P: Nothing.

I: So why do you do it?

P: [Laughs]. That’s why I’m saying I do it but it does not please me. It’s just because of my friends.

I: Okay. The relationship is still exists?

P: Yes.

I: So he just gives you the money freely?

P: It has just started.

I: It has just started?

P: Yes.

I: What about when he will say he wants sexual intercourse?

P: Iiih, on that it will be difficult.

I: It will be difficult?

P: Yes.

I: You, what are you plans on this relationship?

P: I just want to be in a relationship with him right now. The rest should be done in the future. The things that he wants.

I: When in the future? Up to when? Do you go to school?

P: Right now I have stopped going.

I: Why have you stopped going?

P: Notebooks.

I: Because of notebooks?

P: Yes.

I: In which standard where you?

P: Standard seven.

I: Standard seven?

P: Yes.

I: But I thought you have said that you are receiving, he gives you money?

P: The money; it is not often.

I: Okay. So you were saying your plan is that the rest you should do them in the future?

P: Yes.

I: In the future like when?

P: Maybe I when start going to school again and finish.

I: So he will be waiting?

P: He should continue waiting.

I: Okay. Does the boy go to school?

P: He wrote his four examinations.

I: He wrote form four examinations?

P: Yes.

I: Now he is just staying?

P: Yes.

I: Okay. What about here in your village, the behavior of girls getting married while they are young as your age, how common is it?

P: It is very common.

I: What do you think causes it to be like that?

P: What causes it?

I: Yes.

P: It brings poverty when you get married while young. And also sexually transmitted diseases and also death.

I: Okay. So you told me that you are not in school because of lack of notebooks.

P: Yes.

I: What else is it that made you to stop going to school? Apart from the notebooks?

P: It was that when I say I want money for fees to pay and write exams, they wouldn’t give me. For example last term I did not write exams. So I thought “even if I go, where will I get the money to pay for school fees?”

I: Okay. So what is it that made you to join the Dreams Program?

P: Nothing. I just wanted to hear more concerning the youth.

I: Okay. Who is it that approached you to join this program?

P: MKK.

I: Okay. So what are you benefiting from this program? What is the benefit?

P: I am benefiting a lot. For example in this our group, before I got tested for HIV at the hospital, right?

I: Yes,

P: It happened that when we were just joining, they told us that we should go and get tested for HIV; the same people from OC. So the first benefit I found was that one.

I: Yes,

P: And also our parents are doing shares, saving money. Yeah.

I: So for you, your grandmother is the one who does the shares?

P: Yes.

I: Okay. For how long have you been in the program of Dreams?

P: Seven months.

I: Seven months?

P: Yes.

I: Okay. Apart from helping you that you should go to the hospital and get tested for HIV. What other things does the OC do for you at the club there?

P: Just teaching us.

I: Okay. Do you have any thought of maybe getting out of this club?

P: No I don’t have.

I: Okay. What about if your friend wanted to enter in this program, what would you tell her? What would you advice her? A person who wants to join. Or would you advice your friends to join? Those friends that have not yet joined this program?

P: I would tell them to join but they should avoid talking around.

I: Okay. Meaning what? Talking around, what do you mean?

P: Because when you are in a group, it is not necessary that you should be gossipers. It is necessary that you should be united people. So I would advise her to avoid what? To avoid that.

I: Okay. Thank you very much because of your participation in answering these questions, right?

P: Yes.

I: I don’t know. Maybe you have a question? You have something you want to ask?

P: No I don’t have.

I: Okay. Thank you
