# Supplementary material for: Social support and very young adolescent girl’s knowledge on sexual relationships: A comparative qualitative study of Girl Only Clubs’ participants and non-participants in rural Malawi
Source: PLOS Glob Public Health. 2023 Jan 12;3(1):e0001339. doi: 10.1371/journal.pgph.0001339 (PMC10022037; doi:10.1371/journal.pgph.0001339)
Supplement: S1 Data — (ZIP) [file pgph.0001339.s003.zip › Anonymized/IDI2_R2_XX3_Apr.docx]

District: XX

IDI ID: IDI1_R2_XX3_April2019

Location: B

Interviewer: XXX

Data collection date: DD/MM/2019

**Interviewer**: As I have already explained, I’m from COM, We are conducting this research together with OC and PC of the USA. In this survey we are talking to different girls who joined the GG Clubs, especially adolescent girls that are aged between 10 and 14 years old. We want to know what your experiences have been like during the time that you have been participating in the OC clubs and we are also interested in knowing how girls of your ages in this village and the surrounding villages learn about issues to do with HIV and how they can protect themselves from getting pregnant as well as how they access information concerning these issues here in Liwonde. So we are interviewing different types of girls whether they have children or not, at this GG club. Information given will help when planning programs for such girls. Feel free to answer the questions there is no wrong answer, so to begin with whom do you stay with?

**Respondent**: I stay with my parents

**Interviewer:** Other people you stay with?

**Respondent**: My brothers and sisters

**Interviewer**: how many are you?

**Respondent**: four

**Interviewer**: Who is older?

**Respondent**: me.

**Interviewer**: okay, so, how old are you?

**Respondent**: I’m 14

**Interviewer**: so, aaah, (silent) so, (long pause), okay fine who is responsible for proving households needs at your home like buying like food or finding food?

**Respondent**: My father

**Interviewer**: Your father? Okay, okay… you said you have brothers and sisters, how many are boys and girls?

**Respondent**: One boy aah nop... Two boys and three girls.

**Interviewer**: Do they all go to school?

**Respondent**: Yes they do.

**Interviewer**: Do you go to school?

**Respondent**: No, I don’t.

**Interviewer**: So, why you don’t go to school?

**Respondent**: mmm, I stopped for various reasons

**Interviewer**: So, I want you to explain the reasons why you dropped out of school?

**Respondent**: Because I didn’t have books and school uniform.

**Interviewer**: But you have explained that your relatives... they all go to school, so how do they manage to get uniforms and books?

**Respondent**: My brother bought for them.

**Interviewer:** Who is older between you and your brother**?**

**Respondent:** My brother.

**Interviewer**: But you told me that you are the first born?

**Respondent**: Mm no I thought you said to girls, my brother is the oldest in our family.

**Interviewer**: Does he work?

**Respondent**: Yes he does

**Interviewer**: Where?

**Respondent**: He is in South Africa

**Interviewer**: Why didn’t you get that assistance too as your siblings?

**Respondent**: Aaah by then he wasn’t in South Africa.

**Interviewer**: When did you drop out of school?

**Respondent**: Last year

**Interviewer**: ooho you dropped last year, ooh, tell me other reasons you that forced you to dropped out?

**Respondent**: That’s all

**Interviewer**: Was it only lack of school uniform and books?

**Respondent**: Mm

**Interviewer**: What if you find these things can you go back to school?

**Respondent**: Yes, I can start again.

**Interviewer**: But your brother is in south?

**Respondent**: Mm yeah

**Interviewer**: The assistance he is giving to others, why not assisting you as well?

**Respondent**: He will also be giving me.

**Interviewer**: He will also be giving you?

**Respondent**: yeah

**Interviewer**: So, as you have said you don’t go to school, so what do you do when you are just home?

**Respondent**: I just stay

**Interviewer**: you just stay?

**Respondent**: yeah

**Interviewer**: So, what are your future plans?

**Respondent**: (silent)

**Interviewer**: As you are not going to school, when you think of your future, what are your plans?

**Respondent**: I just want to start school again.

**Interviewer**: you want to go back to school?

**Respondent**: yeah

**Interviewer**: okay, so I want to know, you should explain to me your daily schedule at home from morning to the evening?

**Respondent**: Fetching water, Sweeping, cooking and washing dishes.

**Interviewer**: Then what else do you do when you finish your household chores?

**Respondent**: cooking

**Interviewer**: like the way it is now, you have finished cooking, like this afternoon. What do you do?

**Respondent**: I just play.

**Interviewer**: You just play?

**Respondent**: yeah

**Interviewer**: Where do you play?

**Respondent**: At my neighbors compounds.

**Interviewer**: Any other place that you do go apart from being home?

**Respondent**: I go to play netball.

**Interviewer**: you go play netball?

**Respondent**: yeah

**Interviewer**: Okay, I wanted you explain to me…aaah when girls go to play netball, what are other things you face apart from playing sport or any other challenge that you face when you go to play netball or any other thing that make you feel happy whilst there?

**Respondent**: yeah, we do play without any challenges.

**Interviewer**: You face no any challenges there?

**Respondent**: Yeah.

**Interviewer**: Okay, is there any other day you have ever faced any challenge when you went to play netball... as there are other places that when girls go to play, boys do insult the girls.

**Respondent:**  mmmh

**Interviewer:** Like those things.

**Respondent**: They call me an old woman (ntchembere)

**Interviewer**: your friends call you that because you have a baby?

**Respondent**: Yes.

**Interviewer**: So, what do you do, when they call you by that name?

**Respondent**: I just stay quiet and listen to them.

**Interviewer**: You just stay listening, when they are insulting you?

**Respondent**: Yes.

**Interviewer**: Okay, so, I also wanted to know do you have a best friend or someone you spend most of your time with.

**Respondent**: Yes.

**Interviewer**: You do have…, your best friend?

**Interviewer**: What do you often do with this friend?

**Respondent**: Just chatting.

**Interviewer**: mmmh (The interviewer answered a greeting from a certain man) …you just like chatting? What stories do you discuss, I want to know the type of stories you discuss, …? explain about any topic that when you are together you feel free to discuss with her, like maybe something happened to you and then you told her, what issues do you discuss?

**Respondent**: Stories like how we can behave.

**Interviewer**: Feel free, tell me the stories you discuss? What is your friend’s name?

**Respondent**: XXY.

**Interviewer**: okay, XXY

**Respondent**: We discuss about family planning because she also has a baby.

**Interviewer**; mmmh, she also have a baby?

**Respondent**: yeah

**Interviewer**: So, what do you say concerning issues of family planning?

**Respondent:** Like encouraging each other to go for injections, for family planning.

**Interviewer**: okay so what else do you discuss?

**Respondent**: that’s all

**Interviewer**: mmmh, that’s all?

**Respondent**: yeah

**Interviewer**: That’s all that you do? Just discussing about family planning only??

**Respondent**: Yes, and we also discuss about diseases prevention.

**Interviewer**: mmh, I wanted to know, what really happened for you to have a baby?

**Respondent**: I had a boyfriend.

**Interviewer**: You had a boyfriend and now you broke up?

**Respondent**: Yes.

**Interviewer**: When did you meet your boyfriend, I want to know how and where you did you meet?

**Respondent**: We met at the market.

**Interviewer**: You met at the market?

**Respondent**: yeah

**Interviewer**: What does the boy do?

**Respondent**: Works at bus stage (ojiya) minibus call boy.

**Interviewer**: Is he from this village?

**Respondent**: No, from Ntuambuzi.

**Interviewer**: How old is he?

**Respondent**: He is 19 years old.

**Interviewer**: Tell me what really happened at the market, you should be explaining?

**Respondent**: (Laughs mhh), he proposed to me and I accepted.

**Interviewer**: Same day?

**Respondent**: Yeah (laughs)

**Interviewer**: So, I want to understand, when you accepted this relationship what were you expectations?

**Respondent**: Was expecting pregnancy.

**Interviewer**: I mean, what you wanted to benefit…aah to be happening in the relationship, like what was your aim behind this relationship?

**Respondent**: I wanted to be finding things like soap, body lotion and clothes.

**Interviewer**: and clothes, mmmh, okay, so what were the expectations of the boy?

(Baby cries and the interviewer told respondent to breastfeed the baby)

**Respondent**: Like his expectations? Mmmmmmm

**Interviewer**: Feel free, please speak out

**Respondent**: (silent, fails to respond to the question)

**Interviewer**: So, before you met your boyfriend did you know anything about HIV & AIDS or pregnancy?

**Respondent**: Yes.

**Interviewer**: I’m asking were you already aware of these?

**Respondent**: yes, I was already aware of these.

**Interviewer**: Where did you hear about it? Maybe you should tell me the first time you ever heard about HIV & AIDS?

**Respondent**: Was hearing it when people were talking.

**Interviewer**: What did you hear? Be open tell me, what did you hear, like the first time that you heard about HIV, what were people saying and where was that?

**Respondent**: People were saying that people contract HIV through sexual intercourse, sharp objects, and toothbrush.

**Interviewer**: Where did you hear this information?

**Respondent**: At school.

**Interviewer**: Was it like learning in class or you just heard people discussing about it?

**Respondent**: We were learning.

**Interviewer**: you were learning in class? Okay, any other place you heard about HIV & AIDS?

**Respondent**: Here at home.

**Interviewer**: Who was talking about it?

**Respondent**: My Parents.

**Interviewer**: That time did you understand what really HIV is?

**Respondent**: yeah

**Interviewer**: What did you understand about HIV, what is it?

**Respondent**: A virus that causes AIDS.

**Interviewer**: okay okay, in your mindset, what questions did you have concerning HIV & AIDS? Like what you didn’t understand?

**Respondent**: (silent) ……..

**Interviewer**: like when you were living, what did you want to know, as a girl, what questions did you have concerning HIV?

**Respondent:** (silent)

**Interviewer**: Remind me your name? XXXX?

**Respondent**: XXXX.

**Interviewer**: But you are shy be open. (Silent)… With your boyfriend have you ever discussed about HIV &AIDS?

**Respondent**: No, we never did.

**Interviewer**: Why, didn’t you discuss about HIV with your boyfriend?

**Respondent**: Nothing, because we both didn’t have it.

**Interviewer**: How did you know that you didn’t have it?

**Respondent**: We went for the testing.

**Interviewer**: You went for the testing?

**Respondent**: Yes we did.

**Interviewer**: Where, did you get tested?

**Respondent**: At the Y hospital

**Interviewer**: At Y?

**Respondent**: Yes.

**Interviewer**: Okay, anything you ever heard about HIV & AIDS?

**Respondent**: (Asks for clarification)

**Interviewer**: or else have you ever discussed with anyone about HIV & AIDS?

**Respondent**: No.

**Interviewer**: or else someone explaining to you, or you discussing with someone issues concerning HIV?

**Respondent**: no

**Interviewer**: you have never discussed?

**Respondent**: Yeah

**Interviewer**: okay okay so, you when you see girls in this community, like fellow peers. Do you think these girls are at a risk of contracting HIV?

**Respondent**: Yes.

**Interviewer**: Why?

**Respondent**: They like sleeping around.

**Interviewer**: As of now do you have any questions about HIV & AIDS that you desire to know?

**Respondent**: No

**Interviewer**: You really don’t have any questions?

**Respondent**: No, I don’t.

**Interviewer**: okay, okay, tell me when and where you first heard about getting pregnant, where did you hear about getting pregnant?

**Respondent**: Through sexual intercourse.

**Interviewer**: Yeah, so, I want you to tell me where you heard about it for the first time? As I have said that I want to know how and where girls know these issues.

**Respondent**: At school.

**Interviewer**: you heard at school?

**Respondent**: yeah

**Interviewer**: In class?

**Respondent**: No the teacher was teaching us.

**Interviewer**: but you are sounding very low or else we have sat at wrong place?

**Respondent:** no

**Interviewer:** mmmmh

**Respondent:**  They were teaching us.

**Interviewer:** at school?

**Respondent:** yeah

**Interviewer:** okay okay, what were you taught?

**Respondent**: They taught us that we can become pregnant.

**Interviewer**: Through what way?

**Respondent**: Sexual intercourse.

**Interviewer**: mmh, so after learning this, did you have any question that you wanted to know concerning pregnancy?

**Respondent**: No

**Interviewer**: Where is the other place you heard about getting pregnancy?

**Respondent**: Here in the village.

**Interviewer**: Where actually?

**Respondent**: When chatting

**Interviewer**: when chatting with friends?

**Respondent**: yeah

**Interviewer**: mmmh, okay, so, I want to know more, you are a special case since you have a baby. When you slept with your boyfriend did you know that you will fall pregnant? I want to know what really happened.

**Respondent**: I didn’t know

**Interviewer**: I want you to explain, what happened, how often did you have sex to till when you got pregnant.

**Respondent**: Mmm about some months.

**Interviewer**: How often did you sleep with him, were you having sex frequently? I want you to be open.

**Respondent**: (laughs), once

**Interviewer**: Then you just realized that you are pregnant? Why didn’t you use protection?

**Respondent**: I Just thought he is my beloved one, no need for any protection.

**Interviewer**: Did you know that you could get pregnant or not you just didn’t mind?

**Respondent**: I didn’t know.

**Interviewer**: But you have just told me that at school you were told that unprotected sex can end you pregnant? I want to know what was in your mind, why did you accept sleeping with him.

**Respondent**: He was forcing me.

**Interviewer**: He was forcing you? What was he saying as the reasons for forcing you?

**Respondent**: (laughs), I don’t know.

**Interviewer**: When forcing you what was he saying, why was he forcing you? I know that they were reasons behind, I’m I lying?

**Respondent:**  No

**Interviewer**: So, explain to me, why are you not open?

**Respondent**: I don’t know what his reasons were.

**Interviewer**: So he was just saying lets have sex without any reason?

**Respondent**: Yes.

**Interviewer**: Okay, okay, so where is he now? Are you getting married?

**Respondent**: No, he is married now.

**Interviewer**: Why didn’t he marry you?

**Respondent**: I don’t know.

**Interviewer**: After impregnating you how long did you date?

**Respondent**: One year.

**Interviewer**: After impregnating you?

**Respondent**: No, the whole relationship lasted a year.

**Interviewer**: Then after the pregnancy what happened?

**Respondent**: He left me and found another girl.

**Interviewer**: After telling him that you are pregnant what did he say?

**Respondent**: He just said okay.

**Interviewer**: And then what happened?

**Respondent**: I just heard that he has married.

**Interviewer**: Okay, why didn’t he marry you?

**Respondent**: Don’t know, when I was in hospital just heard that he is married.

**Interviewer**: Where do you get assistance for the baby?

**Respondent**: From my mother.

**Interviewer**: okay, does she work?

**Respondent**: No, she doesn’t work, she just stays.

**Interviewer**: I also wanted to know aaah, what you faced after getting pregnant, what did your parents do, I want to know did you tell your parents, or they discovered themselves?

**Respondent**: They discovered themselves.

**Interviewer**: Why didn’t you tell them?

**Respondent**: I didn’t know.

**Interviewer**: Clarify please?

**Respondent**: I didn’t know that I’m pregnant.

**Interviewer**: When getting pregnant you already had monthly period?

**Respondent**: Yes.

**Interviewer**: Then you just realized that you have missed the menstruation period for that month?

**Respondent**: I just thought that I was on a break.

**Interviewer**: Okay, so I wanted to know, how long did your mother discover that you are pregnant?

**Respondent**: After three months.

**Interviewer**: Three months, what did she notice?

**Respondent**: (laughs) I don’t know, she is old enough to know.

**Interviewer**: What did she do, did she ask you? I want to know only that you are not open enough, you know what happened and how she asked you.

**Respondent**: She just asked me if I’m having monthly periods (menstruation), then I answered her no.

**Interviewer**: Then what happened?

**Respondent**: She knew that am pregnant.

**Interviewer**: Then, she told you that you are pregnant? So what happened then?

**Respondent**: It was normal.

**Interviewer**: Didn’t she shout at you?

**Respondent**: No she didn’t.

**Interviewer**: Didn’t she ask you who is responsible?

**Respondent**: She did, and I told her about the guy responsible.

**Interviewer**: mmmh

**Respondent**: That’s all, she didn’t bother.

**Interviewer**: She didn’t bother, okay so, any advice you got from your mother or her friends concerning pregnancy?

**Respondent**: No, I didn’t get any.

**Interviewer**: What did you experience during your entire pregnancy? And how did you get assisted? As you were just young in that condition, what challenges did you face, as when one is pregnant, experiences lot of side effects like stomach problems, leg pains, nausea, vomiting, what did you face?

**Respondent**: Aaah I didn’t feel any symptoms like nausea, stomach problems I didn’t experience.

**Interviewer**: What did you face, you just felt normal until you delivered?

**Respondent**: Yes.

**Interviewer**: When you approached time to deliver, what really happened?

**Respondent**: I experienced the symptoms and went to the hospital.

**Interviewer**: Who did you go with at the hospital, who did you tell after the signs?

**Respondent**: My mother.

**Interviewer**: So, she is the one who took you to the hospital?

**Respondent**: Yes.

**Interviewer**: Did you go to prenatal clinic during your pregnancy?

**Respondent**: Yes, for four months.

**Interviewer**: How did the hospital workers treat you being that you are young?

**Respondent**: They didn’t say anything, they were testing me, screening me

**Interviewer**: how did they treat you upon seeing you that you are just young, …did the medical practitioner tell you anything?

**Respondent**: They did not say anything.

**Interviewer**: They did not say anything, and you didn’t face any challenge?... So how many times did you go to the prenatal clinic?

**Respondent**: Four times, I started going when the pregnancy was four months old...

**Interviewer**: How did midwifes welcome you when you went for delivery?

**Respondent**: They welcomed me warmly.

**Interviewer**: What problems did you face during this labor period, what challenges did you face?

**Respondent**: They did the testing and told me to go back and wait, after that I went back to the hospital and delivered the baby. (But it was through force labor)

**Interviewer**: What have you experienced during your entire period of taking care of the baby?

**Respondent**: Like?

**Interviewer**: Like taking care for the baby.

**Respondent**: I do take care for the baby very well.

**Interviewer**: Since you have a baby and you had a boyfriend, when you did first heard that a girl can be in a relationship with a boy or a boy can be in a relationship with a girl

**Respondent**: I heard it when I was young.

**Interviewer**: Where and how did you hear that a person can be in relationship?

**Respondent**: I heard through my friends, when chatting we were discussing.

**Interviewer**: How often do girls end up in relationships with boys here… like how often?

**Respondent**: Most girls do have boyfriends here.

**Interviewer**: So, what are the reasons girls end up in relationships?

**Respondent**: They want to have their needs.

**Interviewer**: (Long pause) okay, now I want to know how you are a member of GG club, do you participate in the GG club activities?

**Respondent**: yeah

**Interviewer:** So, how did you you know about the GG club?

**Respondent**: I heard from the village headman.

**Interviewer**: Did he call you? Okay, what happen that he should choose you to be in this programme?

**Respondent**: They wanted girls from 13 to 14 years old.

**Interviewer**: So, you were chosen because you were 14?

**Respondent**: yeah

**Interviewer**: Okay, what motivated you to join this club?

**Respondent**: We were learning.

**Interviewer**: I mean before joining the club, since you were many of you, but others didn’t join, what made you to accept and join?

**Respondent**: I wanted to be learning various things.

**Interviewer**: mmmmh, so, for how long have you been in this club?

**Respondent**: I started last year.

**Interviewer**: So, it has been a year?

**Respondent**: Yes.

**Interviewer**: Okay so, what do you benefit from this club, pertaining to what happens to this group, what do girls benefit?

**Respondent**: We learn good things.

**Interviewer**: So, I want that you should be explaining to me those good things, because you just say good things, what I’m I going to write?

**Respondent**: We learn how to avoid getting pregnant and family planning.

**Interviewer**: Any other thing apart from learning family planning?

**Respondent**: How to prevent… contracting diseases.

**Interviewer**: So, you just learn how to prevent contracting diseases and preventing getting pregnant?

**Respondent**: And about menstruation.

**Interviewer**: or is there anything else that is offered there?

**Respondent**: From OC?

**Interviewer**: Maybe services like HIV testing?

**Respondent**: yeah, we did test for HIV.

**Interviewer**: From OC? So, I want to know what happens because I want to know these services.

**Respondent**: yeah, we tested for HIV

**Interviewer:** Do service providers come to test you?

**Respondent:** yeah, they do come.

**Interviewer**: What else? What a about family planning issues?

**Respondent**: We don’t learn that.

**Interviewer**: Who came for the testing services?

**Respondent**: A certain woman, who comes with rations.

**Interviewer**: mmh, okay(silent), so have you ever been referred to somewhere to get services like family planning or HIV & AIDS, by managers from OC?

**Respondent**: Yes, about family planning, no.

**Interviewer**: What about, issues of HIV maybe being given a card so that you can get services somewhere?

**Respondent**: No, we have never been given.

**Interviewer**: Okay, so what have you benefited since joining and attending the GG club or how has your life been transformed?

**Respondent:** (silent)

**Interviewer:** How has your life been transformed?

**Respondent**: We learnt how to prevent contracting diseases.

**Interviewer**: mmmh, okay, okay, can you encourage others to join the club according to how you have benefited from the club?

**Respondent**: Yes.

**Interviewer**: Why?

**Respondent**: Because I have learnt a lot about preventing HIV & AIDS.

**Interviewer**: Thank you very much for attending the interview. Do you have any question?

**Respondent**: No.
