# Supplementary material for: Social support and very young adolescent girl’s knowledge on sexual relationships: A comparative qualitative study of Girl Only Clubs’ participants and non-participants in rural Malawi
Source: PLOS Glob Public Health. 2023 Jan 12;3(1):e0001339. doi: 10.1371/journal.pgph.0001339 (PMC10022037; doi:10.1371/journal.pgph.0001339)
Supplement: S1 Data — (ZIP) [file pgph.0001339.s003.zip › Anonymized/IDI3_R2_XX4_Apr.docx]

District: XX

IDI ID: IDI1_R2_XX4_April2019

Location: B

Interviewer: XXX

Data collection date: DD/MM/2019

**Interviewer: S**o, we are conducting a research study in relation to GG clubs of OC. We want to learn how the GG clubs operate here with adolescent young girls like in the age range of 10 to 14 years. As I had explained at the beginning that my name is Wanangwa and I work for College of Medicine. We are doing this research in collaboration with OC and PC of the USA. In this study I just want to learn about your experiences in the GG club. In addition I want to learn how girls of your age in this community acquire knowledge about various sexual health issues such as HIV/AIDS, pregnancy and other related issues. So I want you to be free with me, there is no response that you can tell me that is wrong. Any response that you give me will be of value to me.

**Respondent:** mmmh

**Interviewer:** So, first of all, whom do you stay with here?

**Respondent:** I stay with my grandparent...

**Interviewer:** Okay, are your parents alive?

**Respondent:** My father passed away.

**Interviewer:** what about your mother?

**Respondent:** She went to LL.

**Interviewer:** What does she do in LL?

**Respondent:** She works there.

**Interviewer:** Okay fine, who else do you stay with, it’s only you and your grandparent?

**Respondent:** Yeah, my grant parent and that elder sister.

**Interviewer:** Which sister?

**Respondent:** That one over there.

**Interviewer:** Okay, who else?

**Respondent:** And rest are children.

**Interviewer:** Okay, do you go to school?

**Respondent:** I dropped out, but I have just started again.

**Interviewer:** Okay, which level are you?

**Respondent:** I’m in standard 6.

**Interviewer:** mmmh, why did you drop out from school?

**Respondent:** I lacked some school materials like, pens, books…

**Interviewer:** mmmh .

**Respondent:** …so now, I have decided to go back since school is very important.

**Interviewer:** mmmh, when did you drop out or for how long have you not been going to school?

**Respondent:** About a month, I lacked examination fee, so I wasn’t writing exams.

**Interviewer:** mmmh, so, how have you managed the issue of lack of pens, books and examination fees?

**Respondent:** With that issue, I can be doing some piece works to raise funds to pay for exams.

**Interviewer:** mmmh, doesn’t your mother send some money to help you in these needs, since you said she is working in Lilongwe?

**Respondent:** currently she is not working, she has gone into farming.

**Interviewer:** mmmh, okay…. okay, can you tell me how you spend your free time after finalising your daily schedule…say when you are done with cooking and other household chores?

**Respondent:** I wash plates, I go to fetch water..., when I am done, I study the things I have learnt.

**Interviewer:** Do you have friends where you go to chat or where else do you go apart from just being home?

**Respondent:** Yeah, I go for a chat.

**Interviewer:** Where do you go?

**Respondent:** Behind that house…

**Interviewer:** mmmh,

**Respondent:** …there is my friend.

**Interviewer:** mmmh, it’s only here, at your friend’s and school where you go or there is another place?

**Respondent:** yeah only those places.

**Interviewer:** True?

**Respondent:** Yeah

**Interviewer:** Don’t you go out for sports or elsewhere?

**Respondent:** yeah, we do go out to watch and play sometimes.

**Interviewer:** mmmh, how safe are these places for girls, don’t you meet any threat…like being harassed?

**Respondent:** no, we don’t.

**Interviewer:** You don’t face any kind of challenge?

**Respondent:** yeah

**Interviewer:** Okay…., okay aaaah, you have said you stay with your grandparent, how happy are you staying with your grandparent, are you free in this place, do you like this place, or you don’t and why?

**Respondent:** I like this place because there is a big land to farm and we are near the market.

**Interviewer:** mmmh, do you like staying here and how happy are you staying with your grandparent and what challenges do you face as a girl?

**Respondent:** I’m always happy, in don’t face any challenge.

**Interviewer:** mmmh, tell me a moment when you faced a challenge and you presented your issue to your grandparent?

**Respondent:** You mean, a challenge?

**Interviewer:** yeah

**Respondent:** I have never faced any challenge.

**Interviewer:** Now let us talk about some changes on your body…what came up into your mind the first time when you started noticing some changes in your body, changes, like development of breast, growing of hair in pubic regions, menstruation? What questions did you have and whom did you approach to advise you on this?

**Respondent:** I approached my grandparent and explained about the changes I experienced about my body.

**Interviewer:** So, what was the comment from your grandparent?

**Respondent:** She told me how I could be taking care of myself.

**Interviewer:** Oh okay, she didn’t even consult or refer you to other people to talk to you?

**Respondent:** she also consulted my elder sister, she advised me on how to take care of myself

**Interviewer:** mmmh, as a girl who has just reached puberty, what other advice did you receive apart from how you can take care of yourself?

**Respondent:** They told me to abstain from sex after menstruation periods in order to prevent pregnancy

**Interviewer:** mmmh, what else?

**Respondent;** To avoid sexual intercourse because it can put me at risk of contracting sexually transmitted diseases.

**Interviewer:** Oh okay, I have you ever attended initiation ceremony?

**Respondent:** you mean initiation for counselling?

**Interviewer:** yeah

**Respondent:** yeah

**Interviewer:** was it before puberty or not?

**Respondent:** Before puberty.

**Interviewer:** Do you remember the advises you received from the initiation ceremony, concerning HIV, pregnancy and many more?

**Respondent:** yeah we were advised on those issues.

**Interviewer:** what did they say? Or is there any difference in the contents of the advice… what you heard during menstrual guidance and during initiation ceremony?

**Respondent:** They advised us on the importance of abstaining from sex, so that we cannot contract sexually transmitted diseases at a young age.

**Interviewer:** you heard this during initiation ceremony?

**Respondent:** yeah

**Interviewer:** Okay, how old were you by the time you were attending initiation?

**Respondent:** when I was 12 years.

**Interviewer:** okay, okay, when and where did you first hear issues pertaining to HIV and AIDS?

**Respondent:** From OC.

**Interviewer:** Before OC is there any other place you heard of HIV and AIDS?

**Respondent:** It’s only OC where I heard about it.

**Interviewer:** what about at school?

**Respondent:** school, mmmh no I have never heard about it.

**Interviewer:** mmmh, what about the radio?

**Respondent:** no, I have never heard this from the radio.

**Interviewer:** So, what information did you gather about HIV from OC?

**Respondent:** We learnt about the use of condom during sexual intercourse.

**Interviewer:** what do they say HIV is?

**Respondent:** (silent)

**Interviewer:** How did they described HIV at OC?

**Respondent:** They said it’s a virus that compromises the body that makes a person who has the virus shy when interacting with people and that brings depression to people.

**Interviewer:** mmmh, where did you first heard issues of family planning and pregnancy?

**Respondent:** From school as well as OC.

**Interviewer:** mmmh, okay, have you ever discussed with anyone else on these issues apart from OC and at school?

**Respondent:** yeah, with friends

**Interviewer:** mmmh, so what did you discuss?

**Respondent:** But we rarely discussed about these issues.

**Interviewer:** okay, where did you get the information concerning relationships that exist among boys and girls?

**Respondent:** School and OC.

**Interviewer:** Mmmh, do you have a boyfriend?

**Respondent:** yeah, I have

**Interviewer:** mmmh, what was your aim for being in this relationship?

**Respondent:** That I should be getting some financial assistance.

**Interviewer:** mmmh, does your boyfriend work or conduct a certain business?

**Respondent:** no, he doesn’t work.

**Interviewer:** How old is he?

**Respondent:** he is 15 years old.

**Interviewer:** is he schooling?

**Respondent:** yeah

**Interviewer:** what level?

**Respondent:** form 1

**Interviewer:** How does he assist you as he is also a mere student as you are?

**Respondent:** (silent)

**Interviewer:** Where does he get the assistance?

**Respondent:** Sometimes he works at the bus depot.

**Interviewer:** mmmh, so what kind of assistance do you receive from him?

**Respondent:** He gives me money...

**Interviewer:** Okay, what does he benefit from the relationship?

**Respondent:** benefits?

**Interviewer:** since you say he gives you money, assistance, what does he benefit?

**Respondent:** We just like chatting in private places

**Interviewer:** mmmh, is this relationship a sexual relationship or you just chat and go?

**Respondent:** It is a sexual relationship

**Interviewer:** How long has your relationship lasted?

**Respondent:** Now we are no longer dating.

**Interviewer:** But it lasted for how long?

**Respondent:** a month

**Interviewer:** mmmh, why did you break up?

**Respondent:** We wanted to concentrate on school

**Interviewer:** mmmh, okay, okay, were you spending some time to discuss issues of HIV and AIDS when your relationship was on going?

**Respondent:** no

**Interviewer:** What about issues of pregnancy or the use of condoms?

**Respondent:** yeah

**Interviewer:** mmmh, did you use a condom during your sexual activity?

**Respondent:** yeah, we used.

**Interviewer:** where did you get them?

**Respondent:** he used to buy.

**Interviewer:** where did he get them?

**Respondent:** he bought from the market .

**Interviewer:** mmmh, okay, have you ever tested for HIV?

**Respondent:** yeah, I tested

**Interviewer:** where did you get tested?

**Respondent:** at the hospital

**Interviewer:** what motivated you to for testing?

**Respondent:** I just wanted to know my status.

**Interviewer:** what actually made you to think like that, was there anybody who directed you or on your own?

**Respondent:** I just thought it on my own.

**Interviewer:** where did you get the information that you can also go to the hospital for the testing to know one’s status?

**Respondent:** From OC

**Interviewer:** Okay, how did you become a member of these clubs, who told you how to join this club?

**Respondent:** My friend explained to me about these clubs, that there is come OC club that provide awareness in various issues

**Interviewer:** what has been your benefit so for being a member of OC?

**Respondent:** benefits from OC?

**Interviewer:** yeah

**Respondent:** I have learnt a lot pertaining to issues of HIV and AIDS.

**Interviewer:** mmmh, okay fine, how can you encourage the one who is interested in joining the goal girl club?

**Respondent:** I can encourage her to join so that she also learns and know various things from this club, ….OC.

**Interviewer:** Are there any challenges you faced when you were in the OC club?

**Respondent: N**o.

**Interviewer:** What challenges do you face as a girl?

**Respondent:** No, challenge that I have ever faced.

**Interviewer:** You don’t face any challenges?

**Respondent:** yeah

**Interviewer:** Okay, thank you for attending this interview, do you have any question?

**Respondent:** No, I don’t have any question.
