# Supplementary material for: Social support and very young adolescent girl’s knowledge on sexual relationships: A comparative qualitative study of Girl Only Clubs’ participants and non-participants in rural Malawi
Source: PLOS Glob Public Health. 2023 Jan 12;3(1):e0001339. doi: 10.1371/journal.pgph.0001339 (PMC10022037; doi:10.1371/journal.pgph.0001339)
Supplement: S1 Data — (ZIP) [file pgph.0001339.s003.zip › Anonymized/IDI4_R1_YY3_May.docx]

District: YY

IDI ID: IDI4_R1_YY_May2018

Location: A

Interviewer: WWW

Data collection date: DD/MM/2018

I: Firstly of all I would like you to explain to me; as a girl in this house, when you work up in the morning what do you do?

P: When I work up I wash my face, clean dishes and sweep the surrounding and by this time water is already fetched and I put it on fire, and thereafter, I take a bath and go to school.

I: So after school what do you do?

P: After school, I clean dishes and fetch water and start reading.

I: Alright, where do you go to chat with friends or who do you chat with?

P: At the Chiefs ground with my friends

I: What kind of games do you do when playing with your friends?

P: *Jingo*, *fulaye* and netball.

I: When you are all girls, what kind of issues do you discuss when chatting?

P: School issues

I: Do you discuss school issues daily?

P: No

I: Sometimes, what do you discuss?

P: Sometimes we discuss with our friends that when we found groups we should be able to tell friends so that we are doing them together.

I: Alright, is there any other person whom you are very open to?

P: Myself?

I: Yes, and who is that?

P: When we go to GGs group

I: Who is this person whom you are very open with?

P: CS

I: What are the issues that you chat with CS?

P: We talk about issue that we discuss at GGs

I: What kind of issues?

P: We learnt about ‘my dreams’ and we talk to each other that it is one’s idea to choose any job that one wishes to do when you finish education. And we discuss about visions, a girl who is focused. And we also learnt how to prevent HIV, use of condoms and use of contraceptives.

I: Alright. That means you were discussing about se, right?

P: Mmm

I: Okay, apart from GG club where you go, and where they talk about issues like sex, prevention of HIV/AIDS and prevention of pregnancy, use of condoms. Who else do you chat with whom you discuss such issues?

P: TW

I: What is it that you discuss with her?

P: We encourage each other to work hard at school so that in future we find a job and help ourselves.

I: Okay, have you ever talk to your parents on such issues?

P: Yes

I: What did they say?

P: They told me that when I go to school, I should pray there but listen to the teacher and when I come I should read my books so that I pass examination.

I: On HIV issues, have you ever talked to your parents about it?

P: Yes

I: What did they say?

P: They said that I should not have sex with anyone or get into a relationship so that I may not contract diseases.

I: Who said that?

P: My parents

I: Your father or mother?

P: My mother.

I: Okay, do you talk to your father on such issues?

P: Yes

I: What does he say?

P: He said that he should never see me being in a relationship with a boy, but I should work hard on my education.

I: In this village, where else can young girls like you go to find assistance on such issues?

P: From a mother group

I: From a mother group?

P: Mmm

I: Where does she stay?

P: Behind M mountain

I: Okay, how far is that place?

P: [pause]

I: It’s far, right?

P: Mmm

I: Have you ever gone to a mother group?

P: No

I: And when you have question concerning issues like HIV where do you go for information?

P: From my teacher

I: At school?

P: No, the counsellor for GG club

I: Mmmh, why do you choose to ask the teacher other than any other person?

P: Because she is the one who can explain better since she is the one who teaches us whenever we meet.

I: Okay, just assuming, if you are in trouble, you’ve found yourself pregnant, who would be the first person to be told?

P: My parents

I: Why?

P: Because they don’t allow me to have boyfriends so if am found pregnant I can tell them.

I: Alright. Sometimes in a village set up it has been found that boys asks for girl-boy relationships, have you ever encountered a thing like that?

P: No

I: It has never happened to you?

P: Yes

I: But maybe you have been curious enough to know what happens in a relationship or how does it go? If you have questions on such issues, whom would you ask?

P: I can ask my grandmother.

I: Why your grandmother?

P: Because she is an elder and knows everything and if I ask her she tell me with no problems.

I: Not your parents?

P: Yes

I: What about contraceptives issues; if you would like to know more about family planning whom would you ask?

P: I would ask the doctor

I: The doctor?

P: Yes

I: Do you have a doctor in this village?

P: No

I: Would you go to the hospital? Would you be able to go to the hospital and ask about contraceptives?

P: No

I: Why?

P: [pause]

I: Why can’t you go to the hospital to ask for contraceptives?

P: [pause]

I: Okay, for example, that your inlaw, right?

P: Yes

I: Your in law can go to the hospital to ask for contraceptives, right?

P: Yes

I: Why?

P: Because she is of the age that can give birth

I: What about you?

P: I haven’t come to an age that I can give birth

I: So, you cannot ask?

P: Yes, but if I reach the age that I can give birth, then I can go there and ask

I: So, those who has not reached child bearing age cannot ask about contraceptives?

P: Maybe they can go and ask just to know how it happens, so that when they reach child bearing age they should not have problems

I: Okay, what about issues of HIV or STI issues, whom would you ask?

P: My teacher

I: Your teacher?

P: Mmm

I: Your teacher from school?

P: Yes, a teacher from school or the one who teaches us GGs

I: Alright. Have you ever gone for initiation?

P: No

I: In your village, do you have organizations who looks after the issues we were discussing, issues like HIV, contraceptives?

P: No

I: Now I would like to ask you about boys and girls relationships in this village. Have you ever heard that girls has relationships?

P: Yes, I heard about that

I: So, what are your views on such relationships?

P: As for me, if a boy asks for that kind of relationship, I would say no.

I: Why?

P: Because one can get pregnant when you were not prepared and you would be suffering and give birth with problems and you will not have adequate materials.

I: What makes boys and girls in this village to have such kind of relationships?

P: Because some girls do not go to school and they feel they have grown up and they should just get married and they do that.

I: Have you discussed such issues with your parents? Issues of boys and girls relationships?

P: No

I: Have they counselled you on such issues?

P: Yes

I: What did they say?

P: They told me not have boyfriends.

I: What about your friends, have you ever discussed such issues?

P: Yes

I: What do you discuss?

P: That we should not indulge in such relationships but continue with our education.

I: As you are growing up, are there questions that you would like to know; especially about relationships?

P: No

I: Alright, in your village, do you have young girls like you who are married as of now?

P: No

I: But in your views, what do you think of young girls of your age being married?

P: I think they can be struggling and have problems when giving birth

I: Alright, you said that at one point in time you were not going to school, right?

P: Yes

I: Why?

P: Because I didn’t have uniform and they were chasing us from school

I: So what made you go back to school?

P: Because when I went to GGs group they taught us about our dreams and they told us that we can choose a jobs that we would love to do in future. And I went back to school so that I should be able to choose the job I would want to do when I finish my education.

I: Mmmh

P: And my parents started to do business and were able to give me some of the needs that I had at that time.

I: Alright, now I would like to know how you joined GG club group?

P: When the group came here, teachers told us about the group and we joined, and they started teaching us, with other things that we also learn from school.

I: How long have you been in this group? When did you start?

P: 2016

I: So, can you tell me what you have gained being in this group?

P: BLM came one day and conducted a test on us and now we know our status and also that at home my brother was not doing any house chores and after learning about gender I explained it to my brother that he doesn’t have to differentiate work between men and women. And he started working for example, when I cook in the afternoon he cooks in the evening, and he also fetches water.

I: What else did you gain?

P: Some of the things we learn at school are also taught at GGs

I: And I would like you to mention what you learn?

P: About gender, of which when we learn them at GGs then we will not fail at school, we also learn about ‘our dreams’ even at school.

I: What are the other things that happen at GGs club that you have taken part in?

P: We play games

I: What kind of games?

P: Netball, *fulaye*, and *kambuzi kalinkhonde*.

I: When you are at GGs club, are there times when you’ve been sent to receive assistance somewhere?

P: Assistance find us here

I: What kind of assistance?

P: I received a bag, and a OC printed shirt.

I: Alright. Are there obstacles that you meet because you are in GGs club?

P: Yes

I: What are the obstacles?

P: When we are going to GG club our friends castigates us that we are not gaining anything there but we don’t listen to them.

I: Why do you think they say that?

P: [pause]

I: What do they say when castigating you? Why do they say you are not gaining anything there?

P: That’s what they say that we do not gain anything but we are gaining

I: If you can be told to mention something that need to be changed at GG club, like on such such part things are not okay and would like to change; what would you say?

P: We were not having plays, we can be doing that and some of the things that our friends doesn’t know but we know them, we need to remind them about those things.

I: If there is a girl in your village and doesn’t know about GG club, would you tell her to come and join?

P: No, because the number is adequate and even if I tell her she cannot be allowed to join.

I: Oh okay, how many are you?

P: 25

I: So, they say that they cannot join?

P: Mmm

I: So, when we look back in 2016 when you joined GG club; how has your life changed?

P: My life has change, at first I didn’t know much but when I came to GG club I learn them.

I: Have you thought of stop being a member?

P: Stop going to GGs?

I: Mmm

P: No I cannot stop

I: Why?

P: Because I learn a lot of things; which we learn at GGs and at school they also teach us

I: For example, what is it that you learn at GGs and also at school?

P: About gender

I: What did you say that gender is?

P: Not differentiating the work between men and women

I: Okay, and are you practicing gender here at home?

P: Yes

I: Can you give me an example?

P: For example, when I clean dishes my brother also cleans dishes and when I fetch water he also fetches water. Everything I do, he also does the same next time.

I: Okay, your brother?

P: Yes

I: Alright, thank you for answering the questions. Do you have a question?

P: No

I: We’ve finished.

P: Okay.

END
