# Supplementary material for: Social support and very young adolescent girl’s knowledge on sexual relationships: A comparative qualitative study of Girl Only Clubs’ participants and non-participants in rural Malawi
Source: PLOS Glob Public Health. 2023 Jan 12;3(1):e0001339. doi: 10.1371/journal.pgph.0001339 (PMC10022037; doi:10.1371/journal.pgph.0001339)
Supplement: S1 Data — (ZIP) [file pgph.0001339.s003.zip › Anonymized/IDI4_R2_XX2_Apr.docx]

District: XX

IDI ID: IDI1_R2_XX2_April2019

Location: B

Interviewer: XXX

Data collection date: DD/MM/2019

**Interviewer**: So once again, I have come from COM and we are working together with OC and population council. We are conducting a research study whereby we are talking to various girls recruited in the GGs clubs. The aim is to learn how GG clubs are operating, especially for young girls like you ages from 10 to 14years, and I’m also curious to know how girls come to know various issues concerning, HIV or else family planning, and various issues concerning your sexual health. How do young girls, how do they learn these things? Are we together? So I want to listen to stories of for instance the first time you heard about HIV or Family planning, where did you hear this? What questions did you have and so forth and so on? Okay? So be free to tell me your story, as I indicated earlier all your responses will be of value to us, there is no wrong response..

**Respondent:** Yes

**Interviewer:** So, first of all I want to know how old are you?

**Respondent:** I’m 14 years old

**Interviewer:** Do you go to school?

**Respondent:** Yes

**Interviewer: W**hat level?

**Respondent:** Standard 6

**Interviewer:** Okay… okay, whom do you stay with here?

**Respondent:** With my mother.

**Interviewer:** your mother, and who else?

**Respondent:** My father.

**Interviewer:** Okay, how many children are you in the family?

**Respondent:** We are seven.

**Interviewer:** Seven…, and your number is what in your family?

**Respondent:** I’m the third born.

**Interviewer:** So how many girls and boys are there in your family?

**Respondent:** There are 4 boys and 2 girls.

**Interviewer:** So, that means you are six in the way you have explained?

**Respondent:** oh, there are five boys.

**Interviewer:** so, there are five boys and two girls?

**Respondent:** yeah.

**Interviewer:** okay, okay, so I wanted to know …. Okay (silent), which religion do you belong to?

**Respondent:** Muslim

**Interviewer:** Okay, you are Muslims, okay, have you ever at some point dropped out of school because of some other reasons?

**Respondent:** yes

**Interviewer:** okay, when did you drop out and for what reasons?

**Respondent:** I was lacking some assistance

**Interviewer:** What kind of assistance?

**Respondent:** Like books.

**Respondent:** How long did you stay without going to school?

**Respondent:** I stayed for three months.

**Interviewer:** for three months, okay so what happened for you to go back to school?

**Respondent:** (silent).

**Interviewer:** What made you go back to school?

**Respondent:** I just thought that I should go back to school.

**Interviewer:** You just thought to go back to school…, now upon going back to school, where are you getting the assistance, as you have said that you dropped due to lack of resources?

**Respondent:** Yeah, OC actually encouraged girls to go back to school, so I then thought of going back to school.

**Interviewer: B**ecause of OC?

**Respondent:**  yeah

**Interviewer:** Okay, so, what else, has OC done for you to go back to school?

**Respondent:** (silent), they have …. You mean what OC has done?

**Interviewer:** yeah, what they have done for you to go back to school?

**Respondent:** they encouraged me to be going to school….

**Interviewer:** mmmmh

**Respondent:** …that I shouldn’t just be staying at home but rather be at school.

**Interviewer:** What did they say would happen if you are just staying home?

**Respondent:** I can get unwanted pregnancy….

**Interviewer:** mmmh

**Respondent:** …and I can also contract diseases….

**Interviewer:** mmmh

**Respondent:** So, it was better for me to be going to school.

**Interviewer:** mmh, can you tell me your daily schedule when at home?

**Respondent:** I do some house hold chores.

**Interviewer:** mmmh, like what kind of chores?

**Respondent: Washing** dishes, cleaning the surrounding, drawing water

**Interviewer:** After finishing all these what else do you do?

**Respondent:** Bathing then eating.

**Interviewer:** After eating…. or how do you spend your free time?

**Respondent:** I do chat with friends

**Interviewer:** Okay, with these friends, where do you mostly like to chat?

**Respondent:** Like at the sports ground.

**Interviewer:** where else do you like chatting?

**Respondent:** That’s all, we like being at the ground, playing netball.

**Interviewer: So,** what stories do you like sharing?

**Respondent:** (laughs)

**Interviewer:** don’t you share some stories?

**Respondent:** Yeah, we do discuss

**Interviewer:** like for example did you chat with friend yesterday?

**Respondent:** yeah

**Interviewer:** which friend?

**Respondent:** A certain friend who stays there.

**Interviewer:** So, when you met yesterday, what did you discuss?

**Respondent:** (laughs), we discuss about going to the market tomorrow.

**Interviewer:** mmmh, okay, what other issues? I wanted to ask issues concerning, HIV, sexual activities, pregnancy, have you ever discussed with your friends or what did you hear from your friends?

**Respondent:** like HIV?

**Interviewer:** yeah, when with friends, do you discuss?

**Respondent:** yeah, we do discuss

**Interviewer:** yeah, just tell me A single day when you discussed issues concerning HIV, wherever you were be it at the ground or where else.

**Respondent:** We were discussing that being in sexual relationships it’s not good because we can contract HIV…

**Interviewer:** mmmh

**Respondent: …**we also discussed the use of condom so that we can prevent infections

**Interviewer:** mmmh, what did you learn or hear all these?

**Respondent:** From OC.

**Interviewer:** Before OC was established, where were you hearing all these issues? Or how can you compare what you heard about these things before OC came into existence and now?

**Respondent:** What we have learnt now with the coming of OC is good

**Interviewer:** How were you acquiring information about these issues before OC and what’s the difference in the way you are coming to know these issues with OC now?

**Respondent:** I did not know anything before OC.

**Interviewer:**  You didn’t know? Okay should we say you heard all these from OC?

**Respondent: Y**eah, we leant a lot from OC, issues of menstruation, HIV….

**Interviewer:** mmmh, pertaining to menstruation, what did you learn?

**Respondent: W**e leant that we should be very hygienic with menstrual pads or cloth by burning them or throwing them into toilets, so that people should not be seeing them.

**Interviewer:** Mmmh okay, what about issues of pregnancy or family planning, when and where did you first hear them and who was talking about this?

**Respondent:** I just heard when my friends were speaking.

**Interviewer:** mmmh, what were they saying?

**Respondent:** They were talking about family planning…to use injections or else use a condom to prevent pregnancy and HIV.

**Interviewer:** What questions did you have pertaining to these issues ?

**Respondent:** I had questions like how to protect ourselves and the use of condoms and its side effects

**Interviewer:** mmmh

**Respondent:** Yeah, so I leant from OC.

**Interviewer:** What other things that you really wanted to know?

**Respondent:** like to the issues we are discussing?

**Interviewer:** yeah, to the issues you have just told me like about the use of condom, and you say you leant from OC. You being so young, are there any other issues you wanted to know during these discussions?

**Respondent:** Yeah, I wanted to know how I could protect myself from pregnancy and how to use a condom... and also, I wanted to know what happens when the condom has teared apart. So, I heard all about these issues from OC…, they told me.

**Interviewer:** OK, what else you discussed…, you are telling me intriguing issues here.

**Respondent:** Like you mean just here or what we discuss with OC somewhere?

**Interviewer:** Anywhere be it here or OC

**Respondent:** Okay, at OC, I discussed with (xxxx), who taught me all these.

**Interviewer:** Okay, whom do you feel comfortable with, discussing all these issues?

**Respondent:** A certain friend of mine.

**Interviewer:** Why do you choose this person?

**Respondent:** I did that for secrecy sake or privacy, as some people are not trustworthy.

**Interviewer:** mmmmmh

**Respondent:** So, I chose her so that I should be so much open discussing issues together.

**Interviewer:** So, you discuss these issues?

**Respondent:** yeah

**Interviewer:** mmmh

**Respondent:** We discuss on issues that can actually make us get into trouble such as falling pregnant…

**Interviewer:** mmmmmh

**Respondent:** …And that for us not to fall pregnant we must be using condoms…

**Interviewer:** mmmh

**Respondent:** or else the injections as a family planning method.

**Interviewer:** mmmh , Okay, (silent), apart from OC, do you have any other place or center in this village where girls can go and get the guidance on issues related to HIV?

**Respondent:** It’s only at the hospital

**Interviewer:** Do the hospitals provide these services to young girls like you?

**Respondent:** mmm no

**Interviewer:** Why?

**Respondent:** It’s because we are very young.

**Interviewer:** So, what happens, do they like deny you from accessing the services?

**Respondent:** We have never gone so we don’t know

**Interviewer:** Can you explain, why you don’t go.

**Respondent:** We are just afraid of going there.

**Interviewer:** What makes you fearful?

**Respondent:** We feel like we can be rejected.

**Interviewer:** Because you are young?

**Respondent:** Yeah

**Interviewer:** Where else can you be free, or would you prefer going to access information pertaining to the issues we have discussed apart from OC?

**Respondent:** We don’t have such places here may be if you can find an adult.

**Interviewer:** mmmh, earlier you have said you stay with your mother and father, so between these two, whom are you open with to discuss these issues?

**Respondent:** My mother

**Interviewer:** So, have you ever discussed issues concerning HIV with her?

**Respondent:** No, we have never discussed.

**Interviewer:** What about asking her questions?

**Respondent:** No, I have never.

**Interviewer:** So, why you have never asked her?

**Respondent:** I just feel shy to do so.

**Interviewer: W**hat about your father advising you on these issues?

**Respondent: H**e advises us.

**Interviewer: Y**our father?

**Respondent: B**oth of them, they do advise us

**Interviewer: T**ell me what happened…, one time for them to start advising you?

**Respondent: Th**ey were telling me not to be in any sexual relationship so that I can prevent contracting deseases and also prevent pregnancy.

**Interviewer:** mmmh okay (Long pause). Now I want us to discuss about issues of growing up…, like breast development, growing of hairs in the pubic body parts as well as menstruation. So, tell me what happened to you when you started growing breast or what questions did you have, or you knew this already?

**Respondent:** No, I had no idea about this.

**Interviewer:** Tell me what happened when you realized those changes on your body

**Respondent:** Yeah, by the time I was developing breast and I also started experiencing menstruation then I was really surprised such that I consulted elders to counsel me on this.

**Interviewer:** Mmmh, so what really happened for you to consult an elder in your first menstruation, why didn’t you consult your friends?

**Respondent:** I was really afraid about it because I had never experienced that, so I wanted to seek guidance pertaining to this.

**Interviewer:** mmmh, so whom did you approach?

**Respondent:** I approached my mother

**Interviewer:** Your mother only, after telling her what happened next?

**Respondent:** She told me the way I could take care of myself during this time.

**Interviewer:** Okay, was there any special ceremony organized to advise you concerning this be it initiation ceremony or whatever?

**Respondent:** No, there wasn’t any.

**Interviewer:** Only her, she didn’t invite may be someone else to advise you?

**Respondent:** No

**Interviewer:** (Long pause), as of now do you have any concern or any questions you would like to ask for you to know further like these issues?

**Respondent:** No, I don’t have any.

**Interviewer:** mmmh, okay, now I just want to know about relationships that exist between boys and girls. When was your first time you ever heard that a boy and a girl can be in such relationships?

**Respondent:** Like you mean boys and girls….

**Interviewer:** yeah like being in a relationship?

**Respondent:** Yeah, it happens like being in a relationship like just as a friendship….

**Interviewer:** mmmh, how frequent in this community for girls to be in a relationship?

**Respondent:** There are a lot of girls.

**Interviewer:** Do you have boyfriend?

**Respondent:** Yeah, I have only one.

**Interviewer:** mmmh, what’s your aim of being in this relationship?

**Respondent:** I just want to be chatting with him that’s all.

**Interviewer:** So, how do you differentiate that, this is my boyfriend and this one is not, since you interact other with boys, like in this community, at school and many other places?

**Respondent:** It depends on the way we relate or interact then we distinguish… either this one is my boyfriend or not.

**Interviewer:** How does it differ? Like how do you interact when he is your boyfriend?

**Respondent:** Like in a relationship we can be engaging in sexual intercourse unlike in mere friendship where we just chat.

**Interviewer:** So, do you have a sexual relationship with your boyfriend?

**Respondent:** Yeah

**Interviewer:** Mmmh, okay, have you ever discussed with your boyfriend anything pertaining to HIV?

**Respondent:** No, we have never discussed

**Interviewer:** Why?

**Respondent:** Nothing.

**Interviewer:** What about issues concerning getting pregnancy?

**Respondent:** We discussed this issue of pregnancy, so we use a condom when doing sex.

**Interviewer:** Mmmh

**Respondent:** So, that we can prevent pregnant.

**Interviewer:** How long has your relationship lasted?

**Respondent:** We are now in the second month since it started.

**Interviewer:** It’s your first boyfriend or you ever had another one?

**Respondent:** No, it’s my first one.

**Interviewer:** So, for how many times have you done sexual intercourse within these two months with your boyfriend?

**Respondent:** Twice.

**Interviewer:** So do you have a purpose for this relationship?

**Respondent:**  Yes, will marry each other in future.

**Interviewer:** How old is your boyfriend?

**Respondent:** He is 18

**Interviewer:** Is he schooling?

**Respondent:** Yeah.

**Interviewer:** What level is he?

**Respondent:** He is in standard 8.

**Interviewer:** standard 8?

**Respondent**: yeah.

**Interviewer**: Are you at the same school?

**Respondent:** No.

**Interviewer:** mmh.

**Respondent:** We are in different schools.

**Interviewer:** What level are you?

**Respondent:** 6.

**Interviewer:** Okay, what are the benefits of being in this relationship?

**Respondent:** (laughs), like the benefits?

**Interviewer:** Yeah

**Respondent:** (laughs), sexual intercourse.

**Interviewer:** Oooh? (Laughs), its only sexual intercourse? Okay, its only sexual intercourse that actually made you to be in this relationship?

**Respondent:** …And also, we should be sharing some ideas.

**Interviewer:** So, do you share those ideas?

**Respondent:** Yeah

**Interviewer:** Mmmh, okay,…. what else, I also wanted to know the benefits you have gained from the GG club as compared to before you joined it?

**Respondent:** I have benefited lots of things as I was blank in various things.

**Interviewer:** mmmh

**Respondent:** Yeah, I have benefited a lot because I wasn’t aware of menstruation.

**Interviewer:** mmmh

**Respondent:** And also, how to use condoms, so I have benefited a lot through these things.

**Interviewer:** Mmh mmh, okay how did you join or what motivated you to join the goal girl club?

**Respondent:** I just made the decision to join.

**Interviewer:** You just thought to join?

**Respondent:** yeah.

**Interviewer:** Okay, who approached you and told you about the GG club?

**Respondent:** Margret

**Interviewer:** So what did she say about the GG club?

**Respondent:** She said that girls were required to join the club. There, they learn a lot things like menstruation, issues of HIV and AIDs, pregnancy and lots of things like also how to interact with each other.

**Interviewer:** Mmmh

**Respondent:** That is why we had to join the club and we indeed learnt lots of things.

**Interviewer:** mmmh, okay, so how long have you been in the club?

**Respondent:** mmmmm, it’s been long, I think may be about one year, but it has been long.

**Interviewer:** Okay, apart from learning issues of HIV, menstruation, pregnancy, what else do you benefit from the OC or else maybe if there are any services provided like distribution of family planning methods or being referred to go somewhere else to access those services, do these take place in OC?

**Respondent:** No, I have never seen this happening.

**Interviewer:** Okay, how open do you feel, attending the GG or what challenges have you encountered while attending goal girl club?

**Respondent:** (Short pause), you do you mean… challenges?

**Interviewer:** yeah

**Respondent:** (silent), mmmh

**Interviewer:** What challenges or any disappointment you face as a result of attending the goal girl club whether be it right at the goal girl club or from the community?

**Respondent:** No, challenge

**Interviewer:** Will you continue being a member of these clubs and why?

**Respondent:** Yah, I will still be a member, because I have known a lot of things I was not aware of.

**Interviewer:** mmmh

**Respondent:** Before, we did not know about different games we play there like “kambuzi kalinkhode” and many more, so now I will work hard attending so that I can continue knowing more than these.

**Interviewer:** Mmmh, okay, how can you encourage your fellow girls, who is willing to join these GG club groups?

**Respondent:** I can encourage her to join by explaining to her lots of things I have benefited from this club, like I have known how to interact with friends, talking of menstruation and what a view, so that she can also benefit.

**Interviewer:** Mmmh

**Respondent: Y**ah

**Interviewer:** Okay, thank you for attending this interview, you have actually been a brilliant respondent, do you have any question?

**Respondent:** No, I don’t have.

**Interviewer:** okay, thanks
