# Supplementary material for: Social support and very young adolescent girl’s knowledge on sexual relationships: A comparative qualitative study of Girl Only Clubs’ participants and non-participants in rural Malawi
Source: PLOS Glob Public Health. 2023 Jan 12;3(1):e0001339. doi: 10.1371/journal.pgph.0001339 (PMC10022037; doi:10.1371/journal.pgph.0001339)
Supplement: S1 Data — (ZIP) [file pgph.0001339.s003.zip › Anonymized/IDI5_R2_XX5_Apr.docx]

District: XX

IDI ID: IDI1_R2_XX5_April2019

Location: B

Interviewer: XXX

Data collection date: DD/MM/2019

**Interviewer**: So once again my name is WWW and I work for COM. We are doing this research study in collaboration with PC of the USA. In the study I am talking with various girls ages 10-14 years who are enrolled the GG I Just want to know how young girls like you know issues about family planning, HIV & AIDS and other sexual health issues. Is it through your parents, uncles, friends, cousins, the radio or OC or any other organization. So I want you to be free and to tell me in detailed manner, your experiences like where you first heard about these things, who told you. Mind you there is no wrong answer and this is not an like an exam. Every response that you give will be of great value. So, firstly I just want to know who you stay with?

**Respondent**: I stay with my mother, my father passed away.

**Interviewer**: Okay…., okay, how many children are in your family?

**Respondent**: We are five.

**Interviewer**: Okay, so you are number what?

**Respondent**: I’m the third born.

**Interviewer**: Oh okay, where are you elder relatives?

**Respondent**: The other one is not around.

**Interviewer**: You all stay here with you older siblings?

**Respondent**: Yes.

**Interviewer**: Okay, how old are you?

**Respondent**: I’m 14 years old?

**Interviewer**: Do you go to school?

**Respondent**: yes.

**Interviewer**: Where do you learn?

**Respondent**: YX LEA

**Interviewer**: which grade are you in?

**Respondent**: I’m in standard 8.

**Interviewer**: Okay thanks, mmmmh have you ever been in a situation which forced you to drop out of school?

**Respondent**: Yes.

**Interviewer**: You dropped out of school?

**Respondent**: Yes.

**Interviewer**: What happened for you to drop out?

**Respondent**: Because I was lacking uniform, books, pens and I could not afford school development fee.

**Interviewer**: What happened for you to go back to school?

**Respondent**: I started school again.

**Interviewer**: mmh, what happened for you to go back to school again since you were facing some financial challenges?

**Respondent**: I learnt here at OC the importance of school, so I decided to start again.

**Interviewer**: So, how did you solve the problems you were facing like lacking school uniform and books?

**Respondent**: I haven’t found the school uniform yet, and sometimes, they send me back because of lack of development fund fee.

**Interviewer**: Okay mmmh (silent), I also want to know what you do when you have finished your household chores like cooking, sweeping, at your free time, who do you like chatting with or where do you like spending your time?

**Respondent**: I like chatting with my fellow friends..., girls... or I go to OC club.

**Interviewer**: So, when chatting with your friends’ what stories do you discuss as girls?

**Respondent**: We discuss about how to prevent contracting sexual transmitted diseases and about family planning.

**Interviewer**: So, what do you discuss as girls on your own?

**Respondent**: We discuss (laughs) things like ….

**Interviewer**: mmmh, I want that you should to tell me when was the first time you have ever heard or discussed about these issues like HIV and AIDS?

**Respondent**: We discussed that... (Silent)… when having sex we must be using a condoms to avoid contracting HIV and getting pregnant.

**Interviewer**: mmmh

**Respondent:** mmh

**Interviewer**: Mmh, so how did you know about this?

**Respondent**: We learned at OC club.

**Interviewer**: Any other place?

**Respondent**: On radio.

**Interviewer**: Okay, any other place?

**Respondent**: Even parents do tell us.

**Interviewer**: Have you ever discussed that with your mother?

**Respondent**: mmm no.

**Interviewer**: So, by parents what do you mean?

**Respondent**: Other women.

**Interviewer**: Okay, do they tell you?

**Respondent**: yes.

**Interviewer**: Okay, so, you also said that sometimes you go to chat with your friends, are those places safe? And have you ever felt insecure at such places or sometimes you just feel insecure, I just want to know that you should tell me a moment when you went chatting or sports where you felt not being protected or maybe boys insulting you.

**Respondent**: silent

**Interviewer**: what challenges do girls face in those places?

**Respondent**: Where we play?

**Interviewer**: Yes ?

**Respondent**: Any challenges?

**Respondent**: Yes, one day a guy when I went to play netball tempted me.

**Interviewer**: How did he tempt you?

**Respondent:** He told me to have sex with him without using a condom, but I refused that I don’t want to contract HIV & AIDS and getting pregnant.

**Interviewer**: Was the guy your boyfriend or just any other person?

**Respondent**: My boyfriend.

**Interviewer**: mmh, so you have a boyfriend.

**Respondent:** yeah

**Interviewer**: Okay, how long have you been in this relationship?

**Respondent**: for three months.

**Interviewer**: is he your first boyfriend?

**Respondent**: yes.

**Interviewer**: Okay, so how many times did he try you?

**Respondent**: once.

**Interviewer**: So did you yes to him?

**Respondent**: what!!?

**Interviewer**: Did you sleep with your boyfriend?

**Respondent**: Yes but once.

**Interviewer**: Okay, and you used a condom?

**Respondent**: Yes.

**Interviewer**: Okay, okay, so other boys apart from your boyfriend who ever tempted you or doing any harm in any way?

**Respondent**: Some guys say if I don’t accept their proposal, they will beat me up.

**Interviewer**: Okay, but your boyfriend, didn’t he force you to end up being in relationship with him?

**Respondent**: No, he didn’t force me.

**Interviewer**: Why did you accept him? And why did you get involved in this relationship?

**Respondent**: (silent)

**Interviewer**: There must be a reason?

**Respondent**: Yes.

.

**Interviewer**: Why did you end up being in a relationship?

**Respondent**: Because I wanted financial assistance from him.

**Interviewer**: Asking things like what?

**Respondent**: Things like soap when I don’t have.

**Interviewer**: Does your boyfriend work or does he do a certain business, or generally what does he do?

**Respondent**: He goes to school.

**Interviewer**: What grade is he in?

**Respondent**: He is in form three.

**Interviewer**: How old he?

**Respondent**: He is 16 years old.

**Interviewer**: 16, but he is also your fellow student where does he get assistance?

**Respondent**: From his parents.

**Interviewer**: Okay, but does your mother know that you have a boyfriend?

**Respondent**: No, she doesn’t.

**Interviewer**: Okay, concerning issues about HIV & AIDS have you ever discussed it with your parents or any adult?

**Respondent**: Yes.

**Interviewer**: You discussed it with who and what happened to find yourself in that discussion?

**Respondent**: They were telling us that there is HIV, so girls like us should be careful and avoid contracting it.

**Interviewer**: So, what happened did they just start telling you?

**Respondent**: They told us that some people are born with HIV, so we should be careful when doing relationships, and we should be going for testing.

**Interviewer**: So they just started telling you that some people are born with HIV, tell me what really happened for them to start advising you?

**Respondent**: They just started telling us.

**Interviewer**: When saying “they just started telling us”, who were you with?

**Respondent**: I was with my friends.

**Interviewer**: Who was telling you?

**Respondent**: women.

**Interviewer**: women from this village?

**Respondent**: Yes.

**Interviewer**: Okay, what about issues of pregnancy, like how one can get it, who have you ever discussed with?

**Respondent**: About that I just listen to the radio.

**Interviewer**: You just hear it on radio?

**Respondent**: Yes.

**Interviewer**: What about at school?

**Respondent**: We also learn about it.

**Interviewer**: Okay, (silent) so who is your close friend? Do you have one?

**Respondent**: Yes, I do.

**Interviewer**: Okay, so tell me, have you ever been in a certain situation like a fix and you told your friend about it?

**Respondent**: Yes, I told my friend about it.

**Interviewer**: Tell me what really happened?

**Respondent**: And she helped me with a solution.

**Interviewer**: So, tell me what happened?

**Respondent**: mmmh

**Interviewer**: please be open girl, tell me.

**Respondent**: When I started my first menstruation, I told my friend, so she advised me that I should be dressing properly so that others should not notice.

**Interviewer**: Why didn’t you tell your parents or any adult?

**Respondent**: (silent)

**Interviewer**: Was there a reason?

**Respondent**: yes.

**Interviewer**: tell me.

**Respondent**: My parents were away by then.

**Interviewer**: Then you told your friend, why didn’t you tell an adult like a neighbor?

**Respondent**: It was hard to tell them.

**Interviewer**: Why?

**Respondent**: Some neighbors cannot keep a secret.

**Interviewer**: Okay, then what did you do to let your parents know?

**Respondent**: When they returned from where they went, I told them.

**Interviewer**: What did they do?

**Respondent**: They advised me.

**Interviewer**: Okay, before you ever heard of HIV & AIDS at places you told me like from friends or at OC club, what did you think HIV & AIDS was?

**Respondent**: I was thinking that…

**Interviewer**: Or to say what questions did you have about HIV & AIDS?

**Respondent**: I had questions like when one is positive, some people say that when you are not taking medication you might die. So I decided to abstain.

**Interviewer**: Before you knew about it, when you were just young, what did you think HIV was? If you are talking about drugs it means it was after you heard about it.

**Respondent**: I was thinking that HIV & AIDS is just a disease.

**Interviewer**: Okay, (silent) when a person is growing up the body starts developing breast and pubic hair, by the time this was happening to you what kind of questions did you have? Or by then you had already discussed it with someone before…, and what were you thinking?

**Respondent**: First I saw it on my friends’ body, they started growing breasts.

**Interviewer**: mmmmh

**Respondent:** So, I was asking myself (laughing).

**Interviewer**: Don’t be shy explain please.

**Respondent**: I was asking myself “I am I going to have to develop breast like that as well”?

**Interviewer**: mmmh, were you admiring to have breast as well or you were scared?

**Respondent**: I was scared.

**Interviewer**: What were you scared of?

**Respondent**: I was afraid that I was going to develop big breast while still young.

**Interviewer**: What were you scared of then if you were to have big breast?

**Respondent**: (laughs) I thought the breast could be heavy on me since I was that young.

**Interviewer**: Did you ever discussed with friends like those who already had some breast?

**Respondent**: Yes.

**Interviewer**: What did you discuss?

**Respondent**: By then we were showing one another if we developed breast and laugh at one another.

**Interviewer**: okay, what made you to join GG club?

**Respondent**: It made me to know a lot of things.

**Interviewer**: mmmh, at GG club?

**Respondent**: yes.

**Interviewer**: What did you know?

**Respondent**: I learned about sexually transmitted infections and how to abstain from contracting them. (Silent)

**Interviewer**: Anything else you learnt at goal girl club?

**Respondent**: (silent) and also about how to strengthen the club.

**Interviewer**: By strengthening the club, what do you mean?

**Respondent**: Being people who are united… (Silent), being people who can be united and doing things together.

**Interviewer**: mmmh (silent) what happened for you to know that there is a group called GG Club and who motivated you to join?

**Respondent**: I heard it from friends that there is a OC club and I joined.

**Interviewer**: Okay, what really motivated you to join the club?

**Respondent**: I wanted to know more.

**Interviewer**: Okay, what have you benefited since joining the club?

**Respondent**: I have learned how to abstain from sexually transmitted diseases and how to strengthen a club.

**Interviewer**: What can you advise any girl who is interested to join the club?

**Respondent**: I can teach her, (silent). I can teach her about menstruation and HIV & AIDS.

**Interviewer**: Okay, I meant that can you encourage her to join the club?

**Respondent**: If she is really interested, she can join.

**Interviewer**: Okay, thank you for answering the questions this is what I had for today.

**Respondent**: okay.

**Interviewer**: thank you.
