# Supplementary material for: Social support and very young adolescent girl’s knowledge on sexual relationships: A comparative qualitative study of Girl Only Clubs’ participants and non-participants in rural Malawi
Source: PLOS Glob Public Health. 2023 Jan 12;3(1):e0001339. doi: 10.1371/journal.pgph.0001339 (PMC10022037; doi:10.1371/journal.pgph.0001339)
Supplement: S1 Data — (ZIP) [file pgph.0001339.s003.zip › Anonymized/IDI6_R1_XX6_May.docx]

**INDEPTH INTERVIEWS WITH 10-14 YEAR OLD GIRLS**

District: XX

IDI ID: IDI6_R1_XX6_May2018

Location: B

Interviewer: WWW

Data collection date: DD May, 2018

I: Now as I earlier said, I am WWW and I have come from COM in Blantyre; we are working together with OC. The issue is that we want to understand how OC clubs are going in this village, for young girls like you. And also I should know more about your daily life; that how is your life like?

P: Okay.

I: So as a girl who is growing up in this village, I wanted to know that your day, most of the times, how does it go like. What you do on a day, when you wake up in the morning, what do you do? Or whether in the morning, what do you do? Where do you like to chat? That’s what I want to know. How do you use your time? So can you explain to me that when you wake up in the morning as a girl, what do you do here?

P: When I wake up in the morning, as a girl,

I: Yes,

P: I go to the bathroom, and wash my face; as by the rules. When I get out, I do household chores; I cook food. And I find time that I should just rest for a while. When I rest for a while, I get back to the kitchen, since I am the only girl, so I go and cook for the parents to eat.

I: Yes,

P: And then when I cook, I also need to prepare them water so that they should bath. And when they bath, they tell me that now that we have bathed, you should also bath; and find time to go and play or either you will sit on the bench and sell.

I: Okay.

P: So that is during the afternoon and morning hours.

I: Okay.

P: In the evening, they help me in cooking. In fact in the evening; I take a bath two times a day; since as a girl, she cannot take a bath once in a day.

I: Yes,

P: And when I stay, I ask myself,

I: Yes,

P: What if I face a problem that maybe it has happened to me like how people have their monthly periods, what would I do?

I: Yes,

P: So when it happened to me, I explained it to my parents. After I explained, they told me how I can take care of myself and cleanliness. So I tried my best to use sanitary pads and now I use them.

I: Okay. Now here you have talked a lot of issues; what you do when you wake up in the morning.

P: Yes.

I: At your home, how many are you there?

P: We are five people.

I: You are five people?

P: Yes.

I: The five people including who and who?

P: There is me,

I: Yes,

P: My older sister, the siblings at my home; three boys,

I: Yes,

P: Mum and Dad, making it seven,

I: Yes,

P: Altogether as family, right?

I: Yes. All of you sleep in the same house?

P: Mother sleeps; we sleep in that small one there. The one you found people seated on.

I: But you explained as if you cook everything on your own, why?

P: Yes I cook. In the morning I cook, in the afternoon I cook. In the evening it is when they help me because my friends are in secondary school.

I: Your older sisters?

P: Yes.

I: You, you don’t go to school?

P: I go. But today I am absent because I have a nose bleeding problem.

I: Okay. So here you talked about monthly periods saying you had questions that when you start having monthly periods, how will it go like? Whom did you ask?

P: My grandmother,

I: Yes,

P: Who is an elderly person.

I: Mmh?

P: Yes.

I: How was it? Who told you to ask your grandmother?

P: Nothing. We were told that, if you have a big problem that you have never faced before, you need to meet an elder person and explain it to them. Or maybe an older girl that you think can tell you about the future, find her and explain to her.

I: So who is it that told you that? That advice?

P: The parents.

I: Okay. So I wanted to know that in this village as you live, the other person whom you feel comfortable with, mostly your issues; or that you have been faced with problems like how you said big problems, who is it?

P: That is YYY,

I: Yes,

P: And the owner of that house there. But it’s a woman.

I: Okay. YYY’s inlaw?

P: Yes.

I: She is the one who tells you?

P: Yes.

I: Okay. So you ask her all questions?

P: Yes. she asks me. If she says she doesn’t know the rest, then I go and kneel to my parents, saying “mother, I have a question,” and she explains to me that do this and do this, you will find an answer.

I: Okay. I want to know that questions like what? Just for example.

P: For example like maybe a person is asking me to be his girlfriend and I am refusing him, right?

I: Yes,

P: He is being disturbing me. To listen to my questions, he isn’t listening but continue blocking me on the way. So I ask people saying “on that, for me to walk; how should I walk?” because when I go this way, this person is blocking me. How should I walk? So they told me that, if he touches you; you should act to be on your own and be in front. If he touches you, you should scream. And then I did those things and they came. And they advised him; it ended. And also during monthly periods, I did not know. And also that the other name is monthly periods, I did not know. For me, I learnt it from the book. And when I saw it happening to me, I stayed for two days; afraid, saying “should I tell them? Or whom should I tell? Won’t they say that I’ve gotten hurt or feel embarrassed?” I tried in one way or another but I failed to take care of myself. It was discovered and then people advised me as to how it is.

I: It was discovered, how did they see you?

P: They saw me because my dressing; you know I am a child, right?

I: Yes,

P: I didn’t know. I wore a short skirt which was white. And then the blood stained on it. I just wore with a pant inside. And the blood stained on it, when it stained, people asked me, “saying what is that?” and then they called me “this needs to be hidden, not everyone should see it. Yes people go through it. But it is not how you are doing it; you are wrong. You should wear like this and this.” So those instructions I follow them up until today.

I: Okay. The other counseling that you received in that time, what was it?

P: From parents?

I: Yes. when you started having monthly periods, when the elders took you and saw you that you have started having monthly periods. Didn’t they take you privately to counsel you?

P: They took me. And they told me that, “it is necessary that when I am cooking any type of food I should not add salt to it. A younger child should do it instead.

I: Alright. The other thing that they advised you?

P: They said that my movements with boys; I should discipline myself. I should say that just because they are boys, right? I should be chatting with them like how I do with girls; No.

I: Yes,

P: And also I need to protect myself from the boys not just chatting anyhow with them. Or when they tell me something, I just agree; No.

I: So they said you should not do that, why?

P: They said that I should not do that because in the future, I might get pregnant. Or sexually transmitted diseases.

I: Okay. The person who gave you this counseling, who was it?

P: My fellow girls. For example the one I mentioned Eneless.

I: She is the one who advised you?

P: Yes.

I: What about your parents. Your mother, what part did she play during your monthly period?

P: She played; she’s the one who told me that “salt, don’t add it when you are alone, you need to call someone aside. And also what to wear during your monthly period; if I have money, I will buy you small purity sanitary pads. But if I don’t have money, you should use these rags.

I: Okay. That was your mother?

P: Yes.

I: What about on the part of sexual intercourse or issues dealing with the disease of HIV? With your mother have you ever talked about this issue before?

P: No. she has never told me.

I: She has never told you?

P: Yes.

I: What about your father?

P: Umm, my father I fear him.

I: You fear him? Has he never talked to you issues like these?

P: No.

I: Advising you things like these?

P: His advice he just says, you girls, you need to protect yourselves. And also I cannot talk more to you, right? Because when I talk to you, you say “it is not my part.” Other people’s rudeness nowadays they say, “It is not my part, you don’t need to talk to me.” So from then onwards, our Dad does not talk to us much. But our mother is whom we feel comfortable with.

I: Your mother is the one you feel comfortable with?

P: Yes.

I: But you have said with your mother you have never talked about AIDS, right?

P: Yes. About AIDS, she has never told me.

I: What about sexual intercourse?

P:

I: Alright. Now do you have an Aunt?

P: Yes I have.

I: Them, on issues like these, what part do they play?

P: Umm, I shouldn’t lie, my aunties are in far places. The ones I have near me are my uncles.

I: Your uncles?

P: Yes.

I: Okay. So them, don’t they play any part?

P: Umm, them too are busy people, so to find time to advice me; it is scarce.

I: It is scarce?

P: Yes.

I: Now as you earlier said, where you find counseling on issues like these it at YYY’s place?

P: Yes.

I: Your friend?

P: At My friends place Eneless….

I ….Or at YYY’s in-law’s place?

P: Yes. At YYY’s in-law’s place

I: Okay. And your other friends’ place?

P: Yes. others maybe when we meet at school, Yeah.

I: It is where you discuss issues like these?

P: Yes.

I: Alright. Now in this village, where else is it that girls like you age can go? Apart from the relatives places, where else is it that girls go for counseling concerning issues of HIV/AIDS, or issues of how to prevent pregnancies, or that you should get sexually transmitted diseases? Where else do you maybe hear……

P: … At that place there [pointing] there is one girl.

I: Which one girl is that?

P: Beatrice, she uses that name. Her name is CCC. But us in this village, the name we know is BBB.

I: Okay. BBB; what is her role?

P: Her role is….

I: …That she should be found counseling girls?

P: Maybe her too at her home they tell her and it is what she hears that that she tells us.

I: Did she go to some kind of maybe special training?

P: No. she is here in primary school.

I: Okay. She is a student?

P: Yes. she is our fellow student.

I: So she is able to counsel you?

P: Yes. what she knows, she shares with us, us too what we know, we share with her.

I: Okay. Now I wanted to know that as you live in this village; girls, you have said that you go for a chat whether it is at CCC’s place. Sometimes you go and play netball, and others places for chats. Your care as girls, how well is it? Or your security? Because sometimes I know there are activities, maybe girls are being abused.

P: Yes, it happens.

I: How often does it happen? I wanted to know how it is here for girls? For young girls especially like you? As you walk. Whether in the homes it happens that girls sometimes are being abused. I wanted to know that here, how often is it? Or maybe you, You should tell me what you have been witnessing in your life as a girl who is growing. Abuses that maybe have been done to you; sometimes it is difficult to report to people.

P: Aaah, me…..

I: As I said earlier, fell free, right?

P: Yes.

I: Don’t hide anything else.

P: Abuses at my home?

I: Yes.

P: No. to say the truth, there is none.

I: Okay. What about your friends? Your fellow girls?

P: Yes. some are abused. Telling them that when they have started having monthly periods, “you need to find a man; I am tired of feeding you.” So issues like those, it is found that the girls are getting pregnant while they are young. Being forced by parents.

I: Okay. Alright. Have you ever gone for a girls initiation ceremony?

P: Yes.

I: Have you been ever been initiated? While you were young?

P: Yes, while I was young. And also when I was a child.

I: Okay. Now can you remind me some of the counsels that you learnt there?

P: At the girls initiation ceremony?

I: Yes.

P: I will just lie to you. The initiation I was initiated in, I cannot remember, I was a child you know. And also here there is no child who is initiated as a virgin, right.

I: Umm.

P: The people say that that time they used to bribe me with biscuits [laughs]. I was going outside, wanting to go and live with my mother at home. But I didn’t know.

I: Okay. Because you were very young?

P: Yes.

I: Umm. Alright. What about right now, I wanted to know; do you maybe have some questions that you would like to know; Ooh! There was this other issue you talked about, saying sometime back; “I wrote it somewhere so that I should remember.” Some time back, a certain boy was disturbing you, asking you to be his girlfriend. Can you explain properly to me on this issue; how did it go? You were saying that he was blocking you on the way.

P: Yes.

I: Yes. I want to understand properly. A boy from where? There was a boy who was disturbing you, asking you to be his girlfriend?

P: Yes.

I: So how was it?

P: Umm. I don’t know where to start from [laughs]

I: No. anywhere is a starting point.

P: Okay. They say when people have started having monthly periods, people show signs. And also when you are a girl enough, they say boys recognize signs on you. So maybe when he looked at my body he found the signs, that was when he was asking me to be his girlfriend. Now for me, not wanting to agree with him, I refused him. That was when he started blocking me on the way. But the sign on me that boys saw was that “look at her legs growing big, on the hips there.” So I thought they were just joking, but still my other friends were busy with it up to the point of falling for it. But for me, No. I tried in my way up to that it ended. And as of now, we don’t talk; me and that boy. We just pass each other.

I: Umm. It was only one boy?

P: Yes. One boy.

I: So he says he was seeing your hips have grown?

P: He said hips growing and also legs, they said looking beautiful.

I: They were admiring your legs looking beautiful?

P: Yes.

I: So they were blocking you on the way?

P: Yes. blocking me on the way; maybe I’ll use this way from coming to school, they were busy; when I am coming from school, when I’m with friends, they just pass me by; just swore and passed. Just passing each other without talking. But when I am alone, “it is you, I have found you, you were acting up, right?” I would run away in the bushes and get here.

I: Mmh?

P: Yes.

I: Okay. Is it the only boy who has ever asked you to be his girlfriend?

P: Yes, he’s the only one.

I: Have you ever had a boyfriend?

P: Me?

I: Yes.

P: Yes, the boyfriend is exists.

I: Right now the boyfriend exists?

P: But right now I ended it with him because my mother discovered me and when she discovered me and shouted at me. [laughs] so she said I should never see you again at your size having a boyfriend. I should never hear it again. Or I’ll figure out what to do with you and I’ll take you to the police with that your boyfriend you are chatting with.” So then I just said “You, let us break up, everyone should go their separate ways.” So now I am just staying.

I: Okay. Where is the boyfriend from? Where was he from?

P: From XX there.

I: Why did you have a boyfriend? Not that I am saying it s wrong, I just want to know, that what is it that makes girls to have boyfriends.

P: [laughs]. I did it forcefully because you know my friends, right?

I: Yes.

P: When they had money, they used to say “you are struggling for money? But you have a body, why not use it?” it was when I knew that so these people are finding money through using the body?

I: Mmh?

P: Yes.

I: So when he asked you to be his girlfriend, you just accepted?

P: After a week, he kept on passing through my home here, passing, passing. Then on the third week, it was on a Wednesday, that’s when I accepted him.

I: Mmh?

P: That it should be a relationship. So while it was a relationship like that, it happened that there was; umm there was what? There was a certain celebration, a dance. So I went. So the time I was advised home to return back and the time I came back was different. So they told me that you cannot be courage enough to come back home at this time. There is something that you are relying on. And also people from his place when they searched, they found it. Also my mother visited her friends and also my friends whom I chat with and they said, “nothing more, she has a boyfriend.” So that was where the story started. Up to the point that the relationship should end.

I: Okay. Now, you; in that relationship, what were you benefiting?

P: Aah, I shouldn’t lie, there is no benefit. If it is a benefit, then it is the money that they find there; they say it is a benefit. But there is no serious benefit.

I: You, have you ever been given money by this boy?

P: Yes money I was given. One time.

I: Like how much?

P: 3500.

I: Eeh, it is a lot, right. [laughs]

P: [Laughs] No. it is a little. When you start using it, that’s all, it has ended.

I: So what did you do with it?

P: I just spent it.

I: What did you buy with it?

P: I just went to the China shop, I just bought a dozen of purity sanitary pads, brought home and kept it. I bought body lotion, and other under wears, panties. Yes. and then realized that the money has finished.

I: Okay. So when he gave you money, wasn’t he expecting something that you will give him something?

P: No. even when it came to being clever, I was the one making him clever. [Laughs]

I: Okay. Didn’t he ask you that since he has given you money, then he will also want something in return?

P: Maybe it is possible he was thinking, right?

I: Yes.

P: Maybe it is possible he was thinking like that, but because I rushed in ending the relationship; it didn’t even last a month. It was when the relationship ended.

I: It ended very long time ago?

P: Very long. I would say this is the third month.

I: Okay. So you don’t ever meet?

P: Even when we meet, we just greet each other, then pass each other.

I: Okay. Alright. While on this same issue of sexual relationships between boys and girls, You, what are your views? Would you encourage other girls to be involved in these relationships? Why is that? and also if you cannot encourage them, other girls, what would you advice them? On the issue that they should have boyfriends and be having sexual intercourse. These are your views, don’t take into account that I have come from OC, but I want to hear your views. Do you see that it is good or not?

P: For me it is bad.

I: Why?

P: Because when you have a boyfriend, and be forced to have sexual intercourse, you should get ready to get pregnant or get sexually transmitted diseases. So not wanting to have these, its better to just not have a boyfriend and just live your live as you were, a health life.

I: Mmh?

P: Yes. But still some engage in sexual relationships and live a healthy life, Aaah; there is something that they use.

I: But I thought there are condoms?

P: Aaah, condoms can burst. With how I read the book, it is not good also. Abstinence is good.

I: Abstinence is good?

P: Yes.

I: Okay. Now, how common is it that in this village girls should be found that; girls like you size, being found that they are married, how common is it?

P: How common is it? Because of abuses that fall on them from parents, they just do as their heart says. Because if a person is forcing you to get married, you need to find somewhere you can go to report issues like these. But them when they are faced with such, some are those who already do such behaviors so they just say “isn’t this the chance.” And continue with it up to the point that at my age, they are married.

I: They exist girls like those, who are married?

P: Aaah, no need to ask.

I: At your age?

P: Maybe I am also older. Maybe 12 years old.

I: So how is it for them?

P: Nothing; just getting the husband at home; he should feed the parents, the husband, wife and children. He should feed them together.

I: What ages are the husbands?

P: Same ages.

I: Bothe children?

P: Both children.

I: Okay. So what problems do they face?

P: For example lacking of social support in their life.

I: Yes,

P: Food, here and there. It happens that a person if you were okay while with your parents, you start looking stunted; your body. Saying “it was better if they had stayed at their parents home; they would have looked otherwise.”

I: Mmh?

P: Yes.

I: Alright. So you are in school?

P: Yes. I am in school. I went back to school.

I: At first you had stopped going to school?

P: Yes I had stopped.

I: What is it that made you to stop going to school?

P: What made me to stop going to school?

I: Yes.

P: I fell sick for a whole month. The second month I went to the hospital. It lasted three months while I was still sick and I came here at home. Now when I wanted to go to school, I was afraid that what if my friends ask me, “What will I say I was suffering from? Or in my life, won’t they see that I have changed in appearance?” through those burdens I just decided that I should not be going to school and I stayed. I saw that school has an importance. All my friends here were going to school and I was left alone. I said “iih, this is also a burden.” I told my mother, “mother, I want to go back to school.” My mother said “what made you think that way?” “Nothing, most girls who dropped out of school are going back to school. Me too I need to go back to school.” Then she approved me, “go and start.” So I stopped when I was in standard five. And right now I am in standard eight.

I: Okay. So what are your thoughts? About your future?

P: I need to continue with my studies. When I continue with my studies, there is something that I wish in my life to fulfill it.

I: What do you wish to be? What do you wish to fulfill?

P: The two things that I wish for?

I: Yes.

P: A nurse, and a journalist.

I: A nurse or a journalist?

P: Yes.

I: Okay. Now how was it possible that you should be found that you have joined this program of OC? Of girls?

P: When I had stopped going to school, because I had nothing to do at home, when it was morning; the sun would set while I was at home.

I: Umm,

P: So when this organization came, our treasure is the one who came to pick us saying “there is an organization, don’t just stay here at home. What you would have been learning at school, you will be learning them here at home. So not to waste time, this organization wants children between 10 up to; he said whether 14.” So I asked him that “now above that age there is none that a person should join?” and he said “there is no problem, as long as you are learning.” So I went there and started learning. Learning and learning. Then it took me time to discover that “what are they talking about?” But after a short while since a literate person is a literate person, I always stayed closer to the book, that’s when I learnt how to read and other stories, understanding them that “what are these stories talking about.”

I: Okay. So, right now, what part do you play? In this group?

P: Of OC?

I: Yes.

P: Learning what they teach, for example like strength, there is also abstinence or I should say getting umm would I say early pregnancies?

I: Yes.

P: Yes, they are there. So issues like those, they are what make it to be specially an advice. Even if no one gave you advice, but if you get hold of that book, it gives advice to different people.

I: What about are there any other activities that happen there at OC? That you also do?

P: Yes. Physical exercises.

I: Like what?

P: They sing, people jumping up and down; just like how it is on a celebration, right?

I: Umm.

P: Yes. It happens.

I: Okay. Your life, how would it say it has changed ever since joining this group of OC?

P: My life has changed?

I: Umm. Benefiting, how have you benefited?

P: Yes. it is better you ask me like that. [Laughs]

I: [Laughs]

P: My life has benefited, for example like, strength; I did not know that it is in how many groups. I just said strength is strength.

I: When you say strength, I also want to understand. What do you mean?

P: On strength?

I: Yes.

P: [Silence]

I: Can you tell me maybe the groups that you have learnt.

P: There is strength “WITH”,

I: Mmh?

P: There is strength “WITH”,

I: Yes,

P: There is strength “on”, strength “AT”; Inner strength.

I: Okay. So can you explain to me, when you say strength “ON”, what does it mean?

P: Strength “ON” is a person who regards himself as “I am me.”

I: Umm. Strength “AT”?

P: Strength “AT” is a person who is able to do things on his own in trying to teach his friends.

I: The other strength?

P: Strength “WITH”, are things that you cannot do on your own.

I: Okay. I also did not know, you have taught me.

P: [Laughs]

I: I have never heard it before.

P: [Laughs] you know it.

I: No. I don’t know.

P: Aaah.

I: [Laughs]

P: [Laughs]

I: I didn’t know. I have just heard it from here. Alright. Now the question I had prepared; Okay, the last question is that other girls what would you tell them? Those that have not yet joined the clubs, what would you tell them?

P: I would tell them that they need to go to this club so that. [silence]….Umm but people from here, disagree with Non Governmental Organizations.

I: Umm.

P: So even telling them, it might be understood but aside they gossip about you.

I: Mmh?

P: So just that I should be busy telling them? Aaah, there are a few whom I connect with to tell them about that.

I: Okay. Alright. Anyway, thank you very much because of your answering of these questions. You have answered very well. You were comfortable. Maybe you have a question….

P: Not that I was uncomfortable?

I: [Laughs]. Do you have a question?

P: Umm. A question was there.
