# Supplementary material for: Social support and very young adolescent girl’s knowledge on sexual relationships: A comparative qualitative study of Girl Only Clubs’ participants and non-participants in rural Malawi
Source: PLOS Glob Public Health. 2023 Jan 12;3(1):e0001339. doi: 10.1371/journal.pgph.0001339 (PMC10022037; doi:10.1371/journal.pgph.0001339)
Supplement: S1 Data — (ZIP) [file pgph.0001339.s003.zip › Anonymized/IDI6_R2_YY6_Apr.docx]

District: YY

IDI ID: IDI6_R2_YY6_Apr2019

Location: A

Interviewer: WWW

Data collection date: DD/MM/2019

**Interviewer:** As I explained earlier, I just want to know how girls in this village know about HIV , pregnancies, relationships between boys and girls in this area and how you have benefited from GG Club. Firstly, tell me do you go to school or you don’t?

**Respondent:** Yes, I go to school

**Interviewer:** Which class are you?

**Respondent:** Standard 5

**I:** Which school do you go?

**R:** CC

**I:** You learn at CC?

**R:** Yes

**I:** Whom do you stay with?

**R:** My Mother and my Father

**I:** Do you have siblings?

**R:** Yes

**I:** How many?

**R:** A lot

**I:** How many girls are you?

R: At my mother’s house?

I: Yes, your mother, you and your siblings

R: We are 3 girls

I: How many boys?

R: 2

I: Who is older in your family?

R: He is a boy X

I: How old is he?

R: He is 17 years

I: Ok, what do you like to do when you are not doing household chores, maybe you have finished sweeping the surrounding. You have cleaned the dishes, you have drawn water and you have taken a bath?

R: I like studying

I: You like studying?

R: Yes

I: What else do you do apart for studying?

R: If it’s the day to go to OC then I go.

I: If it’s not the day to go OC?

R: Then I go to play

I: Mmh, ok, what are the challenges you encounter when you go to play or to one community? Let me ask this way, what are the challenges girls encounter when they go to play?

R: They encounter a lot of challenges like boys proposing to them.

I: What else?

R: Quarrelling with friends

I: On being proposed by boys, has it ever happened to you?

R: Yes

I: What do you say?

R: I refuse

I: How often does it happen?

R: It happens often, the moment I go out eeh

I: What do they say?

R: I want you to be my girlfriend but I tell them that am still young to do that.

I: Oh, Ok why do you refuse?

R: Because I know there is no benefit from that.

I: Ok, Tell me about how you first heard about HIV?

R: Time?

I: Explain to me the first time you heard about HIV, where were you? Who explained to you about HIV/AIDS

R: OC

I: OC?

R: Yes

I: Was it your first time to hear about it? You never heard about it somewhere else?

R: Yes

I: What did they say is HIV?

R: HIV is a disease that causes AIDS

I: HIV is a disease that causes AIDS?

R: HIV is a virus that causes AIDS

I an R: Laughing

I: Ok, what else have you learnt at OC?

R: We have learnt how to take care of your body

I: What do you mean when you say taking care of your body?

R: Like washing clothes, taking a bath.

I: What else?

R: And how we avoid pregnancies

I: What did they say on how you can avoid pregnancies?

R: Use condoms, taking injection or loop

I: This is what you learn at OC?

R: Yes

I: What else?

R: Knowing each other

I: Where else do you learn about how to avoid pregnancies?

R: We have the mother group at our school.

I: Mother group?

R: Yes. They call all menstruating girls in a class then they tell us that, they explain.

I: You have mentioned about menstruation, I want to know, before you started menstruation did you hear about it?

R: Yes

I: Where did you hear about it?

R: My friend told me, I didn’t know about it, I asked her how it happens. She said you are about just wait you will do it, every girl when she reaches the age of 15 or 12 does that but I started when I was 12.

I: So she told you that it will happen to you?

R: Yes

I: What questions did you have when you are alone?

R: My parents didn’t explain it to me ,my friend is the one who explained to me, when I started I explained to my friend first before I did to my parents.

I: Why did you explain to your friend first?

R: Because she explained it to me, we keep secrets for each other with my friend, so she is the one who helps me in many things.

I: Then what did you do? You explained to your parents?

R: Yes, I explained to them that this is what has happened to me then she also helped me.

I: What did she say?

R: When we doing this we don’t stay where there are boys, or we don’t stay close to a boy.

I: She didn’t send you to other counsellor to counsel you?

R: She sent me

I: To a different counsellor?

R: Yes

I: Where?

R: To the counsellors

I: When you started menstruation, your mother sent you to the counsellors for counselling?

R: Yes

I: What did they say?

R: They said what my parents talked about.

I: Why did they sent you there?

R: Laughing

I: Or that’s what happens here?

R: Yes

I: They take you to the counsellors?

R: Yes

I: Think of the past, now you know about HIV, pregnancies, and menstruations, what questions do you have? About things that they have never talked about but you want to know?

R: About HIV?

I: Yes or pregnancies, things that you don’t understand and you want to know more. The questions that you have?

R: That question is difficult

I: (Laughs, ) don’t you have question?

R: I do have

I: Like what?

R: People get pregnant when they have sex before they do their menstruation?

I: Mmh

R: So I have been asking myself aaah how people get pregnant… because there was this month I missed my menstruation

I: you thought you are pregnant?

R: Yes

I: You thought you are pregnant while you haven’t slept with a man?

R: Yes, because people say that if you miss a month without menstruating then you are pregnant ,so I was wondering I haven’t slept with a man but I have missed my menstruation so I have been thinking then I just saw that the other month I did on the first date of the month.

I: Why didn’t you ask counsellors?

R: Laughs

I: Were you shy to ask?

R: Yes

I: Relationships between boys and girls in this area how does it go? Have you ever been counselled about this? Were you told about how relationships between boys and girls are supposed to be like?

R: No

I: You have never had counselling about relationships?

R: No

I: Do relations between boys and girls exist in this village?

R: Yes very much, many of them.

I: Why do girls engage in relationships?

R: They want money, they want to be like bosses at school.

I: Do you have a boyfriend

R: No

I: You never had one?

R: No

I: You have never had one?

R: No

R and I: Laughing

I: Why?

R: My parents advised me that it’s not good, I can get pregnant while am still young

I: What made you to join GG Club?

R: I wanted to know more

I: You wanted to know more, like what?

R: Like prevention of AIDS and pregnancy

I: Who told you to join the club?

R: The owner of this house is the one who told me she came to our house and explained it to my parents that I will be going there.

I: How has your life changed since you joined GG Club?

R: It has changed, am protecting my life, even when I explain to my parents they say should not stop going to the club.

I: What has changed in your life? Maybe what you used to do in the past, now you have stopped?

R: They used to ask me only to do household chores but now we learnt that boys and girls have to work equally, so me and my siblings we work equally.

I: What else has changed? Like what it never happened in the past but now it’s happening?

R: That’s all

I: Ok, between your mother and fathers and maybe your sisters whom do you feel open with?

R: My mother.

I: Have you ever discussed about HIV/AIDS with your mother?

R: No

I: Thank you very much for answering my questions, do you have any question?

R: NO.

R
