# Supplementary material for: Social support and very young adolescent girl’s knowledge on sexual relationships: A comparative qualitative study of Girl Only Clubs’ participants and non-participants in rural Malawi
Source: PLOS Glob Public Health. 2023 Jan 12;3(1):e0001339. doi: 10.1371/journal.pgph.0001339 (PMC10022037; doi:10.1371/journal.pgph.0001339)
Supplement: S1 Data — (ZIP) [file pgph.0001339.s003.zip › Anonymized/IDI7_R1_XX7_May.docx]

District: XX

IDI ID: IDI7_R1_XX7_May2018

Location: B

Interviewer: WWW

Data collection date: DD/MM/ 2018

I: As I said earlier, am WWW from COM and I have already explained that we are chatting with girls who are found in GG club OC, to know how these clubs are functioning and how its benefiting girls in this village. And we would also like to know how your daily life goes on as girls on issues of HIV, prevention of STIs, prevention of pregnancy.

So firstly I would like to know how your day goes by as a girl, how you spend your time? For example, when you woke up in the morning what do you do?

P: When I woke up in the morning I do what is there for me to do. But there are a lot of challenges like sometimes I fail to go to school, because I don’t have uniform, and sometimes am sent back from school.

I: Who do you stay with here?

P: I stay with my mother

I: Who else?

P: Just my mother

I: Where is your father?

P: My parents were divorced and my father stays in his own village and I stay with my mother only.

I: Do you have siblings?

P: Yes

I: How many?

P: 5

I: Who is the first one?

P: The first one is TX and am the second born, and the third is GX, and fourth is BX and fifth is AGX and the 6^th^ is DX.

I: So, you all stay together here?

P: Mmm

I: Alright, so what do you do when you wake up in the morning?

P: When I wake up in the morning, I do house chores like sweeping, and other things and if that day they said that those without uniform should not go, then I just stay, with nothing to do.

I: And for the days that you don’t go to school, what do you do?

P: I just stay here

I: You don’t go for a chat?

P: No

I: You don’t have friends to chat with?

P: I do have friends but for me to go for a chat am afraid of some other things

I: What are the problems that you are afraid of?

P: For example, when I go for games, I meet different kinds of people there, a boy might ask for a relationship with me, and if I accept that will be a problem that will result in trouble in future. Since the boy will not just be satisfied with being accepted and leave like a sister and a brother and one day he would want to have sex with me, without protection and this might lead to pregnancy. So this is one problem that I avoid that is why I just stay home.

I: Alright, as a girl in this village, which person are you very open to, who you can chat with on girl issues or any other personal issues?

P: For the girls I should say they were there, and now they have children so now I don’t have that time to be with them to tell them my personal issues?

I: And now, you seem like you don’t have friends?

P: I chat with the same people but I don’t tell them my personal issues, but I just chat with them as friends that’s all.

I: What about the relatives that you are able to share with and open to?

P: I don’t want to lie

I: There’s no one?

P: When I have a problem I tell my mother

I: What kind of problems?

P: When a boy proposes to me I tell my mother that such such boy has proposed to me.

I: What does your mother say?

P: She just looks at me

I: Have you ever heard about HIV?

P: Yes

I: What is HIV?

P: HIV is a disease that causes AIDS while AIDS is different kind of illnesses at one time.

I: Okay, where did you HIV information?

P: I heard it from radio, and I heard about it clearly when I joined OC

I: Alright, here in the village, apart from OC, where else does girls go to find counselling about AIDS?

P: In this village, I think it is the first time to have an organization like this teaching girls about AIDS, I have never heard about any organization coming and teaching HIV and AIDS.

I: What about prevention of STIs and pregnancy, where else would a young girl find advice in this village?

P: Sometimes at school they are able to call elder people to advise girls like us; telling us that maybe sometime you might have unprotected sex and get pregnant and now they tell us on how to avoid it. They tell us that if one indulge into sexual activities might contract HIV or unplanned pregnancy.

I: When girls are growing up have question that they would like to be told by someone. For example, HIV issues, prevention of STIs and prevention of pregnancy. Where would you go if you have any question?

P: I would ask my friend as you said ‘friends whom you can be open to’. And I can also ask my grandmother, but we don’t have an organization where we can go and ask such questions maybe it’s only OC.

I: What about the aunties and uncles?

P: [pause]

I: No?

P: Yes

I: Did I ask you about your mother? Have you ever talked about HIV/AIDS with her?

P: Yes

I: Who started the discussion?

P: Sometimes it’s her who started

I: What triggered her to start talking about HIV/AIDS?

P: Because she wanted to protect me as her child so that I am not taken up by petty thing and men which can destroy my life.

I: So, she advises you?

P: Yes

I: Alright. I would like to know about the relationships that happens between boys and girls in this community. What are your views on such relationships? Girls and boys having relationships?

P: As for me; I don’t take part in them totally because my parents advises me and I take heed of the advice, because I wasn’t taking heed then I would have been pregnant or contracted diseases. Because the friends that I had, I was playing with them and we are of the same age but as for now some of them has one child each some are pregnant.

I: How often is it that girls of your age get married?

P: Sometimes hardship that one meets at home makes them to do that and sometimes being unable to take heed of the advice given or being taken up by what men do.

I: How often does this happen that girls of your age be found pregnant or married?

P: [pause]

I: Is it rampant or rare?

P: It is more, if you go round the village and ask, most of the girls are married and some has children of whom sometimes the men refused the responsibility, and they are having problems with the babies. And this problem is becoming very common in girls.

I: And when they are married, what are the problems that they meet?

P: When they are married, they meet different kinds of problems. For example, if at her parents house she was able to find help without struggles, when married, they go to fetch firewood with children on the back when they were supposed to have good marriage and well taken care of, and such kind of life here is very rare and there is no one who has attained such life here. To be married and go and leave somewhere and say that they have gained something, no, there none. So, some of the examples we take them from such kind of things. We know that if I do that I will be one of them, very poor.

I: Okay, so what happened for you to join Dream; to join OC?

P: At that time I dropped out of school and visitors came at N school from OC. And the facilitator went there and were told to list down names of those who dropped out of school from 10 – 14 years. And I was listed because at that time I dropped out of school, and when I learnt about OC and that VB who gave birth to a baby returned to school and I saw it wise to go back to school. And now am in school.

I: How has the program helped you?

P: I have benefitted because I have learned how to avoid AIDS, and I have known how a girls body change and to me it’s now not a new thing. And I have learnt how to avoid unplanned pregnancies and a lot of more.

I: We heard that in OC clubs there are other things that happens, what are the activities that you also learn there as girls?

P: Programmes?

I: Mmm

P: At OC we do discuss a lot, we discuss things from a booklet; about you and AIDS, health and your body. We do discuss a lot.

I: Alright. Are girls able to find assistance there, maybe a girl want contraceptives or to avoid pregnancy, is it possible at your club to find assistance?

P: It is possible since one time they came with car and pitched a tent at school and those who wanted were going to get contraceptives and those who wanted were getting condoms to avoid some of the things.

I: Mmmh. Alright, and if your friend want to join OC, would you advise her to join this club, why?

P: It is acceptable to join since maybe there might be some of things that she is lacking on her life and she has heard that they are taught there, so maybe she want to get such kind of assistance. We cannot deny her but encourage her to be part of the group so that we learn together.

I: Okay, in assumption, if a girl is found pregnant, or you are pregnant, or you have a boyfriend, whom would you ask for advice about boy and girl relationship? Let us say you have a boyfriend and is insisting to have sex with you, or you are pregnant or you have been found with STIs. As a young girl, where would you find such advice?

P: I would ask our facilitator, asking what I can do so that I get out of those problems or if I get that disease, I would also tell a friend to encourage me not to worry much. And my parents as I said that it is good to be open to your parents so that they can help you from problems.

I: You’ve already said that more girls here are found to be married or pregnant. What happens, is it that you don’t have a place where they can go to find assistance or what is it that happens?

P: [pause]

I: Is it that they do not have adequate assistance or what?

P: For those ones, sometimes they agree with their boyfriends to use condoms and some men are not frank and they drill a hole on the condom in front and if the girl was thinking of protecting herself from pregnancy and STIs then everything has gone wrong she will get pregnant when she was thinking that she has protected herself with a condom.

I: Okay, going back to the same program of OC, do you have a desire to stop taking part in the group.

P: No, I cannot stop.

I: Why?

P: Because there are a lot of things that are taught that would help one’s life

I: Alright. Can you tell your friends to take part in Dreams program?

P: Most of our friend do not like to join this group, we don’t know why really. Those who like the program are few.

I: So what would you advise them?

P: Some of them says the program encourages sex , and even when you tell them that there is a lot that you would avoid, they do not take it. They believe that this encourages a lot of girls to have sex or having unprotected sex.

I: Okay, thank you very much for answering these questions, those were my questions. Do you have a question?

P: [pause]

END
