# Supplementary material for: Social support and very young adolescent girl’s knowledge on sexual relationships: A comparative qualitative study of Girl Only Clubs’ participants and non-participants in rural Malawi
Source: PLOS Glob Public Health. 2023 Jan 12;3(1):e0001339. doi: 10.1371/journal.pgph.0001339 (PMC10022037; doi:10.1371/journal.pgph.0001339)
Supplement: S1 Data — (ZIP) [file pgph.0001339.s003.zip › Anonymized/IDI7_R1_YY7_May.docx]

District: YY

Location: A

IDI ID: IDI1_R1_YY7_May2019

Interviewer: WWW

Data collection date: DD/MM/2019

I: As explained earlier, I would like to have a chat with you and know more issues about your life as a girl who has grown up in this village. But also know what you have met from the time you joined the Dreams program of GG Club. Are you in GG Club?

P: Yes I am

I: You are in GG Club?

P: Alright. And as said earlier, be free, don’t be shy and speak louder so that we can record, right?

P: Yes

I: Alright, first things I wanted to know; when you are staying here as your home; this is your home, right?

P: Yes

I: This is your parents house, right?

P: Yes.

I: On a day like this, when the day comes out, what do you do?

P: I do household work like sweeping, fetching water and washing dishes.

I: That’s in the morning, right?

P: Yes

I: So, after washing plates and doing other chores that you do, what else do you do?

P: I play with my friends.

I: Okay, how many friends do you have?

P: Only 4

I: Where are your friends residing?

P: There (pointing to the friend’s houses), there too, and behind the *Bwemba* (Tamarind tree) and there too.

I: So, when you are with these girls, what do you do when you are playing? What are the games that you do?

P: We teach each other what we learn from GGs Club that ‘when I finish my education’- for example, I tell my friends that when I finish my education I would love to work with OC. And my other friends said that if she finishes her education she would like to be a teacher.

I: Okay, and what are other games that you do?

P: [pause]

I: Are they the only games that you play?

P: I am open with my friends.

I: When you say that you are open, what do you do?

P: For example, when I am working she might come to help me, and when we finish working here we also go to her place and I help her with chores.

I: Alright, when we think of this place where we are coming from; where do girls like you love to be to chat when they are free? Which place do you like to go for chatting?

P: We….

I: As girls where do go for a chat?

P: With my friends, we use that house.

I: Mmmh, apart from the houses you’ve mentioned, are there any other places where you go, as girls? Do you only go to that house and that house, or there is another place where you also go?

P: Yes there is a place where we go

I: Which place?

P: At school and also at Girls Club meeting

I: When you are back from school and GGs Club, is there any other place where you meet?

P: Yes

I: Where?

P: We play on the ground.

I: Alright, apart from the ground?

P: At my grandparents

I: You also go to your grandparents?

P: Mmm

I: Do you go to the fields?

P: Mmm, we go

I: Do you go there as girls?

P: Yes

I: What do you do there, at the fields?

P: To work on the fields

I: Alright, in the places where you’ve mentioned, when you go there as girls, can we say they do have enough security or we can say sometimes you meet obstacles or dangerous things?

P: Yes

I: You meet obstacles?

P: Yes

I: What kind of obstacles?

P: Some start cursing friends, and sometimes when you go there, your friends mother and other people there will start saying that ‘why do you chat with that person, do you know her’.

I: Alright, and how do you chat with boys?

P: I don’t chat with boys

I: You don’t chat with boys?

P: Mmm

I: Alright, or when you are playing and the boys coming in to disturb you, does that happen?

P: I get away

I: But that happens that when you are playing boys comes to disturb you?

P: Yes

I: Okay, alright. Now we would like to understand about relationships that you have with other people. You as T, who is the person you like most to chat with? The person might not be a friend, but who is the person whom you are able to open up to and tell her your issues, who is that person?

P: RM, from that house

I: Okay, you and RM, what do you like doing?

P: We like doing some games like what we learn from GGs Club

I: What kind of games?

P: Playing netball and doing other games that we learn from GGs Club.

I: Alright, you and RM, what are the problems that you meet?

P: Problems like when I go wrong in doing something, I might be beaten and not get food.

I: On what kind of things?

P: For example, one day they told me to sweep the compound and that day I said that I am tired someone should do it today, so they didn’t give me food and beat me.

I: Alright, and you’ve said that your friend whom you are open to is RM?

P: Yes

I: Alright, why did you choose RM to be the person that you will be open to? Why are you open to RM and not any other person?

P: I chat well with her

I: Alright, I also wanted to know, if you have a problem, or you have questions; sometimes one may have question, sometimes there might be sensitive issues or those that you cannot say in public, or shameful that you can say them on public, on such issues, who do you ask?

P: I would ask my teacher who teaches me GGs Club

I: Why would you ask this teacher?

P: [pause]

I: Why did you choose this teacher and not any other person?

P: Because the teacher teaches us GGs Club and other problems and when we meet problems we tell them and they meet our parents.

I: Mmm, for example, which problems, you said that when you meet problems? I would like to know what kind of problems that you can tell your teacher and then your teacher should tell your parents?

P: As for me, the problems that I meet is that my father beats me and one day they made me sleep outside, so I tell them.

I: Why does he beats you or make you sleep outside?

P: When I do an errand in a wrong way

I: Have you ever slept outside?

P: I wanted to one day but didn’t happen, he felt pity for me and made me enter the house.

I: Alright, I would like to know, what kind of issues do you discuss at GGs Club?

P: We learn about visions; on that topic they told us that girls should have a vision, be jacked up and independent, and depend on anyone else.

I: Alright. And what did they say on how to fulfil such visions?

P: We should work hard on our education

I: Alright, what else do they teach at GGs Club?

P: About my dreams; and told us that we have a choice of the job to do when we finish school.

I: Alright, and at GG Club, have you ever learnt about issue concerning HIV/AIDS?

P: Yes

I: What did they say about HIV?

P: They say that [pause]

I: What do they say about HIV?

P: Having sex using condoms and using contraceptives

I: They told you about all these?

P: Mmm

I: Okay, so when they are saying using condoms, what do they say that is the advantages of that?

P: That we should not get pregnant

I: You should get pregnant, right?

P: Mmm

I: Alright, I just wanted to know; sometimes it happens that a young girl like you gets pregnant, right?

P: Mmm

I: Does it happen?

P: Mmm

I: So, let us assume that it happens to you, who can you tell this issue?

P: My parents but also the one who teaches us GGs Club, maybe my grandmother

I: Okay, on those three, you’ve mention your parents, the one who teaches you GGs Club and your grandmother, who can be the first one?

P: My mother

I: Your mother?

P: Mmm

I: Why would you tell your mother? Wouldn’t you fear that she will beat you? Why would you tell her first?

P: [pause]

I: Be free, why would you choose your mother not any other person?

P: She should know earlier

I: Alright, why would you choose your grandmother?

P: I would choose my grandmother with the reason that she should know

I: She should just know?

P: Mmm

I: Just to know?

P: [pause]

I: Why didn’t you choose aunt or uncle but you choose grandmother?

P: [pause]

I: Why didn’t you choose RM, you said you are very open to her? Would you tell RM?

P: Yes I can tell her

I: Mmm, alright. And why would you choose a GG Club teacher?

P: [pause]

I: Alright, we are just assuming, right?

P: Mmm

I: If you may find that you have STIs, who can you tell this to?

P: I would tell the one who teaches us GGs Club, so that if they know they might come and tell my parents whom I haven’t told.

I: Okay, *umasamba* ‘do you have monthly periods’?

P: Mmm

I: Not the kind of ‘body bathe’ but monthly periods?

P: No

I: Okay, when one is growing up, sometimes they start having more questions. Sometimes their breast starts growing and boys start asking them for a relationship. On such kind of issues, you as T, when you have questions, who would you love to ask?

P: My friend who chats with me like RM or the one who teaches me at Girls club or my parents.

I: Have you ever discussed these issues with RM?

P: Yes

I: What do you discuss when you are chatting on such issues?

P: For example, for her, she has never been approached by a boy and she told me. I asked her which boy was asking for relationship and she told me the name of the boy. But now the boy went to his home.

I: He was asking for a relationship?

P: Mmm

I: And RM accepted?

P: She refused

I: Alright, and for you; have you ever told her anything?

P: Yes

I: What did you tell her?

P: There was another boy who wanted a relationship with me and I refused and went to tell RM.

I: Mmm. Did you tell your mother?

P: No

I: Why didn’t you tell her?

P: [pause]

I: Why didn’t you tell your mother?

P: [pause]

I: Why didn’t you tell GG Club teacher?

P: [pause]

I: Okay, I also wanted to ask you another question; in your village, apart from GG Club, where else, would girls of your age get counselling about HIV/AIDS or STIs or issues of getting pregnant or contraceptives, where else would girls get this counselling?

P: There was a group of which I was a member where such issues were being discussed but this group no longer exist.

I: Where else?

P: That’s the only group I knew

I: Are there people like Counsellors who are able to tell young people of your age about such issues?

P: Mmm

I: Who is that person?

P: C

I: Who is C? I wanted to know about this person.

P: [pause]

I: C is the one who tells girls about such kind of issues?

P: Yes

I: Where does C live?

P: Behind the house, there.

I: That’s the name C?

P: Yes and I don’t know the other name

I: Is C a woman?

P: No is a man

I: Does he work as C or C is his name?

P: It is his name but he job is to go to girls who has gotten pregnant and ask her

I: Okay, apart from C where else do girls go to find help on issues like; if a girl is found pregnant or has STIs or she want counselling or has questions on HIV? Where else would a girl go?

P: [pause] Even at school, where she goes. She can go there and explain.

I: Okay, you said that you go to school, was there any other time when you were not going to school?

P: Yes

I: Why were you not going to school?

P: I lacked uniform and K500. They told us to pay K500 and my parents were not able to achieve that.

I: And you stopped going to school?

P: Mmm

I: And why did you go back to school?

P: Because I joined GGs group and I learnt about vision and they told us things that would make us fail to attain our vision. And they told us about choices of the jobs that we would like to do when we finis our education, that is why I came back to school.

I: At the time you were not going to school, what were the problems that you were meeting, what were you doing here at home? Were you just staying, what were you doing?

P: At the time when I was meeting obstacles there; at that time I would go to school hungry without eating and when I knocked off from school I couldn’t find anything to eat.

I: So what were you doing when your friends were in school, what were you doing here at home?

P: I was just staying

I: Just staying?

P: Mmm

I: And what was your mother saying at the time you were not going to school?

P: On that day, she told me to go to school and if they will chase me they will follow up on it. And I went there, they chased me and my parents didn’t go to follow up.

I: Alright, when you think about your mother and father; what do you think is your father’s responsibility on you? What does your father do on you?

P: [pause]

I: You said you have a father, right?

P: Mmm

I: What does your father do to help you? Or does your father has any importance to you or on your life?

P: Mmm

I: What is the importance of your father in your life?

P: He buys maize flour for us to eat and also finds money to buy us notebooks.

I: And what is the importance of your mother on your life?

P: [pause]

I: What is the importance of your mother in your life?

P: [pause] She cooks for us and when I was young she was washing my clothes

I: What are the advices that your father gives you?

P: He tells me not to refuse working

I: And your mother what advices does she offer?

P: I shouldn’t refuse to cook

I: Mmmh, what else does your parents advise you to do?

P: I should fetch water and sweep the surroundings.

I: And on those issues we discussed, on HIV, boys relationships, issues of contraceptives, have you ever talked about these issues with your father or your father ever said about such issues?

P: No

I: Has never talked about it?

P: Mmm

I: What about your mother?

P: She has never talked about it

I: Do you have an aunt?

P: She is far from here

I: What about an uncle?

P: Is also far?

I: Alright, any other elder relative whom you have, have they talked about issues like HIV or sex or STIs or contraceptives, have you ever discussed this with any relatives?

P: We’ve never discussed that

I: Where do you get such information?

P: From GGs club

I: Only GGs Club?

P: Mmm

I: Have you ever gone for initiation?

P: No

I: You’ve never gone for initiation?

P: Mmm

I: Alright, what about your friends in this village, have you ever discussed such issues like contraceptives, HIV, when you are playing, do you discuss about such issues?

P: No, we have never discussed that

I: Have you ever met any problems concerning such kind of issues, like HIV?

P: I have never met that

I: Alright, we are going to an end, right?

P: Mmm

I: Now, I would like to hear your views on relationships between girls and boys. What are your views on such relationships?

P: For example, if a boy asks me out, I would say no, and tell him that I would like to continue with education.

I: Alright, and on issues concerning relationships like these, for example between a boy and a girl having relationship; on your own, do you have questions that you would like to know more about such issues things? Do you have questions?

P: [pause]

I: Are there things that you would like to know?

P: [pause]

I: Maybe on your own?

P: No

I: There’s nothing you would like to know? Or discuss them with another person? Do not be shy?

P: [pause]

I: Are there things you would like to know, not you doing them, but you are interested to know?

P: [pause] mmm

I: Tell me, what would you like to know?

P: I would like to know if a man and a woman are in a relationship, what do they do?

I: Alright, who would you love to discuss with about this question?

P: [pause]

I: You would love to know what happens in relationships, right?

P: Mmm

I: Who would you love to discuss this with you? Have you discussed this with RM?

P: These issues?

I: Mmm

P: No, we’ve never discussed them

I: Alright, but whom would you love to discuss with, such issues? Whom would you love to guide you on such issues?

P: Our teacher who teaches us at GGs club

I: What else would you love to know about relationships?

P: That’s all

I: Let us think of the girls in this village. What’s the name of this village?

P: MP

I: Are there girls of your age in this village who are married, they left school they are married?

P: No

I: But what are your views that they should be married at that tender age?

P: [pause]

I: Is it good to for young girls to marry while young?

P: No

I: Why?

P: You might be found pregnant, and thereafter people from organizations might force you to mention the one who impregnated you and you might mention him and later he might also get you arrested.

I: Alright, I would like to know more about GG club, how did you know about GG Club?

P: [pause]

I: Who told you that there is a group of GG club and how did you join it?

P: My parents told me

I: That you should go and join it?

P: Mmm

I: Okay, why did they tell you to join this group?

P: They said that I should learn new ideas

I: How long have you been in this group? When did you join?

P: 2016

I: Alright, can you remind me of what you learn at Girl-go club.

P: Should I tell others?

I: Tell me about what you learn from GG club and what you have gained?

P: The other gain is that we wanted to hear more about our bodies and Banja la Mtsogolo came at MK to conduct some test and we knew about our status.

I: Was it an HIV test?

P: Mmm

I: What else, what else happens at GGs club?

P: They teach us how to read

I: Alright, what else?

P: They teach us to know how to write

I: So, how has your life changed since you joined GG club?

P: Before I joined GG club, I didn’t know how to read and write but now am learning little by little.

I: Alright, what obstacles have you met there that you would like them to change at GG club?

P: [pause]

I: Things that you think you do not like at GG club?

P: [pause]

I: Nothing?

P: Mmm

I: There is nothing that you would like to change at GGs club?

P: [pause]

I: What about your teacher?

P: I should say that our teacher teaches us in a with one heart because they are not paid, they just teach us.

I: Alright. Thank you for answering all my questions. Do you have a question for me?

P: [pause]

I: Alright thank you for answering the questions, you answered very well. Thank you.

END
