# Supplementary material for: Social support and very young adolescent girl’s knowledge on sexual relationships: A comparative qualitative study of Girl Only Clubs’ participants and non-participants in rural Malawi
Source: PLOS Glob Public Health. 2023 Jan 12;3(1):e0001339. doi: 10.1371/journal.pgph.0001339 (PMC10022037; doi:10.1371/journal.pgph.0001339)
Supplement: S1 Data — (ZIP) [file pgph.0001339.s003.zip › Anonymized/IDI7_R2_YY8_Apr.docx]

District: YY

IDI ID: IDI6_R1_YY8_May2019

Location: A

Interviewer: WWW

Data collection date: DD/MM/2019

**Interviewer:** The first thing I want you to tell me where you learn?

**Participant:** At NNN Primary School.

**Interviewer:** Which class are you in?

**Participant:** I am I standard 5.

**Interviewer:** Alright, is there a time when maybe you dropped out of school because of other challenges?

**Participant:** No.

**Interviewer:** All the times you go to school?

**Participant:** Yes.

**Interviewer:** Okay. What do you want to become?

**P:** I want to be a nurse.

**I:** Uhmm…you want to become a nurse?

**P:** Yes.

**I:** Why do you want to become a nurse?

**P:** I want to be an independent person who does not rely on other people, but I also want to help people that are lacking care.

**I:** Uhmm, so what are you doing so that in order to achieve your dreams of becoming a nurse?

**P:** I am working hard at my education.

**I:** Uhmm, do you do well at school?

**P:** Yes.

**I:** What position were you in your class when you wrote exams?

**P:** I achieved position number 9.

**I:** Uhmm. So you should work hard you should achieve position number 1.

**P:** Okay.

**I:** Is that so?

**P:** Yes.

**I:** So here who do you live with?

**P:** I live with my mother…

**I:** And who else?

**P:** No one else.

**I:** Where is your father?

**P:** My father live at YSS.

**I:** So sometimes you live here and sometimes at YSS?

**P:** No, I have never been to YSS before.

**I:** You have never been at YSS? Where is YSS?

**P:** In that direction. *(Uku.)*

**I:** Okay, so who else do you live with; do you have your relatives?

**P:** Yes.

**I:** How many relatives do you have?

**P:** At my place I have my sister, then I have my brother who is in Form 2 and my small sister who is in standard 2.

**I:** In your household or at home who are you comfortable with to talk about issues with? Between you and your relatives; your brother, your sisters, your mother, who are you most comfortable with to share issues with?

**P:** My sister and my mother.

**I:** Uhmm, these people have you ever talked to them about issues related to HIV?

**P:** Yes.

**I:** Have they ever explained to you about it?

**P:** Yes.

**I:** Can you tell me about the day that they explained to you about these issues, what happened?

**P:** They told me that if I am going to church and someone tries to stop me I should be telling them… that I should be refusing.

**I:** Uhmm.

**P:** And if they are following me I should be saying that I do not want. Also if they say that I should engage in sexual intercourse with them I should be refusing.

**I:** Uhmm…

**P:** If they are telling me that let us go somewhere I should be saying to them that I do not want.

**I:** What caused them to be telling you these things?

**P:** I told them that at YY GG club we have learnt about HIV/AIDS.

**I:** Uhmm…

**P:** Yes.

**I:** So that is when they started explaining to you about these issues?

**P:** Yes.

**I:** Okay, can you tell me about where you heard about HIV/AIDS issues, where did you hear about these issues for the first time? Before you had heard of these issues from anywhere, the first day you heard about these issues about HIV/AIDS…?

**P:** I started hearing about these issues at school, they taught us about HIV/AIDS.

**I:** Uhmm, in the classroom?

**P:** Yes.

**I:** What did they say HIV/AIDS is?

**P:** HIV/AIDS is a virus that spreads diseases.

**I:** Alright, where else have you heard about HIV issues?

**P:** Right here at XX GG club…

**I:** Where else?

**P:** At school we also have a club, it is called mother-group; they invite us in the class together and it is girls only and that is where they tell us about these issues.

**I:** Okay, what about issues to do with getting pregnant. Where did you hear these issues for the first time from?

**P:** I heard about these issues for the first time at school and the also here.

**I:** What did they say about how you can getting pregnant?

**P:** They said that a person is supposed to avoid getting diseases…you should avoid getting pregnant.

**I:** Uhmm…

**P:** A person should not often be having sexual relationships because you can get pregnant while you did not plan for it.

**I:** So what did they say about the dangers of getting pregnant?

**P:** If you do not avoid getting pregnant it might result in you failing to attain your education goals and in so doing it means you have failed to achieve your future dreams.

**I:** Uhmm…

**P:** As a result you will face many challenges in the future.

**I:** Uhmm…

**P:** Yes…

**I:** So in order not to get pregnant, what advice did they give you?

**P:** They told us that we should not engage in sexual relationships or we should not engage in sexual intercourse with males.

**I:** Uhmm…

**P:** Yes…

**I:** Alright, I want to take you back, before you started going to the GG club and now when you are going to the GG club, how do you differentiate the advice that you received at school and any other place to the advice that you received at GG club now…

**P:** I differentiate that maybe when I have gone to chat with my friends, my friends tell me that I should go and engage in a sexual relation…I should go and engage in a relationship but when I come here they discourage me from doing so; what I do is that I follow the advice that I am provided with here. When I hear about HIV I am afraid of the HIV virus because I heard that HIV is dangerous and it can kill people so I am now afraid of this.

**I:** Okay, fine. What about…I did not ask you about when you are not doing any household chores. When you are not doing any work where do you like to go? Where do you go to chat?

**P:** When I am not doing any work at home…?

**I:** Yes…

**P:** I do not go to chat but I take my books and read.

**I:** You take your books and read?

**P:** Yes.

**I:** Do you not have any friends that you chat with?

**P:** I do have friends and if my friends’ want that we should be chatting they are the ones who come to my home so I bring out my books so that if I have homework they can be helping me.

**I:** Uhmm…

**P:** Yes.

**I:** Have you started having your menstrual periods?

**P:** Having my menstrual periods, no…

**I:** You have not started?

**P:** No.

**I:** But have you ever talked about issues to do with menstrual periods with any one before?

**P:** No I have not.

**I:** You have never talked to anyone about such issues?

**P:** No.

**I:** Do you not have questions or anything that you would like to know in relation to menstrual periods?

**P:** Yes. I do have some things I would like to know.

**I:** You have issues that you would like to know about?

**P:** Yes.

**I:** What is it you would like to know?

**P:** I would like to know that when people have menstrual periods how does it happen.

**I:** What else would you like to know?

**P:** I would like to know about the HIV virus that if you are not engaging in sexual intercourse and you have gone to get tested for HIV and they find you HIV positive where does the virus come from?

**I:** What else would you like to know?

**P:** I would also want to know the reason why my father and mother are separated?

**I:** Okay, so your father is at Nankhunda because they are separated?

**P:** Yes.

**I:** Okay so with such issues who can you open up to?

**P:** My mother…

**I:** You would like to open up to your mother?

**P:** Yes.

**I:** Have you ever opened up to your mother before?

**P:** I only opened up to her on the day when I was asking her about the HIV virus?

**I:** You are the one who asked her?

**P:** Yes.

**I:** So did she open up to you and explained properly to you?

**P:** Yes.

**I:** So what did she explain to you?

**P:** I have already explained that she talked to me about HIV issues, that if a person is stopping me I should refuse.

**I:** Uhmm, okay fine. So you have said that you would like to open up and discuss these issues with your mother?

**P:** Yes.

**I:** So what stops you from opening up to her and discussing these issues with her?

**P:** I am afraid that she can tell me that when it comes to menstruating, I am not old enough to know about such issues.

**I:** Uhmm…

**P:** Yes…

**I:** Okay, what about on the issues of relationships between boys and girls, are such relationships very common in this village?

**P:** It is very common indeed.

**I:** It is very common?

**I:** What do you think makes a lot of girls to engage in relationships with boys?

**P:** Some of them do not come to XX GG.

**I:** Uhmm…

**P:** Yes. So when we are telling them they tell us that these are just lies and they cannot do that.

**I:** Okay. So when they are engaging in relationships what is it that they say they want?

**P:** I do not know...

**I:** Uhmm, fine. What other challenges do the girls in this community face? When they are walking about the girls in the community, what other many challenges do the girls encounter?

**P:** Being raped. Having their breasted touched…yes…

**I:** Uhmm. Do such things occur very often?

**P:** Yes.

**I:** Okay, when thing like this happen where do the girls go to report such issues?

**P:** We report such issues to the mother-group as well as our teacher who is also a chief. Yes.

**I:** So what happens to the person that has been raped?

**P:** They assist her by taking her to the hospital.

**I:** Uhmm.

**P:** Yes.

**I:** Has something like this ever happened to you?

**P:** No.

**I:** But you have heard that it has happened to someone before?

**P:** Yes.

**I:** What happens to the person who does this, be it a man or a boy?

**P:** They might be taken to the police or maybe being called at the chief’s court so that they can be questioned about it or what made them to do it.

**I:** Uhmm, okay, Fine. So you have told me that you have not yet started your menstrual periods; but maybe you have started noticing some changes in your body, maybe the breasts have started getting big, or maybe hairs have started to grow; so I want you to tell me that when you started noticing these changes on your body how did you feel about it? Were you afraid or did you have any questions? What did you do when you noticed that maybe pimples have started to come out?

**P:** Yes I was afraid.

**I:** You were afraid?

**P:** Yes.

**I:** So what did you do?

**P:** I asked my parents.

**I:** Your mother?

**P:** Yes, and she told me that these are signs that you are growing up.

**I:** Uhmm.

**P:** Yes.

**I:** Why did you ask your mother and not any of your friends?

**P:** I did not ask any of my friends because some of my friends can be hiding information from me that is why I asked my mother so that she could explain it properly to me.

**I:** Uhmm.

**P.** Yes.

**I:** So you have explained to me that you are a member of XX GG club?

**P:** Yes…

**I:** What happened for you to join the GG club?

**P:** I heard from school about the issues of HIV/AIDS and when I came here and when we were taught on the first day I heard about issues to do with knowing each other *(Zo ziwanaza)* so I got interested that I should be coming here in order to know more about issues that are in the My DREAMS book *(Bukhu la Maloto Anga.)* Yes.

**I:** Who told you about this group, who told you about the XX GG club group?

**P:** I was told about the XX GG Club by my teacher who is also a chief. He told us that we should start coming here for club activities. So I came here once that is when I heard about knowing each other *(zo ziwanazo.)* Yes.

**I:** What is it they say about knowing each other, or in what way should you be knowing each other?

**P:** They said that we should know each other; for instance me and my friend we should know each other so my friends needs to introduce herself, then the rest of us will do the same thing by mentioning our names too.

**I:** What do they say is the advantage of knowing each other?

**P:** They said that the advantage of knowing each other is that if you friend is involved in an accident then you can be able to assist her that is if you know her. Yes.

**I:** Okay. Fine, so that is when you joined the GG club?

**P:** Yes.

**I:** Okay. Apart from knowing each other, what else…apart from that they also teach you about HIV/AIDS what else do you benefit from this GG club group?

**P:** We also benefit from being educated.

**I:** What kind of education?

**P:** The benefits of going to school. If a person is enrolled in school you need to work hard at school so that you can help yourself as well as that you should be able to tell your friends that I will be like that person in the future.

**I:** What else?

**P:** They also said that if a person is at school you are supposed to have a dream for the future.

**I:** Okay. So your dream is to become a nurse? Anything else?

**P:** We learnt about the different food groups, yes.

**I:** Okay. Fine. What about other services, like services to do with health issues, have they ever assisted you with such services maybe referring you to the hospital to get tested for HIV, to access Family Planning services or I do not know, what kind of health services do they provide to you?

**P:** They assisted us with accessing HIV testing services, yes.

**I:** They assisted you to get tested?

**P:** Yes.

**I:** What happened for them to get you tested, did they actually come here to provide you with HTC services or did you actually go somewhere to get tested?

**P:** They came here to provide us with the HIV testing services.

**I:** Everyone in the club or only those that were willing to get tested.

**P:** No they got everyone tested.

**I:** Uhmm. Okay. So when you are participating in the activities of your GG club, how free do you feel?

**P:** I feel very free because there are no males so that I cannot just remain quite. So I am able to feel free because it is only us girls that are present there. I am also able to learn about the benefits of school. Yes, a lot of things.

**I:** Do you have intentions to quit being a member of the Go Girl club?

**P:** No. I do not want to quit.

**I:** Why not?

**P:** Because I have leant about HIV issues and I also want to complete the book; I want to know more about the issues that are included in the book.

**I:** Uhmm. What if your friend wanted to join the GG club, a friend that has never joined the group before, how can you encourage such a friend?

**P:** I can tell her that let us go so that you should learn, we have learnt about HIV issues so you too you should know about your DREAMS, the benefits of going to school, eating food from all the different food groups, getting tested for HIV. You should also go so that you can know about all these issues. Yes.

**I:** Uhmm. Thank you very much, we have reached the end of our questions. Do you have any questions?

**P:** No I do not have any question.

**I:** Thank you very much for your good responses. You have really responded well to the questions.

**P:** Alright.

**I:** Thank you

***End of interview.***
