# Supplementary material for: Social support and very young adolescent girl’s knowledge on sexual relationships: A comparative qualitative study of Girl Only Clubs’ participants and non-participants in rural Malawi
Source: PLOS Glob Public Health. 2023 Jan 12;3(1):e0001339. doi: 10.1371/journal.pgph.0001339 (PMC10022037; doi:10.1371/journal.pgph.0001339)
Supplement: S1 Data — (ZIP) [file pgph.0001339.s003.zip › Anonymized/IDI8_R2_YY10_Apr.docx]

District: YY

Location: A

IDI ID: IDI1_R1_YY10_May2019

Interviewer: WWW

Data collection date: DD/MM/2019

**Interviewer:** Once again I am WWW from COM. We are conducting a research study in collaboration with PC of the USA. We are interviewing various girls concerning GG Clubs. We want to know what you learn at GG Club and how you learn about HIV as well as about pregnancies and relationships between boys and girls. How do you get to know about this? Is it from the community, mother groups, is it from your friends or parents, from your sisters and brothers, how do girls know about all this? This information will help give guidance in the designing of health programs for young adolescent girls ages 10 to 14 years. As I have explained I want you to be open. Tell me what you know , It’s not an exam, just say what you know, any answer that you give is right. You understand?

**Respondent:** Yes

**Interviewer:** Firstly I want to know .do you go to school?

**R:** Yes

**I:** Which class are you?

**R:** Standard 6

**I:** What do you think about your future?

**R:** I want to do school so that I become a nurse

**I:** Why do you want to be a nurse?

**R:** I admire when they are injecting people in the hospital.

**I:** Ok, what are you doing for you to become a nurse?

**R:** I work hard in school

**I:** Last year which position did you scoop? Last term which position were you?

**R:** 7

**I:** You are really working hard, congratulations, ok, when you are done with your house hold cores, you have swept, cooked, drawn water and you have bathed, what do you like to do?

**R:** I read my books

**I:** You read your books?

**R:** Yes

**I:** What else do you do at your free time when you are not reading your books?

**R:** I go to play netball

**I:** Where to you play netball?

**R:** That side ….

**I:** Ok, where else do you to chat as a girl?

**R:** I go to my friends

**I:** To chat with them?

**R:** Yes

**I:** I would like to know, what are the dangers that girls encounter in the places that you to chat?

**R:** They can be raped, their breast can be touched

**I:** Ok, what else?

**R:** Being forced to get married

**I:** Oho, who is forced to get married?

**R:** Women

**I:** Have you ever been forced to get married?

**R:** No

**I:** It has never happened to you?

**R:** No

**I** Those that have been raped or their breast touched where do they report?

**R:** They report to their parents then to our teacher at OC

**I:** Do you know person that this happened to her?

**R:** No

**I:** You just hear about it?

**R:** Yes

**I:** Who does that to girls?

**R:** Boys and Men

**I:** Boys and Men, when you are going out with your friend’s maybe to watch sports are you not afraid?

**R:** We are afraid

**I:** But you still go?

**R:** Yes

**I:** How do you protect yourselves?

**R:** We go in a group

**I:** When you are with you fellow girls what stories do you like to talk about?

**R:** We talk about school

**I:** Ok, do you talk about HIV as girls?

**R:** Yes

**I:** What do you say?

**R:** One has to avoid sexual intercourse so that she should not contract HIV

**I:** What else?

**R:** Use a condom to avoid contracting HIV

**I:** Where did you first hear about HIV?

**R:** At school

**I:** In class?

**R:** Yes

**I:** Your teacher was teaching?

**R:** Yes

**I:** You learnt that what is HIV?

**R:** That HIV is a virus that causes AIDS

**I:** What else did they teach you at school?

**R:** I have forgotten others

**I:** Ok, where else did you hear about HIV?

**R:** OC

**I:** What did they teach you at OC?

**R:** They taught us the same things

**I:** What you learnt in school?

**R:** Yes

**I:** How significance it is, you learnt at school, OC and mother group, do the mother group teach you at OC?

**R:** No, the mother group are different

**I:** Ok, it’s different with OC?

**R:** Yes

**I:** What do you learn with the Mother group?

**R:** I don’t go to mother group

**I:** You don’t go but you go to OC?

**R:** Yes

**I:** If you differentiate what you learnt at OC and at school. You learn about HIV and at school and the same thing at OC so what is the benefit of being at OC?

**R:** The benefit?

**I:** Yes

**R:** We know more about HIV

**I:** But you also learn from school?

**R:** Yes

**I:** How different are they?

**R:** It’s not different

**I:** It’s not different? Then there isn’t any benefit?

**R:** There is a benefit

**I;** what else did you learn at OC?

**R:** They taught us that girls and boys are the same. Whatever a boy can do, a girl also can do.

**I:** Mmmh, what else?

**R:** Knowing each other

**I:** Mmh, so they teach you that there no is no difference between boys and girls?

**R:** Yes

**I:** Does this work when you get home?

**R:** When we get home we tell our parents we can share household chores with our brothers, that a boy can clean the dishes while the girl is sweeping the surrounding.

**I:** Your parents understands that? Do they tell the boys to work?

**R:** Yes

**I:** How do you do it for the boys to listen to you?

**R:** We tell our parents

**I:** Do they listen to them?

**R:** Yes

**I:** Have you ever heard about pregnancies?

**R:** Yes

**I:** Where did you first hear about it?

**R:** I heard about it when we were chatting with my friends

**I:** Tell me what you were discussing?

**R:** Problems that come because of the unplanned pregnancies and we should avoid sexual intercourse and early marriages

**I:** You said you have learnt not to have sexual relationships, do you have those relationships in this area?

**R:** They’re very few

**I:** Very few?

**R:** Yes

**I:** What do you think girls engage in sexual relationships?

**R:** So that they get money

**I:** They should get money?

**R:** Yes

**I:** What else? Have you ever been counselled on what to do when you are in a relationship with a boy?

**R:** No

**I:** Or maybe someone just telling you what happens when a boy and a girl are in a relationship

**R:** No, I have never heard of it

**I:** Not even at OC?

**R:** We discussed

**I:** What did they say about relationships between boys and girls?

**R:** Between a boy and a girl?

**I:** Yes

**R:** We should just chat

**I:** You should chat?

**R:** Yes

**I:** Can you explain when you say to chat what do you mean?

**R:** Like when you are at school, maybe they have given you homework, you can ask a boy to help you with homework.

**I:** Ok, just that?

**R:** Yes

**I:** They told you that you can chat between a boy and a girl helping each other homework?

**R:** Yes

**I:** But what happens in the communities? When girls are in a relationship with a boy?

**R:** I have never heard about that

**I:** I want to talk about Menstruation, did you start menstruation?

**R:** Yes

**I:** Were you counselled about menstruation before you started menstruation?

**R:** No

**I:** You didn’t anything?

**R:** Yes

**I:** Tell me what happened to you? Tell me what happened that day you started menstruating ? like a story, tell me?

**R:** I started having abdominal pains, then I just saw blood, I told my sister.

**I:** Then what happened?

**R:** She told my parents

**I:** When this happened like having abdominal pains then blood, how did you feel? Were you afraid or you were happy?

**R:** I was afraid

**I:** How strong were you to tell your parents? You told your mother and fathers?

**R:** My mother

**I:** What did your mother say?

**Silence**

**I:** Why did you tell your mother and not your friends? Why did you chose your mother? Be open

**Silence**

I: Ok, what did your mother say?

**R:** She said when a person start menstruating It means she is a grown up now.

**I:** What else did she tell you?

**R:** That’s what she said

**I:** Ok, she just told you that when you have started menstruating you are now a grown up, all the other things you started doing own your own? She didn’t tell you what to do?

**R:** I have forgotten

**I:** Ok. How did you join GG Club?

**R:** They were moving around writing names, this other day they went to the village chief, they were learning then I just joined them.

**I:** What support do you get from GG Club?

**R:** stammering

**I:** How do you benefit from one what you learn at OC?

**R:** We were given school bad, notebooks and we got tested

**I:** How open are you participating in the GG Club?

**R:** I do respond to the questions

**I** You answer questions?

**R:** Yes

**I:** Ok, how has your life changed since you joined GG Club?

**R:** Before I joined OC, when I knock off from school I was entering into video shows but when I joined we were advised not to enter into video shows, now I stopped entering into video shows.

**I:** What else changed?

**R:** We learnt that there is no difference between boys and girls, girls can do what boys do.

**I:** How did this change? Tell me what happens at your home?

**R:** It changed because I explained to my parents that a boy and a girl can do similar work.

**I:** Since then what happens?

**R:** We do similar work with my brothers

**I:** How old is your brother?

**R:** He is 13years

**I:** Does he cook?

**R:** Yes

**I:** You all cook, and clean the dishes?

**R:** Yes

**I:** Ok, Do you think at some point you will stop going to the Club?

**R:** No

**I:** Why don’t you want to stop?

**R:** I learn a lot from there

**I:** How can you encourage a friend who is interested to join?

**R:** I can encourage her, I can tell her that we learn about how we can prevent HIV and gender equality.

**I:** I have finished the questions I had for you, do you have any question?

**R:** No

**I:** Ok, Thank you very much for answering my questions.

**End of interview**
