# Supplementary material for: Social support and very young adolescent girl’s knowledge on sexual relationships: A comparative qualitative study of Girl Only Clubs’ participants and non-participants in rural Malawi
Source: PLOS Glob Public Health. 2023 Jan 12;3(1):e0001339. doi: 10.1371/journal.pgph.0001339 (PMC10022037; doi:10.1371/journal.pgph.0001339)
Supplement: S1 Data — (ZIP) [file pgph.0001339.s003.zip › Anonymized/IDI9_R2_YY11_Apr.docx]

District: YY

Location: A

IDI ID: IDI1_R1_YY11_May2019

Interviewer: WWW

Data collection date: DD/MM/2019

**Interviewer:** Once again my name is WWW and I work for COM. We are conducting this research in collaboration with PC of the USA. We will are talking with various girls recruited in GG Clubs in Zomba but also in Machinga about what you learn at GG Clubs. We are especially talking to Girls in the age range of 10 to 14 years. In addition I would also like to know how girls acquire knowledge about various issues affecting their Sexual Health. Please be free to answer any question that you feel comfortable to answer. All your responses will be of value to me . First, tell me which school do you go?

**Respondent:** GG Club

**Interviewer:** Do you go to school?

**Respondent:** Yes I go to NMS Primary School

**Interviewer:** You go to NMS? What class are you?

**Respondent:** Standard six

**Interviewer:** Standard Six?

**Respondent:** Yes

**Interviewer:** Was there a time in your life that you did not go to school or you dropped out of school?

**Respondent:** No, I have never dropped out of school

**Interviewer:** You have never dropped out of school?

**Respondent:** No

**Interviewer:** How far do you want to go with your education? What do you to be in future?

**Respondent:** I want to be a Nurse

**Interviewer:** You want to be a Nurse? This area everyone wants to be a nurse, why do you want to be a nurse?

**Respondent:** I want to be like nurses at the clinic

**Interviewer:** Mmmh ,you want to be like nurses at the clinic, what are you doing to fulfill your dream of becoming a nurse?

**Respondent:** I am working hard

**Interviewer:** You are working hard, Whom do you stay with?

**Respondent:** My mum and dad

**Interviewer:** Do you have siblings?

**Respondent:** Yes

**Interviewer:** How many siblings do you have?

**Respondent:** Those that were born in the same family?

**Interviewer:** Yes

**Respondent:** We are six children

**Interviewer:** Ok, out of the six children, you are number what inline?

**Respondent:** Am the last borne in the family.

**Interviewer:** What do your others siblings do?

**Respondent:** Some work at TO

**Interviewer:** Ok, what about the rest?

**Respondent:** We stay together in our house

**Interviewer:** How many girls are in your family?

**Respondent:** 2

**Interviewer:** You and?

**Respondent:** Me, there is X and there is another one.

**Interviewer:** Mmh, is this your home village or you migrated from somewhere?

**Respondent:** They just bought a land here but our home village we take that road

**Interviewer:** Where?

**Respondent:** That road but before JI

**Interviewer:** Mmmh, what do you like doing when you are home? When you wake up in the morning what do you do?

**Respondent:** I sweep, draw water, clean the dishes then I bath going to school

**Interviewer:** After school?

**Respondent:** After school I study, I go again to the borehole to clean the dishes

**Interviewer:** You don’t have time to go to play?

**Respondent:** I do play

**Interviewer:** Where do you play?

**Respondent:** At the BB, where you are coming from.

**Interviewer:** Mmh that is where you like going to play?

**Respondent:** Yes

**Interviewer:** I would like to know the challenges that girls encounter when you go to play

**Respondent:** They can be raped, their breast and bumbum(buttocks) can also be touched

**Interviewer:** Oho, has this ever happened to you?

**Respondent:** No

**Interviewer:** Does happen to other girls here?

**Respondent:** Yes

**Interviewer:** When this happens, what do you do?

**Respondent:** They report to the teachers, to the village chief and some to the Police

**Interviewer:** Who does this to the girls?

**Respondent**: Men and boys

**Interviewer**: How old are these boys?

**Respondent:** Some are older some are young

**Interviewer:** Ok, what do they do the person?

**Respondent:** The person that touches girls breast?

**Interviewer:** Yes

**Respondent:** They advise him not to do that again

**Interviewer:** Ok, Where did you learn about HIV for the first time?

**Respondent:** Here

**Interviewer:** Where?

**Respondent:** Our teacher …

**Interviewer:** The mother group?

**Respondent:** Yes

**Interviewer:** What did you learn?

**Respondent:** About HIV?

**Interviewer:** Yes

**Respondent:** We learnt that HIV is a virus that causes AIDS

**Interviewer:** What else did you learn about HIV and AIDS?

**Respondent:** Avoid sexual intercourse

**Interviewer:** What else?

**Respondent:** We should use condom

**Interviewer:** What about on pregnancy

**Respondent:** Mmmh

**Interviewer:** Did you earn about getting pregnancy?

**Respondent:** Yes

**Interviewer:** What is getting pregnant all about? What happens?

**Respondent:** I have forgotten about that

**Interviewer:** You learnt about that but you have forgotten?

**Respondent:** Yes

**Interviewer:** You also learnt about that here?

**Respondent:** Yes

**Interviewer:** Apart from the mother group where else do you learn?

**Respondent:** The mother group from school also teach us

**Interviewer:** The mother group from school also teach you?

**Respondent:** Yes

**Interviewer:** Ok, what else?

**Silence**

**Interviewer:** What about Nsambo (menstruation?)

**Respondent:** I have never heard about that

**Interviewer:** Have you ever heard that girls do menstruation

**Respondent:** Yes

**Interviewer:** Have you ever experienced menstruation yourself yet?

**Respondent:** Yes

**Interviewer:** Before you started menstruating was there anyone you counselled you about menstruation?

**Respondent:** Yes

**Interviewer:** Who counselled you?

**Respondent:** My mum

**Interviewer:** What did she tell you?

**Respondent:** She advise me to bath

**Interview:** You mean bathing as washing your body?

**Respondent:** Yes

**Interviewer:** With water?

**Respondent:** Yes

**Interviewer:** Have you ever heard about menstruation?

**Respondent:** Yes

**Interviewer:** What is it?

**Respondent:** (Laughs)

**Interviewer:** Don’t be shy, remember I said we should be open and free

**Respondent:** Yes

**Interviewer:** What did they say?

**Respondent:** Growing up

**Interviewer:** Ok, so when I was saying about Nsambo (menstruation) I was talking about menstruation. Did you start menstruating?

**Respondent:** No

**Interviewer:** Have you ever been told about menstruation?

**Respondent:** Yes

**Interviewer:** Who told you?

**Respondent:** My mum

**Interviewer:** What did she say? I want to know what she told you

**Respondent:** She said when you are growing up, breast start growing first …(laughs)

**Interviewer:** Say then what?

**Respondent:** Then on the face

**Interviewer:** Then?

**Respondent:** Breast and on the face

**Interviewer:** Ok, alright, i see you are shy, since you have haven’t reached puberty, what questions do you have? Things you want to know about this? The questions you have and things that surprise about this? Do you have questions?

**Respondent:** No

**Interviewer:** You don’t have questions

**Respondent:** No

**Interviewer:** Are not afraid on when it will happen?

**Respondent:** No

**Interviewer:** If it happens, who will you tell?

**Respondent:** I will tell my mother and the mother group tha teaches us.

**Interviewer:** Why will you tell these and not your friends?

**Respondent:** If I tell my friends they will tell other people and it will be a shame to me, when you tell friends stories they tell other people so I will be ashamed of myself.

**Interviewer:** What about the issues we discussed about HIV and pregnancy, what questions do you have about what people talk about you don’t understand them?

**Respondent:** My question is , Is it good to do that while you are still young?

**Interviewer:** Mmmh that is the question

**Respondent:** Yes

**Interviewer:** Another question?

**Respondent:** That is the only question I have.

**Interviewer:** That is the only question?

**Respondent:** Yes

**Interviewer:** How often….. boy-girl relationships in this area?

**Respondent:** A lot of them, the young can be forced to engage into a relationship like my age, we can be forced into a relationship that we don’t want.

**Interviewer:** Have you ever been forced?

**Respondent:** No

**Interviewer:** you have never been forced but have you seen other people being forced?

**Respondent:** Yes

**Interviewer:** Why do they do that? Why do girls engage into relationships while they are still young?

**Respondent:** The reasons?

**Interviewer:** Yes

**Respondent:** Some just want to have baby and they don’t want school anymore. What they want is a baby.

**Interviewer:** Mmmh ok, do you think this is good or not?

**Respondent:** It’s not good

**Interviewer:** Why is it not good?

**Respondent:** Because school is good, dropping out of school isn’t good.

**Interviewer:** School I good, ok. What position were you?

**Respondent:** Me

**Interviewer:** Yes, last term

**Respondent:** 16

**Interviewer:** you were position 16?

**Respondent:** Yes

**Interviewer:** Mmm ok, what made you to join GG club?

**Respondent:** I wanted to know more

**Interviewer:** To know more about what?

**Respondent:** About HIV/AIDS and to know each other

**Interviewer:** To know each other?

**Respondent:** Yes, when you are at a group you are supposed to know each other

**Interviewer:** Mmh, what is relevance of knowing each in a group?

**Respondent:** Someone can be absent and the group can know who is absent.

**Interviewer:** The other thing that you have benefitted from GG Club?

**Respondent:** About my studies and to know more about my body, they gave us bags and notebooks.

**Interviewer:** What do you mean when you say to know my body?

**Respondent:** We should avoid sexual intercourse

**Interviewer:** Ok, who told you to join GG Club?

**Respondent:** Ourselves, they just wrote our names.

**Interviewer:** Where did the find you?

**Respondent:** They just wrote our names

**Interviewer:** Who wrote your names?

**Respondent:** He/she didn’t tell us

**Interviewer:** Ok, what can you tell a friend who want to join GG Club?

**Respondent:** I can tell her to join so that we can learn more

**Interviewer:** You can encourage her to join?

**Respondent:** Yes

**Interviewer:** Why would you encourage her to join?

**Respondent:** To know about her body and the importance of school.

**Interviewer:** How important it is to know about your body? Is it important?

**Respondent:** Yes

**Interviewer:** Why is it important to know about your body?

**Respondent:** So that she doesn’t engage into relationships

**Interviewer:** She should know about her body so that she doesn’t engage into relationships?

**Respondent:** Yes, she should end the relationships while in the club.

**Interviewer:** she should have relationships while in the group?

**Respondent:** No, she should not have but she should join the group to learn.

**Interviewer:** Ok, Thank you very much for responding to my questions, do you have any question?

**Respondent:** No

**Interviewer:** You don’t want to ask me any question?

**Respondent:** No

**Interviewer:** Aaah I forgot to ask you something, you said you stay with your mother and father, and your sisters, and how open are you with your father and mother on HIV? Have you ever discussed about HIV/AIDS?

**Respondent:** Yes

**Interviewer:** Tell me what happened for you start discussing with your mother and father?

**Respondent:** To help me that I should not engage in relationships and we should not have sex men.

**Interviewer:** They told you that?

**Respondent:** No

**Interviewer:** Am asking that have you ever discussed with them about this?

**Respondent:** No

**Interviewer:** You have never discussed?

**Respondent:** No

**Interviewer:** If you are given a chance to choose between your mothers, fathers, your brothers and sisters whom can you be open discuss with about HIV?

**Respondent:** My mother

**Interviewer:** Mmmh, your mother?

**Respondent:** Yes

**Interviewer:** Why?

**Respondent:** She can teach me more

**Interviewer:** Your father?

**Respondent:** No

**Interviewer:** Why he cannot teach you more?

**Respondent:** Because we are female while with my father am a girl and he is a man so this not good.

**Interviewer:** What about your sisters?

**Respondent:** I can also open up with them

**Interviewer:** Ok, Thank you so much for answering the questions very well.

**End of interviewer**

**I**
