# Supplementary material for: Social support and very young adolescent girl’s knowledge on sexual relationships: A comparative qualitative study of Girl Only Clubs’ participants and non-participants in rural Malawi
Source: PLOS Glob Public Health. 2023 Jan 12;3(1):e0001339. doi: 10.1371/journal.pgph.0001339 (PMC10022037; doi:10.1371/journal.pgph.0001339)
Supplement: Attachment — Submitted filename: Response to reveiwer comments summary table.docx [file pgph.0001339.s004.docx]

| **Comments from the Editor** | | |
| --- | --- | --- |
| **Comment** | **Description** | **Response** |
|  |  |  |
| 1. | Please update your online Competing Interests statement. If you have no competing interests to declare, please state: “The authors have declared that no competing interests exist.” | Thank you, this has been amended accordingly. |
| 2. | Please amend your detailed online Financial Disclosure statement. This is published with the article. It must therefore be completed in full sentences and contain the exact wording you wish to be published.  Please state what role the funders took in the study. If the funders had no role in your study, please state: “The funders had no role in study design, data collection, and analysis, decision to publish, or preparation of the manuscript.” | Thank you, this has been amended accordingly. |
| 3. | In the Funding Information you indicated that no funding was received. Please revise the Funding Information field to reflect funding received.  Please ensure that the funders and grant numbers match between the Financial Disclosure field and the Funding Information tab in your submission form. Note that the funders must be provided in the same order in both places as well. | Thank you this has been amended accordingly. |
| 4. | "Appendix 1a English IDI Guide_Girlsinclubs.docx" and "Appendix 1b English IDI Guide_Girlsnotinclubs.docx" are currently uploaded as file type “Other”, which is not viewable by the reviewers. Please change the file types to 'Supporting Information' and include a legend in the manuscript if you wish them to be included in review | Thank you this has been amended accordingly. |
| 5. | In the online submission form, you indicated that “The interview data of this study are available from the corresponding author upon reasonable request.”. All PLOS journals now require all data underlying the findings described in their manuscript to be freely available to other researchers, either 1. In a public repository, 2. Within the manuscript itself, or 3. Uploaded as supplementary information. This policy applies to all data except where public deposition would breach compliance with the protocol approved by your research ethics board. If your data cannot be made publicly available for ethical or legal reasons (e.g., public availability would compromise patient privacy), please explain your reasons by return email and your exemption request will be escalated to the editor for approval. Your exemption request will be handled independently and will not hold up the peer review process, but will need to be resolved should your manuscript be accepted for publication. One of the Editorial team will then be in touch if there are any issues. | Thank you, this has been amended accordingly. The data underlying the findings presented in this manuscript have been made available as a supplementary information. |
| **Comments from the first reviewer** | | |
| 6. | **Writing and organization**  I highly suggest having a person not involved with the study to proofread the manuscript for clarity. The authors 1) use acronyms such as SHR, AGYW before explaining them in full; 2) introduce essential concepts such as ‘very young adolescents’, ‘Girls Only Clubs’ without defining them at first use. | We are grateful to the reviewer for this important suggestion and observation. We engaged a colleague who was not involved in the study to proofread the manuscript. We now trust that both the language and the formulation of sentences have improved. Furthermore, we have carefully read the manuscript and corrected the typographical errors that were in the manuscript. |
| 7. | **Writing and organization**  The title is kind of misleading. The study is mostly on the interaction between Girls only clubs and social support and the knowledge young adolescents have about sexual relationships, but not about the actual sexual relationships themselves. Consider making this more specific. | We thank the reviewer for this suggestion. The title has now been changed to:  **Social support and very young adolescent girl’s knowledge on sexual relationships: A comparative qualitative study of Girl Only Clubs’ participants and non-participants in rural Malawi** |
| 8. | **Writing and organization**  I would avoid the opening sentence as it is currently used in the abstract. The claim that ‘there is growing evidence that some are’ is not specific and could be said more convincingly by focusing for instance on changing trends in age of sexual debut or the possible consequences of early sexual relationships on health and wellbeing outcomes. | We are grateful to the reviewer for this suggestion. We have revised the opening sentence as per the reviewer’s suggestion. Please refer to the first sentence in the abstract, line number 21-23 highlighted in yellow. |
| 9. | **Introduction**  Normally the introduction would also include a preview of the methods, findings and possibly a guide to introduce the reader to what to expect in the rest of the study | We appreciate this advice from the reviewer. We have now provided a brief preview of the methods and findingds in the last paragraph of the introduction. Please refer to line number 142 to line 146 highlighted in yellow. |
| 10. | **Introduction**  The underlying theoretical framework is lacking. While I understand that there is limited research available on very young adolescents, social support and sexual relationships, the authors should at least set out the relationship between components that have been covered in the existing literature more clearly, in specific 1) the connection between social support and sexual relationships; 2) very young adolescents and sexual and reproductive health outcomes, 3) girls clubs or similar interventions and social support. I recommend using a logical framework or a theory of change to clearly highlight the pathways in which they expect the Girls Only Clubs and social support to have an influence. Using a framework will also help to clarify how the authors view the interaction between social support and the Girls Only Clubs. | Thanks to the reviewer for this insightful comment. We have now added a theoretical framework in the introduction section. Please refer to the introduction section line number 118 to 141 highlighted in yellow. |
| 11. | **Introduction**  The authors could further strengthen the literature used in the study. While Barker’s literature review is salient to this manuscript, he did not develop the categories on social support. Further review of the original literature, as well as including more diversity in perspectives is needed to strengthen the background section. | Thank you for this important suggestion. We have included additional literature and references in the introduction section. We have highlighted this addition in yellow. Please refer to line number 66-76. |
| 12. | **Intervention**  I would like to see more information on the intervention: the Girls Only Clubs and DREAMS initiative. Information such as how girls were selected for the program, how the information was transferred to the adolescents (e.g. who was leading the clubs, how often would they meet, what were the other benefits from attending). | Thank you for this important suggestion. We have added more information on the intervention in the introduction section. Please refer to page 6, from line number 99 to 110. The added information is highlighted in yellow. |
| 13. | **Methodology**  The justification and explanation for the choice of methodology is insufficient and should be addressed. The authors do not give any reasons for why they chose to assess their research questions using a comparative analysis. They should also add further explanation what the benefits of this methodology is for the study. | We are thankful to the reviewer for this comment and suggestion. We have added more information on the choice of methodology. Please refer to the data analysis section from line number 226 to line 231. We have highlighted the added information in yellow. |
| 14. | **Study setting**  Additional information on the non-Girl Only Club locations and sample is required. Were there other considerations other than the lack of the intervention (e.g. the sampling section suggest that no other HIV programming was present, if this was a consideration for selection it should have been included in ‘study setting’ section. | Thanks to the reviewer for this comment. We have added information on the non-Girl Only Club locations and sample. Please refer to the study setting section line 166-167 and line 172-173 highlighted in yellow. |
| 15. | **Sampling**  Describe how the inclusion criteria were assessed, in particular with regards to ‘the ability to communicate experiences and opinions in an articulate, expressive, and reflective manner’. Also, currently it seems that all 7 girls who were not interviewed were below 12 years. Rephrase this section if this was not the case. | We have now provided more information on how the prospective participants’ ability to communicate experiences and opinions in an articulate, expressive, and reflective manner’ was assessed. Please refer to line number 178-190. We have also rephrased this section to clearly show which ages did not participate in the interviews. |
| 16. | **Data analysis** Add who were involved in coding and what the outcomes of the test for inter-coding agreement was (and if applicable what the adjustments and consequences are). | We are grateful to the reviewer for this suggestion. Further information has been provided on who was involved in the coding and the outcome of the inter-coder agreement. Please refer to the data analysis section line numbers 240 to 243. |
| 17. | **Results**  Even though this is a comparative analysis, the sample sizes are small and this is a descriptive analysis only. For that reason, be cautious about using general statements about Girls Only Club girls being more confident in their knowledge and having more to say (p.13). I recommend focusing more on the type of information, social support received rather than reporting which group received more. If you want to use the frequency, it would be good to be more specific. Use something like ‘x out of 23 Girls Only Club girls expressed being confident about their knowledge, while this was only so for x out of 20 non-Girls Only Club girls’ | Thank you for this important suggestion. The sentence indicating that Girls Only Club girls were more confident in their knowledge has been revised. Please refer to the results section in the information support from GOCs section line 329 to 334 highlighted in yellow. |
| 18. | **Discussion**  •The authors do a good job describing the findings in the discussion and putting them in context. When using additional literature, be careful to clarify why the literature was different (see e.g. line 385 add if the findings from the other DREAMS study are only for older adolescents). | We thank the reviewer for for this suggestion. We have now provided clarification on areas where the literature added in different.. Please refer to the discussion section line number 440-443. |
| 19. | **Discussion**  With regards to the two main implications, the first part of the first implication does not follow directly from the analysis. You are not assessing whether adolescents should get SHR programs at a younger age. You can say that you found that younger adolescents seem to benefit from having access to these programs at their age. It is a subtle difference, but since you are not doing a comparative analysis with older adolescents I find the first part of the implication a bit of a stretch. I agree with the second implication, but please reread it to correct the language | The sentence referred to by the reviewer has been rephrased. Please refer to the discussion section line number 479-479. |
| 20. | **Discussion**  On the limitations, given that the study claims its main strength is the comparative analysis, the authors should also acknowledge any limitations in that respect. E.g. difference in primary caregivers could make for a difference in answers. | Thanks for this suggestion. We have added an additional limitation. Please refer to the discussion section line number 503-505 highlighted in yellow. |
| 21. | **Conclusions** In line 442 and 443 when you mention that there is ‘no difference in SS’, it would be good to be more specific, because currently it contradicts with the rest of the sentence | Thank you for pointing this out, we have rephrased the sentence. Please refer to line 509 to 512 in the conclusion section. |
| **Comments from the second reviewer** | | |
| 22. | Several typos were noted, and some awkward phrasing (especially the paragraph beginning on line 415) made it difficult to read in parts. A thorough proofread would be beneficial. | We are thankful for the reviewer for this suggestion. We have engaged a third party to proofread the entire manuscript. We hope that the reviewers will find the manuscript improved. |
| 23. | Overall, the findings were interesting and analysis appears to be thorough and rigorous. From line 168: a bit more detail in terms of coding and analysis would be helpful - why was the specific methodology (constant comparative analysis) chosen? How was it ensure that translations did not lose their original meanings? | We have added more information on why we chose the constant comparative analysis. Please refer to the data analysis section from line number 226 to line 231. We have highlighted the section in yellow. We have also added information on how we ensured original meanings in transcripts were maintained. Please refer to the data analysis section from line number 218 to line 220. We have highlighted the section in yellow. |
| 24. | Line 215 - the definition of informational SS is a bit vague. Since it's so critical to the findings, expanding on this concept might be helpful to readers. | We have rephrased the definition and included some examples to make it clearer. Please refer to the results section, line number 274-276. |
| 25. | You introduce the concept of social asset building at the end of the paper (around line 415) without explaining or elaborating. Since you also include this as a recommendation for future programming, it would be helpful to explain what it is and why it is so important to programming. | Thank you for this important suggestion. We have added a definition of the asset-building framework and more details about this concept. Please refer to lines 484 to 489. |
| 26. | The findings are very rich and could help to inform future programming. As such, it would be worth considering adding a bit more information on the importance and the challenges of providing SRH education to VYA - e.g., protective factors of confidential adults (club facilitators, parents, etc.) are mentioned, but consider expanding on why these are important. More discussion about the challenges of delivering SRH to VYA who are not yet sexually active (which the majority of your sample appeared not to be), or how this information might have affected this study would also help to bolster recommendations for future studies/programming. | We thank the reviewer for these suggestions. We have expanded our discussion on the significance and challenges of providing SRH education to VYAs. added these details. Please refer to 473-484. |
