# Supplementary material for: Social support and very young adolescent girl’s knowledge on sexual relationships: A comparative qualitative study of Girl Only Clubs’ participants and non-participants in rural Malawi
Source: PLOS Glob Public Health. 2023 Jan 12;3(1):e0001339. doi: 10.1371/journal.pgph.0001339 (PMC10022037; doi:10.1371/journal.pgph.0001339)
Supplement: Attachment — Submitted filename: Response Letter_16.11.2022.docx [file pgph.0001339.s005.docx]

**Response to Editor Letter**

| **Comment** | **Description** | **Response** |
| --- | --- | --- |
| 1 | We note that there is potentially identifying data in the Supporting Information file "SS_VYA_DATA.zip". Due to the inclusion of these potentially identifying data, you have removed this file from your file inventory. | Thank you for this important observation. “We concur that there is potentially identifying data in the Supporting Information file "SS_VYA_DATA.zip. As such now We have ensured that all potentially identifying information has been removed in the data shared. |
| 2 | Prior to sharing human research participant data, authors should consult with an ethics committee to ensure data are shared in accordance with participant consent and all applicable local laws.  Data sharing should never compromise participant privacy. It is therefore not appropriate to publicly share personally identifiable data on human research participants. The following are examples of data that should not be shared:  -Names, initials, physical address -Ages more specific than whole numbers -Internet protocol (IP) address -Specific dates (birth dates, death dates, examination dates, etc.) -Contact information such as phone number or email address -Location data -ID numbers that seem specific (long numbers, include initials, titled “Hospital ID”) rather than random (small numbers in numerical order)  Data that are not directly identifying may also be inappropriate to share, as in combination they can become identifying.   For example, data collected from a small group of participants, vulnerable populations, or private groups should not be shared if they involve indirect identifiers (such as sex, ethnicity, location, etc.) that may risk the identification of study participants.  Additional guidance on preparing raw data for publication can be found in our Data Policy (<https://journals.plos.org/globalpublichealth/s/data-availability#loc-human-research-participant-data-and-other-sensitive-data>) and in the following article: <http://www.bmj.com/content/340/bmj.c181.long>. | Thank you for this important comment. As such now we have ensured that all data shared does not contain any of the information indicated by the editor. |
| 3 | Please remove or anonymize all personal information, in the case of identifying interviews, we encourage you to ensure that the data shared are in accordance with participant consent, and re-upload a fully anonymized data set. Please note that spreadsheet columns with personal information must be removed and not hidden as all hidden columns will appear in the published file. You also have the option to upload the data to an external database (such as figshare or Dryad) and provide us with the relevant access information. | Thank you for this important comment. We have now gone through all transcript and anonymized all information that might be potentially identifying. |
|  |  |  |
|  |  |  |
